# Kinetic Resolution of 2-Aryl-4-methylenepiperidines toward Enantioenriched Functionalizable Piperidine Fragments

**DOI:** 10.1021/acs.joc.2c00862

**Published:** 2022-06-14

**Authors:** Anthony Choi, Anthony J. H. M. Meijer, Ilaria Proietti Silvestri, Iain Coldham

**Affiliations:** † Department of Chemistry, University of Sheffield, Brook Hill, Sheffield S3 7HF, U.K.; ‡ Liverpool ChiroChem, Department of Chemistry, University of Liverpool, Liverpool L69 7ZD, U.K.

## Abstract

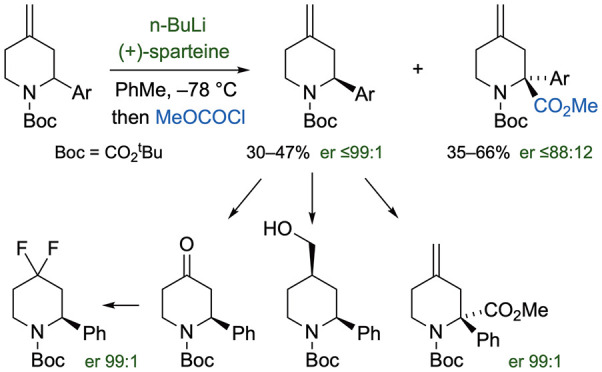

The base *n*-BuLi with sparteine allows a kinetic resolution of *N*-Boc-2-aryl-4-methylenepiperidines. The 2,2-disubstituted products and recovered starting materials were isolated with high enantiomeric ratios. From VT-NMR spectroscopy and DFT studies, the rate of rotation of the *N*-Boc group is fast. Lithiation and trapping of the enantioenriched starting materials gave 2,2-disubstituted piperidines with retention of stereochemistry. Functionalization of the 4-methylene group led to a variety of 2,4-disubstituted piperidines without loss of enantiopurity that could be useful building blocks for drug discovery.

Piperidines are important molecular motifs found in many pharmaceutical drugs and natural products.^1,2^ Examples include glasdegib, which is a drug that has been used in the treatment of acute myeloid leukemia,^3^ and LNP023, which has been developed as a serine protease factor B inhibitor (Figure 1).^4^ Within these structures, an aryl group is present in the 2-position of the piperidine core along with an additional functional group in the 4-position. In other piperidine-based drugs, different functional groups extend from the piperidine core resulting in the presence of various stereogenic centers.^5^ The ability to control the stereochemistry of these stereocenters within a substituted piperidine remains an area of interest in organic synthesis to allow the development of novel, structurally diverse 3D molecules. These molecules are particularly important in the expansion of fragment libraries that are used in screening processes when developing new drugs.^5^ To support the synthesis of these compounds, the use of functionalizable 3D fragments that possess substituents and synthetic handles prove useful as they would allow a large array of compounds to be synthesized in a more timely and cost-efficient manner.

**Figure 1:**
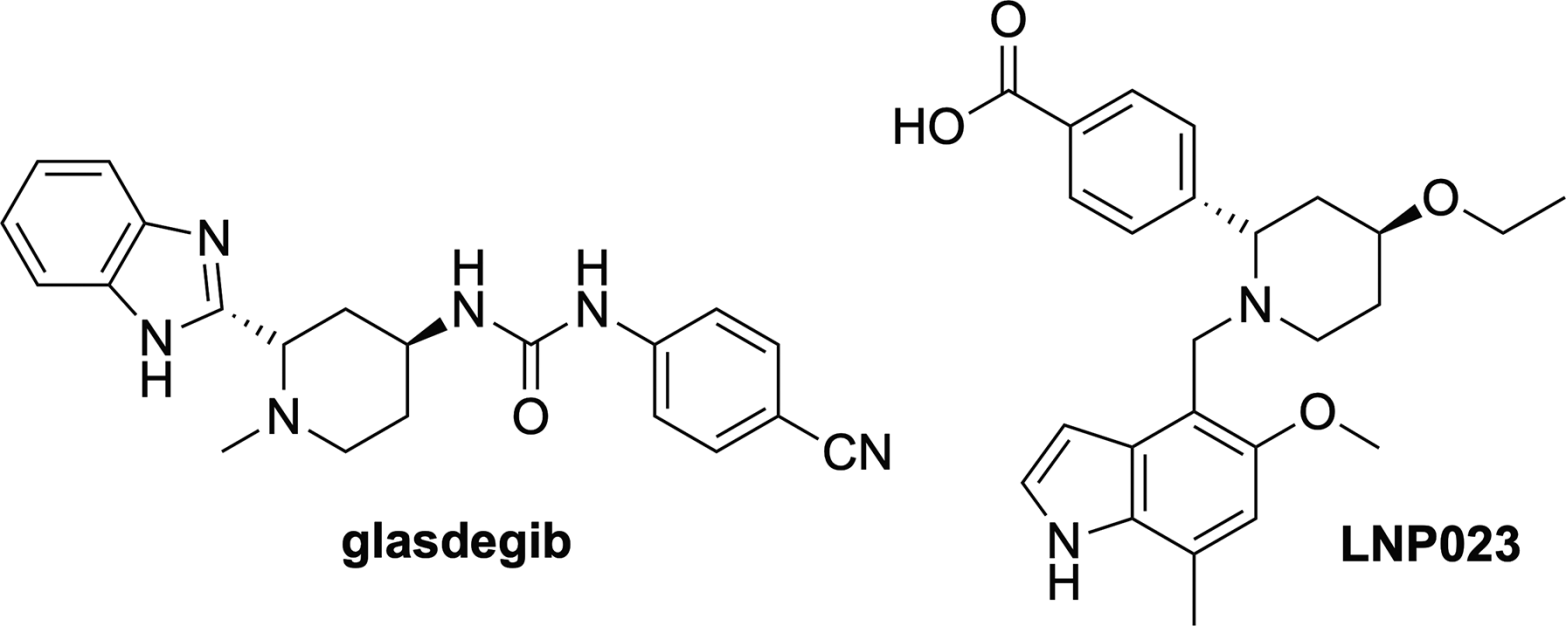
Structures of glasdegib and LNP023.

Previously, we have reported the synthesis of enantiomerically enriched 2-arylpiperidines via kinetic resolution using the chiral base system of *n*-BuLi and (−)-sparteine, which selectively deprotonates one enantiomer of the starting material (Scheme 1, Boc = CO_2_^*t*^Bu).^6^ The resolved 2-arylpiperidines can then be treated with *n*-BuLi and an electrophile to give enantioenriched 2,2-disubstituted compounds without a loss of selectivity. To expand on this research, we wanted to investigate the lithiation of substituted 2-arylpiperidines containing functionalizable groups in the 4-position.^7^ There are limited examples of kinetic resolution of disubstituted piperidines^8,9^ and only one method as far as we are aware for the asymmetric synthesis of 2,4-dialkyl-substituted piperidines by kinetic resolution.^9^ This approach uses a chiral hydroxamic acid to obtain good selectivities, although it was important that the starting diastereoisomeric 2,4-disubstituted piperidine was the *trans*-isomer and the chiral hydroxamic acid needed for the reaction is not available commercially.

**Scheme 1:**
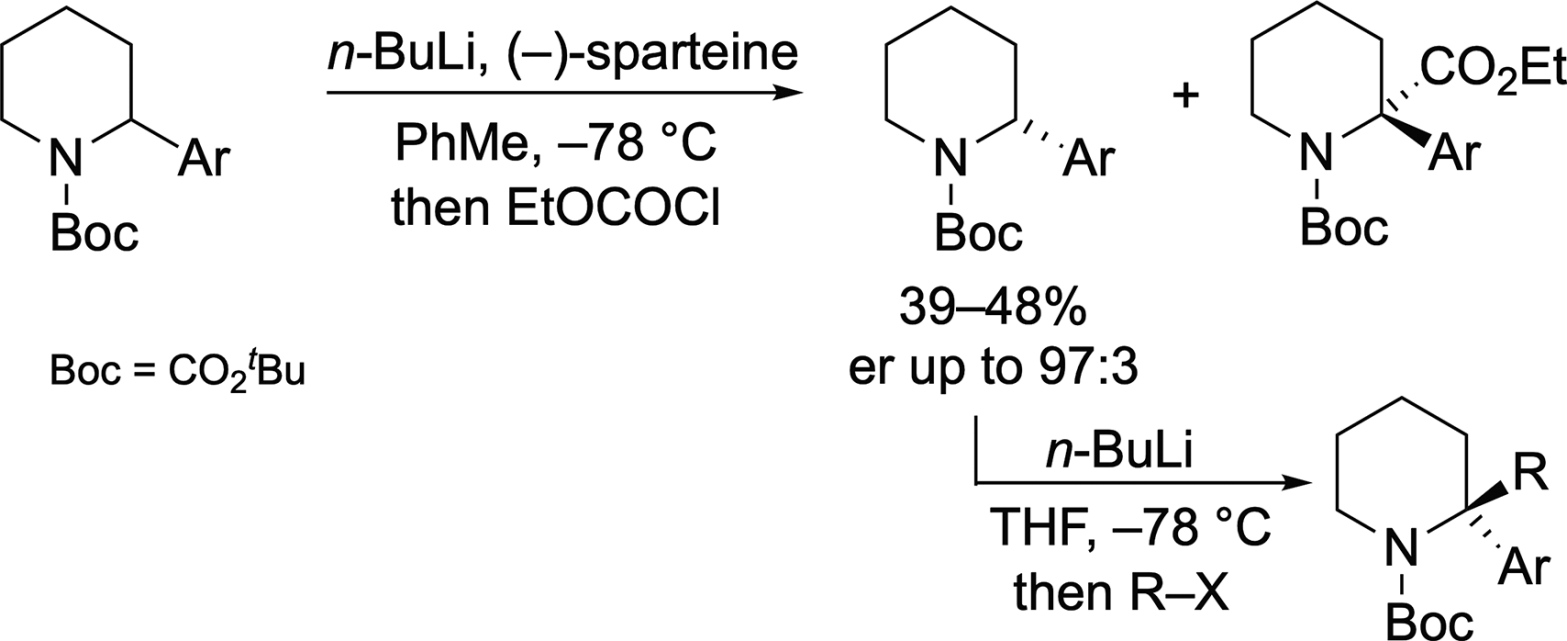
Previous Work on Kinetic Resolution of 2-Arylpiperidines

To apply our chemistry to the formation of a variety of derivatives it was important to ensure the functional group installed in the 4-position was not only inert toward lithiation but also able to undergo further functional group interconversions. We chose to install a methylene group in the 4-position as it meets these criteria. In this paper, we report that *N*-Boc-2-aryl-4-methylenepiperidines are viable substrates for highly selective kinetic resolution by asymmetric deprotonation and can be converted to a selection of 2,4-disubstituted piperidines.

Accessing the 2-aryl-4-methylenepiperidines required for our study was achieved from the enones **1a**–**h**, which could be synthesized using known methods.^10^ Reduction of the enone using L-Selectride gave the 2-aryl-4-piperidones **2a**–**h** which were converted to the desired 4-methylene derivatives **3a**–**h** using a Wittig olefination (Scheme 2).

**Scheme 2:**
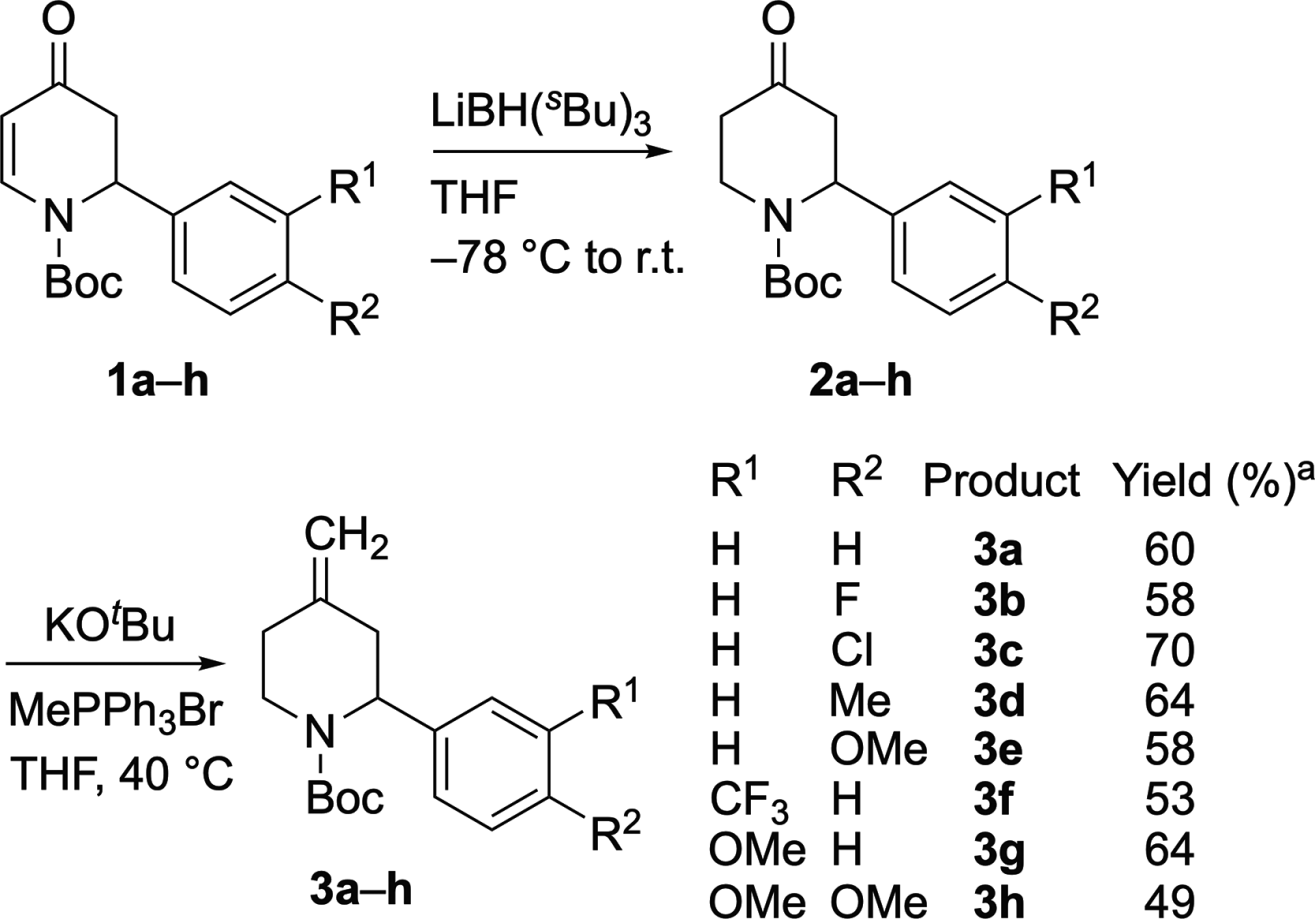
Preparation of *N*-Boc-2-aryl-4-methylenepiperidines Yields quoted over two steps.

Applying knowledge from previous work,^11^ it was pleasing that lithiation of piperidine **3a** occurred smoothly at −40 °C in THF. The organolithium intermediate could be trapped with a variety of electrophiles to give 2,2-disubstituted compounds **4a**–**9a** in 70–90% yields (Scheme 3). Electrophiles screened included methyl chloroformate (to give **4a**), alkyl or allyl halides (to give **5a**–**7a**), Bu_3_SnCl, and Me_3_SiCl (to give **8a**–**9a**).

**Scheme 3:**
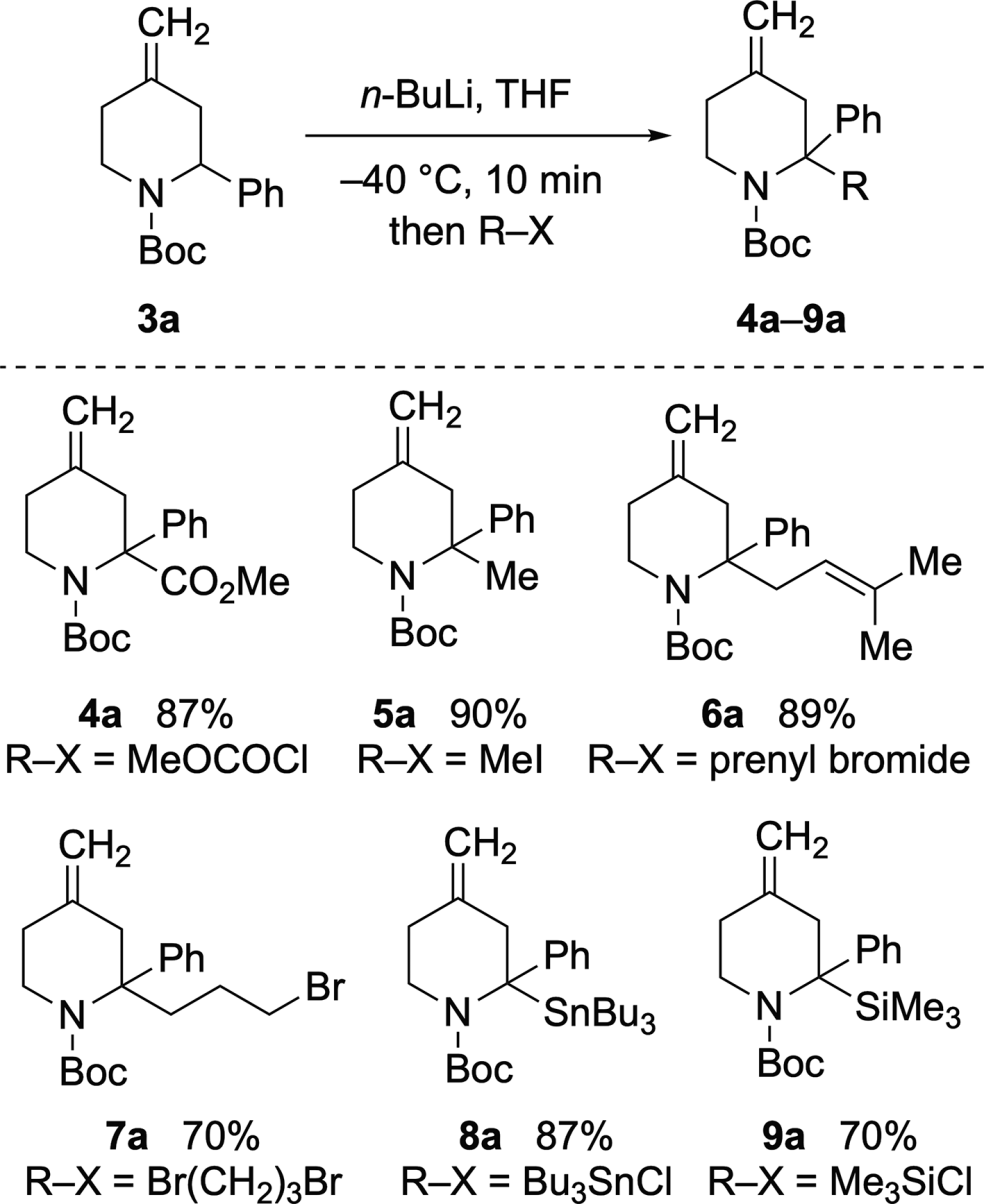
Initial Lithiation–Trapping Studies

The successful formation of products **4a**–**9a** suggests that rotation of the Boc group is rapid under the conditions of the reaction. The base *n*-BuLi is known to coordinate to the carbonyl group prior to lithiation.^12^ Lithiation occurs only from the rotamer in which the carbonyl group is directed toward the benzylic proton. The rate of rotation of the carbonyl was determined by VT-NMR spectroscopy (see the Supporting Information). Coalescence of the signals for the benzylic proton in the ^1^H NMR spectrum at ∼5.5 ppm occurred at about 253 K. Line shape analysis of the mixture of rotamers (ratio ∼1.2:1) showed that the activation parameters for rotation of the Boc group were Δ*H*^⧧^ ≈ 50 kJ/mol and Δ*S*^⧧^ ≈ – 10 J/K/mol, and hence, the half-life for rotation *t*_1/2_ ≈ 10 s at 195 K (Δ*G*^⧧^ ≈ 52 kJ mol^–1^; see the Supporting Information).

DFT calculations on the rotamers of piperidine **3a** were also carried out based on previous work using the B3LYP-D3BJ functional with the def2-TZVP basis set (B3LYP-D3BJ//def2-TZVP).^13−15^ The minimal energy structures for the rotamers of piperidine **3a** were found to be when the phenyl group occupied an axial position (Figure 2a,c). Of these two structures, the rotamer in which the carbonyl group is pointing toward the benzylic proton was lower in thermal energy by ∼170 Jmol^–1^. Transition-state calculations indicated rotation of the Boc group was most likely to occur through the lowest energy equatorial transition state (Figure 2b). The Gibbs energy of activation was calculated to be ∼55 kJ/mol at 195 K (with Δ*H* ≈ 43 kJ/mol and Δ*S* ≈ – 63 J/K/mol). This value matches well with the results obtained from VT-NMR and indicates that the rate of ring flipping between the axial and equatorial positions of the phenyl group was fast along with rotation of the Boc group under the reaction conditions.

**Figure 2:**
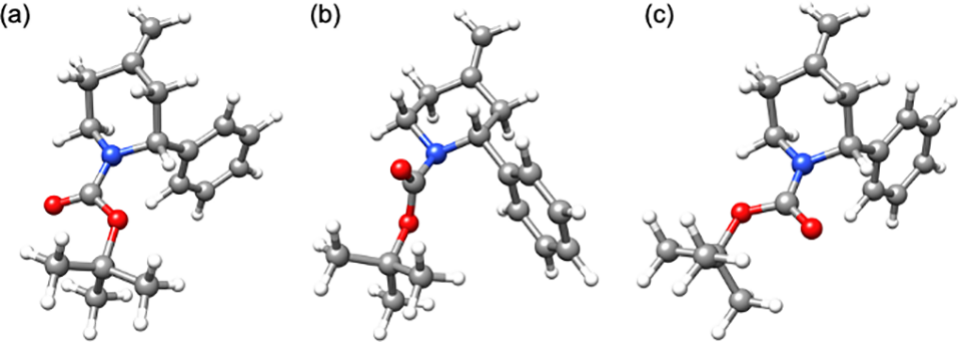
Optimized geometries of **3a** in THF solution. (a,c) Minimum energy structures with the phenyl group in the axial position. (b) Lowest energy transition state with the phenyl group in the equatorial position.

Having obtained the knowledge that rotation of the Boc group is fast and that racemic lithiation–trapping is successful, we began investigating the kinetic resolution of piperidine **3a** using the chiral base system *n*-BuLi/(+)-sparteine. Optimization of the reaction conditions (see the Supporting Information) led to the best results when 0.8 equiv of *n*-BuLi was added to a mixture containing the piperidine **3a** and 0.9 equiv of (+)-sparteine in toluene at −78 °C (Scheme 4). Quenching the reaction after 1 h with MeOCOCl gave the recovered (*S*)-**3a** in 41% yield with an excellent enantiomer ratio (er 97:3) together with the substituted product (*R*)-**4a** (58%, er 73:27). This equates to a selectivity factor *s* ≈ 16.

**Scheme 4:**
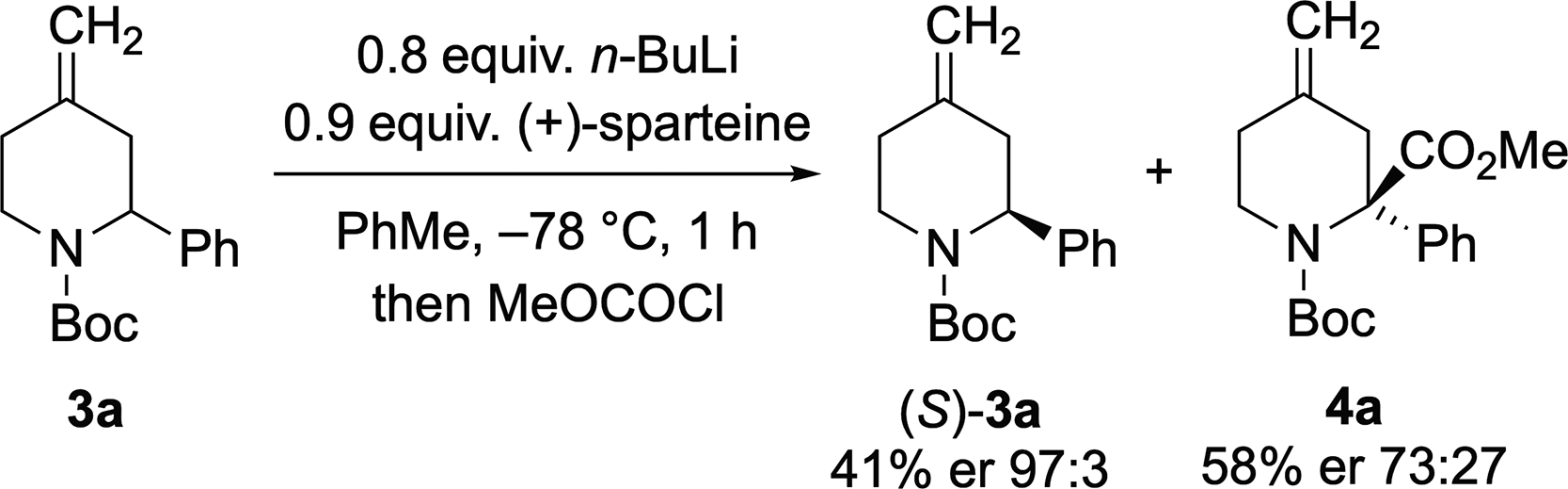
Optimized Kinetic Resolution of **3a**

These initial optimized conditions for piperidine **3a** were then applied to other 2-aryl-4-methylenepiperidines to obtain similar results (Scheme 5). The kinetic resolution reaction tolerated a variety of functional groups in the *meta* or *para* position of the 2-aryl ring including electron-donating and -withdrawing groups (Me, OMe, F, Cl, CF_3_). However, limitations of the reaction could be observed with the methoxy-substituted substrates (**3e**, **3g**–**h**) where more equivalents of *n*-BuLi and (+)-sparteine were required (1.0 and 1.1 equiv, respectively) to achieve good enantiomer ratios of the recovered 2-aryl-4-methylenepiperidines. A possible explanation for this is that some of the chiral base may coordinate to the oxygen atom in the methoxy group; hence, more equivalents of the base were required to ensure adequate deprotonation. By applying this revised method, and sometimes by increasing the lithiation time to 2 h, the recovered (*S*)-**3e**, **3g**–**h** were isolated with good enantiomer ratios although typically with slightly lower yields.

**Scheme 5:**
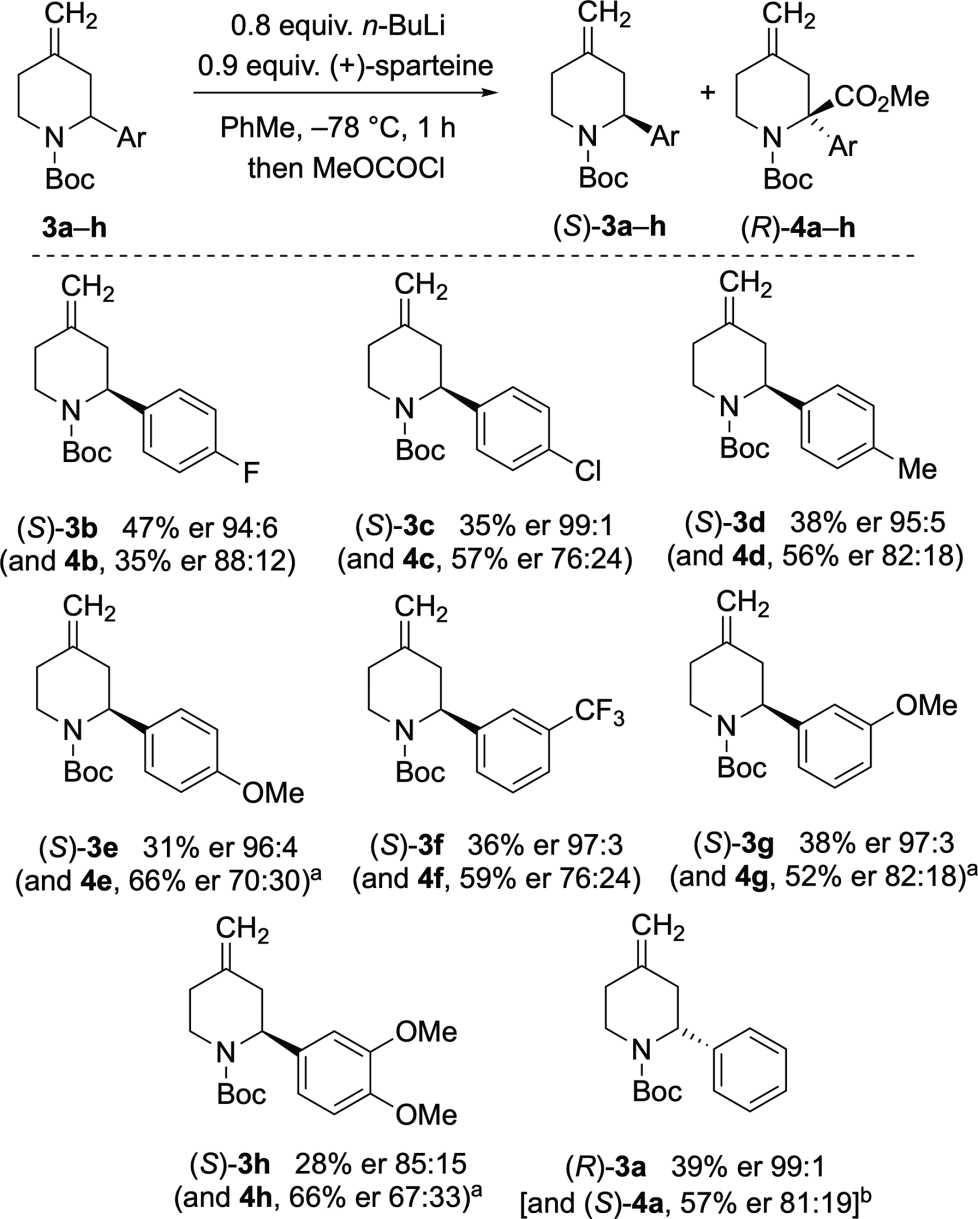
Scope of Kinetic Resolution Using 1.0 equiv of *n*-BuLi, 1.1 equiv of (+)-sparteine (and 2 h for **3e** and **3h**). Using (−)-sparteine.

In addition to changing the aryl group in the 2-position, the scope of the kinetic resolution reaction could be expanded further. By using the chiral ligand (−)-sparteine the enantioselectivity of the reaction could be inverted. This allowed formation of the recovered starting material (*R*)-**3a** with good selectivity, along with the substituted product (*S*)-**4a** (Scheme 5). The optimized reaction conditions for the kinetic resolution of piperidines **3a** and **3b** were tested at larger (gram) scales (Scheme 6). In both reactions, by applying an acid–base wash on the crude products, the chiral ligand (+)-sparteine could be recovered in good yields. After column chromatography, the recovered starting materials (*S*)-**3a** and (*S*)-**3b** were isolated in good yields and selectivities along with their corresponding quenched products.

**Scheme 6:**
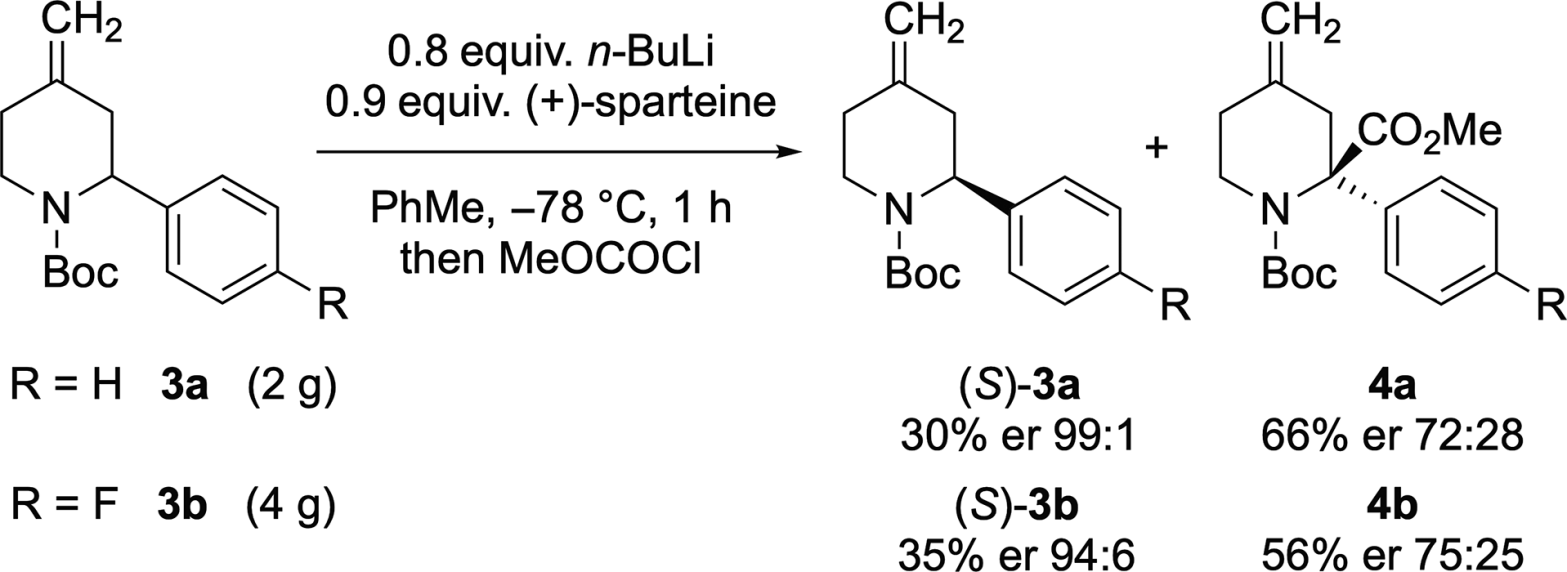
Scale-up of the Kinetic Resolution

Having investigated the scope of the kinetic resolution reaction with various *N*-Boc-2-aryl-4-methylenepiperidines we sought to illustrate their use as potential fragments in organic synthesis. To explore this possibility, several reactions were carried out on piperidine (*S*)-**3a** (er 99:1) to generate a diverse range of compounds without loss of enantiopurity. Lithiation of piperidine (*S*)-**3a** with *n*-BuLi in THF at −78 °C followed by trapping with MeOCOCl gave ester (*S*)-**4a** and maintained the high enantioenrichment (er 99:1) (Scheme 7) due to the configurationally stable intermediate organolithium species.^11^ Removal of the Boc group in piperidine (*S*)-**3a** was achieved using HCl in dioxane, which gave piperidine (*S*)-**10** as the hydrochloride salt. The same reaction on piperidine (*S*)-**3b** (er 94:6) gave (*S*)-**11** as the hydrochloride salt which was recrystallized. Subsequent reattachment of the Boc group to give (*S*)-**3b** followed by chiral stationary-phase HPLC confirmed an improvement in the enantiomeric ratio (er 99:1) (Scheme 8).

**Scheme 7:**
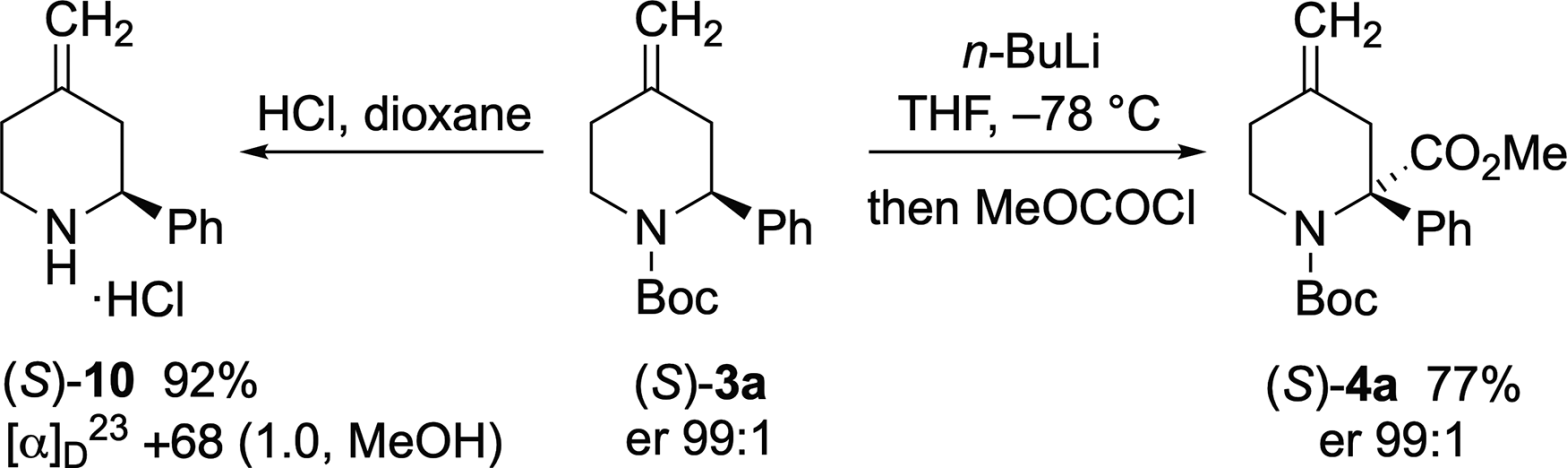
Lithiation–Trapping of (*S*)-**3a** and Removal of Boc

**Scheme 8:**
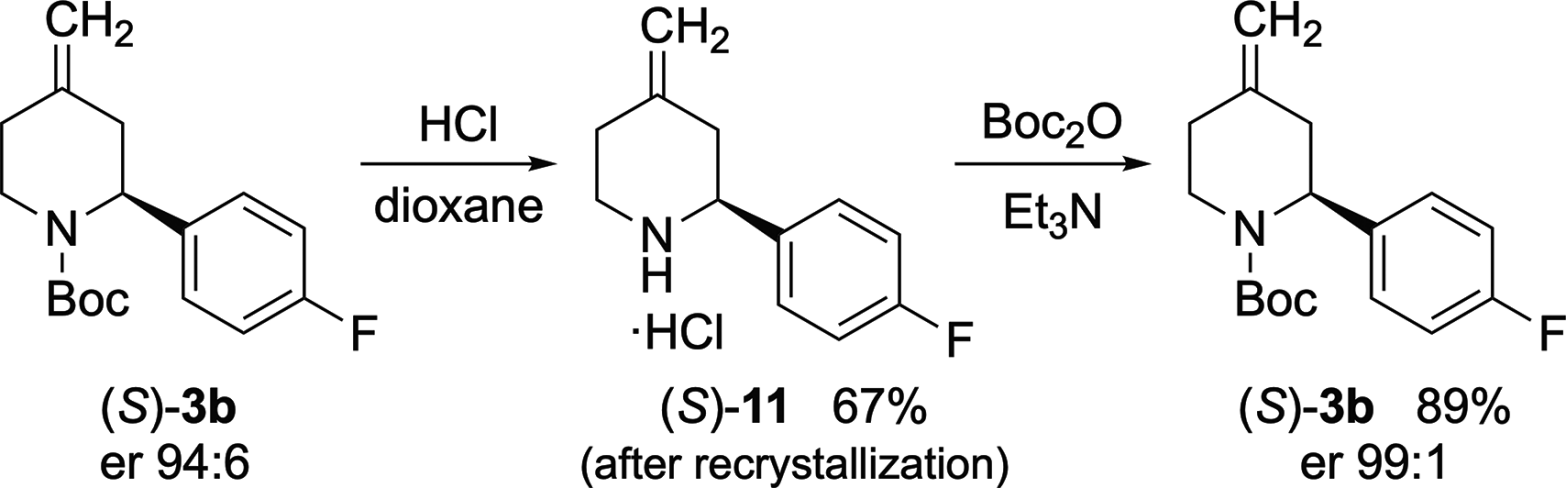
Improving the Enantiomeric Ratio of (*S*)-**3b**

In addition to these transformations, functionalization of the 4-methylene group in piperidine (*S*)-**3a** was investigated. Hydroboration of the alkene was carried out using BH_3_·THF, and the intermediate organoborane was oxidized to give alcohol **11** as a single diastereoisomer (dr 99:1) in 67% yield (Scheme 9). This was subsequently reacted with *p*-bromobenzoyl chloride in the presence of triethylamine and DMAP, and the absolute configuration of the ester product was determined by single- crystal X-ray analysis (Figure 3). This confirmed the *cis*-stereochemistry of **12** and that the piperidine **3a** had the 2*S* configuration, as expected based on the use of (+)-sparteine.^16,17^ Alternatively, ozonolysis of the 4-methylene group in piperidine (*S*)-**3a** was carried out at −78 °C, and subsequent reductive workup using Me_2_S gave the enantioenriched 2-aryl-4-piperidone (*S*)-**2a** in high yield. This could be reacted with DAST to give difluoride (*S*)-**14** without any loss in enantiopurity. Furthermore, this compound was found to be stable toward lithiation using *n*-BuLi in THF at −78 °C. Trapping the organolithium intermediate with methyl chloroformate gave the ester (*S*)-**15** without loss of enantiopurity.

**Scheme 9:**
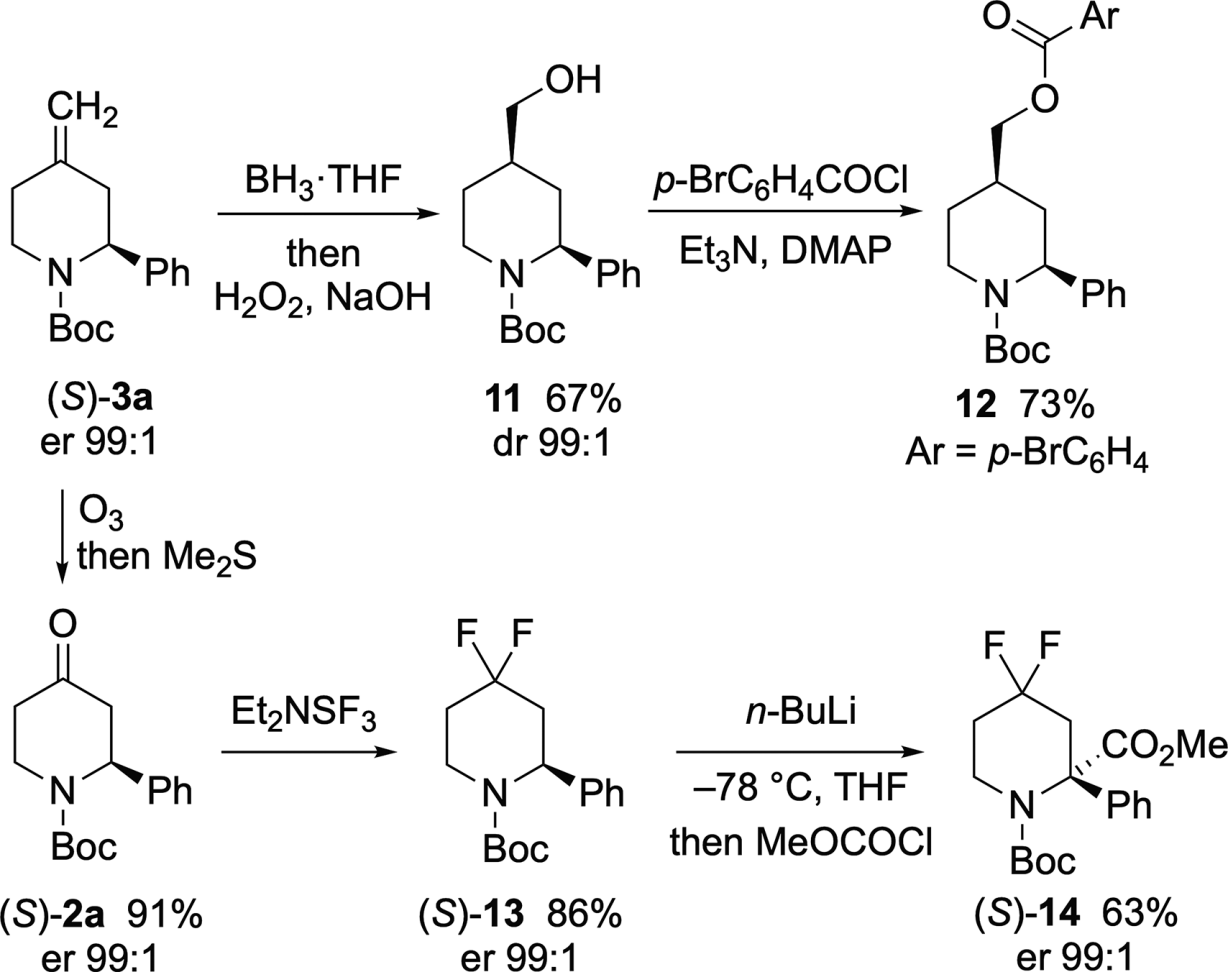
Alkene Functionalization of (*S*)-**3a**

**Figure 3:**
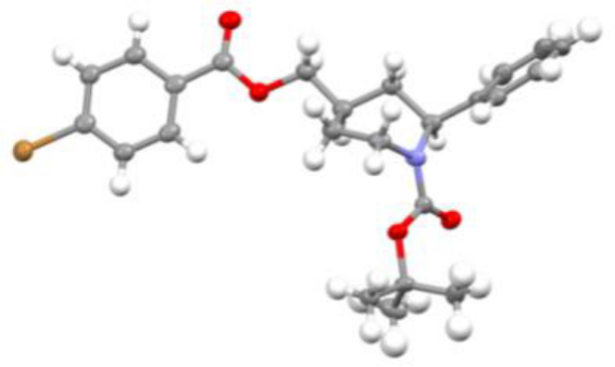
X-ray analysis of *p*-bromobenzoate **12** (ellipsoids at 50% probability).

In summary, kinetic resolution by deprotonation of 2-arylpiperidines can be extended to their 4-methylene derivatives. High enantiomer ratios can be obtained by proton abstraction preferentially from one enantiomer of the substrates using the base *n*-BuLi and the chiral ligand sparteine (either enantiomer). The *N*-Boc group was found to rotate rapidly under the reaction conditions, and therefore, the presence of both rotamers is not detrimental to the lithiation chemistry. Functional group interconversion of the 4-methylene group provides access to a selection of different substituted piperidines. The ability to achieve an effective kinetic resolution allows the asymmetric synthesis of 2,4-disubstituted and 2,2,4-trisubstituted piperidines.

## Supporting Information Available

Experimental procedures; characterization data; NMR spectra; HPLC traces; X-ray crystallographic data; VT-NMR spectra and analysis; DFT analyses with Cartesian coordinates (PDF)

## Accession Codes

CCDC 2155914 contains the supplementary crystallographic data for this paper. These data can be obtained free of charge via www.ccdc.cam.ac.uk/data_request/cif, or by emailing data_request@ccdc.cam.ac.uk, or by contacting The Cambridge Crystallographic Data Centre, 12 Union Road, Cambridge CB2 1EZ, UK; fax: +44 1223 336033.
