# Supplementary material for: Kinetic Resolution of 2-Aryl-4-methylenepiperidines toward Enantioenriched Functionalizable Piperidine Fragments
Source: J Org Chem. 2022 Jun 14;87(13):8819–23. doi: 10.1021/acs.joc.2c00862 (PMC9490820; doi:10.1021/acs.joc.2c00862)
Supplement: Supplementary file 1 — jo2c00862_si_001.pdf [file jo2c00862_si_001.pdf]

Supporting Information

Kinetic Resolution of 2-Aryl-4-methylenepiperidines towards Enantioenriched Functionalisable Piperidine Fragments

Anthony Choi, Anthony J. H. M. Meijer, Ilaria Proietti Silvestri, and Iain Coldham*

Contents

1. General procedures S-2

2. Experimental procedures and characterization data S-4

   2.1 General procedures S-4

   2.2 Preparation of enones **1b**–**h** S-6

   2.3 Preparation of 2-aryl-4-piperidones **2a**–**h** S-11

   2.4 Preparation of 2-aryl-4-methylenepiperidines **3a**–**h** S-17

   2.5 Preparation of racemic 2,2-disubstituted products S-25

   2.6 Kinetic resolution of racemic substrates **3a**–**h** S-37

   2.7 Further functionalization of **3a** and (*S*)-**3a** S-48

3. References S-58

4. ^{1}H, ^{19}F and ^{13}C{1H} NMR spectra S-60

5. HPLC traces S-107

6. X-ray data for ester (2*S*,4*R*)-**13** S-133

7. Variable temperature ^{1}H NMR spectra for carbamate **3a** S-140

8. DFT data S-143

1. General procedures

Reagents were obtained from commercial suppliers and were used without further purification or after distillation; *n*-BuLi was titrated before use. Solvents were obtained from a Grubbs dry solvent system. *tert*-Butyl 4-oxopyridine-1(4*H*)-carboxylate and compound **1a** were synthesised using the methods reported by Dieter and co-workers.[1] $LiBH(^{s}Bu)_3$ was obtained as a solution of L-Selectride® (1 M in THF) from Sigma Aldrich. Thin layer chromatography was performed on Merck silica gel 60F254 plates and visualised by UV irradiation at 254 nm or by staining with an alkaline KMnO4 dip. Flash column chromatography was performed using DAVISIL or Geduran silica gel (40-63 micron mesh). Melting points were recorded on a Gallenkamp hot stage and were uncorrected. InfraRed spectra were recorded on a Perkin Elmer Spectrum RX Fourier Transform – IR System and only selected peaks are reported. ^{1}H NMR spectra were recorded on a Bruker AC400 (400 MHz) instrument. Chemical shifts are reported in ppm with respect to the residual solvent peaks, with multiplicities given as s = singlet, d = doublet, t = triplet, q = quartet, m = multiplet, br = broad. Coupling constants (J values) are quoted to the nearest 0.5 Hz with values in Hertz (Hz). $^{13}C\{1H\}$ NMR spectra (proton-decoupled) were recorded on the above instrument at 100 MHz. Low and high resolution (accurate mass) mass spectra were recorded on a Walters LCT instrument for Electro–Spray (ES).

Intensity data for X-ray crystal structures were collected at 100 K on a Bruker D8 Venture diffractometer using a Cu Kα microfocus X-ray source. Suitable crystals were mounted on a MiTiGen microloop using fomblin oil and transferred directly to the cold nitrogen stream at 100 K for data collection on a Bruker D8 VENTURE diffractometer equipped with an Oxford 700+ cryostream, a PHOTON 100 CMOS detector and using Cu-Kα micro-focus X-ray source. Intensity data was collected in shutterless mode with a final fast scan collected at lower incident beam intensity to enable correction for any detector saturation for low-angle data. Data

reduction was performed using the Bruker Apex3 software.[1] Intensity data were corrected for absorption using empirical methods (SADABS) based upon symmetry equivalent reflections combined with measurements at different azimuthal angles.[2] The crystal structure was solved using ShelXT[3] and refined against all F^2 values using the SHELXL[4] accessed via the Olex2 program.[5] Non-hydrogen atoms were refined anisotropically. Hydrogen atoms were placed in calculated positions with idealized geometries and then refined by employing a riding model and isotropic displacement parameters.

The selectivity factors (S) in the manuscript were calculated using the formula

$$S = \ln[(1-C)(1-ee)] / \ln[(1-C)(1+ee)]$$

where C is the conversion based on the amount of recovered starting material (e.g. for 46% recovered starting material, C = 0.54) and ee is the enantiomeric excess of the recovered starting material (e.g. enantiomer ratio, er 90:10 is 80% ee, so ee = 0.8).

All calculations were performed using density functional theory, employing the B3LYP[6] functional as implemented in the D.01 version of Gaussian 09.[7] Calculations included dispersion corrections using the GD3-BJ[8] method. Calculations used the def2TZVP[9] basis set. Solvent was included via the PCM method[10] as implemented in Gaussian with the default parameters for THF.

2. Experimental Procedures and Characterization Data

2.1 General Procedures

General Procedure A: Synthesis of enones 1b–h

Following methods reported by Dieter and co-workers.[11]

To a solution of aryl magnesium halide (1.2 equiv.) [PhMgCl (2 M in THF) or prepared from refluxing aryl bromide with magnesium turnings (1:1 molar ratio) in THF (2 M) for 30 min and cooling to rt] at –78 °C was added a mixture of *tert*-Butyl 4-oxopyridine-1(4*H*)-carboxylate (1 equiv.) and Me_3SiCl (3 equiv.) in THF (0.33 M). The mixture was warmed to rt over 16 h and quenched with saturated aq NH_4Cl. The mixture was diluted with water followed by Et_2O and the layers were separated. The organic layer was dried over anhydrous $MgSO_4$, filtered and the solvent was evaporated to give the crude product. The crude product was purified by column chromatography on silica gel to give the enone product.

General Procedure B: Synthesis of 2-aryl-4-piperidones 2a–h

To a solution of enone **1a**–**h** (1 equiv.) in THF (0.2 M) under an argon atmosphere at –78 °C was added $LiBH(^sBu)_3$ (1.1 equiv., 1 M in THF). After 2 h, the mixture was warmed to rt and quenched with quenched with saturated aq $NaHCO_3$. The mixture was diluted with water followed by Et_2O and the layers were separated. The organic layer was dried over anhydrous $MgSO_4$, filtered and the solvent was evaporated to give the crude product. The crude product was purified by column chromatography on silica gel to give the 2-aryl-4-piperidone product.

General Procedure C: Synthesis of 2-aryl-4-methylenepiperidines 3a–h

To a suspension of $MePPh_3Br$ (4 equiv.) in THF was added KO^tBu (4 equiv.). The mixture was heated at 40 °C for 30 min and cooled to rt. 2-Aryl-4-piperidone **2a–h** (1 equiv.) in THF was added dropwise and the mixture was heated (stirrer hotplate and drysyn block) at 40 °C for 2–3 h. Upon cooling to rt, the mixture was diluted with water followed by Et_2O and the layers were separated. The organic layer was dried over anhydrous $MgSO_4$, filtered and the solvent was evaporated to give the crude product. The crude product was purified by column chromatography on silica gel to give the 2-aryl-4-methylenepiperidine product.

General Procedure D: Lithiation-trapping of 2-aryl-4-substituted-piperidines

To a solution of 2-aryl-4-substituted-piperidine (1 equiv.) in THF (0.25 M) under an argon atmosphere was added *n*-BuLi (1.2 equiv., 2.0–2.4 M in hexanes) at –40 °C or –78 °C. After 10 min the electrophile (2.0–3.5 equiv.) was added and the mixture was warmed to rt over 16 h. The reaction was quenched with MeOH (1 mL) and the solvent was evaporated to give the crude product.

2.2 Preparation of enones 1b–h

tert-Butyl 2-(4-Fluorophenyl)-4-oxo-3,4-dihydropyridine-1(2*H*)-carboxylate **1b**

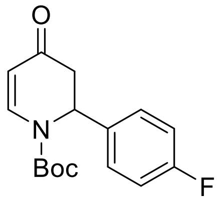

Using general procedure A, magnesium (0.15 g, 6.1 mmol), 1-bromo-4-fluorobenzene (0.68 mL, 6.1 mmol), *tert*-butyl 4-oxopyridine-1(4*H*)-carboxylate (1 g, 5.1 mmol) and Me_3SiCl (2.0 mL, 15 mmol) in THF (15 mL) gave the crude product. Purification by column chromatography on silica gel, eluting with CH_2Cl_2–MeOH (49:1) gave enone **1b** (1.4 g, 94%) as a white amorphous solid; mp 98–100 °C [CH_2Cl_2–MeOH] (no lit. mp reported); R_f 0.63 [CH_2Cl_2−MeOH (19:1)]; ^{1}H NMR ($CDCl_3$, 400 MHz) δ = 7.96 (d, 1H, *J* = 8.0 Hz), 7.26–7.17 (m, 2H), 7.08–6.96 (m, 2H), 5.66 (d, 1H, *J* = 8.0 Hz), 5.38 (d, 1H, *J* = 8.0 Hz), 3.16 (dd, 1H, *J* = 16.5, 8.0 Hz), 2.76 (d, 1H, *J* = 16.5 Hz), 1.49 (s, 9H); ^{13}C{1H} NMR ($CDCl_3$, 100 MHz) δ = 191.9, 162.3 (d, *J* = 247.0 Hz), 151.3, 142.8, 134.8, 127.63 (d, *J* = 8.0 Hz), 115.7 (d, *J* = 21.5 Hz), 107.0, 83.9, 55.1, 41.9, 28.0; ^{19}F NMR ($CDCl_3$, 377 MHz) δ = −114.31; Data as reported.[12]

tert-Butyl 2-(4-Chlorophenyl)-4-oxo-3,4-dihydropyridine-1(2*H*)-carboxylate **1c**

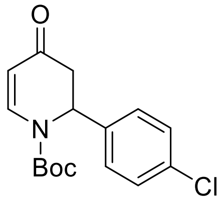

Using general procedure A, magnesium (0.15 g, 6.1 mmol), 1-bromo-4-chlorobenzene (1.2 g, 6.1 mmol), *tert*-butyl 4-oxopyridine-1(4*H*)-carboxylate (1 g, 5.1 mmol) and Me_3SiCl (2.0 mL, 15 mmol) in THF (15 mL) gave the crude product. Purification by column chromatography on

silica gel, eluting with CH_2Cl_2–MeOH (49:1) gave enone **1c** (1.2 g, 76%) as a white amorphous solid; mp 106–108 °C [CH_2Cl_2–MeOH] (no lit. mp reported); R_f 0.68 [CH_2Cl_2–MeOH (19:1)]; ^{1}H NMR ($CDCl_3$, 400 MHz) δ = 7.97 (d, 1H, J = 7.5 Hz), 7.36–7.24 (m, 2H), 7.22–7.13 (m, 2H), 5.65 (d, 1H, J = 7.5 Hz), 5.38 (d, 1H, J = 7.5 Hz), 3.17 (dd, 1H, J = 16.5, 7.5 Hz), 2.75 (d, 1H, J = 16.5 Hz), 1.49 (s, 9H); ^{13}C{1H} NMR ($CDCl_3$, 100 MHz) δ = 191.7, 151.3, 142.8, 137.4, 133.8, 129.0, 127.3, 107.0, 84.0, 55.1, 41.7, 28.0; Data as reported.[12]

tert-Butyl 2-(4-Methylphenyl)-4-oxo-3,4-dihydropyridine-1(2*H*)-carboxylate **1d**

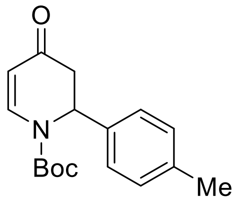

Using general procedure A, magnesium (0.15 g, 6.1 mmol), 1-bromo-4-methylbenzene (1 g, 6.1 mmol) dropwise, *tert*-butyl 4-oxopyridine-1(4*H*)-carboxylate (1 g, 5.1 mmol) and Me_3SiCl (2.0 mL, 15 mmol) in THF (15 mL) gave the crude product. Purification by column chromatography on silica gel, eluting with CH_2Cl_2–MeOH (49:1) to give enone **1d** (1.3 g, 89%) as a clear oil; R_f 0.65 [CH_2Cl_2–MeOH (19:1)]; ^{1}H NMR ($CDCl_3$, 400 MHz) δ = 7.96 (d, 1H, J = 7.5 Hz), 7.18–7.03 (m, 4H), 5.65 (d, 1H, J = 7.5 Hz), 5.36 (d, 1H, J = 7.5 Hz), 3.14 (dd, 1H, J = 16.5, 7.5 Hz), 2.79 (d, 1H, J = 16.5 Hz), 2.33 (s, 3H), 1.49 (s, 9H); ^{13}C{1H} NMR ($CDCl_3$, 100 MHz) δ = 192.4, 151.5, 142.9, 137.6, 135.8, 129.4, 125.8, 107.0, 83.7, 55.4, 41.9, 28.0, 21.0; Data as reported.[13]

tert-Butyl 2-(4-Methoxyphenyl)-4-oxo-3,4-dihydropyridine-1(2*H*)-carboxylate **1e**

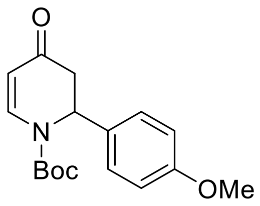

Using general procedure A, magnesium (0.15 g, 6.1 mmol), 1-bromo-4-methoxybenzene (0.76 mL, 6.1 mmol), *tert*-butyl 4-oxopyridine-1(4*H*)-carboxylate (1 g, 5.1 mmol) and Me_3SiCl (2.0 mL, 15 mmol) in THF (15 mL) gave the crude product. Purification by column chromatography on silica gel, eluting with CH_2Cl_2–MeOH (49:1) gave enone **1e** (1.5 g, 97%) as a clear oil; R_f 0.69 [CH_2Cl_2–MeOH (19:1)]; ^{1}H NMR ($CDCl_3$, 400 MHz) δ = 7.94 (d, 1H, *J* = 7.5 Hz), 7.17 (d, 2H, *J* = 8.5 Hz), 6.84 (d, 2H, *J* = 8.5 Hz), 5.64 (d, 1H, *J* = 7.5 Hz), 5.37 (d, 1H, *J* = 7.5 Hz), 3.79 (s, 3H), 3.14 (dd, 1H, *J* = 16.5, 7.5 Hz), 2.77 (d, 1H, *J* = 16.5 Hz), 1.49 (s, 9H); ^{13}C{1H} NMR ($CDCl_3$, 100 MHz) δ = 192.5, 159.2, 151.5, 142.9, 131.0, 127.2, 114.1, 106.9, 83.7, 55.3, 55.1, 41.9, 28.0. Data as reported.[13]

tert-Butyl 2-(4-Trifluoromethylphenyl)-4-oxo-3,4-dihydropyridine-1(2*H*)-carboxylate **1f**

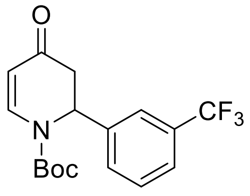

Using general procedure A, magnesium (0.15 g, 6.1 mmol), 1-bromo-4-trifluoromethylbenzene (0.85 mL, 6.1 mmol), *tert*-butyl 4-oxopyridine-1(4*H*)-carboxylate (1 g, 5.1 mmol) and Me_3SiCl (2.0 mL, 15 mmol) in THF (15 mL) gave the crude product. Purification by column chromatography on silica gel, eluting with CH_2Cl_2–MeOH (49:1) gave enone **1f** (1.7 g, 98%) as a pale yellow oil; R_f 0.56 [CH_2Cl_2–MeOH (19:1)]; FT-IR ν_{max} (film)/cm^{-1} 1723 (C=O), 1668

(C=O), 1605 (C=C); ^{1}H NMR ($CDCl_3$, 400 MHz) δ = 8.01 (d, 1H, *J* = 8.5 Hz), 7.62–7.51 (m, 1H), 7.50–7.39 (m, 3H), 5.72 (d, 1H, *J* = 7.5 Hz), 5.41 (d, 1H, *J* = 8.5 Hz), 3.21 (dd, 1H, *J* = 16.5, 7.5 Hz), 2.81 (d, 1H, *J* = 16.5 Hz), 1.49 (s, 9H); ^{13}C{1H} NMR ($CDCl_3$, 100 MHz, one quaternary carbon not observed) δ = 191.4, 151.2, 142.8, 140.0, 131.61–130.85 (m), 129.5, 129.0, 125.07–124.64 (m), 123.19–122.59 (m), 107.2, 84.3, 55.3, 41.5, 27.9; ^{19}F NMR ($CDCl_3$, 377 MHz) δ = −62.78; HRMS (ESI-TOF) *m/z*: $[M+Na]^+$ Calcd for $C_{17}H_{18}F_3NO_3Na$ 364.1131; Found 364.1134; LRMS *m/z* (ES) 286 (100%), 364 (20%, MNa^+).

tert-Butyl 2-(3-Methoxyphenyl)-4-oxo-3,4-dihydropyridine-1(2*H*)-carboxylate **1g**

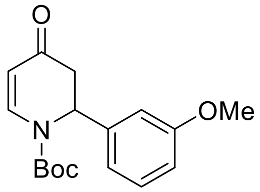

Using general procedure A, magnesium (0.15 g, 6.1 mmol), 1-bromo-3-methoxybenzene (0.77 mL, 6.1 mmol), *tert*-butyl 4-oxopyridine-1(4*H*)-carboxylate (1 g, 5.1 mmol) and Me_3SiCl (2.0 mL, 15 mmol) in THF (15 mL) gave the crude product. Purification by column chromatography on silica gel, eluting with CH_2Cl_2–MeOH (49:1) gave enone **1g** (1.3 g, 84%) as a clear oil; R*f* 0.69 [CH_2Cl_2–MeOH (19:1)]; FT-IR ν_{max} (film)/cm^{-1} 1753 (C=O), 1665 (C=O), 1602 (C=C); ^{1}H NMR ($CDCl_3$, 400 MHz) δ = 7.98 (d, 1H, *J* = 8.5 Hz), 7.32–7.18 (m, 1H), 6.84–6.78 (m, 2H), 6.78–6.74 (m, 1H), 5.64 (d, 1H, *J* = 7.5 Hz), 5.37 (d, 1H, *J* = 8.5 Hz), 3.78 (s, 3H), 3.15 (dd, 1H, *J* = 16.5, 7.5 Hz), 2.80 (d, 1H, *J* = 16.5 Hz), 1.49 (s, 9H); ^{13}C{1H} NMR ($CDCl_3$, 100 MHz) δ = 192.1, 159.9, 151.4, 143.0, 140.5, 129.9, 118.0, 112.9, 111.9, 107.0, 83.7, 55.6, 55.2, 41.9, 28.0; HRMS (ESI-TOF) *m/z*: $[M+Na]^+$ Calcd for $C_{17}H_{21}NO_4Na$ 326.1363; Found 326.1379; LRMS *m/z* (ES) 248 (100%), 304 (15%), 326 (50%, MNa^+).

tert-Butyl 2-(3,4-Dimethoxyphenyl)-4-oxo-3,4-dihydropyridine-1(2*H*)-carboxylate **1h**

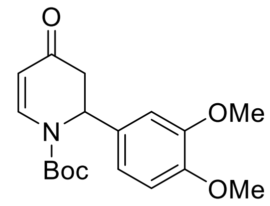

Using general procedure A, magnesium (0.15 g, 6.1 mmol), 4-bromo-1,2-dimethoxybenzene (0.88 mL, 6.1 mmol), *tert*-butyl 4-oxopyridine-1(4*H*)-carboxylate (1 g, 5.1 mmol) and Me_3SiCl (2.0 mL, 15 mmol) in THF (15 mL) gave the crude product. Purification by column chromatography on silica gel, eluting with CH_2Cl_2–MeOH (49:1) gave enone **1h** (0.68 g, 40%) as a clear oil; R_f 0.56 [CH_2Cl_2–MeOH (19:1)]; FT-IR ν_{max} (film)/cm^{-1} 1720 (C=O), 1665 (C=O), 1602 (C=C); ^{1}H NMR ($CDCl_3$, 400 MHz) δ = 7.92 (d, 1H, *J* = 7.5 Hz), 6.86–6.72 (m, 3H), 5.65 (d, 1H, *J* = 7.5 Hz), 5.37 (d, 1H, *J* = 7.5 Hz), 3.87 (s, 3H), 3.85 (s, 3H), 3.14 (dd, 1H, *J* = 16.5, 7.5 Hz), 2.81 (d, 1H, *J* = 16.5 Hz), 1.51 (s, 9H); ^{13}C{1H} NMR ($CDCl_3$, 100 MHz) δ = 192.5, 151.5, 149.1, 148.7, 142.7, 131.5, 118.3, 111.0, 109.5, 106.8, 83.7, 55.9, 55.8, 55.2, 41.7, 28.0; HRMS (ESI-TOF) *m/z*: $[M+Na]^+$ Calcd for $C_{18}H_{23}NO_5Na$ 356.1468; Found 356.1462; LRMS *m/z* (ES) 278 (100%), 334 (30%), 356 (40%, MNa^+).

2.3 Preparation of 2-aryl-4-piperidones 2a–h

tert-Butyl 4-Oxo-2-phenylpiperidine-1-carboxylate **2a**

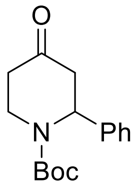

Using general procedure B, enone **1a** (2 g, 7.3 mmol) and LiBH(^{s}Bu)$_3$ (8 mL, 8 mmol, 1 M in THF) in THF (37 mL) gave the crude product. Purification by column chromatography on silica gel, eluting with petrol–EtOAc (9:1), gave piperidone **2a** (1.7 g, 85%) as a clear oil; R$_f$ 0.63 [petrol–EtOAc (1:1)]; ^{1}H NMR (CDCl$_3$, 400 MHz) δ = 7.41–7.18 (m, 5H), 5.74 (br s, 1H), 4.25 (br, 1H), 3.17 (br t, 1H, *J* = 11.0 Hz), 2.99 (dd, 1H, *J* = 15.5, 2.5 Hz), 2.87 (dd, 1H, *J* = 15.5, 7.0 Hz), 2.62–2.46 (m, 1H), 2.44–2.30 (m, 1H), 1.50 (s, 9H); ^{13}C{1H} NMR (CDCl$_3$, 100 MHz) δ = 207.9, 154.8, 140.5, 128.8, 127.6, 126.6, 80.8, 54.4, 44.4, 40.7, 38.7, 28.4. Data as reported.[14]

Resolution between the enantiomers of piperidone **2a** was achieved using a Beckman system fitted with a Phenomenex Cellulose-1 column (250 mm × 4.60 mm i.d.) as the stationary phase with a mixture of *n*-hexane:isopropanol (97:3 v/v) as the mobile phase at a flow rate of 1 mL·min^{-1}; ambient temperature, detection by UV absorbance at 210 nm. Injection volume was 10 μL of the sample prepared in a 2 g·L^{-1} solution of the eluent. Under these conditions, the components were eluted at 12.1 min and 14.1 min.

tert-Butyl 2-(4-Fluorophenyl)-4-oxopiperidine-1-carboxylate **2b**

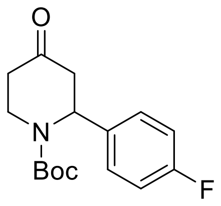

Using general procedure B, enone **1b** (1.3 g, 4.5 mmol) and LiBH(^{s}Bu)$_3$ (5 mL, 5 mmol, 1 M in THF) in THF (23 mL) gave the crude product. Purification by column chromatography on silica gel, eluting with petrol–EtOAc (4:1), gave piperidone **2b** (0.94 g, 71%) as a clear oil; R$_f$ 0.30 [petrol–EtOAc (7:3)]; FT-IR ν_{max} (film)/cm^{-1} 1717 (C=O), 1691 (C=O); ^{1}H NMR (CDCl$_3$, 400 MHz) δ = 7.25–7.17 (m, 2H), 7.07–6.94 (m, 2H), 5.73 (br, 1H), 4.24 (br, 1H), 3.14 (br t, 1H, *J* = 11.0 Hz), 2.99–2.79 (m, 2H), 2.63–2.47 (m, 1H), 2.47–2.30 (m, 1H), 1.50 (s, 9H); ^{13}C{1H} NMR (CDCl$_3$, 100 MHz) δ =207.7, 162.1 (d, *J* = 246.5 Hz), 154.7, 136.2, 128.3 (d, *J* = 8.0 Hz), 115.6 (d, *J* = 21.5 Hz), 80.9, 53.9, 44.5, 40.7, 38.7, 28.4; ^{19}F NMR (CDCl$_3$, 377 MHz) δ = −114.77; HRMS (ESI-TOF) *m/z*: [M+Na]$^+$ Calcd for $C_{16}H_{20}FNO_3Na$ 316.1319; Found 316.1325; LRMS *m/z* (ES) 142 (30%), 194 (25%), 238 (100%), 316 (45%, MNa$^+$).

tert-Butyl 2-(4-Chlorophenyl)-4-oxopiperidine-1-carboxylate **2c**

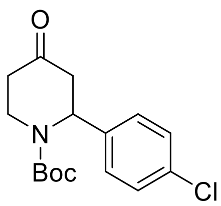

Using general procedure B, enone **1c** (0.85 g, 2.8 mmol) and LiBH(^{s}Bu)$_3$ (3.1 mL, 3.0 mmol, 1 M in THF) in THF (14 mL) gave the crude product. Purification by column chromatography on silica gel, eluting with petrol–EtOAc (4:1), gave piperidone **2c** (0.69 g, 81%) as a clear oil; R$_f$ 0.28 [petrol–EtOAc (7:3)]; FT-IR ν_{max} (film)/cm^{-1} 1727 (C=O), 1691 (C=O); ^{1}H NMR

($CDCl_3$, 400 MHz) δ = 7.38–7.25 (m, 2H), 7.25–7.11 (m, 2H), 5.70 (br, 1H), 4.38–4.11 (m, 1H), 3.16 (t, 1H, *J* = 11.5 Hz), 2.99–2.81 (m, 2H), 2.61–2.47 (m, 1H), 2.47–2.30 (m, 1H), 1.49 (s, 9H); $^{13}C\{^1H\}$ NMR ($CDCl_3$, 100 MHz) δ = 207.5, 154.7, 139.1, 133.5, 128.9, 128.0, 81.0, 54.0, 44.4, 40.6, 38.8, 28.4; HRMS (ESI-TOF) *m/z*: $[M+Na]^+$ Calcd for $C_{16}H_{20}{}^{35}ClNO_3Na$ 332.1024; Found 332.1033; LRMS *m/z* (ES) 332 (100%, MNa^+ for ^{35}Cl), 333 (15%), 334 (35%, MNa^+ for ^{37}Cl), 335 (5%).

tert-Butyl 2-(4-Methylphenyl)-4-oxopiperidine-1-carboxylate **2d**

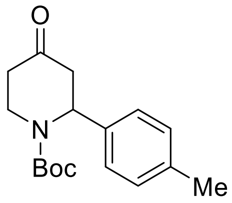

Using general procedure B, enone **1d** (1.3 g, 4.5 mmol) and $LiBH(^sBu)_3$ (5 mL, 5 mmol, 1 M in THF) in THF (23 mL) gave the crude product. Purification by column chromatography on silica gel, eluting with petrol–EtOAc (4:1), gave piperidone **2d** (1.1 g, 84%) as a clear oil; R_f 0.23 [petrol–EtOAc (4:1)]; FT-IR ν_{max} (film)/cm^{-1} 1720 (C=O), 1688 (C=O); ^1H NMR ($CDCl_3$, 400 MHz) δ = 7.21–7.08 (m, 4H), 5.74 (br, 1H), 4.23 (br, 1H), 3.20–3.06 (m, 1H), 2.97 (dd, 1H, *J* = 15.5, 2.0 Hz), 2.85 (dd, 1H, *J* = 15.5, 7.0 Hz), 2.61–2.46 (m, 1H), 2.43–2.27 (m, 4H), 1.51 (s, 9H); $^{13}C\{^1H\}$ NMR ($CDCl_3$, 100 MHz, one quaternary carbon not observed) δ = 208.1, 154.8, 137.3, 129.4, 126.6, 80.7, 54.1, 44.4, 40.7, 38.6, 28.4, 21.0; HRMS (ESI-TOF) *m/z*: $[M+Na]^+$ Calcd for $C_{17}H_{23}NO_3Na$ 312.1570; Found 312.1581; LRMS *m/z* (ES) 142 (65%), 190 (15%), 234 (75%), 312 (100%, MNa^+).

tert-Butyl 2-(4-Methoxyphenyl)-4-oxopiperidine-1-carboxylate **2e**

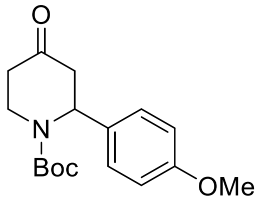

Using general procedure B, enone **1e** (1.5 g, 4.9 mmol) and LiBH(^{s}Bu)$_3$ (5.4 mL, 5.4 mmol, 1 M in THF) in THF (25 mL) gave the crude product. Purification by column chromatography on silica gel, eluting with petrol–EtOAc (17:3), gave piperidone **2e** (1.2 g, 80%) as a clear oil; R$_f$ 0.30 [petrol–EtOAc (7:3)]; FT-IR ν_{max} (film)/cm^{-1} 1718 (C=O), 1687 (C=O); ^{1}H NMR ($CDCl_3$, 400 MHz) δ = 7.18 (d, 2H, *J* = 8.5 Hz), 6.87 (d, 2H, *J* = 8.5 Hz), 5.74 (br, 1H), 4.21 (br, 1H), 3.80 (s, 3H), 3.18–3.03 (m, 1H), 2.95 (dd, 1H, *J* = 15.5, 1.5 Hz), 2.84 (dd, 1H, *J* = 15.5, 6.5 Hz), 2.63–2.45 (m, 1H), 2.42–2.29 (m, 1H), 1.51 (s, 9H); ^{13}C{1H} NMR ($CDCl_3$, 100 MHz) δ = 208.1, 158.9, 154.8, 132.3, 127.9, 114.1, 80.7, 55.3, 53.8, 44.4, 40.8, 38.5, 28.4; HRMS (ESI-TOF) *m/z*: [M+Na]$^+$ Calcd for $C_{17}H_{23}NO_4Na$ 328.1519; Found 328.1534; LRMS *m/z* (ES) 328 (100%, MNa$^+$).

tert-Butyl 2-(3-Trifluoromethylphenyl)-4-oxopiperidine-1-carboxylate **2f**

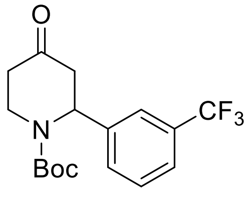

Using general procedure B, enone **1f** (1.7 g, 5.0 mmol) and LiBH(^{s}Bu)$_3$ (5.5 mL, 5.5 mmol, 1 M in THF) in THF (25 mL) gave the crude product. Purification by column chromatography on silica gel, eluting with petrol–EtOAc (3:1), gave piperidone **2f** (1.2 g, 70%) as a clear oil; R$_f$ 0.29 [petrol–EtOAc (1:1)]; FT-IR ν_{max} (film)/cm^{-1} 1686 (C=O); ^{1}H NMR ($CDCl_3$, 400 MHz)

δ = 7.61–7.42 (m, 4H), 5.71 (br, 1H), 4.29 (br d, 1H, J = 12.0 Hz), 3.33–3.18 (m, 1H), 3.01–2.86 (m, 2H), 2.66–2.52 (m, 1H), 2.42 (dt, 1H, J = 16.5, 3.5 Hz), 1.47 (s, 9H); ^{13}C{1H} NMR ($CDCl_3$, 100 MHz) δ = 207.2, 154.7, 131.7–130.9 (m), 129.6, 129.3, 125.3, 124.8–124.2 (m), 123.7–123.0 (m), 122.6, 81.2, 54.4, 44.4, 40.5, 39.0, 28.3; ^{19}F NMR ($CDCl_3$, 377 MHz) δ = −62.72; HRMS (ESI-TOF) *m/z*: $[M+Na]^+$ Calcd for $C_{17}H_{20}F_3NO_3Na$ 366.1287; Found 366.1302; LRMS *m/z* (ES) 244 (20%), 288 (100%), 366 (30%, MNa^+).

tert-Butyl 2-(3-Methoxyphenyl)-4-oxopiperidine-1-carboxylate **2g**

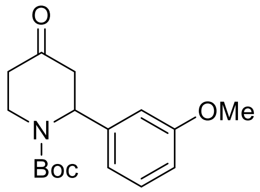

Using general procedure B, enone **1g** (1.3 g, 4.3 mmol) and LiBH(^{s}Bu)$_3$ (4.7 mL, 4.7 mmol, 1 M in THF) in THF (22 mL) gave the crude product. Purification by column chromatography on silica gel, eluting with petrol–EtOAc (3:1), gave piperidone **2g** (1 g, 76%) as a clear oil; R_f 0.26 [petrol–EtOAc (7:3)]; FT-IR ν_{max} (film)/cm^{-1} 1723 (C=O), 1686 (C=O); ^{1}H NMR ($CDCl_3$, 400 MHz) δ = 7.32–7.19 (m, 1H), 6.90–6.77 (m, 3H), 5.71 (br, 1H), 4.23 (br, 1H), 3.80 (s, 3H), 3.26–3.08 (m, 1H), 2.97 (dd, 1H, J = 15.5, 3.5 Hz), 2.85 (dd, 1H, J = 15.5, 7.0 Hz), 2.60–2.46 (m, 1H), 2.42–2.33 (m, 1H), 1.50 (s, 9H); ^{13}C{1H} NMR ($CDCl_3$, 100 MHz) δ = 207.8, 160.0, 154.8, 142.1, 129.8, 118.8, 112.9, 112.4, 80.7, 55.2, 54.3, 44.4, 40.6, 38.8, 28.4; HRMS (ESI-TOF) *m/z*: $[M+Na]^+$ Calcd for $C_{17}H_{23}NO_4Na$ 328.1519; Found 328.1530; LRMS *m/z* (ES) 188 (75%), 232 (100%), 328 (90%, MNa^+).

tert-Butyl 2-(3,4-Dimethoxyphenyl)-4-oxopiperidine-1-carboxylate **2h**

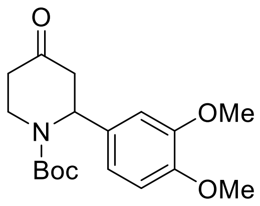

Using general procedure B, enone **1h** (0.67 g, 2.0 mmol) and LiBH(^{s}Bu)$_3$ (2.2 mL, 2.2 mmol, 1 M in THF) in THF (20 mL) gave the crude product. Purification by column chromatography on silica gel, eluting with petrol−EtOAc (1:1), gave piperidone **2h** (0.39 g, 58%) as a clear oil; R$_f$ 0.14 [petrol−EtOAc (7:3)]; FT-IR ν_{max} (film)/cm^{-1} 1713 (C=O), 1687 (C=O); ^{1}H NMR ($CDCl_3$, 400 MHz) δ = 6.92–6.68 (m, 3H), 5.77 (br, 1H), 4.21 (br, 1H), 3.88 (s, 3H), 3.87 (s, 3H), 3.07 (t, 1H, *J* = 11.5 Hz), 2.97 (d, 1H, *J* = 15.5 Hz), 2.85 (dd, 1H, *J* = 15.5, 7.0 Hz), 2.61–2.47 (m, 1H), 2.36 (d, 1H, *J* = 15.5 Hz), 1.53 (s, 9H); ^{13}C{1H} NMR ($CDCl_3$, 100 MHz) δ = 208.1, 154.8, 149.2, 148.5, 132.8, 118.9, 110.9, 110.0, 80.7, 55.9, 55.8, 54.0, 44.2, 40.8, 38.6, 28.4; HRMS (ESI-TOF) *m/z*: [M+Na]$^+$ Calcd for $C_{18}H_{25}NO_5Na$ 358.1625; Found 358.1635; LRMS *m/z* (ES) 98 (20%), 236 (15%), 358 (100%, MNa$^+$).

2.4 Preparation of 2-aryl-4-methylenepiperidines 3a–h

tert-Butyl 4-Methylidene-2-phenylpiperidine-1-carboxylate **3a**

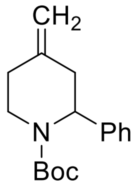

Using general procedure C, $MePPh_3Br$ (26 g, 73 mmol), KO^tBu (8 g, 73 mmol) and piperidone **2a** (5 g, 18 mmol) in THF (130 mL) gave the crude product. Purification by column chromatography on silica gel, eluting with petrol–EtOAc (9:1), gave alkene **3a** (3.5 g, 71%) as a clear oil; R_f 0.52 [petrol–EtOAc (4:1)]; ^1H NMR ($CDCl_3$, 400 MHz) δ = 7.38–7.20 (m, 5H), 5.51 (br, 1H), 4.98–4.77 (m, 2H), 4.17–3.99 (m, 1H), 2.95–2.74 (m, 2H), 2.72–2.61 (m, 1H), 2.42–2.13 (m, 2H), 1.50 (s, 9H); $^{13}C\{1H\}$ NMR ($CDCl_3$, 100 MHz) δ = 155.3, 142.4, 140.8, 128.3, 127.2, 126.7, 111.4, 79.9, 53.9, 40.1, 36.6, 33.8, 28.5. Data as reported.[15]

Resolution between the enantiomers of alkene **3a** was achieved using a Beckman system fitted with a Phenomenex Cellulose-2 column (250 mm × 4.60 mm i.d.) as the stationary phase with a mixture of *n*-hexane:isopropanol (99:1 v/v) as the mobile phase at a flow rate of 1 $mL\cdot min^{-1}$; ambient temperature, detection by UV absorbance at 210 nm. Injection volume was 10 μL of the sample prepared in a 2 $g\cdot L^{-1}$ solution of the eluent. Under these conditions, the components were eluted at 5.6 min and 12.3 min.

tert-Butyl 2-(4-Fluorophenyl)-4-methylidenepiperidine-1-carboxylate **3b**

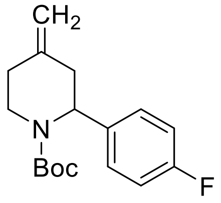

Using general procedure C, $MePPh_3Br$ (4.3 g, 12 mmol), KO^tBu (1.3 g, 12 mmol) and piperidone **2b** (0.92 g, 3.1 mmol) in THF (22 mL) gave the crude product. Purification by column chromatography on silica gel, eluting with petrol−EtOAc (9:1), gave alkene **3b** (0.73 g, 81 %) as a clear oil; R_f 0.58 [petrol−EtOAc (4:1)]; FT-IR ν_{max} (film)/cm^{-1} 1687 (C=O), 1652 (C=C); ^1H NMR ($CDCl_3$, 400 MHz) δ = 7.35–7.26 (m, 2H), 7.05–6.95 (m, 2H), 5.48 (br d, 1H, *J* = 6.0 Hz), 4.88 (br s, 2H), 4.07 (dd, 1H, *J* = 13.0, 4.0 Hz), 2.87–2.71 (m, 2H), 2.67 (dd, 1H, *J* = 14.5, 6.0 Hz), 2.38–2.18 (m, 2H), 1.50 (s, 9H); $^{13}C\{1H\}$ NMR ($CDCl_3$, 100 MHz) δ = 161.7 (d, *J* = 245.0 Hz), 155.1, 142.2, 136.6, 128.8 (d, *J* = 8.0 Hz), 115.1 (d, *J* = 21.0 Hz), 111.6, 80.0, 53.2, 39.9, 36.7, 33.7, 28.5; ^{19}F NMR ($CDCl_3$, 377 MHz) δ = −116.28; HRMS (ESI-TOF) *m/z*: $[M+Na]^+$ Calcd for $C_{17}H_{22}FNO_2Na$ 314.1527; Found 314.1535; LRMS *m/z* (ES) 140 (15%), 236 (100%), 314 (25%, MNa^+).

Resolution between the enantiomers of alkene **3b** was achieved using a Beckman system fitted with a Phenomenex Cellulose-2 column (250 mm × 4.60 mm i.d.) as the stationary phase with a mixture of *n*-hexane:isopropanol (99:1 v/v) as the mobile phase at a flow rate of 1 $mL{\cdot}min^{-1}$; ambient temperature, detection by UV absorbance at 210 nm. Injection volume was 10 μL of the sample prepared in a 2 $g{\cdot}L^{-1}$ solution of the eluent. Under these conditions, the components were eluted at 5.2 min and 11.2 min.

Alternatively, resolution between the enantiomers of alkene **3b** was achieved using a Agilent system fitted with a Chiralcel OX-H column (250 mm × 4.60 mm i.d.) as the stationary phase with a mixture of *n*-hexane:isopropanol (99:1 v/v) as the mobile phase at a flow rate of

1 mL·min^{-1}; ambient temperature, detection by UV absorbance at 210 nm. Injection volume was 5 μL of the sample prepared in a 2 g·L^{-1} solution of the eluent. Under these conditions, the components were eluted at 5.4 min and 6.0 min.

tert-Butyl 2-(4-Chlorophenyl)-4-methylidenepiperidine-1-carboxylate **3c**

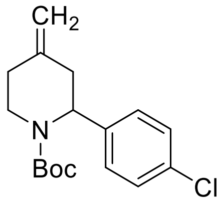

Using general procedure C, $MePPh_3Br$ (3.1 g, 8.7 mmol), KO^{t}Bu (1.0 g, 8.7 mmol) and piperidone **2c** (0.68 g, 2.2 mmol) in THF (16 mL) gave the crude product. Purification by column chromatography on silica gel, eluting with petrol–EtOAc (9:1), gave alkene **3c** (0.58 g, 86%) as a clear oil; R_f 0.48 [petrol–EtOAc (4:1)]; FT-IR ν_{max} (film)/cm^{-1} 1685 (C=O); ^{1}H NMR ($CDCl_3$, 400 MHz) δ = 7.40–7.22 (m, 4H), 5.46 (br d, 1H, *J* = 4.5 Hz), 4.87 (br s, 2H), 4.08 (dd, 1H, *J* = 13.0, 4.0 Hz), 2.86–2.72 (m, 2H), 2.72–2.60 (m, 1H), 2.39–2.15 (m, 2H), 1.50 (s, 9H); ^{13}C{1H} NMR ($CDCl_3$, 100 MHz) δ = 155.1, 142.0, 139.4, 132.5, 128.6, 128.4, 111.6, 80.1, 53.4, 40.1, 36.6, 33.7, 28.4; HRMS (ESI-TOF) *m/z*: [M+Na]$^+$ Calcd for $C_{17}H_{22}{}^{35}ClNO_2Na$ 330.1123; Found 330.1238; LRMS *m/z* (ES) 330 (100%, MNa$^+$ for ^{35}Cl), 331 (15%), 332 (35%, MNa$^+$ for ^{37}Cl), 333 (10%).

Resolution between the enantiomers of alkene **3c** was achieved using a Beckman system fitted with a Phenomenex Cellulose-2 column (250 mm × 4.60 mm i.d.) as the stationary phase with a mixture of *n*-hexane:isopropanol (99:1 v/v) as the mobile phase at a flow rate of 1 mL·min^{-1}; ambient temperature, detection by UV absorbance at 210 nm. Injection volume was 10 μL of the sample prepared in a 2 g·L^{-1} solution of the eluent. Under these conditions, the components were eluted at 4.9 min and 6.4 min.

tert-Butyl 2-(4-Methylphenyl)-4-methylidenepiperidine-1-carboxylate **3d**

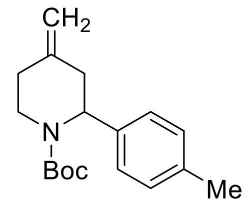

Using general procedure C, $MePPh_3Br$ (5.4 g, 15 mmol), KO^tBu (1.7 g, 15 mmol) and piperidone **2d** (1.1 g, 3.7 mmol) in THF (27 mL) gave the crude product. Purification by column chromatography on silica gel, eluting with petrol–EtOAc (9:1), gave alkene **3d** (0.81 g, 76%) as a clear oil; R_f 0.54 [petrol–EtOAc (4:1)]; FT-IR ν_{max} (film)/cm^{-1} 1689 (C=O), 1652 (C=C); ^{1}H NMR ($CDCl_3$, 400 MHz) δ = 7.23 (d, 2H, *J* = 8.0 Hz), 7.14 (d, 2H, *J* = 8.0 Hz), 5.49 (br, 1H), 4.94–4.78 (m, 2H), 4.08 (d, 1H, *J* = 9.0 Hz), 2.92–2.73 (m, 2H), 2.65 (dd, 1H, *J* = 14.0, 5.5 Hz), 2.40–2.13 (m, 5H), 1.51 (s, 9H); ^{13}C{1H} NMR ($CDCl_3$, 100 MHz) δ = 155.3, 142.5, 137.6, 136.3, 129.0, 127.1, 111.3, 79.8, 53.6, 40.0, 36.6, 33.9, 28.5, 21.0; HRMS (ESI-TOF) *m/z*: $[M+Na]^+$ Calcd for $C_{18}H_{25}NO_2Na$ 310.1778; Found 310.1792; LRMS *m/z* (ES) 140 (40%), 232 (100%), 310 (50%, MNa^+).

Resolution between the enantiomers of alkene **3d** was achieved using a Beckman system fitted with a Phenomenex Cellulose-2 column (250 mm × 4.60 mm i.d.) as the stationary phase with a mixture of *n*-hexane:isopropanol (99:1 v/v) as the mobile phase at a flow rate of 1 mL·min^{-1}; ambient temperature, detection by UV absorbance at 210 nm. Injection volume was 10 µL of the sample prepared in a 2 g·L^{-1} solution of the eluent. Under these conditions, the components were eluted at 5.3 min and 9.3 min.

tert-Butyl 2-(4-Methoxyphenyl)-4-methylidenepiperidine-1-carboxylate **3e**

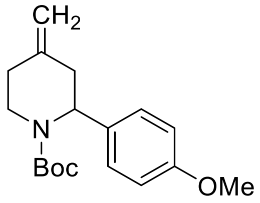

Using general procedure C, $MePPh_3Br$ (5.7 g, 16 mmol), KO^tBu (1.8 g, 16 mmol) and piperidone **2e** (1.2 g, 3.9 mmol) in THF (28 mL) gave the crude product. Purification by column chromatography on silica gel, eluting with petrol–EtOAc (9:1), gave alkene **3e** (0.89 g, 73%) as a clear oil; R_f 0.57 [petrol–EtOAc (4:1)]; FT-IR ν_{max} (film)/cm^{-1} 1685 (C=O), 1654 (C=C); ^{1}H NMR ($CDCl_3$, 400 MHz) δ = 7.27 (d, 2H, *J* = 9.0 Hz), 6.86 (d, 2H, *J* = 9.0 Hz), 5.47 (br d, 1H, *J* = 5.5 Hz), 4.93-4.81 (m, 2H), 4.06 (dd, 1H, *J* = 13.0, 4.5 Hz), 3.81 (s, 3H), 2.89–2.72 (m, 2H), 2.70–2.60 (m, 1H), 2.37–2.18 (m, 2H), 1.51 (s, 9H); ^{13}C{1H} NMR ($CDCl_3$, 100 MHz) δ =158.3, 155.2, 142.6, 132.9, 128.4, 113.6, 111.3, 79.8, 55.2, 53.2, 39.8, 36.6, 33.9, 28.5; HRMS (ESI-TOF) *m/z*: [M+Na]$^+$ Calcd for $C_{18}H_{25}NO_3Na$ 326.1727; Found 326.1718; LRMS *m/z* (ES) 140 (100%), 248 (100%), 326 (90%, MNa$^+$).

Resolution between the enantiomers of alkene **3e** was achieved using a Beckman system fitted with a Daicel Chiralpak-IA column (250 mm × 4.60 mm i.d.) as the stationary phase with a mixture of *n*-hexane:isopropanol (99:1 v/v) as the mobile phase at a flow rate of 1 mL·min^{-1}; ambient temperature, detection by UV absorbance at 210 nm. Injection volume was 10 µL of the sample prepared in a 2 g·L^{-1} solution of the eluent. Under these conditions, the components were eluted at 10.7 min and 13.1 min.

tert-Butyl 2-(3-Trifluoromethylphenyl)-4-methylidenepiperidine-1-carboxylate **3f**

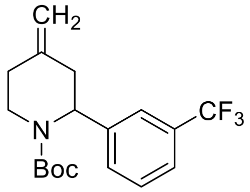

Using general procedure C, $MePPh_3Br$ (5.0 g, 14 mmol), KO^tBu (1.6 g, 14 mmol) and piperidone **2f** (1.2 g, 3.5 mmol) in THF (25 mL) gave the crude product. Purification by column chromatography on silica gel, eluting with petrol−EtOAc (9:1), gave alkene **3f** (0.91 g, 76%) as a clear oil; R_f 0.48 [petrol−EtOAc (4:1)]; FT-IR ν_{max} (film)/cm^{-1} 1695 (C=O); ^{1}H NMR ($CDCl_3$, 400 MHz) δ = 7.63–7.40 (m, 4H), 5.51 (br, 1H), 4.89 (br s, 2H), 4.10 (d, 1H, *J* = 13.5 Hz), 2.93–2.63 (m, 3H), 2.40–2.20 (m, 2H), 1.50 (s, 9H); ^{13}C{1H} NMR ($CDCl_3$, 100 MHz) δ = 155.2, 142.0, 141.8, 130.5, 128.8, 126.9 (q, *J* = 267.0 Hz), 124.23–123.85 (m), 123.85–123.44 (m), 111.8, 80.3, 53.7, 40.2, 36.6, 33.6, 28.4; ^{19}F NMR ($CDCl_3$, 377 MHz) δ = −62.67; HRMS (ESI-TOF) *m/z*: $[M+Na]^+$ Calcd for $C_{18}H_{22}F_3NO_2Na$ 364.1495; Found 364.1512; LRMS *m/z* (ES) 242 (10%), 286 (100%), 364 (15%, MNa^+).

Resolution between the enantiomers of alkene **3f** was achieved using a Beckman system fitted with a Phenomenex Amylose-2 column (250 mm × 4.60 mm i.d.) as the stationary phase with a mixture of *n*-hexane:isopropanol (99:1 v/v) as the mobile phase at a flow rate of 1 mL·min^{-1}; ambient temperature, detection by UV absorbance at 210 nm. Injection volume was 10 μL of the sample prepared in a 2 g·L^{-1} solution of the eluent. Under these conditions, the components were eluted at 6.0 min and 6.5 min.

tert-Butyl 2-(3-Methoxyphenyl)-4-methylidenepiperidine-1-carboxylate **3g**

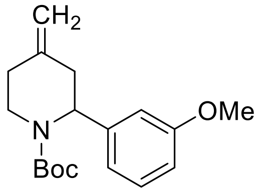

Using general procedure C, $MePPh_3Br$ (4.6 g, 13 mmol), KO^tBu (1.5 g, 13 mmol) and piperidone **2g** (0.95 g, 3.1 mmol) in THF (22 mL) gave the crude product. Purification by column chromatography on silica gel, eluting with petrol–EtOAc (9:1), gave alkene **3g** (0.79 g, 84%) as a clear oil; R_f 0.50 [petrol–EtOAc (4:1)]; FT-IR ν_{max} (film)/cm^{-1} 1689 (C=O); ^{1}H NMR ($CDCl_3$, 400 MHz) δ = 7.32–7.20 (m, 1H), 6.97–6.88 (m, 2H), 6.82–6.76 (m, 1H), 5.48 (br, 1H), 4.94–4.82 (m, 2H), 4.09 (br d, 1H, *J* = 10.0 Hz), 3.80 (s, 3H), 2.94–2.75 (m, 2H), 2.66 (dd, 1H, *J* = 14.0, 6.0 Hz), 2.40–2.17 (m, 2H), 1.51 (s, 9H); ^{13}C{1H} NMR ($CDCl_3$, 100 MHz) δ = 159.6, 155.2, 142.54, 142.47, 129.3, 119.6, 113.2, 111.9, 111.4, 79.9, 55.1, 53.8, 40.1, 36.6, 33.8, 28.5; HRMS (ESI-TOF) *m/z*: $[M+Na]^+$ Calcd for $C_{18}H_{25}NO_3Na$ 326.1727; Found 326.1720; LRMS *m/z* (ES) 161 (35%), 204 (15%), 248 (100%), 326 (45%, MNa^+).

Resolution between the enantiomers of alkene **3g** was achieved using a Beckman system fitted with a Phenomenex Cellulose-2 column (250 mm × 4.60 mm i.d.) as the stationary phase with a mixture of *n*-hexane:isopropanol (99:1 v/v) as the mobile phase at a flow rate of 1 mL·min^{-1}; ambient temperature, detection by UV absorbance at 210 nm. Injection volume was 10 µL of the sample prepared in a 2 g·L^{-1} solution of the eluent. Under these conditions, the components were eluted at 9.6 min and 18.6 min.

tert-Butyl 2-(3,4-Dimethoxyphenyl)-4-methylidenepiperidine-1-carboxylate **3h**

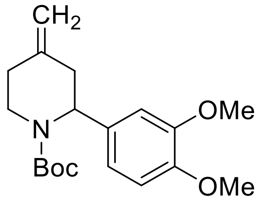

Using general procedure C, $MePPh_3Br$ (1.7 g, 4.6 mmol), KO^tBu (0.52 g, 4.6 mmol) and piperidone **2h** (0.39 g, 1.2 mmol) in THF (8.4 mL) gave the crude product. Purification by column chromatography on silica gel, eluting with petrol–EtOAc (7:3), gave alkene **3h** (0.33 g, 85%) as a clear oil; R_f 0.29 [petrol–EtOAc (4:1)]; FT-IR ν_{max} (film)/cm^{-1} 1690 (C=O), 1650 (C=C); ^1H NMR ($CDCl_3$, 400 MHz) δ = 6.98–6.89 (m, 2H), 6.82 (d, 1H, *J* = 8.0 Hz), 5.48 (d, 1H, *J* = 5.0 Hz), 4.90 (br s, 2H), 4.06 (d, 1H, *J* = 10.0 Hz), 3.88 (s, 3H), 3.86 (s, 3H), 2.92–2.59 (m, 3H), 2.39–2.16 (m, 2H), 1.52 (s, 9H); $^{13}C\{1H\}$ NMR ($CDCl_3$, 100 MHz) δ = 155.2, 148.8, 147.8, 143.0, 133.5, 119.6, 111.2, 110.8, 110.7, 79.8, 55.84, 55.78, 53.2, 39.9, 36.6, 33.9, 28.5; HRMS (ESI-TOF) *m/z*: $[M+Na]^+$ Calcd for $C_{19}H_{27}NO_4Na$ 356.1832; Found 356.1841; LRMS *m/z* (ES) 96 (5%), 140 (35%), 191 (20%), 278 (25%), 356 (100%, MNa^+).

Resolution between the enantiomers of alkene **3h** was achieved using a Beckman system fitted with a Phenomenex Cellulose-1 column (250 mm × 4.60 mm i.d.) as the stationary phase with a mixture of *n*-hexane:isopropanol (97:3 v/v) as the mobile phase at a flow rate of 1 mL·min^{-1}; ambient temperature, detection by UV absorbance at 210 nm. Injection volume was 10 μL of the sample prepared in a 2 g·L^{-1} solution of the eluent. Under these conditions, the components were eluted at 8.1 min and 9.3 min.

2.5 Preparation of racemic 2,2-disubstituted products

1-*tert*-Butyl 2-Methyl 4-Methylidene-2-phenylpiperidine-1,2-dicarboxylate **4a**

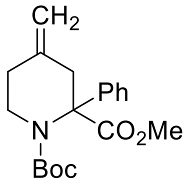

Using general procedure D, *n*-BuLi (0.18 mL, 0.44 mmol, 2.4 M in hexanes) and alkene **3a** (100 mg, 0.366 mmol) in THF (1.5 mL) at –40 °C and MeOCOCl (0.1 mL, 1.28 mmol) gave the crude product. Purification by column chromatography on silica gel, eluting with pentane–EtOAc (9:1), gave carbamate **4a** (106 mg, 87%) as a clear oil; R_f 0.41 [petrol–EtOAc (4:1)]; FT-IR ν_{max} (film)/cm^{-1} 1744 (C=O), 1696 (C=O), 1654 (C=C); ^{1}H NMR ($CDCl_3$, 400 MHz) δ = 7.46 (d, 2H, *J* = 7.5 Hz), 7.34 (t, 2H, *J* = 7.5 Hz), 7.30–7.23 (m, 1H), 4.82–4.70 (m, 2H), 4.01–3.87 (m, 1H), 3.72 (s, 3H), 3.54 (br, 1H), 3.13–2.88 (m, 2H), 2.57–2.21 (m, 2H), 1.34 (br s, 9H); ^{13}C{1H} NMR ($CDCl_3$, 100 MHz, two quaternary carbon signals and one CH_2 signal not observed) δ = 172.7, 140.0, 127.7, 127.3, 127.0, 112.1, 80.8, 52.2, 41.8, 31.0, 28.1; HRMS (ESI-TOF) *m/z*: [M+Na]$^+$ Calcd for $C_{19}H_{25}NO_4Na$ 354.1676; Found 354.1691; LRMS *m/z* (ES) 232 (100%), 354 (55%, MNa^+).

Resolution between the enantiomers of carbamate **4a** was achieved using a Beckman system fitted with a Phenomenex Cellulose-2 column (250 mm × 4.60 mm i.d.) as the stationary phase with a mixture of *n*-hexane:isopropanol (99:1 v/v) as the mobile phase at a flow rate of 1 mL·min$^{–1}$; ambient temperature, detection by UV absorbance at 210 nm. Injection volume was 10 µL of the sample prepared in a 2 g·L$^{–1}$ solution of the eluent. Under these conditions, the components were eluted at 10.9 min and 12.6 min.

1-*tert*-Butyl 2-Methyl 2-(4-Fluorophenyl)-4-methylidenepiperidine-1,2-dicarboxylate **4b**

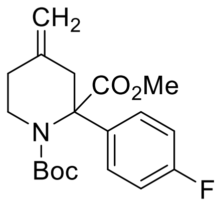

Using general procedure D, *n*-BuLi (0.18 mL, 0.44 mmol, 2.4 M in hexanes) and alkene **3b** (108 mg, 0.366 mmol) in THF (1.5 mL) at –40 °C and MeOCOCl (0.1 mL, 1.28 mmol) gave the crude product. Purification by column chromatography on silica gel, eluting with pentane–EtOAc (93:7) gave carbamate **4b** (116 mg, 90%) as a clear oil; R_f 0.23 [petrol–EtOAc (4:1)]; FT-IR ν_{max} (film)/cm^{-1} 1744 (C=O), 1696 (C=O); ^{1}H NMR ($CDCl_3$, 400 MHz) δ = 7.50–7.37 (m, 2H), 7.07–6.95 (m, 2H), 4.75 (br s, 2H), 3.97–3.86 (m, 1H), 3.73 (s, 3H), 3.54 (br, 1H), 3.04 (d, 1H, *J* = 14.5 Hz), 2.98–2.79 (m, 1H), 2.59–2.21 (m, 2H), 1.34 (br s, 9H); ^{13}C{1H} NMR ($CDCl_3$, 100 MHz, three quaternary carbon signals & three CH_2 signals not observed) δ = 172.7, 161.8 (d, *J* = 246.0 Hz), 139.6, 129.0, 114.5 (d, *J* = 21.0 Hz), 112.3, 81.0, 52.3, 28.1; ^{19}F NMR ($CDCl_3$, 377 MHz) δ = −116.13; HRMS (ESI-TOF) *m/z*: $[M+Na]^+$ Calcd for $C_{19}H_{24}FNO_4Na$ 372.1582; Found 372.1596; LRMS *m/z* (ES) 187 (10%), 250 (100%), 372 (90%, MNa^+).

Resolution between the enantiomers of carbamate **4b** was achieved using a Beckman system fitted with a Phenomenex Cellulose-2 column (250 mm × 4.60 mm i.d.) as the stationary phase with a mixture of *n*-hexane:isopropanol (99:1 v/v) as the mobile phase at a flow rate of 1 mL·min^{-1}; ambient temperature, detection by UV absorbance at 210 nm. Injection volume was 10 µL of the sample prepared in a 2 g·L^{-1} solution of the eluent. Under these conditions, the components were eluted at 11.4 min and 12.6 min.

Alternatively, resolution between the enantiomers of carbamate **4b** was achieved using a Agilent system fitted with a Chiral Art Cellulose-C column (250 mm × 4.60 mm i.d.) as the

stationary phase with a mixture of *n*-hexane:isopropanol (99:1 v/v) as the mobile phase at a flow rate of 1 mL·min^{-1}; ambient temperature, detection by UV absorbance at 210 nm. Injection volume was 5 μL of the sample prepared in a 2 g·L^{-1} solution of the eluent. Under these conditions, the components were eluted at 7.7 min and 8.4 min.

1-*tert*-Butyl 2-Methyl 2-(4-Chlorophenyl)-4-methylidenepiperidine-1,2-dicarboxylate **4c**

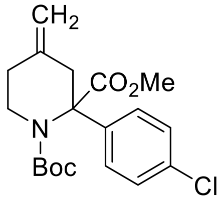

Using general procedure D, *n*-BuLi (0.20 mL, 0.44 mmol, 2.2 M in hexanes) and alkene **3c** (114 mg, 0.366 mmol) in THF (1.5 mL) at –40 °C and MeOCOCl (0.1 mL, 1.28 mmol) gave the crude product. Purification by column chromatography on silica gel, eluting with pentane–EtOAc (93:7), gave carbamate **4c** (106 mg, 78%) as a clear oil; R_f 0.32 [petrol–EtOAc (4:1)]; FT-IR ν_{max} (film)/cm^{-1} 1744 (C=O), 1698 (C=O); ^{1}H NMR ($CDCl_3$, 400 MHz) δ = 7.45–7.37 (m, 2H), 7.34–7.27 (m, 2H), 4.75 (br s, 2H), 3.95–3.84 (m, 1H), 3.73 (s, 3H), 3.54 (br, 1H), 3.04 (d, 1H, *J* = 14.5 Hz), 2.96–2.79 (m, 1H), 2.59–2.18 (m, 2H), 1.34 (br s, 9H); ^{13}C{1H} NMR ($CDCl_3$, 100 MHz, two quaternary carbon signals & three CH_2 signals not observed) δ = 172.5, 139.3, 132.9, 128.8, 127.8, 112.4, 81.1, 67.6, 52.4, 28.1; HRMS (ESI-TOF) *m/z*: [M+Na]$^+$ Calcd for $C_{19}H_{24}{}^{35}ClNO_4Na$ 388.1287; Found 388.1300; LRMS *m/z* (ES) 388 (100%, MNa$^+$ for ^{35}Cl), 389 (20%), 390 (35%, MNa$^+$ for ^{37}Cl), 391 (5%).

Resolution between the enantiomers of carbamate **4c** was achieved using a Beckman system fitted with a Phenomenex Cellulose-2 column (250 mm × 4.60 mm i.d.) as the stationary phase with a mixture of *n*-hexane:isopropanol (99:1 v/v) as the mobile phase at a flow rate of 1 mL·min^{-1}; ambient temperature, detection by UV absorbance at 210 nm. Injection volume

was 10 μL of the sample prepared in a 2 g·L^{-1} solution of the eluent. Under these conditions, the components were eluted at 8.5 min and 10.0 min.

1-*tert*-Butyl 2-Methyl 2-(4-Methylphenyl)-4-methylidenepiperidine-1,2-dicarboxylate **4d**

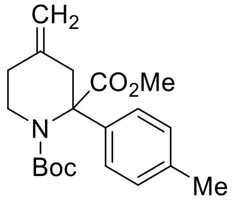

Using general procedure D, *n*-BuLi (0.18 mL, 0.44 mmol, 2.4 M in hexanes) and alkene **3d** (106 mg, 0.366 mmol) in THF (1.5 mL) at –40 °C and MeOCOCl (0.1 mL, 1.28 mmol) gave the crude product. Purification by column chromatography on silica gel, eluting with pentane–EtOAc (93:7), gave carbamate **4d** (117 mg, 92%) as a clear oil; R_f 0.47 [petrol–EtOAc (4:1)]; FT-IR ν_{max} (film)/cm^{-1} 1741 (C=O), 1693 (C=O), 1655 (C=C); ^{1}H NMR ($CDCl_3$, 400 MHz) δ = 7.32 (d, 2H, J = 8.0 Hz), 7.14 (d, 2H, J = 8.0 Hz), 4.84–4.67 (m, 2H), 3.99–3.88 (m, 1H), 3.71 (s, 3H), 3.44 (br, 1H), 3.07–2.91 (m, 2H), 2.52–2.18 (m, 5H), 1.37 (br s, 9H); ^{13}C{1H} NMR ($CDCl_3$, 100 MHz, one quaternary carbon signal & one CH_2 signal not observed) δ = 172.8, 155.8, 140.2, 136.7, 128.5, 127.3, 112.0, 80.8, 67.7, 52.2, 42.0, 31.1, 28.1, 21.0; HRMS (ESI-TOF) *m/z*: [M+Na]$^+$ Calcd for $C_{20}H_{27}NO_4Na$ 368.1832; Found 368.1839; LRMS *m/z* (ES) 185 (10%), 246 (100%), 368 (90%, MNa$^+$).

Resolution between the enantiomers of carbamate **4d** was achieved using a Beckman system fitted with a Phenomenex Cellulose-2 column (250 mm × 4.60 mm i.d.) as the stationary phase with a mixture of *n*-hexane:isopropanol (99:1 v/v) as the mobile phase at a flow rate of 1 mL·min^{-1}; ambient temperature, detection by UV absorbance at 210 nm. Injection volume was 10 μL of the sample prepared in a 2 g·L^{-1} solution of the eluent. Under these conditions, the components were eluted at 10.3 min and 12.2 min.

1-*tert*-Butyl 2-Methyl 2-(4-Methoxyphenyl)-4-methylidenepiperidine-1,2-dicarboxylate **4e**

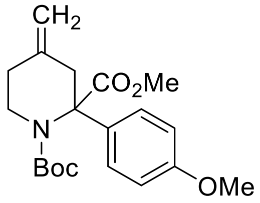

Using general procedure D, *n*-BuLi (0.18 mL, 0.44 mmol, 2.4 M in hexanes) and alkene **3e** (112 mg, 0.366 mmol) in THF (1.5 mL) at –40 °C and MeOCOCl (0.1 mL, 1.28 mmol) gave the crude product. Purification by column chromatography on silica gel, eluting with pentane–EtOAc (93:7), gave carbamate **4e** (80 mg, 60%) as a clear oil; R_f 0.29 [petrol–EtOAc (4:1)]; FT-IR ν_{max} (film)/cm^{-1} 1744 (C=O), 1698 (C=O); ^{1}H NMR ($CDCl_3$, 400 MHz) δ = 7.36 (d, 2H, *J* = 9.0 Hz), 6.86 (d, 2H, *J* = 9.0 Hz), 4.85–4.68 (m, 2H), 3.98–3.87 (m, 1H), 3.82 (s, 3H), 3.71 (s, 3H), 3.42 (br, 1H), 3.10–2.86 (m, 2H), 2.51–2.18 (m, 2H), 1.37 (br s, 9H); ^{13}C{1H} NMR ($CDCl_3$, 100 MHz, $CDCl_3$, three quaternary carbon signals & one CH_2 signal not observed) δ = 172.9, 158.5, 140.2, 128.7, 113.1, 112.1, 80.8, 55.2, 52.2, 41.9, 31.2, 28.1; HRMS (ESI-TOF) *m/z*: $[M+Na]^+$ Calcd for $C_{20}H_{27}NO_5Na$ 384.1781; Found 384.1799; LRMS *m/z* (ES) 384 (100%, MNa^+).

Resolution between the enantiomers of carbamate **4e** was achieved using a Beckman system fitted with a Phenomenex Cellulose-2 column (250 mm × 4.60 mm i.d.) as the stationary phase with a mixture of *n*-hexane:isopropanol (99:1 v/v) as the mobile phase at a flow rate of 1 mL·min^{-1}; ambient temperature, detection by UV absorbance at 210 nm. Injection volume was 10 μL of the sample prepared in a 2 g·L^{-1} solution of the eluent. Under these conditions, the components were eluted at 18.2 min and 23.7 min.

1-*tert*-Butyl 2-Methyl 2-(3-Trifluoromethylphenyl)-4-methylidenepiperidine-1,2-dicarboxylate **4f**

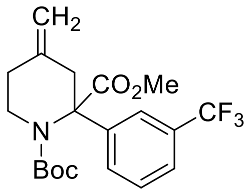

Using general procedure D, *n*-BuLi (0.19 mL, 0.44 mmol, 2.3 M in hexanes) and alkene **3f** (126 mg, 0.366 mmol) in THF (1.5 mL) at –40 °C and MeOCOCl (0.1 mL, 1.28 mmol) gave the crude product. Purification by column chromatography on silica gel, eluting with pentane–EtOAc (93:7), gave carbamate **4f** (139 mg, 94%) as a clear oil; R_f 0.30 [petrol–EtOAc (4:1)]; FT-IR ν_{max} (film)/cm^{-1} 1747 (C=O), 1698 (C=O), 1656 (C=C); ^{1}H NMR ($CDCl_3$, 400 MHz) δ = 7.78–7.64 (m, 2H), 7.59–7.41 (m, 2H), 4.75 (br s, 2H), 4.00–3.85 (m, 1H), 3.82–3.40 (m, 4H), 3.08 (d, 1H, *J* = 14.5 Hz), 2.94–2.80 (m, 1H), 2.61–2.22 (m, 2H), 1.31 (br s, 9H); ^{13}C{1H} NMR ($CDCl_3$, 100 MHz, four quaternary carbon signals, three CH_2 signals & one CH signal not observed) δ = 172.4, 139.0, 131.0–130.6 (m), 128.1, 124.22–123.61 (m), 112.5, 81.2, 52.5, 28.0; ^{19}F NMR ($CDCl_3$, 377 MHz) δ = −62.50; HRMS (ESI-TOF) *m/z*: $[M+Na]^+$ Calcd for $C_{20}H_{24}F_3NO_4Na$ 422.1550; Found 422.1571; LRMS *m/z* (ES) 300 (100%), 422 (50%, MNa^+).

Resolution between the enantiomers of carbamate **4f** was achieved using a Beckman system fitted with a Phenomenex Cellulose-2 column (250 mm × 4.60 mm i.d.) as the stationary phase with a mixture of *n*-hexane:isopropanol (99:1 v/v) as the mobile phase at a flow rate of 1 mL·min^{-1}; ambient temperature, detection by UV absorbance at 210 nm. Injection volume was 10 μL of the sample prepared in a 2 g·L^{-1} solution of the eluent. Under these conditions, the components were eluted at 6.9 min and 7.5 min.

1-*tert*-Butyl 2-Methyl 2-(3-Methoxyphenyl)-4-methylidenepiperidine-1,2-dicarboxylate **4g**

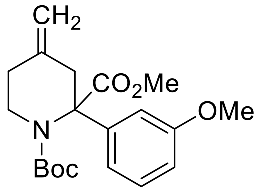

Using general procedure D, *n*-BuLi (0.19 mL, 0.44 mmol, 2.3 M in hexanes) and alkene **3g** (112 mg, 0.366 mmol) in THF (1.5 mL) at –40 °C and MeOCOCl (0.1 mL, 1.28 mmol) gave the crude product. Purification by column chromatography on silica gel, eluting with pentane–EtOAc (93:7), gave carbamate **4g** (93 mg, 70%) as a clear oil; R_f 0.24 [petrol–EtOAc (4:1)]; FT-IR ν_{max} (film)/cm^{-1} 1747 (C=O), 1698 (C=O); ^{1}H NMR ($CDCl_3$, 400 MHz) δ = ^{1}H NMR (400 MHz, $CDCl_3$) δ 7.28–7.20 (m, 1H), 7.10–7.01 (m, 2H), 6.83–6.77 (m, 1H), 4.82–4.72 (m, 2H), 4.00–3.86 (m, 1H), 3.82 (s, 3H), 3.72 (s, 3H), 3.50 (br, 1H), 3.09–2.84 (m, 2H), 2.57–2.16 (m, 2H), 1.35 (br s, 9H); ^{13}C{1H} NMR ($CDCl_3$, 100 MHz, three quaternary carbon signals & one CH_2 signal not observed) δ = 172.5, 159.2, 140.0, 128.6, 119.8, 113.6, 112.2, 112.1, 80.8, 55.2, 52.2, 41.9, 30.8, 28.1; HRMS (ESI-TOF) *m/z*: [M+Na]$^+$ Calcd for $C_{20}H_{27}NO_5Na$ 384.1781; Found 384.1792; LRMS *m/z* (ES) 384 (100%, MNa^+).

Resolution between the enantiomers of carbamate **4g** was achieved using a Beckman system fitted with a Phenomenex Cellulose-2 column (250 mm × 4.60 mm i.d.) as the stationary phase with a mixture of *n*-hexane:isopropanol (99:1 v/v) as the mobile phase at a flow rate of 1 mL·min^{-1}; ambient temperature, detection by UV absorbance at 210 nm. Injection volume was 10 µL of the sample prepared in a 2 g·L^{-1} solution of the eluent. Under these conditions, the components were eluted at 18.6 min and 22.5 min.

1-*tert*-Butyl 2-Methyl 2-(3,4-Dimethoxyphenyl)-4-methylidenepiperidine-1,2-dicarboxylate **4h**

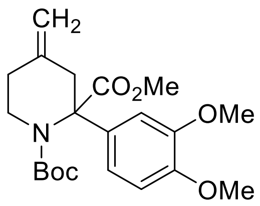

Using general procedure D, *n*-BuLi (0.19 mL, 0.44 mmol, 2.3 M in hexanes) and alkene **3h** (123 mg, 0.366 mmol) in THF (1.5 mL) at –40 °C and MeOCOCl (0.1 mL, 1.28 mmol) gave the crude product. Purification by column chromatography on silica gel, eluting with pentane–EtOAc (3:1), gave carbamate **4h** (96 mg, 66%) as a clear oil; R_f 0.54 [petrol–EtOAc (1:1)]; FT-IR ν_{max} (film)/cm^{-1} 1735 (C=O), 1699 (C=O), 1656 (C=C); ^{1}H NMR ($CDCl_3$, 400 MHz) δ = 7.06 (d, 1H, *J* = 2.0 Hz), 6.99 (dd, 1H, *J* = 8.5, 2.0 Hz), 6.82 (d, 1H, *J* = 8.5 Hz), 4.78 (br s, 2H), 4.00–3.81 (m, 7H), 3.71 (s, 3H), 3.41 (br, 1H), 3.08–2.92 (m, 2H), 2.58–2.14 (m, 2H), 1.38 (br s, 9H); ^{13}C{1H} NMR ($CDCl_3$, 100 MHz, one quaternary carbon signal & one CH_2 signal not observed) δ = 172.8, 155.8, 148.2, 148.0, 140.3, 120.0, 112.1, 111.2, 110.3, 80.8, 67.4, 55.9, 55.8, 52.2, 42.1, 31.2, 28.2; HRMS (ESI-TOF) *m/z*: $[M+Na]^+$ Calcd for $C_{21}H_{29}NO_6Na$ 414.1887; Found 414.1902; LRMS *m/z* (ES) 208 (5%), 292 (50%), 414 (100%, MNa^+).

Resolution between the enantiomers of carbamate **4h** was achieved using a Beckman system fitted with a Phenomenex Cellulose-1 column (250 mm × 4.60 mm i.d.) as the stationary phase with a mixture of *n*-hexane:isopropanol (97:3 v/v) as the mobile phase at a flow rate of 1 mL·min^{-1}; ambient temperature, detection by UV absorbance at 210 nm. Injection volume was 10 μL of the sample prepared in a 2 g·L^{-1} solution of the eluent. Under these conditions, the components were eluted at 14.1 min and 17.5 min.

tert-Butyl 2-Methyl-4-methylidene-2-phenylpiperidine-1-carboxylate **5a**

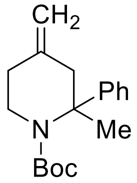

Using general procedure D, *n*-BuLi (0.18 mL, 0.44 mmol, 2.4 M in hexanes) and alkene **3a** (100 mg, 0.366 mmol) in THF (1.5 mL) at –40 °C and MeI (0.08 mL, 1.28 mmol) gave the crude product. Purification by column chromatography on silica gel, eluting with pentane–EtOAc (9:1), gave carbamate **5a** (96 mg, 90%) as a clear oil; R_f 0.28 [petrol–EtOAc (9:1)]; FT-IR ν_{max} (film)/cm^{-1} 1683 (C=O), 1651 (C=C); ^{1}H NMR ($CDCl_3$, 400 MHz) δ = 7.34–7.27 (m, 4H), 7.24–7.18 (m, 1H), 4.86 (s, 1H), 4.80 (s, 1H), 4.07–3.93 (m, 1H), 3.81–3.69 (m, 1H), 2.70 (d, 1H, *J* = 14.5 Hz), 2.64–2.55 (m, 2H), 2.30 (d, 1H, *J* = 14.5 Hz), 1.76 (s, 3H), 1.10 (s, 9H); ^{13}C{1H} NMR (100 MHz, $CDCl_3$) δ = 155.4, 149.4, 141.4, 127.9, 125.9, 124.4, 111.3, 79.6, 60.3, 49.7, 40.9, 30.4, 28.0, 25.0; HRMS (ESI-TOF) *m/z*: [M+Na]$^+$ Calcd for $C_{18}H_{25}NO_2Na$ 310.1778; Found 310.1789; LRMS *m/z* (ES) 145 (20%), 232 (100%), 310 (30%, MNa$^+$).

tert-Butyl 2-(3-Methylbut-2-en-1-yl)-4-methylidene-2-phenylpiperidine-1-carboxylate **6a**

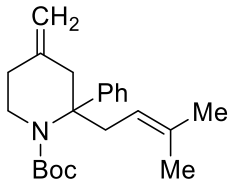

Using general procedure D, *n*-BuLi (0.18 mL, 0.44 mmol, 2.4 M in hexanes) and alkene **3a** (100 mg, 0.366 mmol) in THF (1.5 mL) at –40 °C and prenyl bromide (0.15 mL, 1.28 mmol) gave the crude product. Purification by column chromatography on silica gel, eluting with pentane−EtOAc (19:1) gave carbamate **6a** (112 mg, 89%) as a clear oil; R_f 0.52 [petrol−EtOAc

(4:1)]; FT-IR ν_{max} (film)/cm^{-1} 1687 (C=O), 1654 (C=C); ^{1}H NMR ($CDCl_3$, 400 MHz) δ = 7.37–7.14 (m, 5H), 5.33–5.23 (m, 1H), 4.72 (s, 1H), 4.54 (s, 1H), 4.27–4.13 (m, 1H), 3.43–3.31 (m, 1H), 3.14 (dd, 1H, *J* = 14.0, 6.0 Hz), 2.96 (d, 1H, *J* = 14.5 Hz), 2.83 (dd, 1H, *J* = 14.0, 9.0 Hz), 2.71–2.45 (m, 2H), 2.12 (d, 1H, *J* = 14.5 Hz), 1.79 (s, 3H), 1.72 (s, 3H), 1.14 (s, 9H); ^{13}C{1H} NMR ($CDCl_3$, 100 MHz) δ = 155.4, 148.1, 141.0, 134.8, 127.5, 125.7, 125.0, 119.6, 111.6, 79.6, 63.6, 45.6, 41.0, 37.4, 30.1, 28.1, 26.2, 18.3; HRMS (ESI-TOF) *m/z*: [M+Na]$^+$ Calcd for $C_{22}H_{31}NO_2Na$ 364.2247; Found 364.2263; LRMS *m/z* (ES) 286 (100%), 364 (35%, MNa$^+$).

tert-Butyl 2-(3-Bromopropyl)-4-methylidene-2-phenylpiperidine-1-carboxylate **7a**

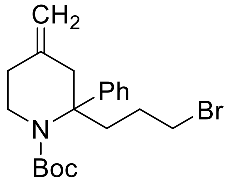

Using general procedure D, *n*-BuLi (0.18 mL, 0.44 mmol, 2.4 M in hexanes) and alkene **3a** (100 mg, 0.366 mmol) in THF (1.5 mL) at –40 °C and 1,3-dibromopropane (0.13 mL, 1.28 mmol) gave the crude product. Purification by column chromatography on silica gel, eluting with pentane–EtOAc (93:7), gave carbamate **7a** (102 mg, 70%) as a clear oil; R_f 0.61 [petrol–EtOAc (4:1)]; FT-IR ν_{max} (film)/cm^{-1} 1681 (C=O); ^{1}H NMR ($CDCl_3$, 400 MHz) δ = 7.37–7.13 (m, 5H), 4.75 (s, 1H), 4.63 (s, 1H), 4.20–4.06 (m, 1H), 3.58–3.44 (m, 3H), 2.87 (d, 1H, *J* = 14.5 Hz), 2.74–2.45 (m, 3H), 2.31 (d, 1H, *J* = 14.5 Hz), 2.20 (td, 1H, *J* = 12.5, 4.0 Hz), 2.16–1.88 (m, 2H), 1.17 (s, 9H); ^{13}C{1H} NMR ($CDCl_3$, 100 MHz) δ = 155.4, 148.1, 140.6, 127.7, 126.0, 124.8, 112.0, 79.9, 62.8, 46.6, 41.1, 39.1, 34.3, 30.2, 28.1, 27.8; HRMS (ESI-TOF) *m/z*: [M+Na]$^+$ Calcd for $C_{20}H_{28}{}^{79}BrNO_2Na$ 416.1196; Found 416.1204; LRMS *m/z* (ES) 416 (100%, MNa$^+$ for ^{79}Br), 417 (20%), 418 (100%, MNa$^+$ for ^{81}Br), 419 (20%).

tert-Butyl 2-(Tributylstannyl)-4-methylidene-2-phenylpiperidine-1-carboxylate **8a**

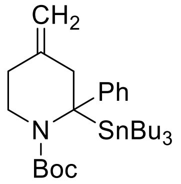

Using general procedure D, *n*-BuLi (0.18 mL, 0.44 mmol, 2.4 M in hexanes) and alkene **3a** (100 mg, 0.366 mmol) in THF (1.5 mL) at –40 °C and Bu_3SnCl (0.20 mL, 0.73 mmol) gave the crude product. Purification by column chromatography on a mixture of silica gel and K_2CO_3 (10% w/w), eluting with pentane–EtOAc (99:1), gave carbamate **8a** (181 mg, 87%) as a clear oil; R_f 0.59 [petrol–EtOAc (19:1)]; FT-IR ν_{max} (film)/cm^{-1} 1667 (C=O); ^{1}H NMR ($CDCl_3$, 400 MHz) δ = 7.32–7.21 (m, 2H), 7.08–6.92 (m, 3H), 4.84–4.72 (m, 2H), 4.07–3.90 (m, 1H), 3.23–3.09 (m, 1H), 2.89–2.63 (m, 2H), 2.25 (dt, 1H, *J* = 13.0, 6.5 Hz), 2.04 (d, 1H, *J* = 13.0 Hz), 1.53 (s, 9H), 1.41–1.16 (m, 12H), 0.85 (t, 9H, *J* = 7.5 Hz), 0.80–0.58 (m, 6H); ^{13}C{1H} NMR ($CDCl_3$, 100 MHz) δ = 157.3, 144.8, 143.0, 128.1, 125.0, 123.7, 110.6, 80.1, 58.8, 42.1, 40.6, 34.3, 29.0, 28.4, 27.7, 13.7, 13.2; HRMS (ESI-TOF) *m/z*: [M+Na]$^+$ Calcd for $C_{29}H_{49}NO_2SnNa$ 586.2677; Found 586.2682; LRMS *m/z* (ES) 360 (100%), 381 (30%), 586 (30%, MNa$^+$).

tert-Butyl 2-(Trimethylsilyl)-4-methylidene-2-phenylpiperidine-1-carboxylate **9a**

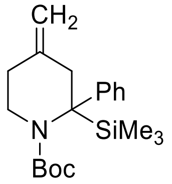

Using general procedure D, *n*-BuLi (0.18 mL, 0.44 mmol, 2.4 M in hexanes) and alkene **3a** (100 mg, 0.366 mmol) in THF (1.5 mL) at –40 °C and Me_3SiCl (0.16 mL, 1.28 mmol) gave

the crude product. Purification by column chromatography on silica gel, eluting with pentane–EtOAc (19:1), gave carbamate **9a** (89 mg, 70%) as a clear oil; R_f 0.69 [petrol–EtOAc (4:1)]; FT-IR ν_{max} (film)/cm^{-1} 1687 (C=O), 1652 (C=C); ^{1}H NMR ($CDCl_3$, 400 MHz) δ = 7.33–7.25 (m, 2H), 7.17–7.04 (m, 3H), 4.87–4.75 (m, 2H), 4.01–3.87 (m, 1H), 3.02 (d, 1H, *J* = 14.0 Hz), 2.73–2.54 (m, 2H), 2.29–2.16 (m, 1H), 2.03 (d, 1H, *J* = 14.0 Hz), 1.54 (s, 9H), −0.02 (s, 9H); ^{13}C{1H} NMR ($CDCl_3$, 100 MHz) δ = 156.1, 142.9, 142.0, 127.7, 127.5, 125.0, 111.3, 79.7, 57.8, 42.5, 38.5, 34.1, 28.4, 0.9; HRMS (ESI-TOF) *m/z*: $[M+H]^+$ Calcd for $C_{20}H_{32}NO_2Si$ 346.2197; Found 346.2212; LRMS *m/z* (ES) 274 (80%), 346 (100%, MH^+).

2.6 Kinetic resolution of racemic substrates 3a–h

Optimization of the kinetic resolution was carried out with substrate **3a** and (+)-sparteine:

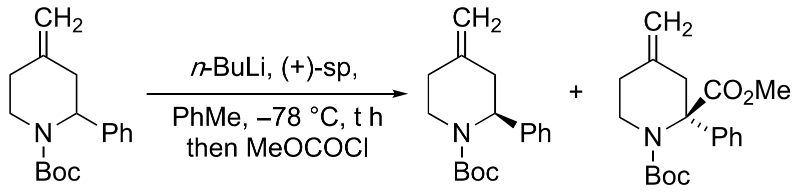

(±)-**3a** (*S*)-**3a** (*R*)-**4a**

n-BuLi (2.1 to 2.4 M in hexanes) was added to the alkene **3a** (1 eq) and (+)-sparteine (freshly distilled) in dry PhMe (0.25 M) at −78 °C (Table S1). After time t (h), MeOCOCl (2 eq) was added and the mixture was warmed to rt over 16 h. MeOH (1 mL) was added and the solvent was evaporated under reduced pressure. The crude product was purified by column chromatography on silica gel, eluting with petrol−EtOAc (93:7), to give recovered alkene (*S*)-**3a** and carbamate (*R*)-**4a**. The enantiomeric ratio of both compounds was determined by CSP-HPLC as described above.

Table S1 Optimization of the kinetic resolution

Entry	(+)-sp eq	*n*-BuLi eq	t h	(*S*)-**3a** % Yield	(*S*)-**3a** er	(*R*)-**4a** % Yield	(*R*)-**4a** er	S
1	0.7	0.7	2 h	61%	88:12	35%	91:9	-
2	0.85	0.85	2 h	43%	94:6	43%	82:18	14
3	1	1	2 h	43%	90:10	24%	80:20	10
4	0.9	0.8	2 h	42%	96:4	54%	70:30	16
5	0.9	0.8	1 h	45%	92:8	35%	90:10	14
6[a]	0.9	0.8	1 h	41%	97:3	58%	73:27	16

[a]Entries 1–5 on 100 mg **3a**; entry 6 on 300 mg **3a** as detailed on page S-38

Resolution of alkene **3a** with (+)-sparteine

tert-Butyl (2*S*)-4-Methylidene-2-phenylpiperidine-1-carboxylate (*S*)-**3a** and 1-*tert*-Butyl 2-Methyl (2*R*)-4-Methylidene-2-phenylpiperidine-1,2-dicarboxylate (*R*)-**4a**

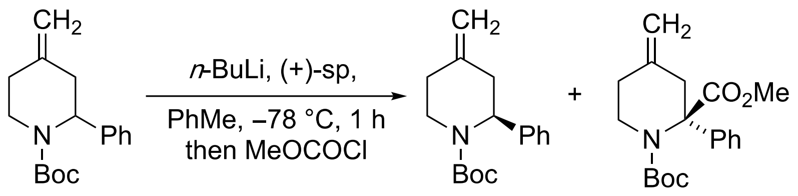

(±)-**3a** (*S*)-**3a** (*R*)-**4a**

Reaction with 300 mg of alkene **3a**:

n-BuLi (0.40 mL, 0.88 mmol, 2.2 M in hexanes) was added to a mixture of (+)-sparteine (231 mg, 0.988 mmol) and the racemic alkene **3a** (300 mg, 1.10 mmol) in dry PhMe (4.4 mL) at –78 °C. After 1 h, MeOCOCl (0.17 mL, 2.2 mmol) was added and the mixture was allowed to warm to rt over 16 h then MeOH (3 mL) was added. The solvent was evaporated, and the residue was purified by column chromatography on silica gel, eluting with petrol–EtOAc (93:7), to give recovered alkene (*S*)-**3a** (122 mg, 41%) as a clear oil; data as above; the enantiomeric ratio was determined to be 97:3 by CSP-HPLC as described above (major component eluted at 12.6 min); $[\alpha]_D^{23}$ –70 (*c* 1.0, $CHCl_3$). In addition, the carbamate (*R*)-**4a** (210 mg, 58%) was isolated as a clear oil, data as above; the enantiomeric ratio was determined to be 73:27 by CSP-HPLC (major component eluted at 10.6 min); $[\alpha]_D^{23}$ +9 (*c* 1.0, $CHCl_3$).

Reaction with 2 g of alkene **3a**:

n-BuLi (2.4 mL, 5.8 mmol, 2.4 M in hexanes) was added to a mixture of (+)-sparteine (1.6 g, 6.6 mmol) and the racemic alkene **3a** (2.0 g, 7.3 mmol) in dry PhMe (29 mL) at –78 °C. After 1 h, MeOCOCl (1.1 mL, 15 mmol) was added and the mixture was allowed to warm to rt over 16 h then MeOH (15 mL) was added. The solvent was evaporated, and the residue was diluted with Et_2O (150 mL) and water (60 mL). The aqueous layer was adjusted to pH 1 using aq HCl

(2 M) and the organic layer was separated, dried over anhydrous $MgSO_4$, filtered and concentrated to give the crude product. Purification by column chromatography on silica gel, eluting with petrol–EtOAc (23:2), to give recovered alkene (*S*)-**3a** (0.6 g, 30%) as a clear oil; data as above; the enantiomeric ratio was determined to be 99:1 by CSP-HPLC as described above (major component eluted at 12.6 min); $[\alpha]_D^{23}$ –84 (*c* 1.0, $CHCl_3$). In addition, the carbamate (*R*)-**4a** (1.6 g, 66%) was isolated as a clear oil, data as above; the enantiomeric ratio was determined to be 72:28 by CSP-HPLC (major component eluted at 10.9 min); $[\alpha]_D^{23}$ +7 (*c* 1.0, $CHCl_3$).

NB - The aqueous layer from the extraction was adjusted to pH 14 using NaOH pellets and Et_2O (150 mL) was added. The organic layer was separated, dried over anhydrous $MgSO_4$, filtered and the solvent was evaporated to give crude recovered (+)-sparteine (1.5 g) as a yellow oil.

Resolution of alkene **3a** with (–)-sparteine

tert-Butyl (2*R*)-4-Methylidene-2-phenylpiperidine-1-carboxylate (*R*)-**3a** and 1-*tert*-Butyl 2-Methyl (2*S*)-4-Methylidene-2-phenylpiperidine-1,2-dicarboxylate (*S*)-**4a**

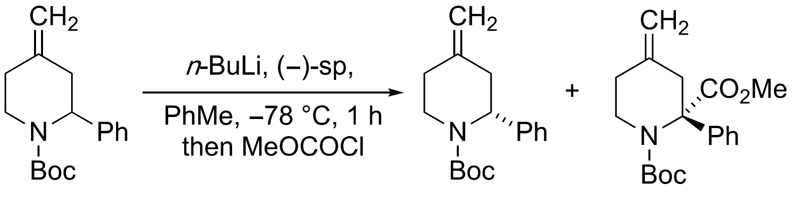

(±)-**3a** (*R*)-**3a** (*S*)-**4a**

n-BuLi (0.13 mL, 0.29 mmol, 2.3 M in hexanes) was added to a mixture of (–)-sparteine (77 mg, 0.33 mmol) and the racemic alkene **3a** (100 mg, 0.366 mmol) in dry PhMe (1.5 mL) at –78 °C. After 1 h, MeOCOCl (0.06 mL, 0.7 mmol) was added and the mixture was allowed to warm to rt over 16 h then MeOH (1 mL) was added. The solvent was evaporated, and the

residue was purified by column chromatography on silica gel, eluting with petrol–EtOAc (93:7), to give recovered alkene (*R*)-**3a** (39 mg, 39%) as a clear oil; data as above; the enantiomeric ratio was determined to be 99:1 by CSP-HPLC as described above (major component eluted at 5.6 min); $[\alpha]_D^{25}$ +81 (*c* 1.2, $CHCl_3$). In addition, the carbamate (*S*)-**4a** (70 mg, 57%) was isolated as a clear oil, data as above; the enantiomeric ratio was determined to be 81:19 by CSP-HPLC (major component eluted at 12.2 min); $[\alpha]_D^{25}$ –11 (*c* 1.1, $CHCl_3$).

Resolution of alkene **3b** with (+)-sparteine

tert-Butyl (2*S*)-2-(4-Fluorophenyl)-4-methylidenepiperidine-1-carboxylate (*S*)-**3b** and 1-*tert*-Butyl 2-Methyl (2*R*)-2-(4-Fluorophenyl)-4-methylidenepiperidine-1,2-dicarboxylate (*R*)-**4b**

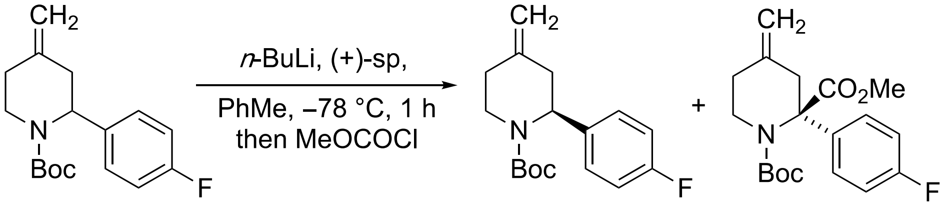

(±)-**3b** (*S*)-**3b** (*R*)-**4b**

Reaction with 108 mg of alkene **3b**:

n-BuLi (0.13 mL, 0.29 mmol, 2.3 M in hexanes) was added to a mixture of (+)-sparteine (77 mg, 0.33 mmol) and the racemic alkene **3b** (108 mg, 0.366 mmol) in dry PhMe (1.5 mL) at –78 °C. After 1 h, MeOCOCl (0.06 mL, 0.7 mmol) was added and the mixture was allowed to warm to rt over 16 h then MeOH (1 mL) was added. The solvent was evaporated, and the residue was purified by column chromatography on silica gel, eluting with petrol–EtOAc (47:3), to give recovered alkene (*S*)-**3b** (51 mg, 47%) as a clear oil; data as above; the enantiomeric ratio was determined to be 94:6 by CSP-HPLC as described above (major component eluted at 11.2 min); $[\alpha]_D^{23}$ –60 (*c* 1.0, $CHCl_3$). In addition, the carbamate (*R*)-**4b**

(45 mg, 35%) was isolated as a clear oil, data as above; the enantiomeric ratio was determined to be 88:12 by CSP-HPLC (major component eluted at 11.4 min); $[\alpha]_D^{23}$ +8 (*c* 1.0, $CHCl_3$).

Reaction with 4 g of alkene **3b**:

n-BuLi (4.8 mL, 11 mmol, 2.3 M in hexanes) was added to a mixture of (+)-sparteine (2.9 g, 12 mmol) and the racemic alkene **3b** (4.0 g, 14 mmol) in dry PhMe (62 mL) at –78 °C. After 1 h, MeOCOCl (2.7 mL, 34 mmol) was added and the mixture was allowed to warm to rt over 16 h then MeOH (20 mL) was added. The solvent was evaporated, and the residue was diluted with Et_2O (150 mL) and water (75 mL). The aqueous layer was adjusted to pH 1 using aq HCl (2 M) and the organic layer was separated, dried over anhydrous $MgSO_4$, filtered and concentrated to give the crude product. Purification by column chromatography on silica gel, eluting with *n*-hexane–EtOAc (93:7), to give recovered alkene (*S*)-**3b** (1.4 g, 35%) as a clear oil; data as above; the enantiomeric ratio was determined to be 94:6 by CSP-HPLC as described above (major component eluted at 6.1 min on Agilent system fitted with a Chiralcel OX-H column). In addition, the carbamate (*R*)-**4b** (2.7 g, 56%) was isolated as a clear oil, data as above; the enantiomeric ratio was determined to be 75:25 by CSP-HPLC (major component eluted at 8.6 min on Agilent system fitted with a Chiral Art Cellulose-C column.

NB - The aqueous layer from the extraction was adjusted to pH 14 using NaOH pellets and Et_2O (150 mL) was added. The organic layer was separated, dried over anhydrous $MgSO_4$, filtered and the solvent was evaporated to give crude recovered (+)-sparteine (2.9 g) as a yellow oil.

Resolution of alkene **3c** with (+)-sparteine

tert-Butyl (2*S*)-2-(4-Chlorophenyl)-4-methylidenepiperidine-1-carboxylate (*S*)-**3c** and 1-*tert*-Butyl 2-Methyl (2*R*)-2-(4-Chlorophenyl)-4-methylidenepiperidine-1,2-dicarboxylate (*R*)-**4c**

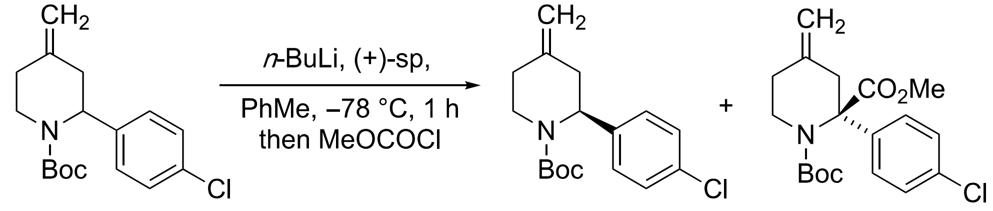

(±)-**3c** (*S*)-**3c** (*R*)-**4c**

n-BuLi (0.13 mL, 0.29 mmol, 2.3 M in hexanes) was added to a mixture of (+)-sparteine (77 mg, 0.33 mmol) and the racemic alkene **3c** (114 mg, 0.366 mmol) in dry PhMe (1.5 mL) at –78 °C. After 1 h, MeOCOCl (0.06 mL, 0.7 mmol) was added and the mixture was allowed to warm to rt over 16 h then MeOH (1 mL) was added. The solvent was evaporated, and the residue was purified by column chromatography on silica gel, eluting with petrol–EtOAc (47:3), to give recovered alkene (*S*)-**3c** (40 mg, 35%) as a clear oil; data as above; the enantiomeric ratio was determined to be 99:1 by CSP-HPLC as described above (major component eluted at 6.6 min); $[\alpha]_D^{25}$ –78 (*c* 1.2, $CHCl_3$). In addition, the carbamate (*R*)-**4c** (77 mg, 57%) was isolated as a clear oil, data as above; the enantiomeric ratio was determined to be 76:24 by CSP-HPLC (major component eluted at 8.6 min); $[\alpha]_D^{27}$ +4 (*c* 1.0, $CHCl_3$).

Resolution of alkene **3d** with (+)-sparteine

tert-Butyl (2*S*)-2-(4-Methylphenyl)-4-methylidenepiperidine-1-carboxylate (*S*)-**3d** and 1-*tert*-Butyl 2-Methyl (2*R*)-2-(4-Methylphenyl)-4-methylidenepiperidine-1,2-dicarboxylate (*R*)-**4d**

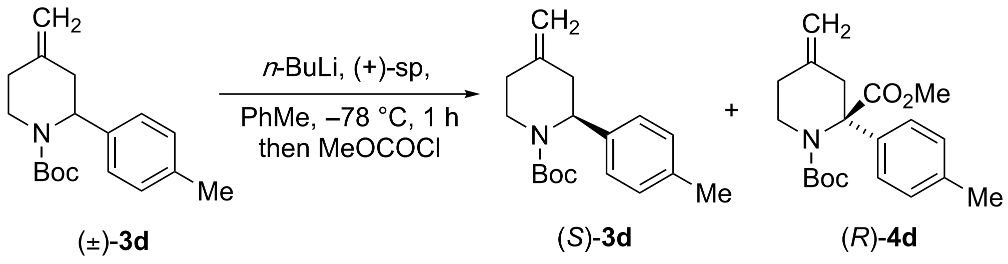

n-BuLi (0.13 mL, 0.29 mmol, 2.3 M in hexanes) was added to a mixture of (+)-sparteine (77 mg, 0.33 mmol) and the racemic alkene **3d** (106 mg, 0.366 mmol) in dry PhMe (1.5 mL) at –78 °C. After 1 h, MeOCOCl (0.06 mL, 0.7 mmol) was added and the mixture was allowed to warm to rt over 16 h then MeOH (1 mL) was added. The solvent was evaporated, and the residue was purified by column chromatography on silica gel, eluting with petrol–EtOAc (47:3), to give recovered alkene (*S*)-**3d** (40 mg, 38%) as a clear oil; data as above; the enantiomeric ratio was determined to be 95:5 by CSP-HPLC as described above (major component eluted at 9.3 min); $[\alpha]_D^{23}$ –76 (*c* 1.0, $CHCl_3$). In addition, the carbamate (*R*)-**4d** (71 mg, 56%) was isolated as a clear oil, data as above; the enantiomeric ratio was determined to be 82:18 by CSP-HPLC (major component eluted at 10.2 min); $[\alpha]_D^{27}$ +12 (*c* 1.0, $CHCl_3$).

Resolution of alkene **3e** with (+)-sparteine

tert-Butyl (2*S*)-2-(4-Methoxyphenyl)-4-methylidenepiperidine-1-carboxylate (*S*)-**3e** and 1-*tert*-Butyl 2-Methyl (2*R*)-2-(4-Methoxyphenyl)-4-methylidenepiperidine-1,2-dicarboxylate (*R*)-**4e**

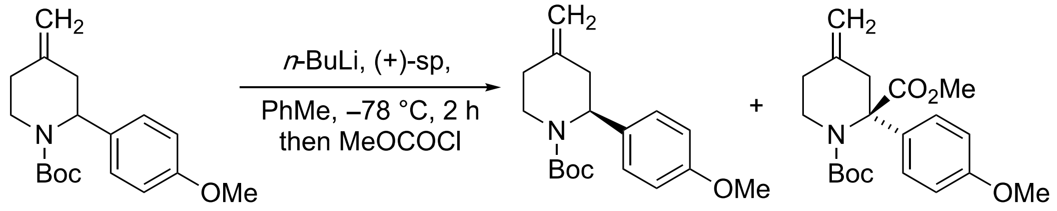

(±)-**3e** (*S*)-**3e** (*R*)-**4e**

n-BuLi (0.17 mL, 0.37 mmol, 2.2 M in hexanes) was added to a mixture of (+)-sparteine (95 mg, 0.41 mmol) and the racemic alkene **3e** (112 mg, 0.366 mmol) in dry PhMe (1.5 mL) at –78 °C. After 2 h, MeOCOCl (0.06 mL, 0.7 mmol) was added and the mixture was allowed to warm to rt over 16 h then MeOH (1 mL) was added. The solvent was evaporated, and the residue was purified by column chromatography on silica gel, eluting with petrol–EtOAc (22:3), to give recovered alkene (*S*)-**3e** (35 mg, 31%) as a clear oil; data as above; the enantiomeric ratio was determined to be 96:4 by CSP-HPLC as described above (major component eluted at 12.6 min); $[\alpha]_D^{26}$ –71 (*c* 1.1, $CHCl_3$). In addition, the carbamate (*R*)-**4e** (88 mg, 66%) was isolated as a clear oil, data as above; the enantiomeric ratio was determined to be 70:30 by CSP-HPLC (major component eluted at 18.4 min); $[\alpha]_D^{26}$ +7 (*c* 1.1, $CHCl_3$).

Resolution of alkene **3f** with (+)-sparteine

tert-Butyl (2*S*)-4-Methylidene-2-[3-(trifluoromethyl)phenyl]piperidine-1-carboxylate (*S*)-**3f** and 1-*tert*-Butyl 2-Methyl (2*R*)-4-Methylidene-2-[3-(trifluoromethyl)phenyl]piperidine-1,2-dicarboxylate (*R*)-**4f**

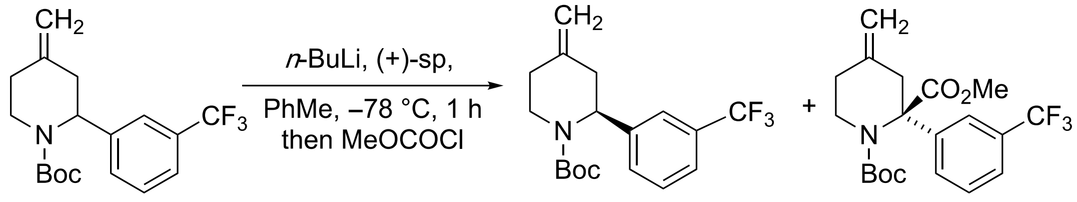

(±)-**3f** (*S*)-**3f** (*R*)-**4f**

n-BuLi (0.13 mL, 0.29 mmol, 2.3 M in hexanes) was added to a mixture of (+)-sparteine (77 mg, 0.33 mmol) and the racemic alkene **3f** (126 mg, 0.366 mmol) in dry PhMe (1.5 mL) at –78 °C. After 1 h, MeOCOCl (0.06 mL, 0.7 mmol) was added and the mixture was allowed to warm to rt over 16 h then MeOH (1 mL) was added. The solvent was evaporated, and the residue was purified by column chromatography on silica gel, eluting with petrol–EtOAc (9:1), to give recovered alkene (*S*)-**3f** (45 mg, 36%) as a clear oil; data as above; the enantiomeric ratio was determined to be 97:3 by CSP-HPLC as described above (major component eluted at 5.8 min); $[\alpha]_D^{23}$ –65 (*c* 1.1, $CHCl_3$). In addition, the carbamate (*R*)-**4f** (87 mg, 59%) was isolated as a clear oil, data as above; the enantiomeric ratio was determined to be 76:24 by CSP-HPLC (major component eluted at 7.0 min); $[\alpha]_D^{26}$ +5 (*c* 1.0, $CHCl_3$).

Resolution of alkene **3g** with (+)-sparteine

tert-Butyl (2*S*)-2-(3-Methoxyphenyl)-4-methylidenepiperidine-1-carboxylate (*S*)-**3g** and 1-*tert*-Butyl 2-Methyl (2*R*)-2-(3-Methoxyphenyl)-4-methylidenepiperidine-1,2-dicarboxylate (*R*)-**4g**

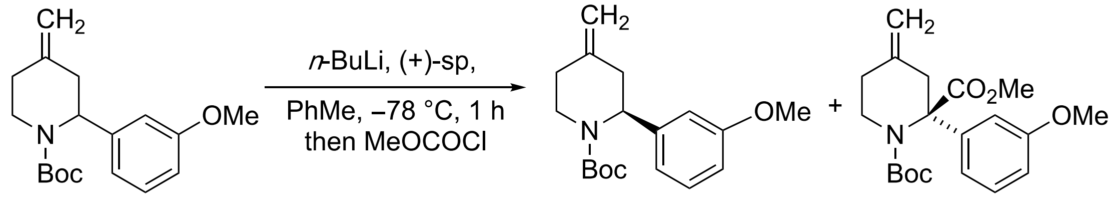

(±)-**3g** (*S*)-**3g** (*R*)-**4g**

n-BuLi (0.16 mL, 0.37 mmol, 2.3 M in hexanes) was added to a mixture of (+)-sparteine (95 mg, 0.41 mmol) and the racemic alkene **3g** (112 mg, 0.366 mmol) in dry PhMe (1.5 mL) at –78 °C. After 1 h, MeOCOCl (0.06 mL, 0.7 mmol) was added and the mixture was allowed to warm to rt over 16 h then MeOH (1 mL) was added. The solvent was evaporated, and the residue was purified by column chromatography on silica gel, eluting with petrol–EtOAc (9:1), to give recovered alkene (*S*)-**3g** (42 mg, 38%) as a clear oil; data as above; the enantiomeric ratio was determined to be 97:3 by CSP-HPLC as described above (major component eluted at 19.4 min); $[\alpha]_D^{23}$ –72 (*c* 1.0, $CHCl_3$). In addition, the carbamate (*R*)-**4g** (70 mg, 52%) was isolated as a clear oil, data as above; the enantiomeric ratio was determined to be 82:18 by CSP-HPLC (major component eluted at 18.7 min); $[\alpha]_D^{26}$ +16 (*c* 1.0, $CHCl_3$).

Resolution of alkene **3h** with (+)-sparteine

tert-Butyl (2*S*)-2-(3,4-Dimethoxyphenyl)-4-methylidenepiperidine-1-carboxylate (*S*)-**3h** and 1-*tert*-Butyl 2-Methyl (2*R*)-2-(3,4-dimethoxyphenyl)-4-methylidenepiperidine-1,2-dicarboxylate (*R*)-**4h**

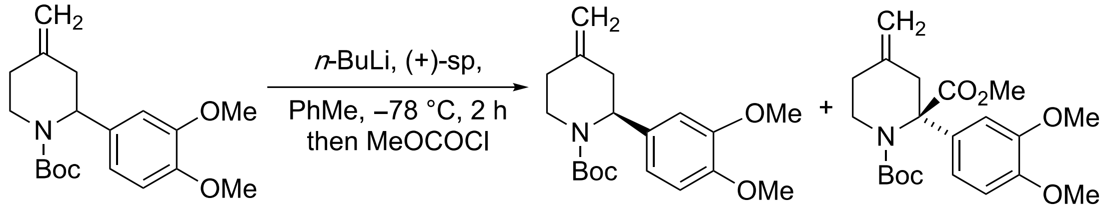

n-BuLi (0.16 mL, 0.37 mmol, 2.3 M in hexanes) was added to a mixture of (+)-sparteine (95 mg, 0.41 mmol) and the racemic alkene **3h** (123 mg, 0.366 mmol) in dry PhMe (1.5 mL) at –78 °C. After 2 h, MeOCOCl (0.06 mL, 0.7 mmol) was added and the mixture was allowed to warm to rt over 16 h then MeOH (1 mL) was added. The solvent was evaporated, and the residue was purified by column chromatography on silica gel, eluting with petrol–EtOAc (22:3), to give recovered alkene (*S*)-**3h** (35 mg, 28%) as a clear oil; data as above; the enantiomeric ratio was determined to be 85:15 by CSP-HPLC as described above (major component eluted at 8.4 min); $[\alpha]_D^{25}$ –54 (*c* 1.2, $CHCl_3$). In addition, the carbamate (*R*)-**4h** (97 mg, 66%) was isolated as a clear oil, data as above; the enantiomeric ratio was determined to be 66:33 by CSP-HPLC (major component eluted at 10.5 min); $[\alpha]_D^{25}$ +18 (*c* 1.0, $CHCl_3$).

2.7 Further functionalization of 3a and (*S*)-3a and (*S*)-3b

1-*tert*-Butyl 2-Methyl (2*S*)-4-Methylidene-2-phenylpiperidine-1,2-dicarboxylate (*S*)-**4a**

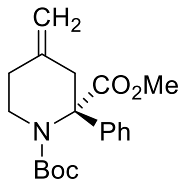

Using general procedure D, *n*-BuLi (0.09 mL, 0.22 mmol, 2.4 M in hexanes) and alkene (*S*)-**3a** (50 mg, 0.18 mmol, er 99:1) in THF (0.75 mL) at –78 °C and MeOCOCl (0.05 mL, 0.6 mmol) gave the crude product. Purification by column chromatography on silica gel, eluting with petrol–EtOAc (9:1), gave carbamate (*S*)-**4a** (47 mg, 77%) as a clear oil; data as above; the enantiomeric ratio was determined to be 99:1 by CSP-HPLC (major component eluted at 12.5 min); $[\alpha]_D^{23}$ –18 (*c* 1.0, $CHCl_3$).

(2*S*)-4-Methylidene-2-phenylpiperidine hydrochloride (*S*)-**10**

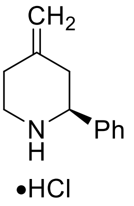

HCl (1.3 mL, 5.1 mmol, 4 M in dioxane) was added to alkene (*S*)-**3a** (139 mg, 0.508 mmol, er 99:1) in CH_2Cl_2 (5 mL) at 0 °C then the mixture was warmed to rt. After 16 h, the solvent was evaporated and the oily residue and was suspended in Et_2O. The solvent was evaporated to give hydrochloride salt (*S*)-**10** (98 mg, 92%) as an amorphous off-white solid; mp 174–176 °C [Et_2O] (no lit. mp reported); R_f 0.01 [petrol−EtOAc (1:1)]; FT-IR ν_{max} (film)/cm^{-1} 1654 (C=C): ^{1}H NMR (DMSO-d^6, 400 MHz) δ = 10.14 (br s, 1H), 9.50 (br s, 1H), 7.77–7.59 (m, 2H), 7.50–

7.36 (m, 3H), 5.02–4.88 (m, 2H), 4.30–4.14 (m, 1H), 3.44–3.28 (m, 1H), 3.07–2.91 (m, 1H), 2.83–2.67 (m, 2H), 2.66–2.37 (m, 2H); $^{13}C\{1H\}$ NMR (DMSO-d^6, 100 MHz) δ = 141.5, 137.6, 129.4, 129.2, 128.1, 112.3, 60.6, 45.4, 38.9, 30.0; HRMS (ESI-TOF) *m/z*: $[M+H]^+$ Calcd for $C_{12}H_{16}N$ 174.1284; Found 174.1284; LRMS *m/z* (ES) 174 (100%, MH^+); $[\alpha]_D^{23}$ +68 (*c* 1.0, MeOH).

(2*S*)-4-Methylidene-2-(4-fluorophenyl)piperidine hydrochloride (*S*)-**11**

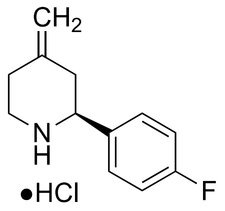

HCl (12 mL, 47 mmol, 4 M in dioxane) was added to alkene (*S*)-**3b** (1.3 mg, 4.5 mmol, er 94:6) in CH_2Cl_2 (45 mL) at 0 °C then the mixture was warmed to rt. After 16 h, the solvent was evaporated and the oily residue and was suspended in Et_2O. The solvent was evaporated to give the crude product. The crude product was recrystallized from EtOH–Et_2O to give hydrochloride salt (*S*)-**11** (0.67 g, 67%) as an amorphous white solid; mp 206–208 °C [EtOH–Et_2O]; R_f 0.01 [petrol−EtOAc (1:1)]; FT-IR ν_{max} (film)/cm^{-1} 1655 (C=C): ^1H NMR (DMSO-d^6, 400 MHz) δ = 10.15 (br s, 1H), 9.49 (br s, 1H), 7.81–7.69 (m, 2H), 7.36–7.20 (m, 2H), 5.00–4.88 (m, 2H), 4.34–4.14 (m, 1H), 3.46–3.29 (m, 1H), 3.08–2.84 (m, 1H), 2.81–2.65 (m, 2H), 2.61–2.39 (m, 2H); $^{13}C\{1H\}$ NMR (DMSO-d^6, 100 MHz) δ = 162.7 (d, *J* = 245.5 Hz), 141.4, 133.9 (d, *J* = 3.0 Hz), 130.6 (d, *J* = 8.5 Hz), 116.0 (d, *J* = 21.5 Hz), 112.3, 59.8, 45.3, 38.7, 29.9; ^{19}F NMR (377 MHz, DMSO-d^6) δ = −112.86; HRMS (ESI-TOF) *m/z*: $[M+H]^+$ Calcd for $C_{12}H_{14}FN$ 192.1183; Found 192.1174; LRMS *m/z* (ES) 192 (100%, MH^+).

tert-Butyl (2*S*)-2-(4-fluorophenyl)-4-methylidenepiperidine-1-carboxylate (*S*)-**3b**

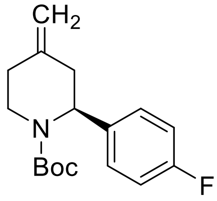

To a solution of hydrochloride salt (*S*)-**11** (72 mg, 0.32 mmol) in CH_2Cl_2 (2 mL) at 0 °C was added Et_3N (0.13 mL, 0.95 mmol) followed by Boc_2O (0.10 g, 0.47 mmol). After 30 min, the mixture was warmed to rt and was stirred for 16 h. The solvent was evaporated to give the crude product. Purification by column chromatography on silica gel, eluting with *n*-hexane−EtOAc (9:1), gave carbamate (*S*)-**3b** (82 mg, 89%) as an oil; data as above; the enantiomeric ratio was determined to be 99:1 by CSP-HPLC (major component eluted at 6.9 min* on an Agilent system fitted with a Chiralcel OX-H column); $[\alpha]_D^{25}$ −65 (0.6, $CHCl_3$).

*HPLC retention times for enantiomers were different when compared to original racemic compound (see above). A new HPLC for the racemic compound was run at the same time which gave the retention times 6.1 min and 6.9 min.

(±)-*cis*-*tert*-Butyl 4-(Hydroxymethyl)-2-phenylpiperidine-1-carboxylate **12**

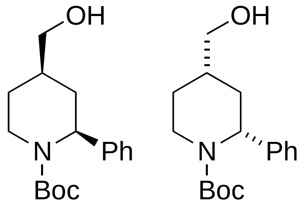

BH_3•THF complex solution (2.2 mL, 2.2 mmol, 1 M in THF) was added to alkene **3a** (100 mg, 0.366 mmol) at 0 °C. The mixture was warmed (stirrer hotplate and drysyn block) to 35 °C and stirred for 2 h. After cooling to 0 °C, aq NaOH (0.74 mL, 1.5 mmol, 2 M) was added followed

by H_2O_2 (0.41 mL, 3.5 mmol, 30% w/w). The mixture was warmed to 35 °C and stirred for 1 h. After cooling to rt, the mixture was diluted with water (60 mL) and EtOAc (60 mL). The aqueous layer was adjusted to pH 7 using aq HCl (2 M) and the organic layer was separated, dried over anhydrous $MgSO_4$, filtered and concentrated under reduced pressure to give the crude product. Purification by column chromatography on silica gel, eluting with CH_2Cl_2–MeOH (47:3), gave alcohol **12** (70 mg, 65%) as a clear oil; R_f 0.29 [CH_2Cl_2–MeOH (47:3)]; FT-IR ν_{max} (film)/cm^{-1} 3400 (O-H), 1691 (C=O); ^{1}H NMR ($CDCl_3$, 400 MHz) δ = 7.39–7.30 (m, 2H), 7.26–7.20 (m, 3H), 4.98–4.85 (m, 1H), 4.13–4.02 (m, 1H), 3.57–3.35 (m, 1H), 3.33–3.23 (m, 1H), 2.18–2.10 (m, 1H), 2.05–1.91 (m, 2H), 1.78–1.65 (m, 1H), 1.53–1.38 (m, 2H), 1.32 (s, 9H); ^{13}C{1H} NMR ($CDCl_3$, 100 MHz) δ = 155.8, 144.4, 128.4, 126.5, 125.1, 79.6, 66.4, 56.3, 38.0, 34.2, 32.9, 28.3, 25.4; HRMS (ESI-TOF) *m/z*: $[M+Na]^+$ Calcd for $C_{17}H_{25}NO_3Na$ 314.1727; Found 314.1742; LRMS *m/z* (ES) 236 (75%), 314 (100%, MNa^+). Data as reported.[5]

Resolution between the enantiomers of alcohol **12** was achieved using a Beckman system fitted with a Phenomenex Cellulose-1 column (250 mm × 4.60 mm i.d.) as the stationary phase with a mixture of *n*-hexane:isopropanol (9:1 v/v) as the mobile phase at a flow rate of 1 mL·min^{-1}; ambient temperature, detection by UV absorbance at 210 nm. Injection volume was 10 µL of the sample prepared in a 2 g·L^{-1} solution of the eluent. Under these conditions, the components were eluted at 16.9 min and 26.6 min.

tert-Butyl (2*S*,4*R*)-4-(Hydroxymethyl)-2-phenylpiperidine-1-carboxylate (2*S*,4*R*)-**12**

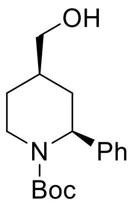

BH_3•THF complex solution (2.2 mL, 2.2 mmol, 1 M in THF) was added to alkene (*S*)-**3a** (100 mg, 0.366 mmol, er 99:1) at 0 °C. The mixture was warmed (stirrer hotplate and drysyn block) to 35 °C and stirred for 1 h. After cooling to 0 °C, aq NaOH (0.74 mL, 1.5 mmol, 2 M) was added followed by H_2O_2 (0.41 mL, 4.4 mmol, 30% w/w). The mixture was warmed to 35 °C and stirred for 1 h. After cooling to rt, the mixture was diluted with water (30 mL) and EtOAc (60 mL). The aqueous layer was adjusted to pH 7 using aq HCl (2M) and the organic layer was separated, dried over anhydrous $MgSO_4$, filtered and concentrated under reduced pressure to give the crude product. Purification by column chromatography on silica gel, eluting with CH_2Cl_2−MeOH (19:1), gave alcohol (2*S*,4*R*)-**12** (71 mg, 67%) as a clear oil; data as above; the enantiomeric ratio was determined to be 99:1 by CSP-HPLC (major component eluted at 17.0 min); $[\alpha]_D^{23}$ −62 (*c* 1.0, $CHCl_3$).

tert-Butyl (2*S*,4*R*)-4-{[(4-Bromobenzoyl)oxy]methyl}-2-phenylpiperidine-1-carboxylate (2*S*,4*R*)-**13**

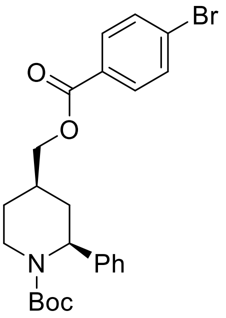

Et_3N (0.15 mL, 1.1. mmol) was added to alcohol (2*S*,4*R*)-**12** (70 mg, 0.24 mmol, er 99:1) and DMAP (2 mg, 0.01 mmol) in CH_2Cl_2 (0.5 mL) at 0 °C, followed by 4-bromobenzoyl chloride (116 mg, 0.528 mmol) in CH_2Cl_2 (0.23 mL). The mixture was warmed to rt over 16 h and the solvent was evaporated to give the crude product. Purification by column chromatography on silica gel, eluting with petrol–EtOAc (9:1), gave ester (2*S*,4*R*)-**13** (83 mg, 73%) as a white amorphous solid; mp 94–96 °C [petrol–EtOAc]; R_f 0.38 [petrol–EtOAc (7:3)]; FT-IR ν_{max} (film)/cm^{-1} 1720 (C=O), 1687 (C=O); ^{1}H NMR ($CDCl_3$, 400 MHz) δ = 7.88 (d, 2H, *J* = 8.5 Hz), 7.60 (d, 2H, *J* = 8.5 Hz), 7.36–7.30 (m, 2H), 7.26–7.20 (m, 3H), 5.01–4.90 (m, 1H), 4.23–4.06 (m, 3H), 3.42–3.27 (m, 1H), 2.34–2.01 (m, 3H), 1.90–1.75 (m, 1H), 1.56–1.45 (m, 1H), 1.33 (s, 9H); ^{13}C{1H} NMR ($CDCl_3$, 100 MHz) δ = 165.7, 155.7, 143.9, 131.8, 131.1, 129.0, 128.5, 128.2, 126.7, 125.2, 79.8, 68.1, 56.1, 37.8, 33.0, 31.2, 28.3, 25.7; HRMS (ESI-TOF) *m/z*: $[M+Na]^+$ Calcd for $C_{24}H_{28}{}^{79}BrNO_4Na$ 496.1094; Found 496.1115; LRMS *m/z* (ES) 496 (100%, MNa^+ for ^{79}Br), 497 (20%), 498 (100%, MNa^+ for ^{81}Br), 499 (20%); $[\alpha]_D^{23}$ −16 (*c* 1.0, $CHCl_3$).

tert-Butyl (2*S*)-4-Oxo-2-phenylpiperidine-1-carboxylate (*S*)-**2a**

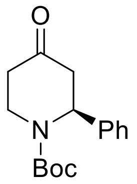

Ozone was bubbled through a solution of alkene (*S*)-**3a** (300 mg, 1.10 mmol, er 99:1) in CH_2Cl_2 (99 mL) and MeOH (11 mL) at –78 °C for 1 h. Me_2S (1.6 mL, 22 mmol) was added dropwise and the mixture was warmed to rt over 16 h. The solvent was evaporated to give the crude product. Purification by column chromatography on silica gel, eluting with petrol−EtOAc (15:5), gave piperidone (*S*)-**2a** (275 mg, 91%) as a clear oil; data as above; the enantiomeric ratio was determined to be 99:1 by CSP-HPLC (major component eluted at 12.0 min); $[\alpha]_D^{23}$ –71 (*c* 1.0, $CHCl_3$), lit.[14] $[\alpha]_D^{20}$ –68.2 (*c* 1.95, $CHCl_3$).

tert-Butyl 4,4-Difluoro-2-phenylpiperidine-1-carboxylate **14**

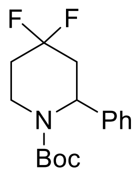

Et_2NSF_3 (1.5 ml, 11 mmol) was added to piperidone **2a** (1.0 g, 3.6 mmol) in CH_2Cl_2 (6 mL) at 0 °C. After 4 h, the mixture was warmed to rt and stirred for 16 h. The mixture was cooled to 0 °C and carefully quenched with saturated aq $NaHCO_3$. The mixture was diluted with water (50 mL) and CH_2Cl_2 (100 mL). The organic layer was separated, dried over anhydrous $MgSO_4$, filtered and the solvent was evaporated to give the crude product. Purification by column

chromatography on silica gel, eluting with petrol–EtOAc (19:1), gave difluoride **14** (0.69 g, 64%) as a clear oil; R_f 0.43 [petrol–EtOAc (4:1)]; FT-IR ν_{max} (film)/cm^{-1} 1693 (C=O); ^{1}H NMR ($CDCl_3$, 400 MHz) δ = 7.43–7.33 (m, 2H), 7.32–7.22 (m, 3H), 5.63 (br d, 1H, *J* = 6.0 Hz), 4.34–4.19 (m, 1H), 3.20–3.01 (m, 1H), 2.90–2.73 (m, 1H), 2.42–2.21 (m, 1H), 2.09–1.85 (m, 2H), 1.48 (s, 9H); ^{13}C{1H} NMR ($CDCl_3$, 100 MHz) δ = 155.0, 139.0, 128.5, 126.9, 125.8, 121.8 (t, *J* = 242.5 Hz), 80.7, 52.4 (d, *J* = 9.5 Hz), 37.1 (d, *J* = 9.5 Hz), 35.8 (t, *J* = 23.0 Hz), 34.0 (t, *J* = 23.5 Hz), 28.3; ^{19}F NMR ($CDCl_3$, 377 MHz) δ = −87.60 (d, *J* = 238.5 Hz), −96.15 (d, *J* = 238.5 Hz); HRMS (ESI-TOF) *m/z*: [M+Na]$^+$ Calcd for $C_{16}H_{21}F_2NO_2Na$ 320.1433; Found 320.1442; LRMS *m/z* (ES) 198 (20%), 222 (20%), 242 (100%), 320 (20%, MNa$^+$).

Resolution between the enantiomers of difluoride **14** was achieved using a Beckman system fitted with a Phenomenex Cellulose-2 column (250 mm × 4.60 mm i.d.) as the stationary phase with a mixture of *n*-hexane:isopropanol (99:1 v/v) as the mobile phase at a flow rate of 1 mL·min^{-1}; ambient temperature, detection by UV absorbance at 210 nm. Injection volume was 10 μL of the sample prepared in a 2 g·L^{-1} solution of the eluent. Under these conditions, the components were eluted at 6.0 min and 6.9 min.

tert-Butyl (2*S*)-4,4-Difluoro-2-phenylpiperidine-1-carboxylate (*S*)-**14**

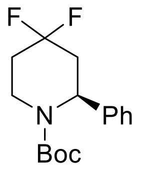

Et_2NSF_3 (1.5 ml, 11 mmol) was added to piperidone (*S*)-**2a** (275 mg, 0.998 mmol, er 99:1) in CH_2Cl_2 (1.7 mL) at 0 °C. After 2 h, the mixture was warmed to rt and stirred for 16 h. The mixture was cooled to 0 °C and carefully quenched with saturated aq $NaHCO_3$. The mixture

was diluted with water (40 mL) and CH_2Cl_2 (60 mL). The organic layer was separated, dried over anhydrous $MgSO_4$, filtered and the solvent was evaporated to give the crude product. Purification by column chromatography on silica gel, eluting with petrol–EtOAc (9:1), gave difluoride (*S*)-**14** (256 mg, 86%) as a clear oil; data as above; the enantiomeric ratio was determined to be 99:1 by CSP-HPLC (major component eluted at 6.8 min); $[\alpha]_D^{22}$ –71 (*c* 1.0, $CHCl_3$).

1-*tert*-Butyl 2-Methyl 4,4-Difluoro-2-phenylpiperidine-1,2-dicarboxylate **15**

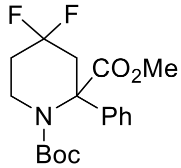

Using general procedure D, *n*-BuLi (0.22 mL, 0.44 mmol, 2.0 M in hexanes) and difluoride **14** (110 mg, 0.366 mmol) in THF (1.5 mL) at –40 °C and MeOCOCl (0.1 mL, 1.28 mmol) gave the crude product. Purification by column chromatography on silica gel, eluting with pentane–EtOAc (9:1). gave carbamate **15** (100 mg, 76%) as a clear oil; R_f 0.52 [petrol–EtOAc (4:1)]; FT-IR ν_{max} (film)/cm^{-1} 1747 (C=O), 1699 (C=O); ^{1}H NMR ($CDCl_3$, 400 MHz) δ = 7.50–7.18 (m, 5H), 4.03–3.88 (m, 1H), 3.74 (s, 3H), 3.68–3.52 (m, 1H), 2.94–2.70 (m, 2H), 2.20–1.95 (m, 2H), 1.35 (s, 9H); ^{13}C{1H} NMR ($CDCl_3$, 100 MHz, one quaternary carbon signal not observed) δ = 171.2, 155.9, 128.1, 127.5, 126.8, 121.13 (t, *J* = 243.0 Hz), 81.6, 66.9, 52.7, 41.9, 40.35 (t, *J* = 5.0 Hz), 33.5 (t, *J* = 24.0 Hz), 28.0; ^{19}F NMR ($CDCl_3$, 377 MHz) δ = −90.81 (br), −92.86 (d, *J* = 236.5 Hz); HRMS (ESI-TOF) *m/z*: [M+Na]$^+$ Calcd for $C_{18}H_{23}F_2NO_4Na$ 378.1487; Found 378.1506; LRMS *m/z* (ES) 256 (100%), 378 (60%, MNa$^+$).

Resolution between the enantiomers of carbamate **15** was achieved using a Beckman system fitted with a Phenomenex Cellulose-2 column (250 mm × 4.60 mm i.d.) as the stationary phase with a mixture of *n*-hexane:isopropanol (99:1 v/v) as the mobile phase at a flow rate of 1 mL·min^{-1}; ambient temperature, detection by UV absorbance at 210 nm. Injection volume was 10 μL of the sample prepared in a 2 g·L^{-1} solution of the eluent. Under these conditions, the components were eluted at 9.3 min and 12.2 min.

1-*tert*-Butyl 2-Methyl (2*S*)-4-Oxo-2-phenylpiperidine-1,2-dicarboxylate (*S*)-**15**

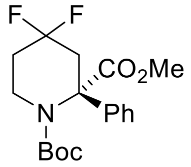

Using general procedure D, *n*-BuLi (0.10 mL, 0.24 mmol, 2.4 M in hexanes) and (*S*)-**14** (60 mg, 0.20 mmol, er 99:1) in THF (0.8 mL) at –78 °C and MeOCOCl (0.05 mL, 0.7 mmol) gave the crude product. Purification by column chromatography on silica gel, eluting with pentane–EtOAc (9:1), gave carbamate (*S*)-**15** (45 mg, 63%) as a clear oil; data as above; the enantiomeric ratio was determined to be 99:1 by CSP-HPLC (major component eluted at 12.1 min); $[\alpha]_{D}^{22}$ –12 (*c* 1.0, $CHCl_3$).

3. References

1. Bruker (2017) Apex3 v2017.3-0. Bruker AXS Inc., Madison, Wisconsin, USA.

2. L. Krause, R. Herbst-Irmer, G. M. Sheldrick, D. Stalke, J. Appl. Cryst., 2015, 48, 3.

3. G. M. Sheldrick, Acta Cryst., 2015, A71, 3.

4. G. M. Sheldrick, Acta Cryst., 2015, C71, 3.

5. O.V. Dolomanov, L. J. Bourhis, R. J. Gildea, J. A. K. Howard, H. Puschmann, J. Appl. Cryst., 2009, 42, 339.

6. A. D. Becke, J. Chem. Phys. 1993, 98, 5648.

7. M. J. Frisch, G. W. Trucks, H. B. Schlegel, G. E. Scuseria, M. A. Robb, J. R. Cheeseman, G. Scalmani, V. Barone, B. Mennucci, G. A. Petersson, H. Nakatsuji, M. Caricato, X. Li, H. P. Hratchian, A. F. Izmaylov, J. Bloino, G. Zheng, J. L. Sonnenberg, M. Hada, M. Ehara, K. Toyota, R. Fukuda, J. Hasegawa, M. Ishida, T. Nakajima, Y. Honda, O. Kitao, H. Nakai, T. Vreven, J. A. Montgomery Jr, J. E. Peralta, F. Ogliaro, M. Bearpark, J. J. Heyd, E. Brothers, K. N. Kudin, V. N. Staroverov, R. Kobayashi, J. Normand, K. Raghavachari, A. Rendell, J. C. Burant, S. S. Iyengar, J. Tomasi, M. Cossi, N. Rega, J. M. Millam, M. Klene, J. E. Knox, J. B. Cross, V. Bakken, C. Adamo, J. Jaramillo, R. Gomperts, R. E. Stratmann, O. Yazyev, A. J. Austin, R. Cammi, C. Pomelli, J. W. Ochterski, R. L. Martin, K. Morokuma, V. G. Zakrzewski, G. A. Voth, P. Salvador, J. J. Dannenberg, S. Dapprich, A. D. Daniels, Ö. Farkas, J. B. Foresman, J. V. Ortiz, J. Cioslowski, D. J. Fox, Gaussian 09, Revision D.01, Gaussian, Inc., Wallingford CT, 2009.

8. S. Grimme, S. Ehrlich, L. Goerigk, J. Comput. Chem. 2011, 32, 1456.

9. F. Weigend, R. Ahlrichs, Phys. Chem. Chem. Phys., 2005, 7, 3297.

10. a) G. Scalmani, M. J. Frisch, J. Chem. Phys. 2010, 132, 114110; b) M. Cossi, N. Rega, G. Scalmani, V. Barone, J. Comput. Chem. 2003, 24, 669 and references therein.

11. Guo, F.; Dhakal, R. C.; Dieter, R. K. *J. Org. Chem.* **2013**, *78*, 8451.

12. Li, H.; Wu, J. *Org. Lett.* **2015**, *17*, 5424.

13. Guo, F.; McGilvary, M.A.; Jeffries, M.C.; Graves, B.N.; Graham, S.A.; Wu, Y. *Molecules* **2017**, *22*, 723.

14. Xu, Q.; Zhang, R.; Zhang, T.; Shi, M. *J. Org. Chem.* **2010**, *75*, 3935.

15. Rice, K. Patent WO 2012071509 A2.

4. ^{1}H, ^{19}F and $^{13}C\{1H\}$ NMR spectra

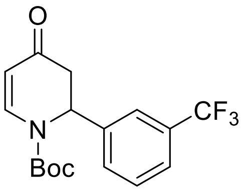

1f

^{1}H NMR spectrum ($CDCl_3$, 400 MHz):

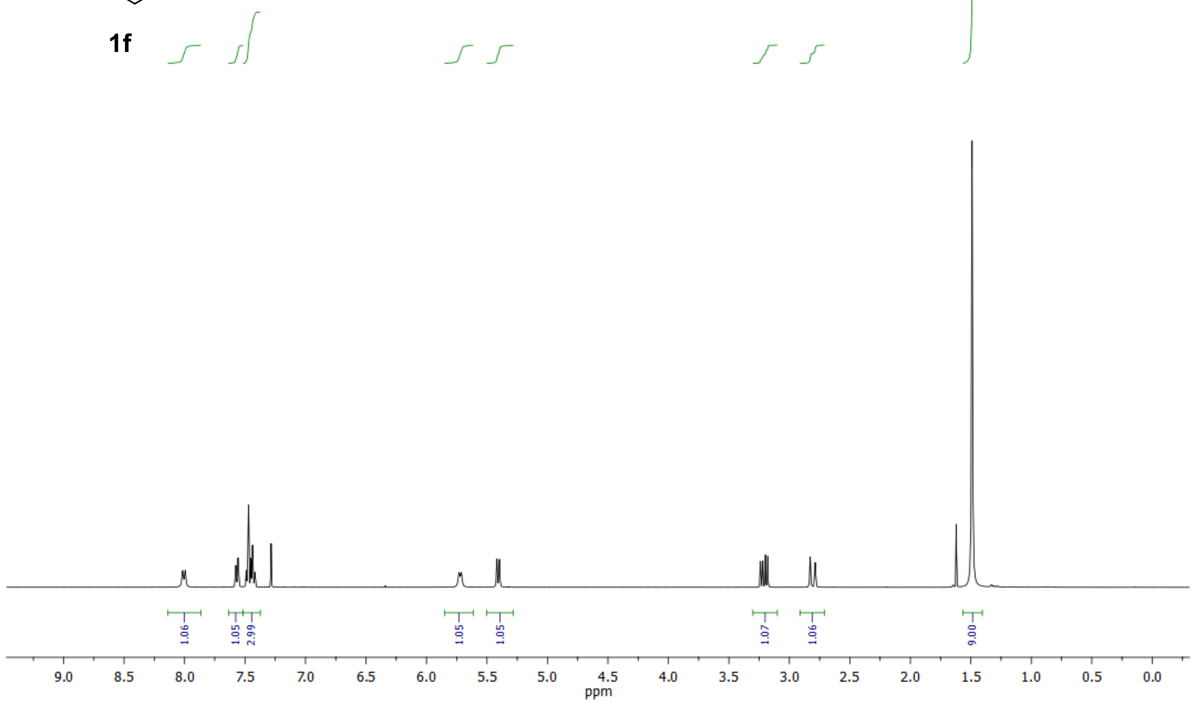

$^{13}C\{1H\}$ NMR spectrum ($CDCl_3$, 100 MHz):

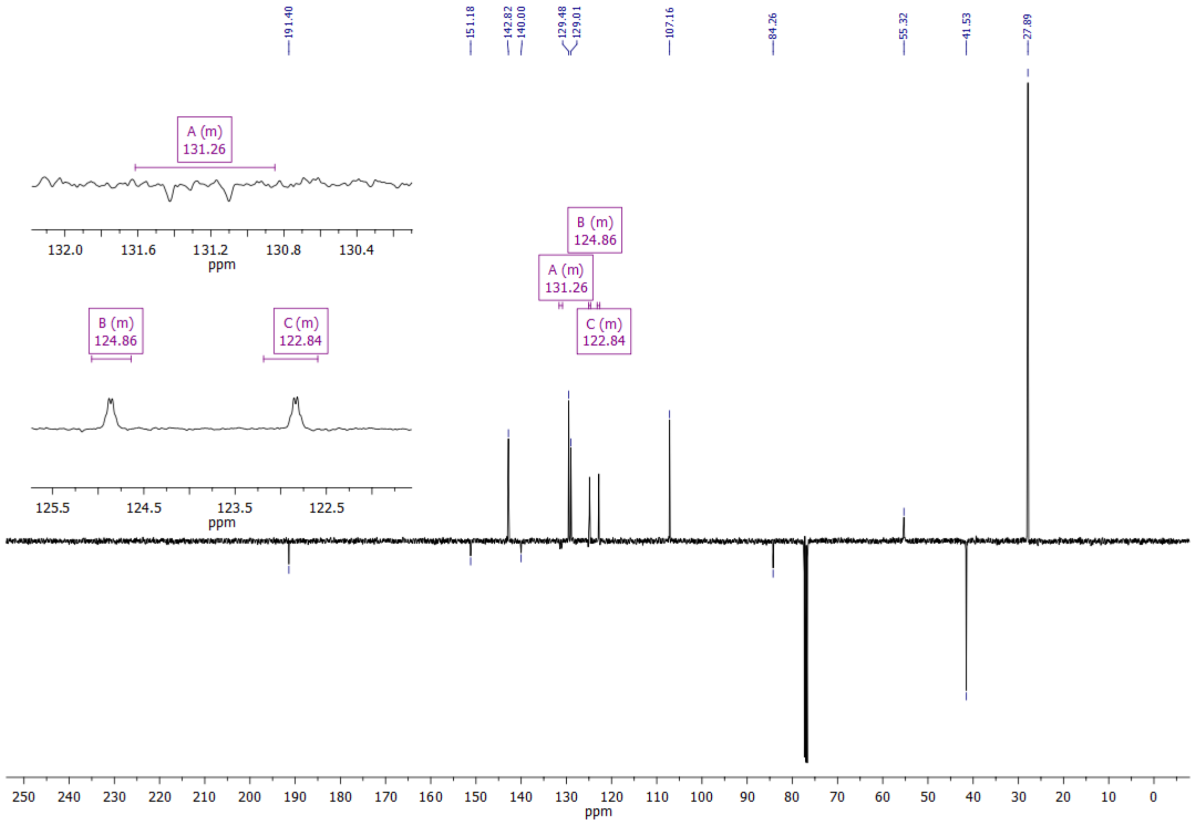

^{19}F NMR spectrum ($CDCl_3$, 377 MHz):

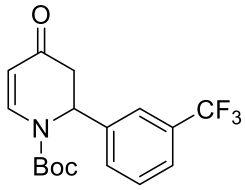

1f (^{19}F-NMR)

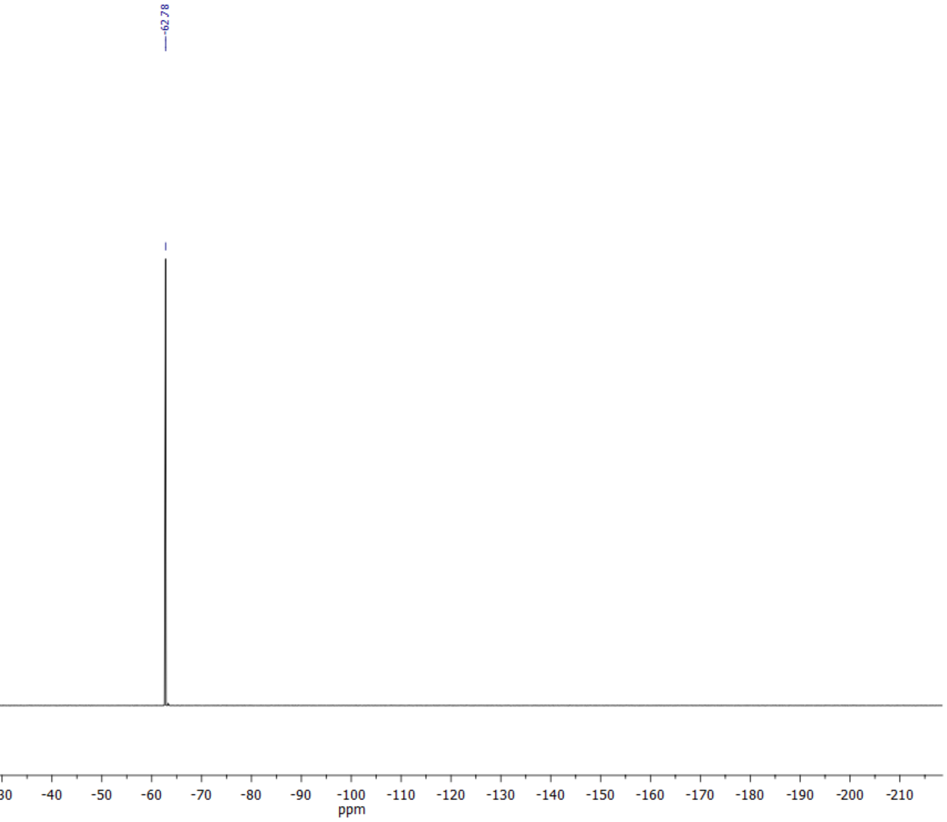

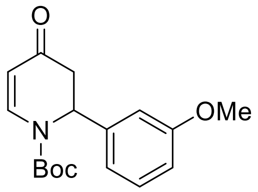

1g

^{1}H NMR spectrum ($CDCl_3$, 400 MHz):

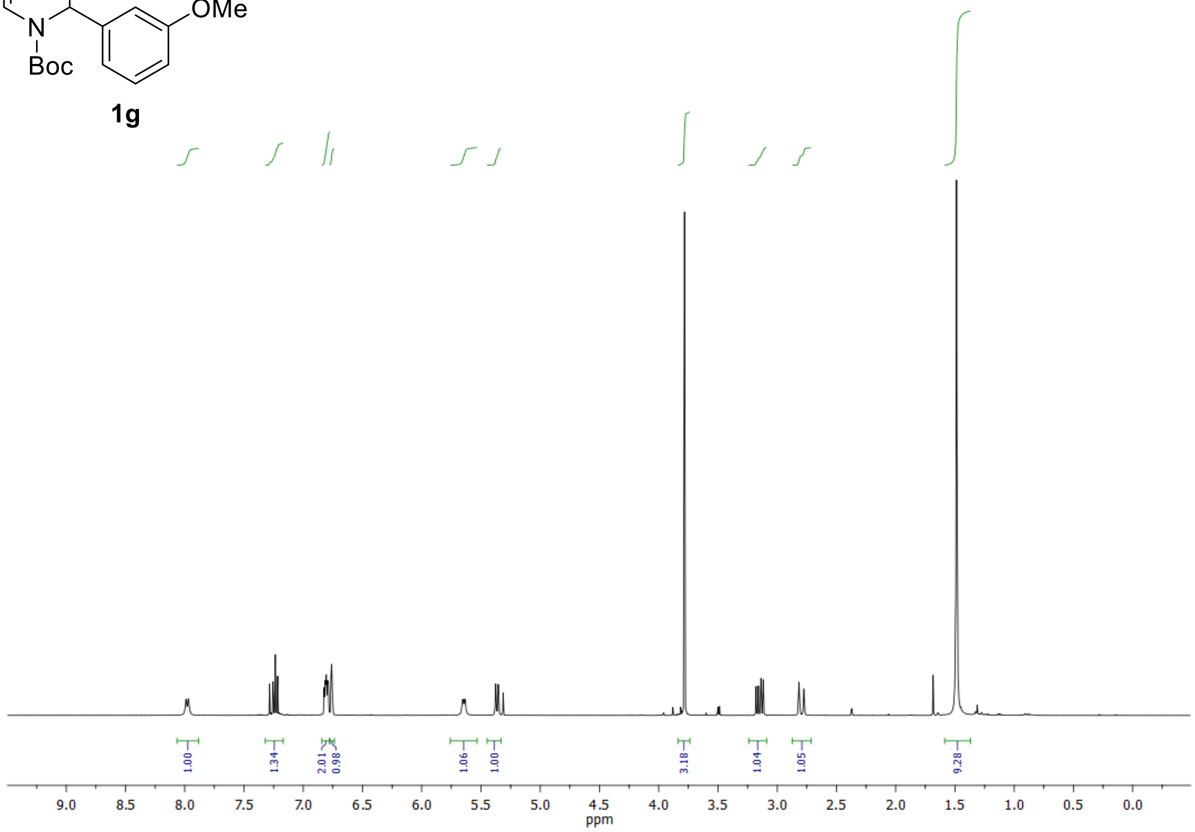

^{13}C{1H} NMR spectrum ($CDCl_3$, 100 MHz):

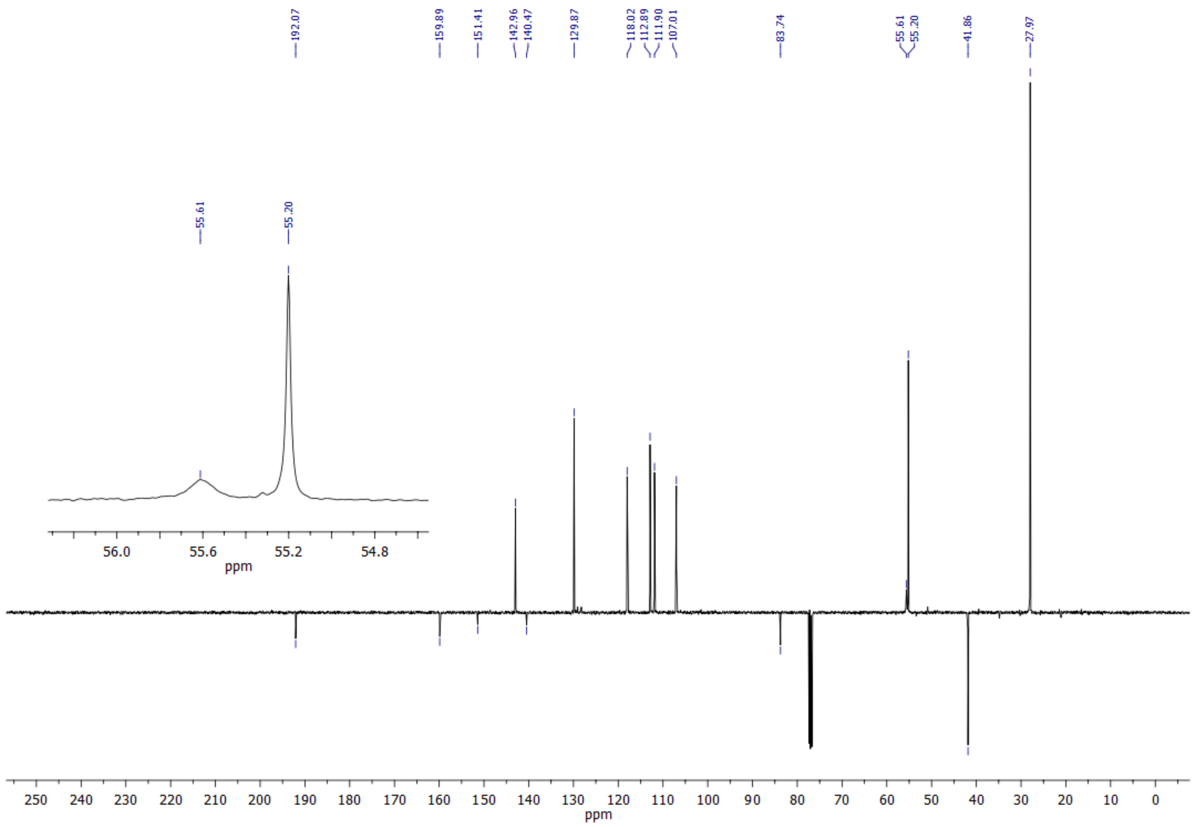

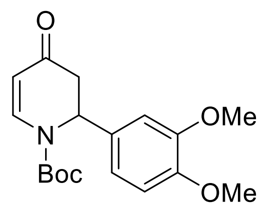

1h

^{1}H NMR spectrum ($CDCl_3$, 400 MHz):

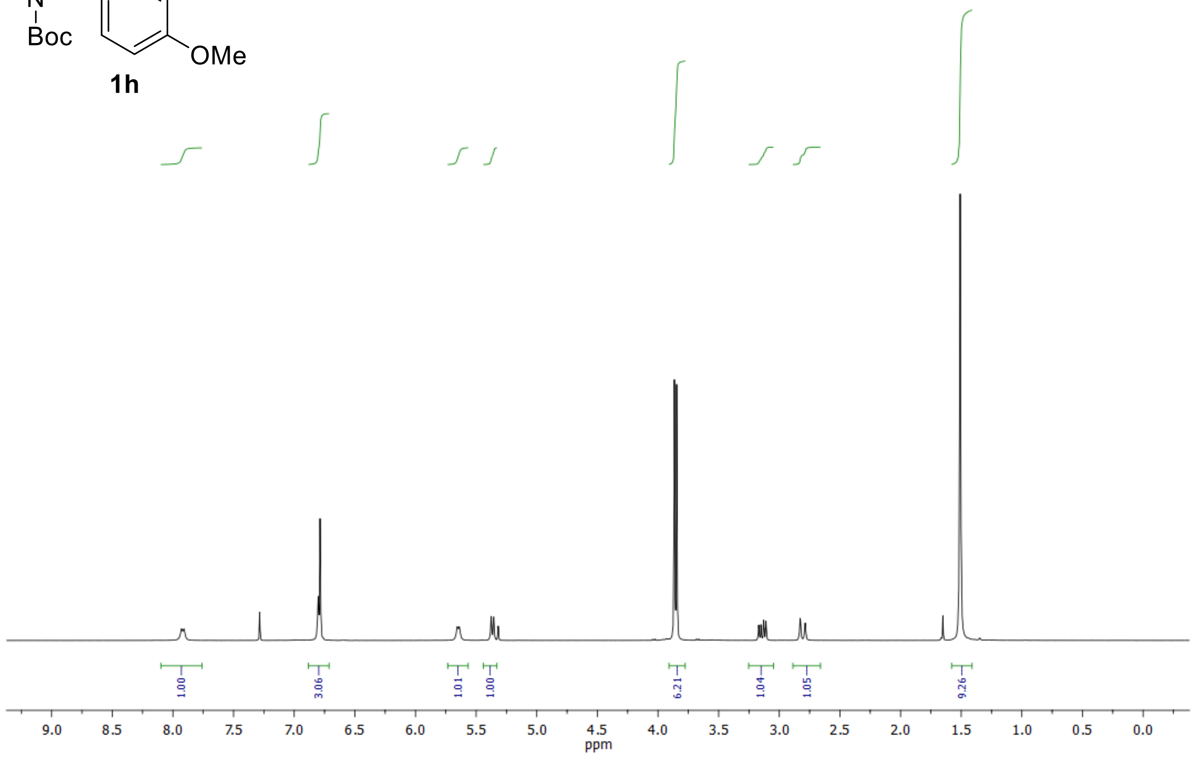

$^{13}C\{1H\}$ NMR spectrum ($CDCl_3$, 100 MHz):

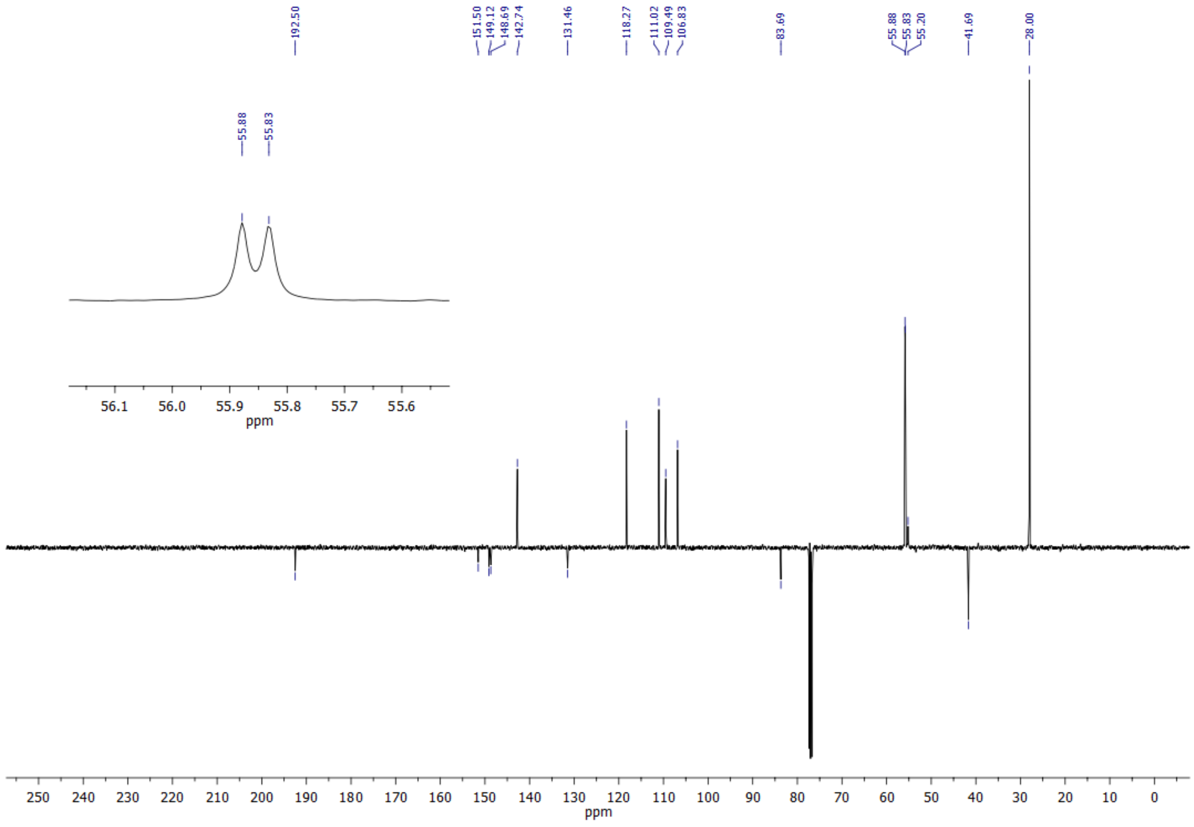

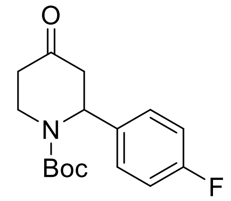

2b

^{1}H NMR spectrum (CDCl3, 400 MHz):

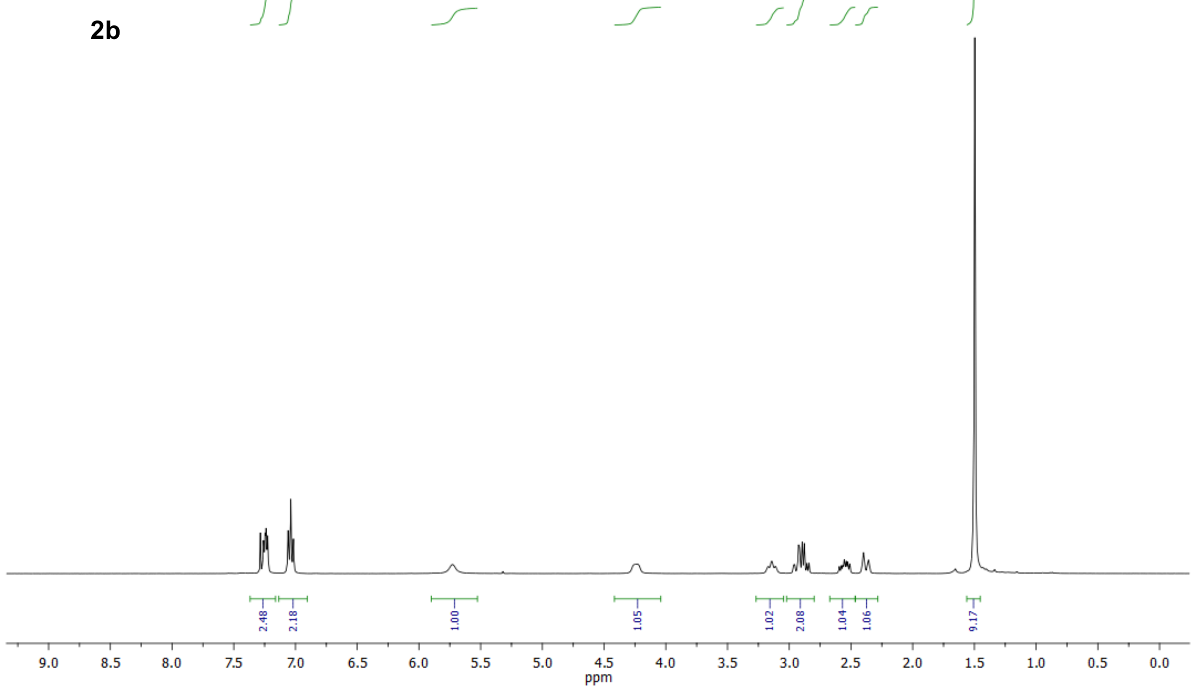

^{13}C{1H} NMR spectrum (CDCl3, 100 MHz):

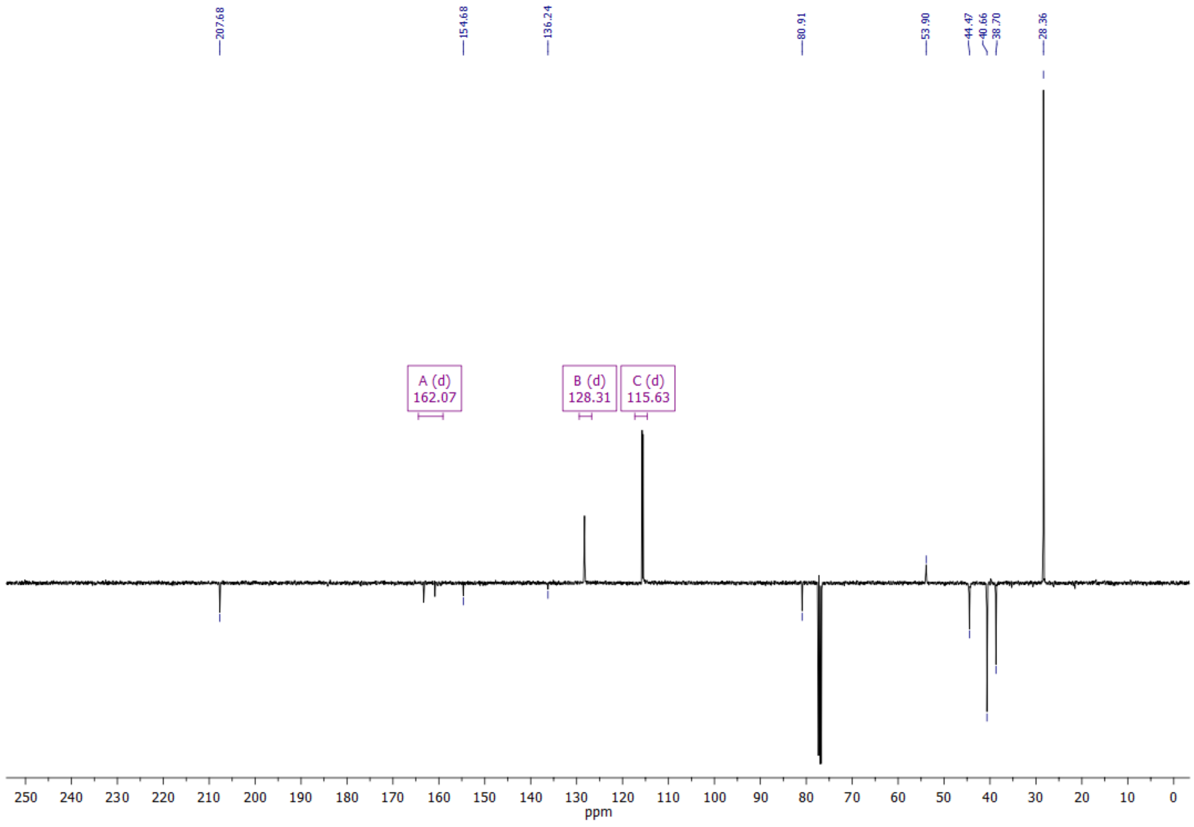

^{19}F NMR spectrum ($CDCl_3$, 377 MHz):

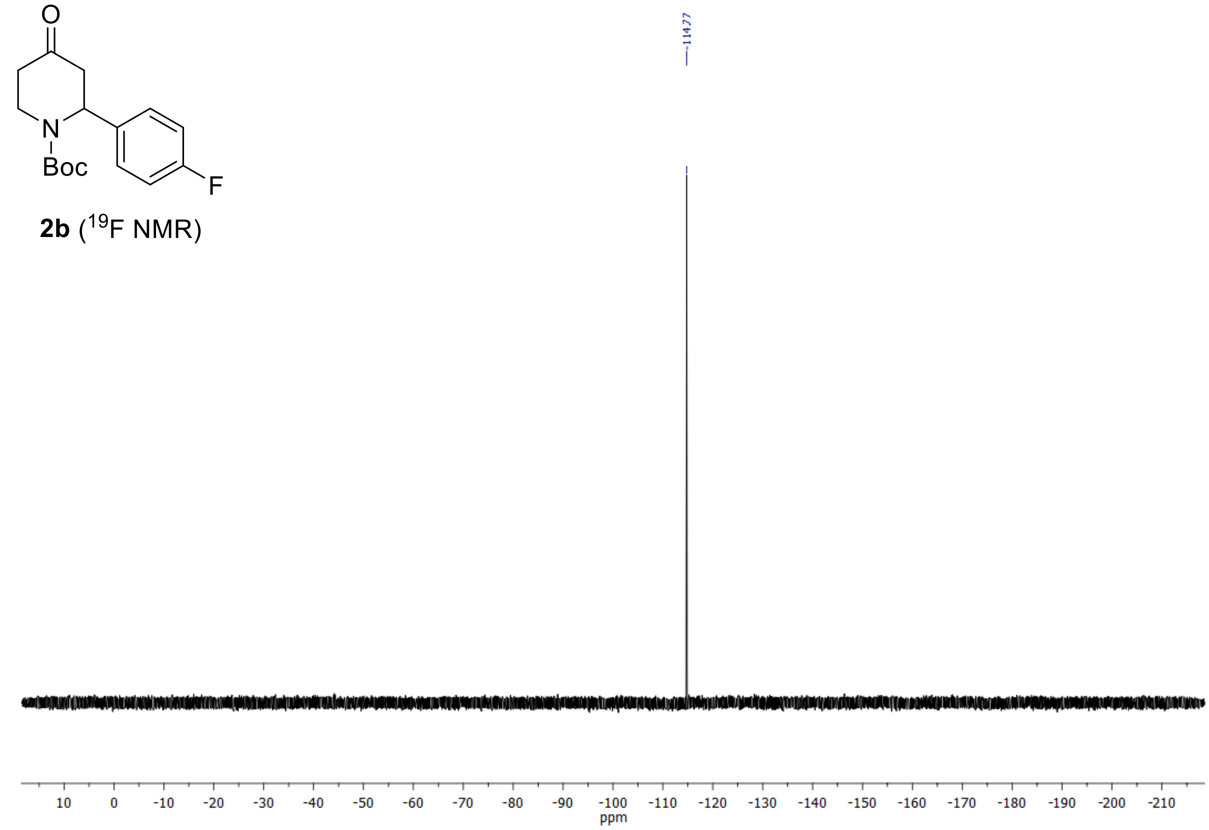

2b (^{19}F NMR)

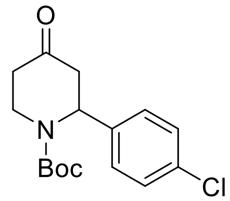

2c

^{1}H NMR spectrum ($CDCl_3$, 400 MHz):

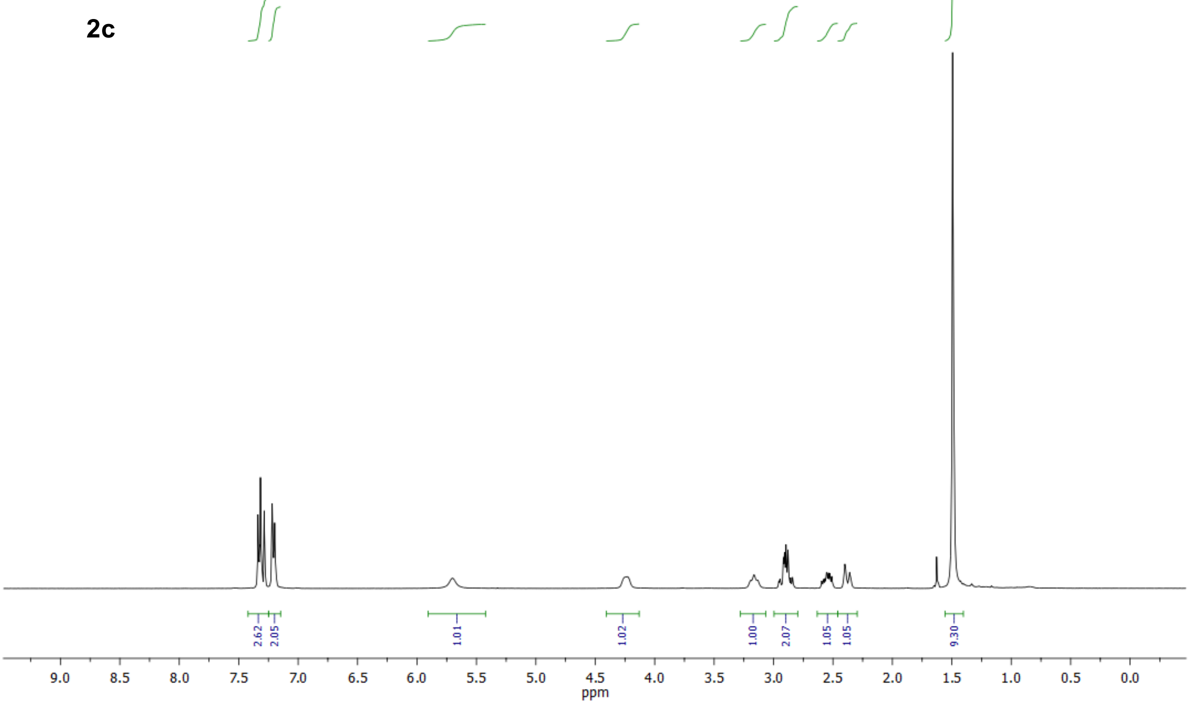

$^{13}C\{1H\}$ NMR spectrum ($CDCl_3$, 100 MHz):

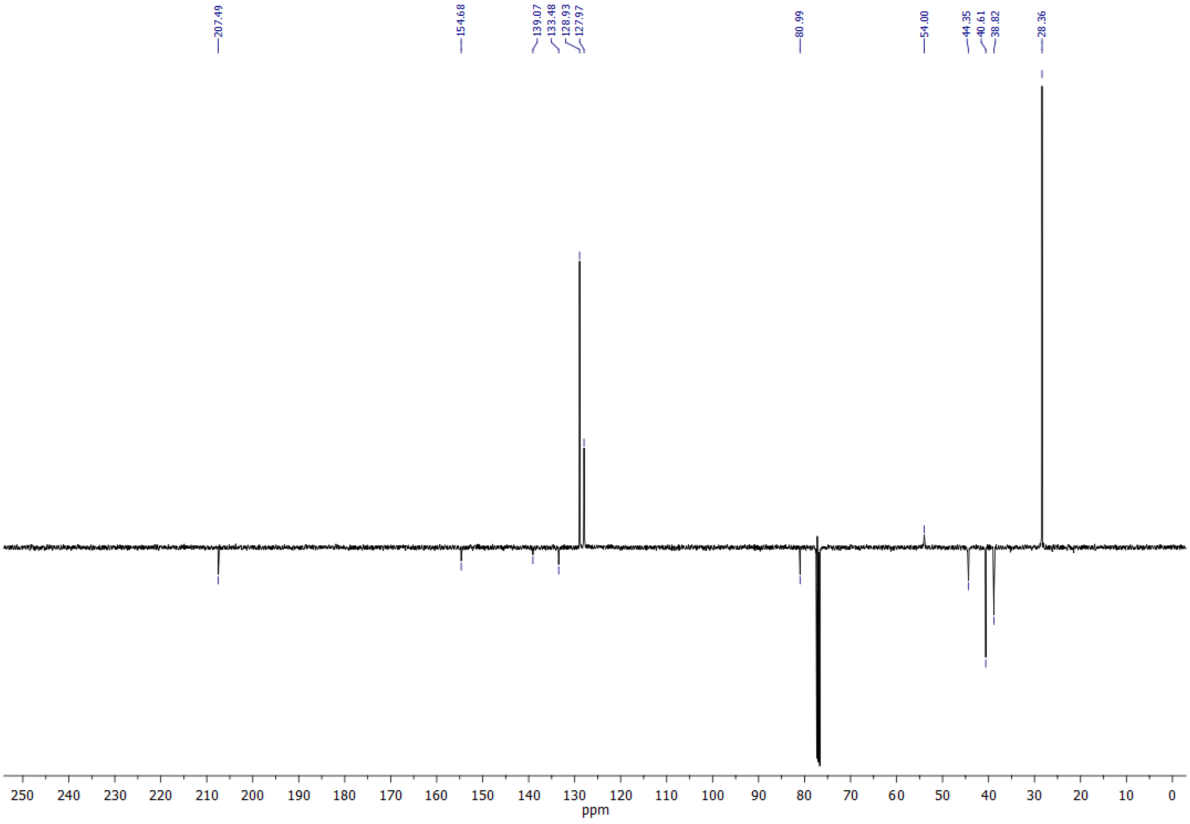

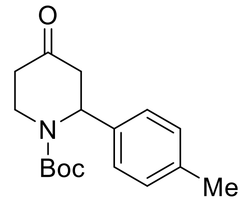

2d

^{1}H NMR spectrum ($CDCl_3$, 400 MHz):

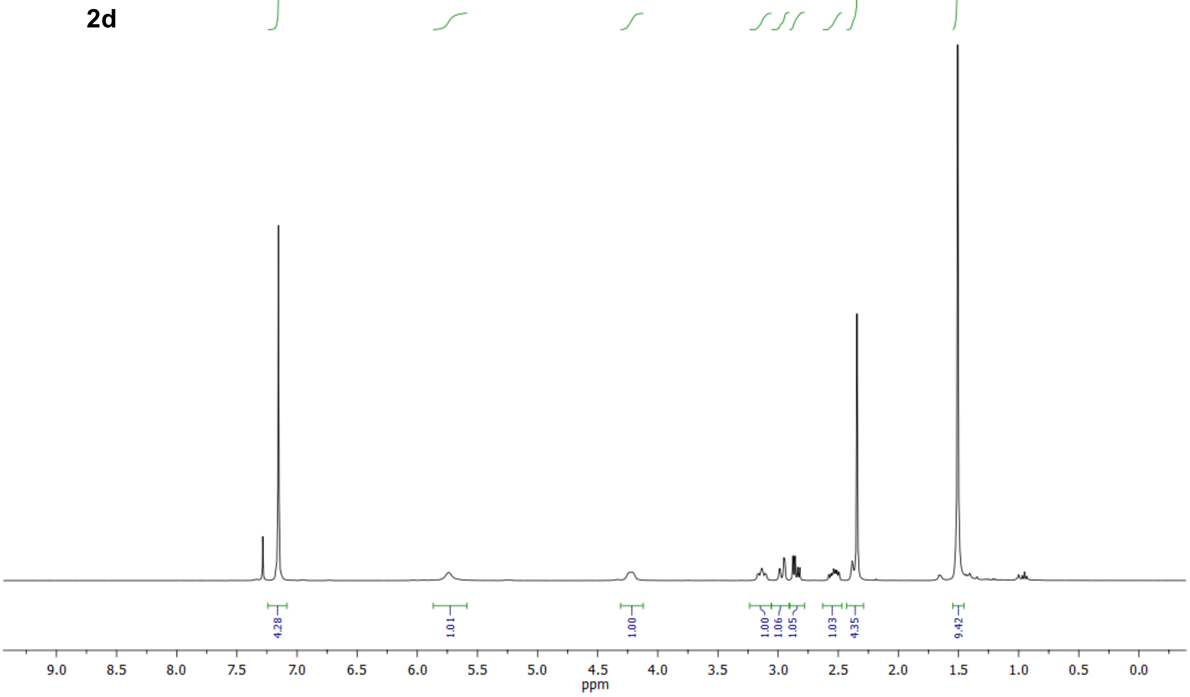

^{13}C{1H} NMR spectrum ($CDCl_3$, 100 MHz):

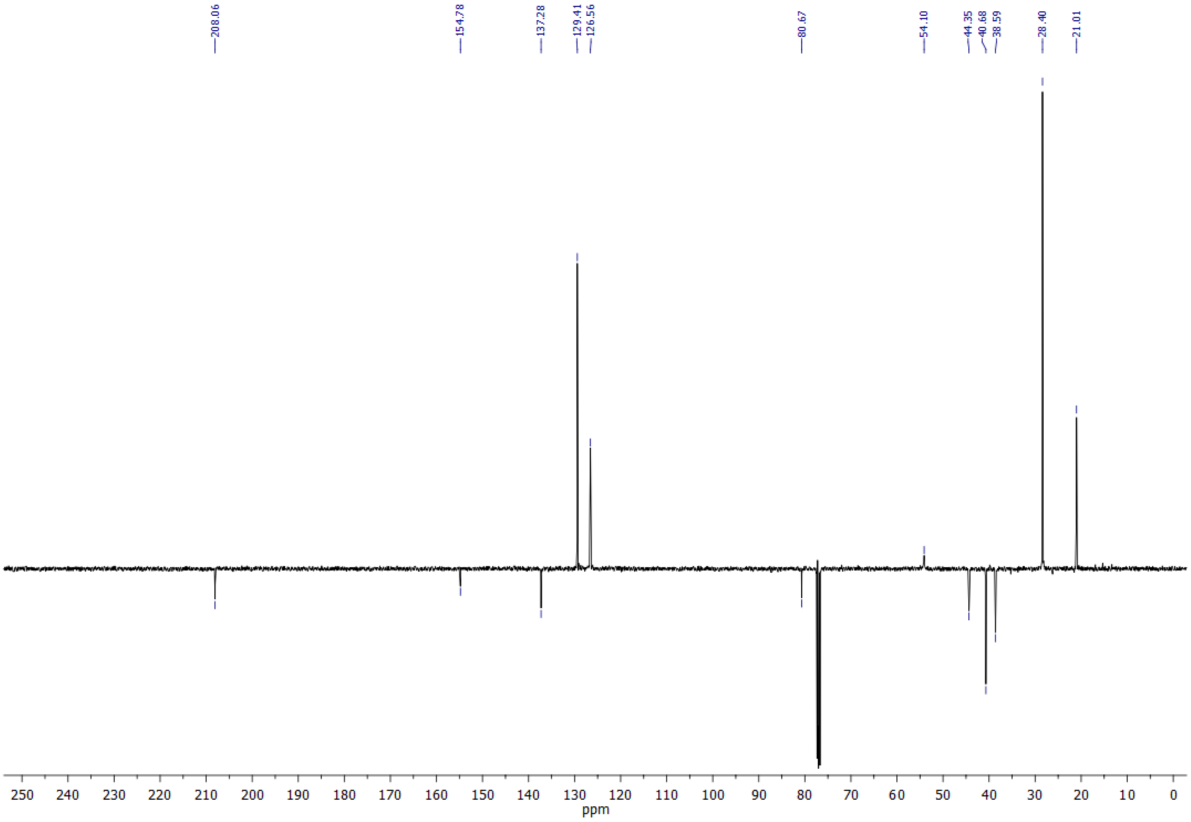

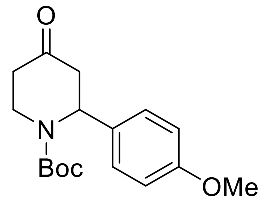

2e

^{1}H NMR spectrum ($CDCl_3$, 400 MHz):

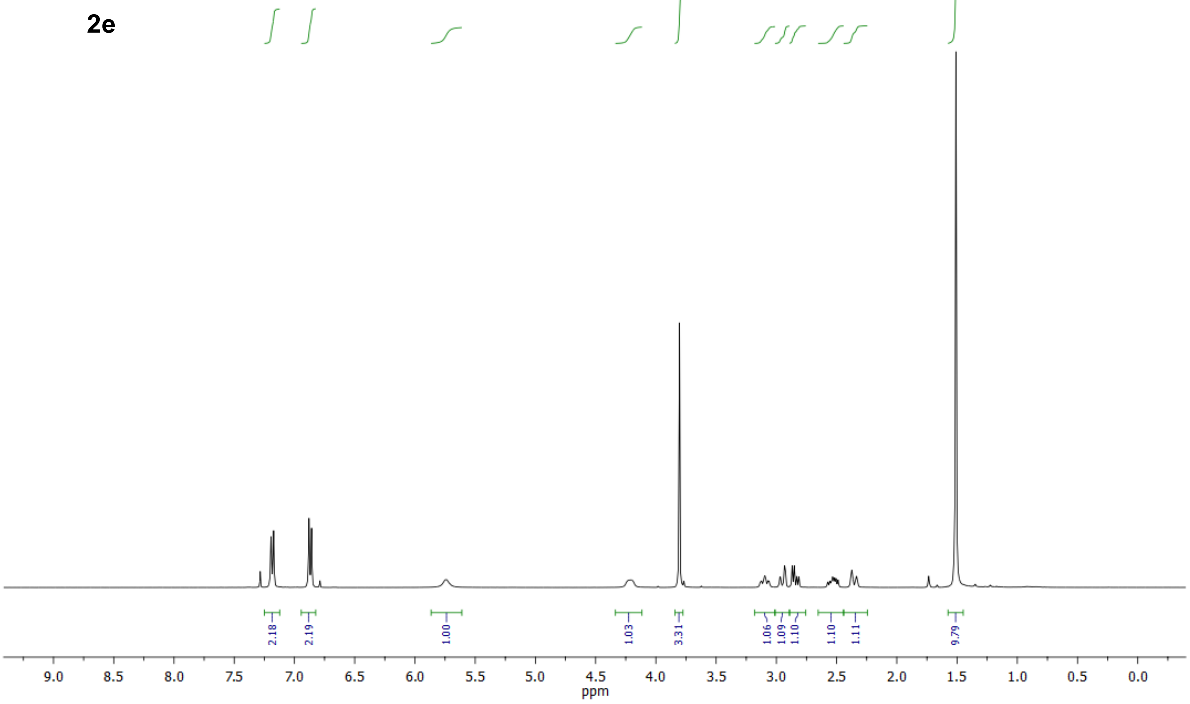

^{13}C{1H} NMR spectrum ($CDCl_3$, 100 MHz):

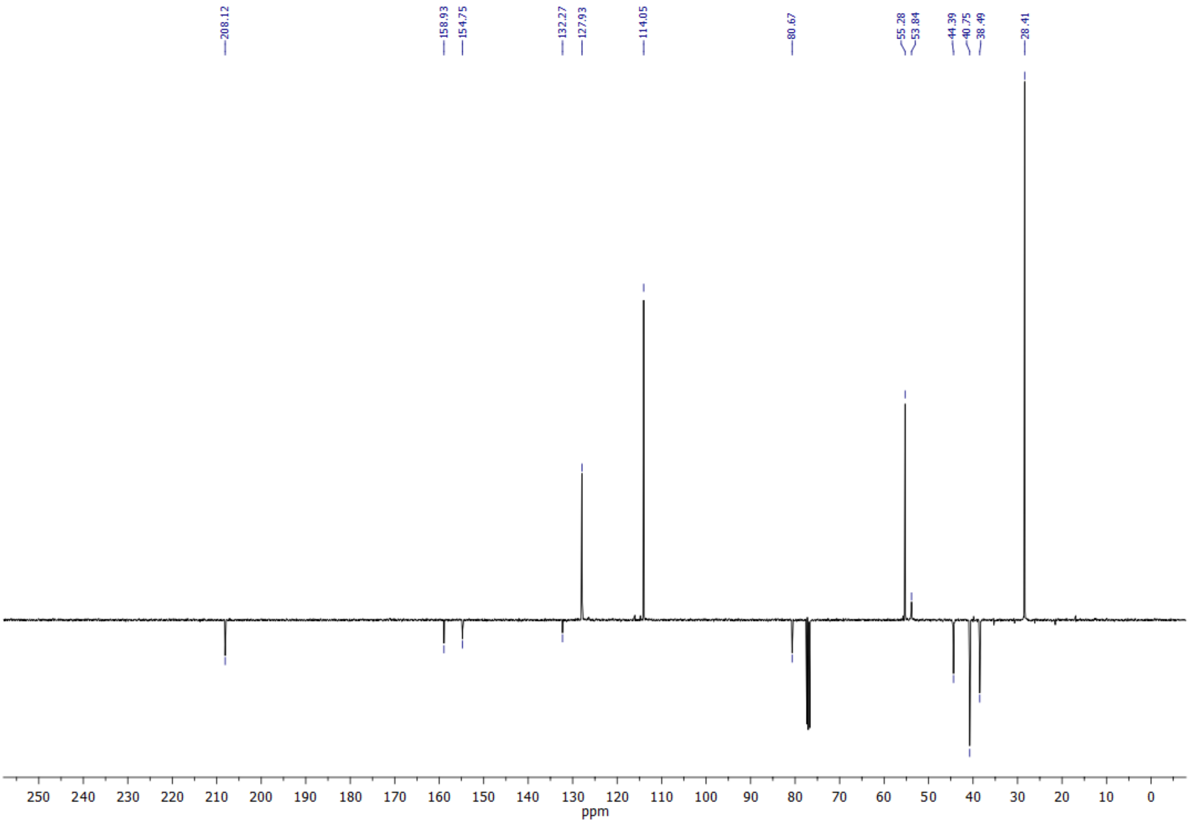

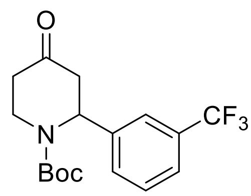

2f

^{1}H NMR spectrum (CDCl$_3$, 400 MHz):

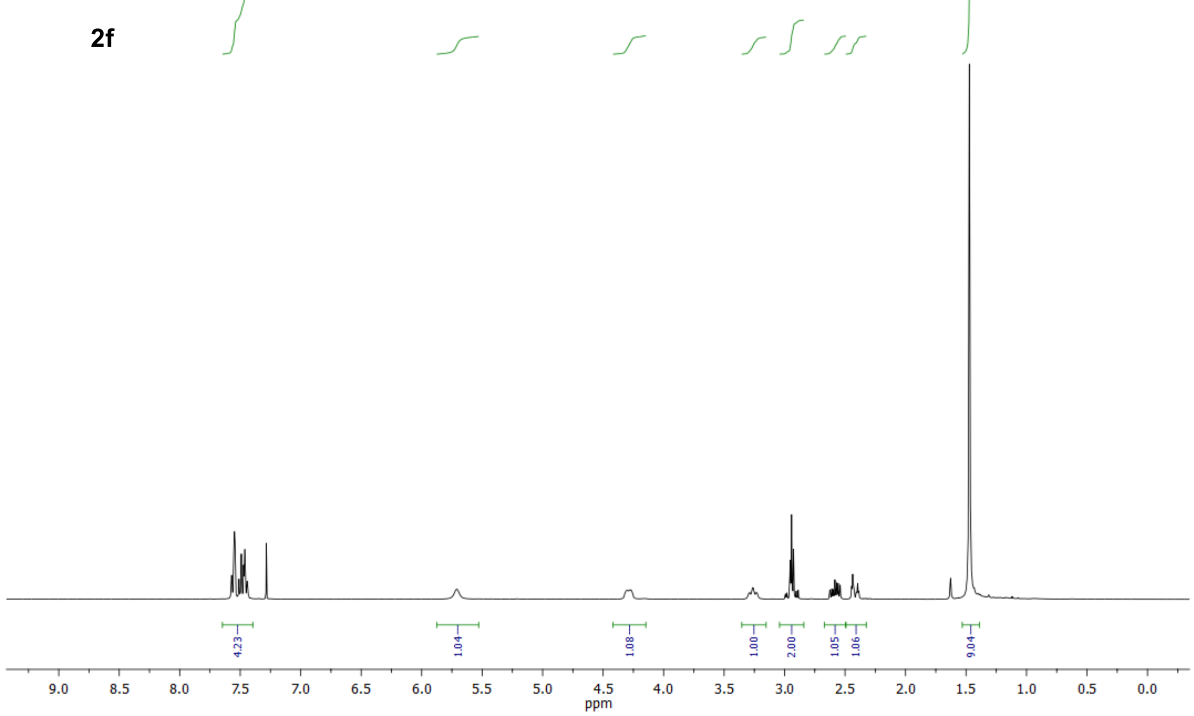

^{13}C{1H} NMR spectrum (CDCl$_3$, 100 MHz):

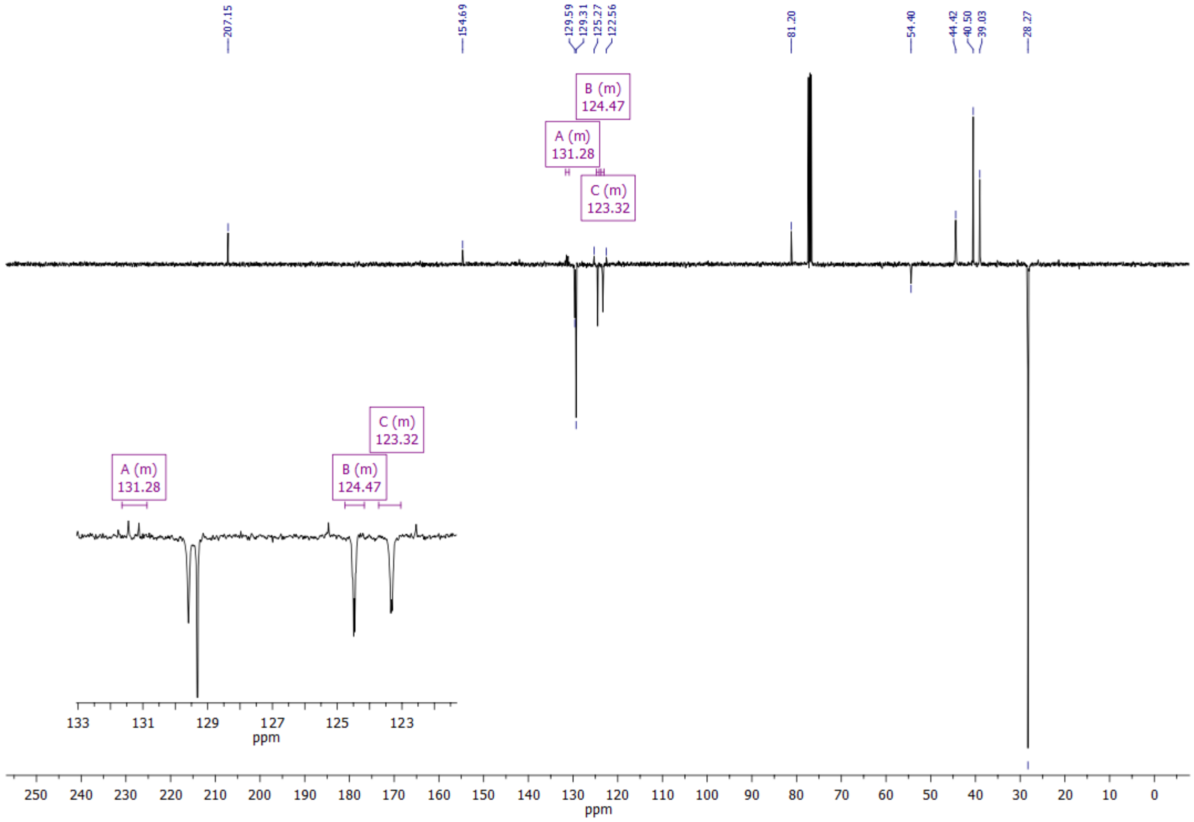

^{19}F NMR spectrum ($CDCl_3$, 377 MHz):

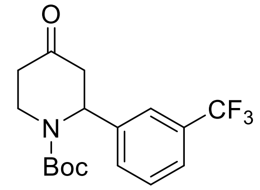

2f (^{19}F-NMR)

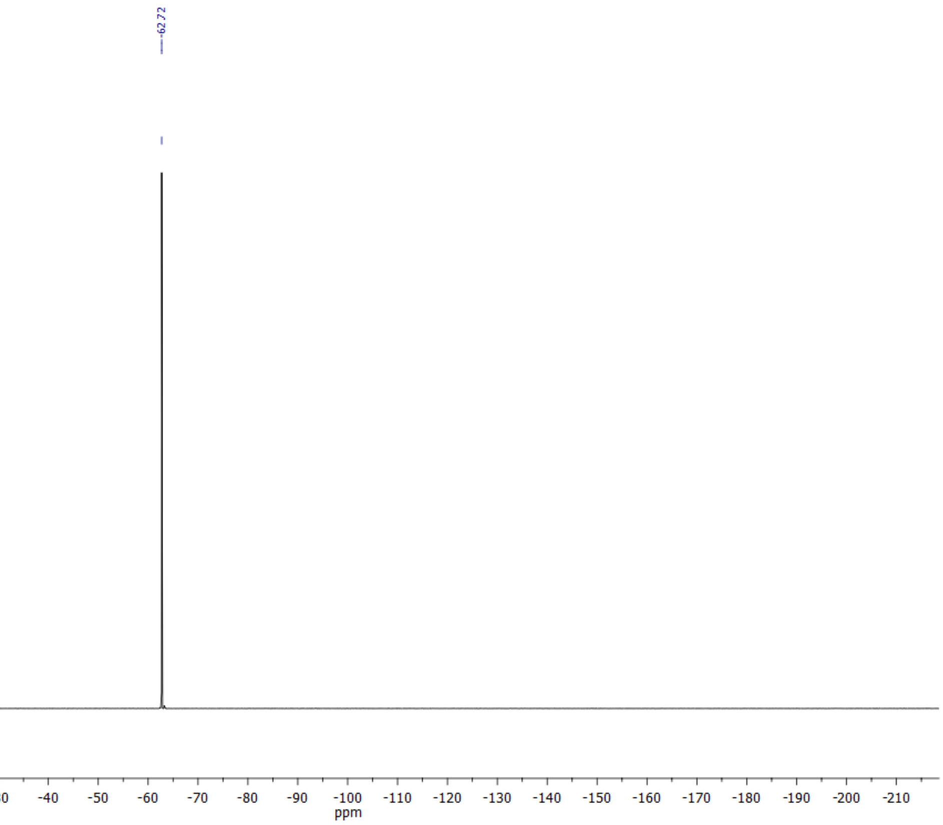

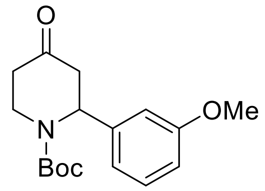

2g

^{1}H NMR spectrum ($CDCl_3$, 400 MHz):

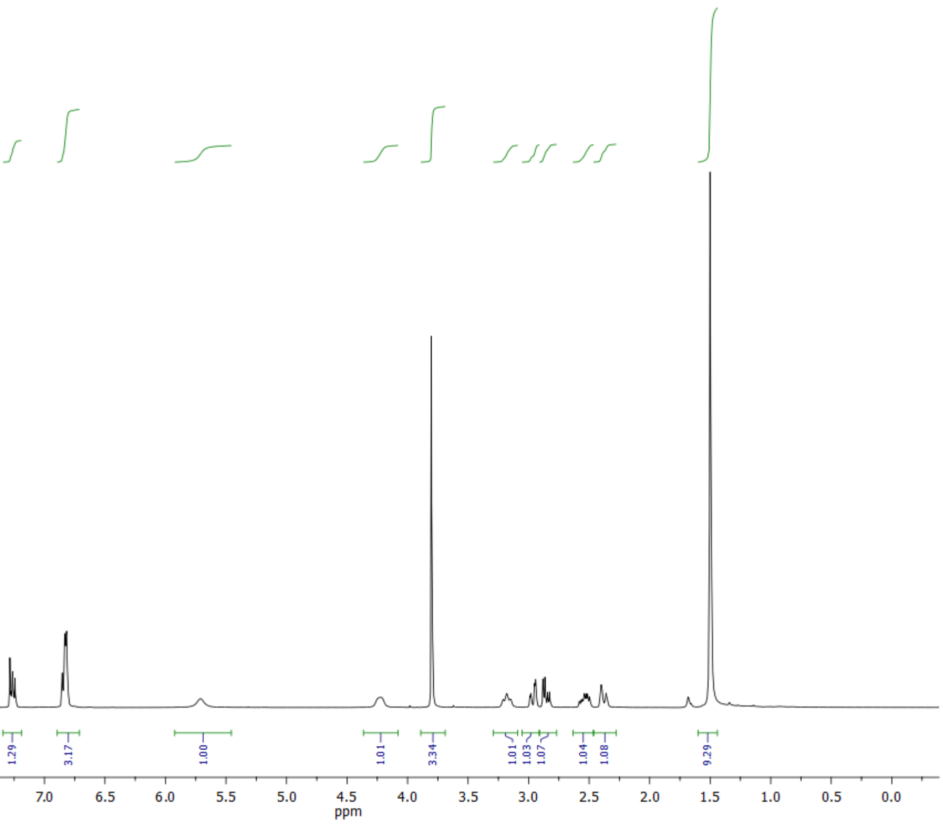

^{13}C{1H} NMR spectrum ($CDCl_3$, 100 MHz):

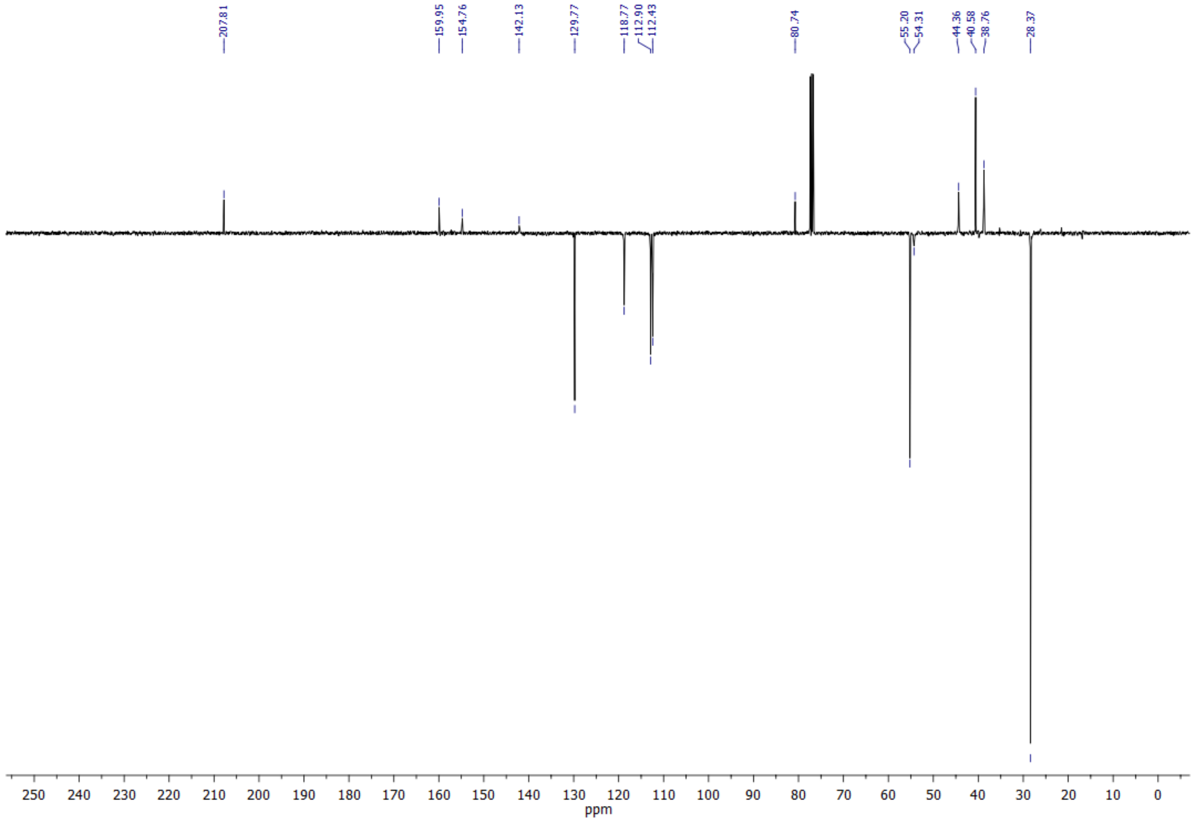

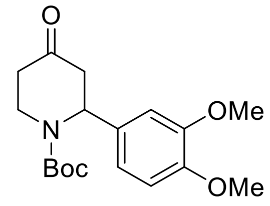

2h

^{1}H NMR spectrum ($CDCl_3$, 400 MHz):

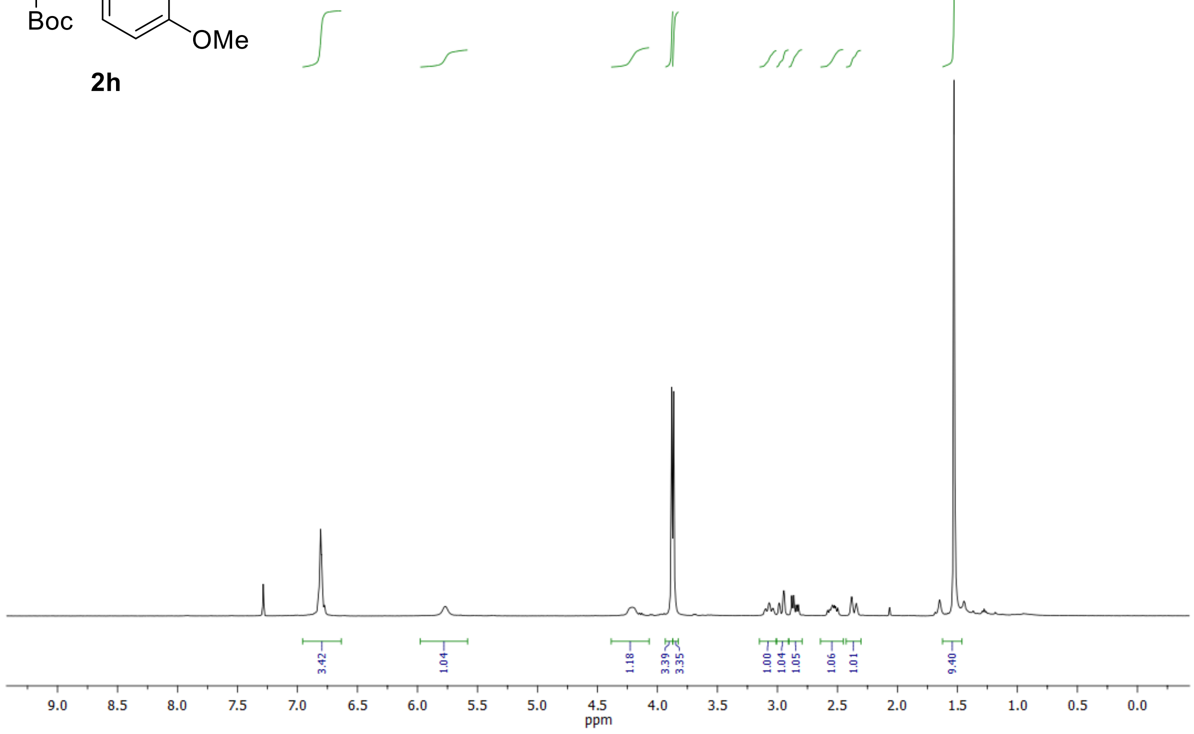

^{13}C{1H} NMR spectrum ($CDCl_3$, 100 MHz):

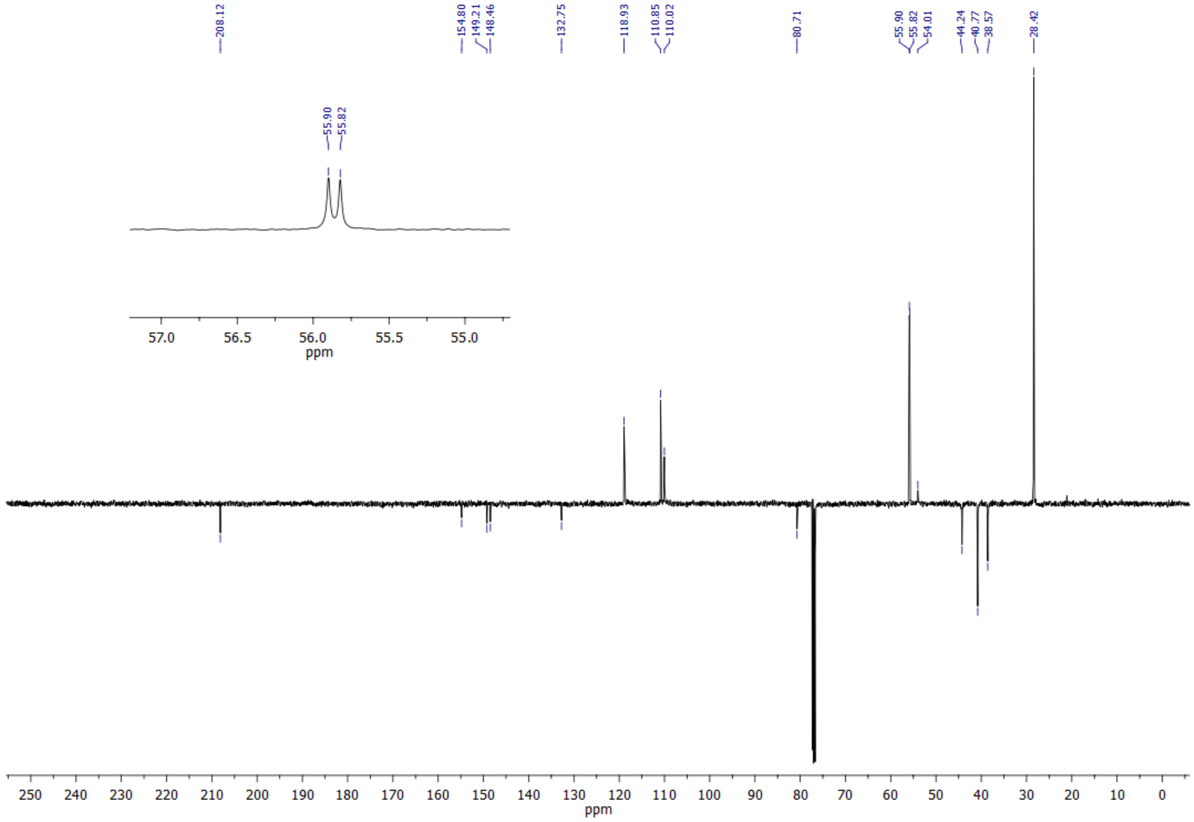

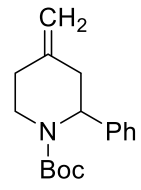

3a

^{1}H NMR spectrum ($CDCl_3$, 400 MHz):

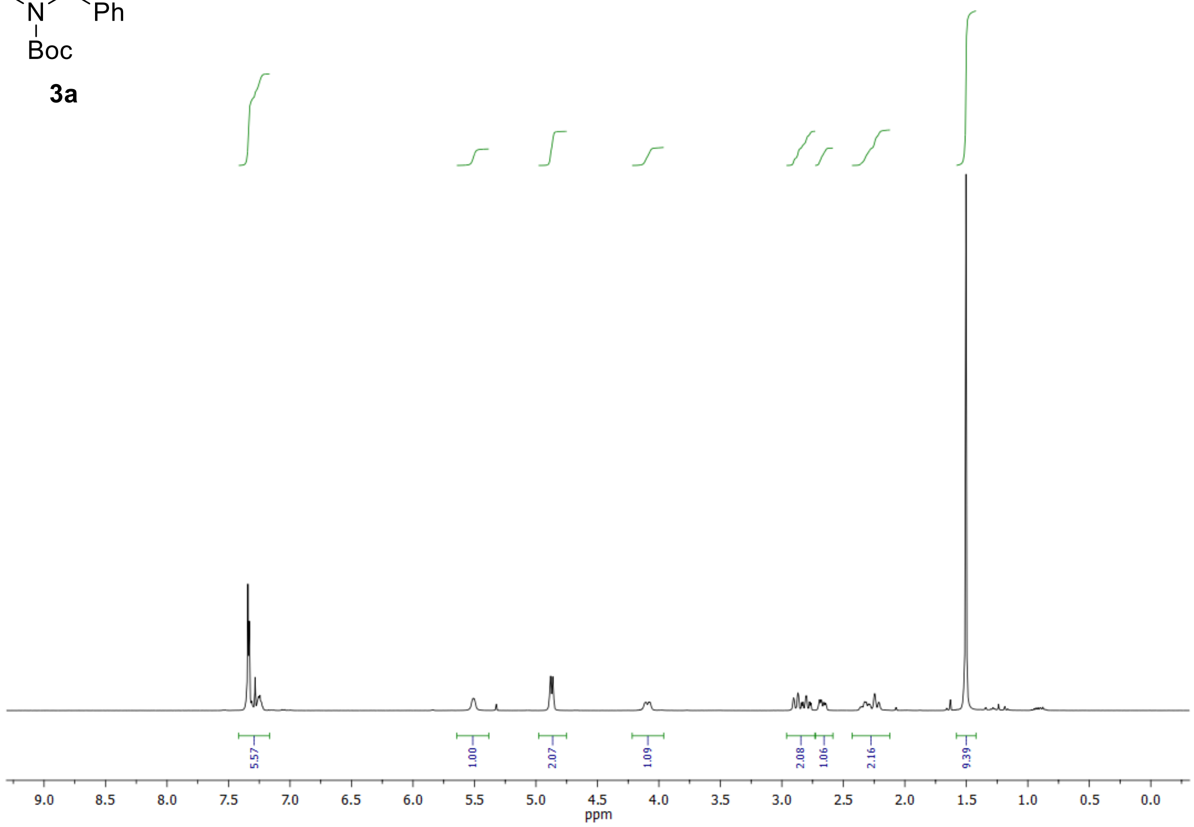

^{13}C{1H} NMR spectrum ($CDCl_3$, 100 MHz):

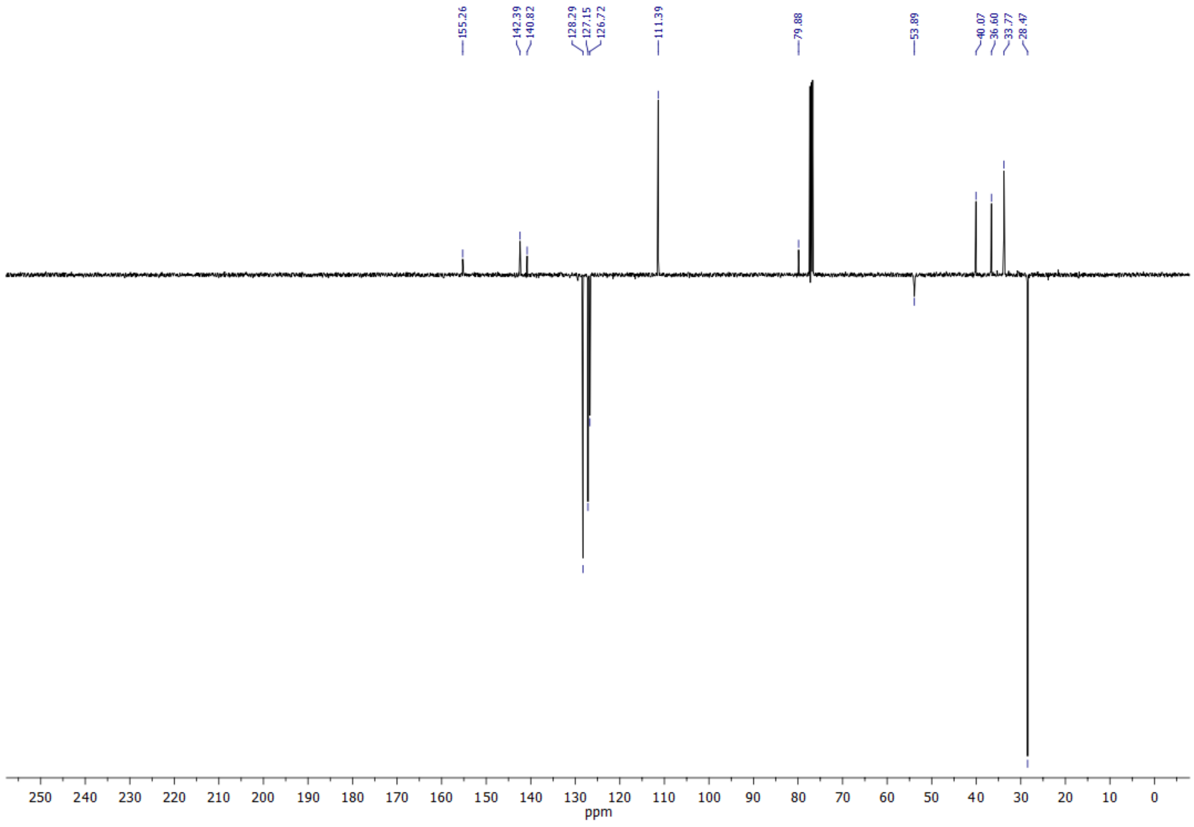

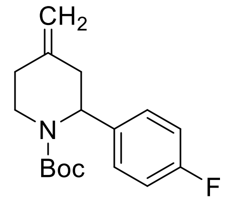

3b

^{1}H NMR spectrum (CDCl3, 400 MHz):

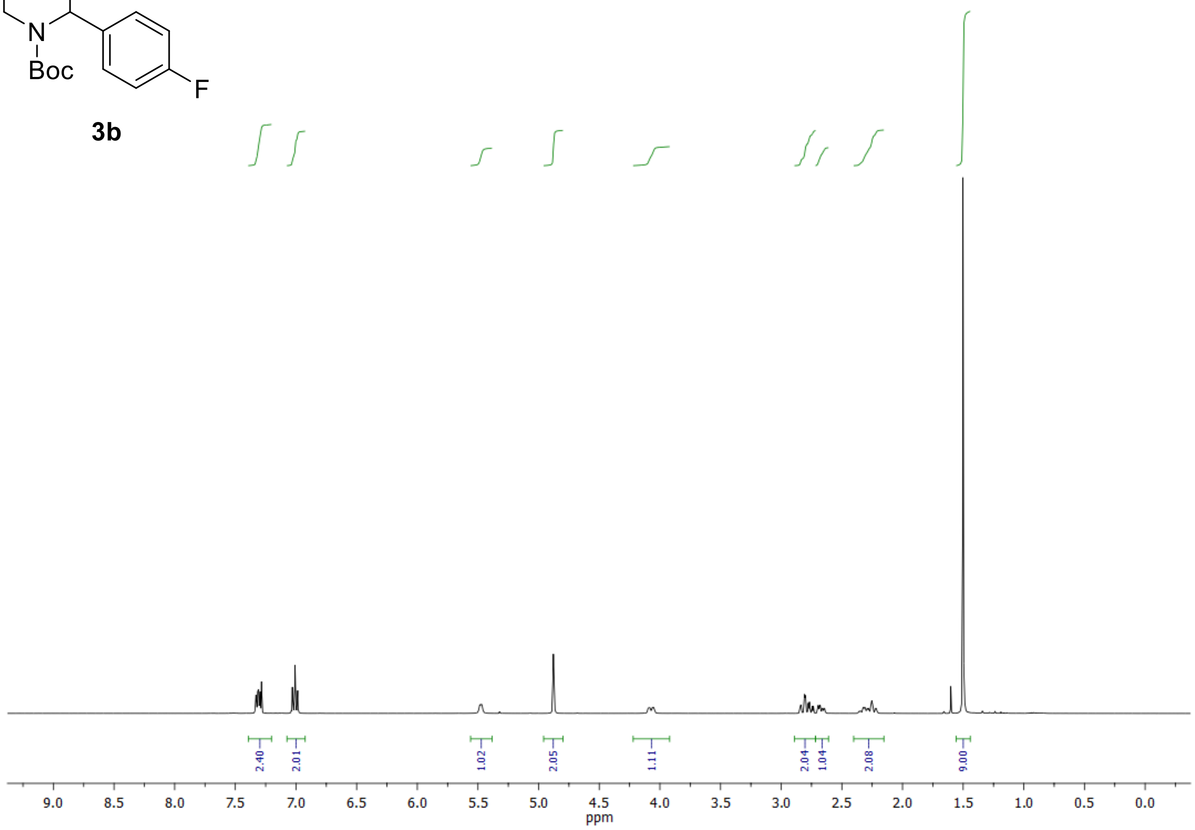

^{13}C{1H} NMR spectrum (CDCl3, 100 MHz):

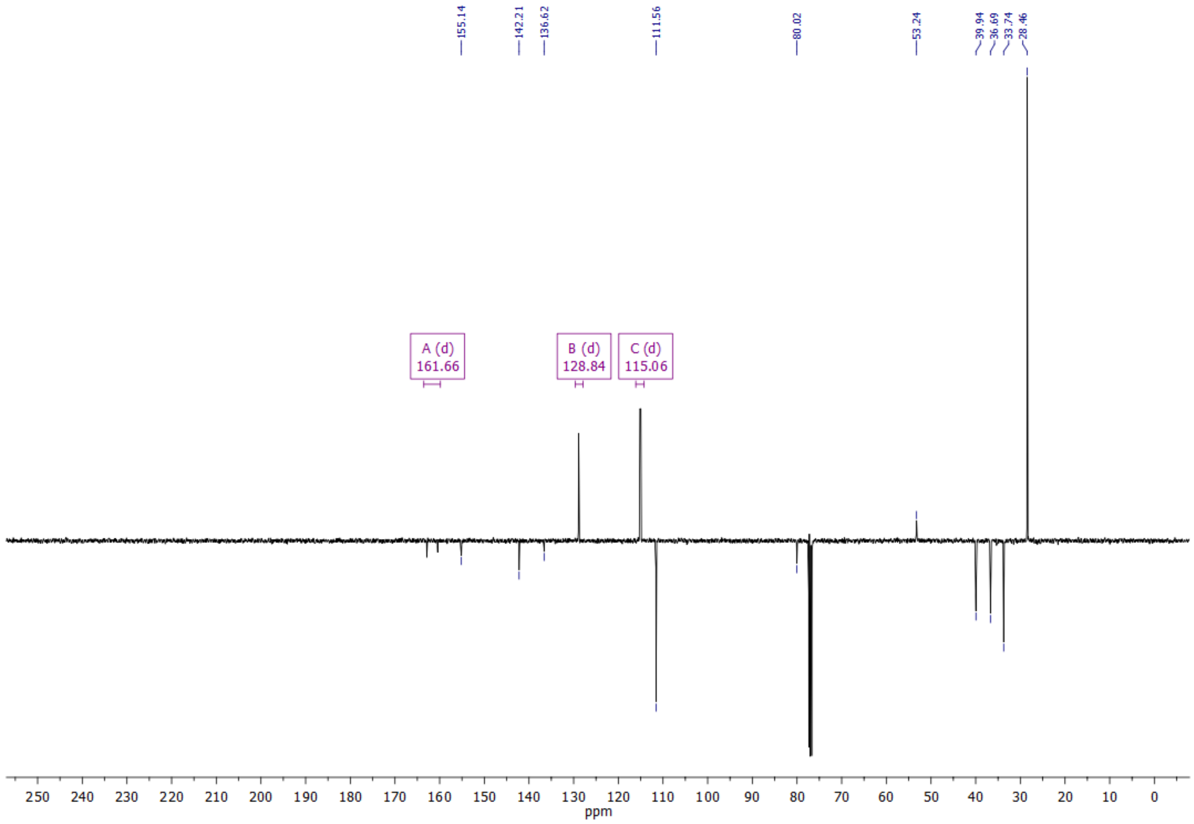

^{19}F NMR spectrum ($CDCl_3$, 377 MHz):

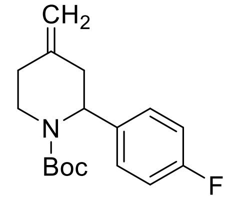

3b (^{19}F-NMR)

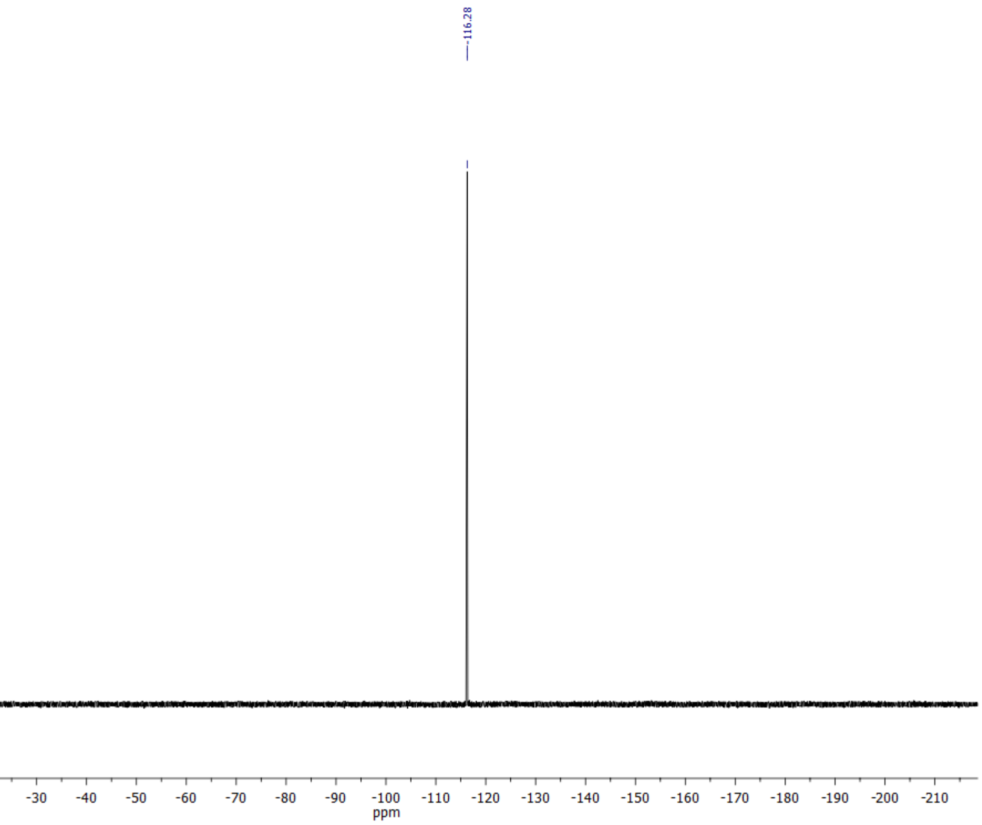

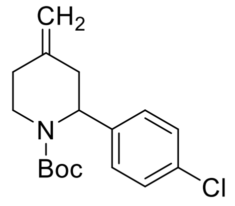

3c

^{1}H NMR spectrum ($CDCl_3$, 400 MHz):

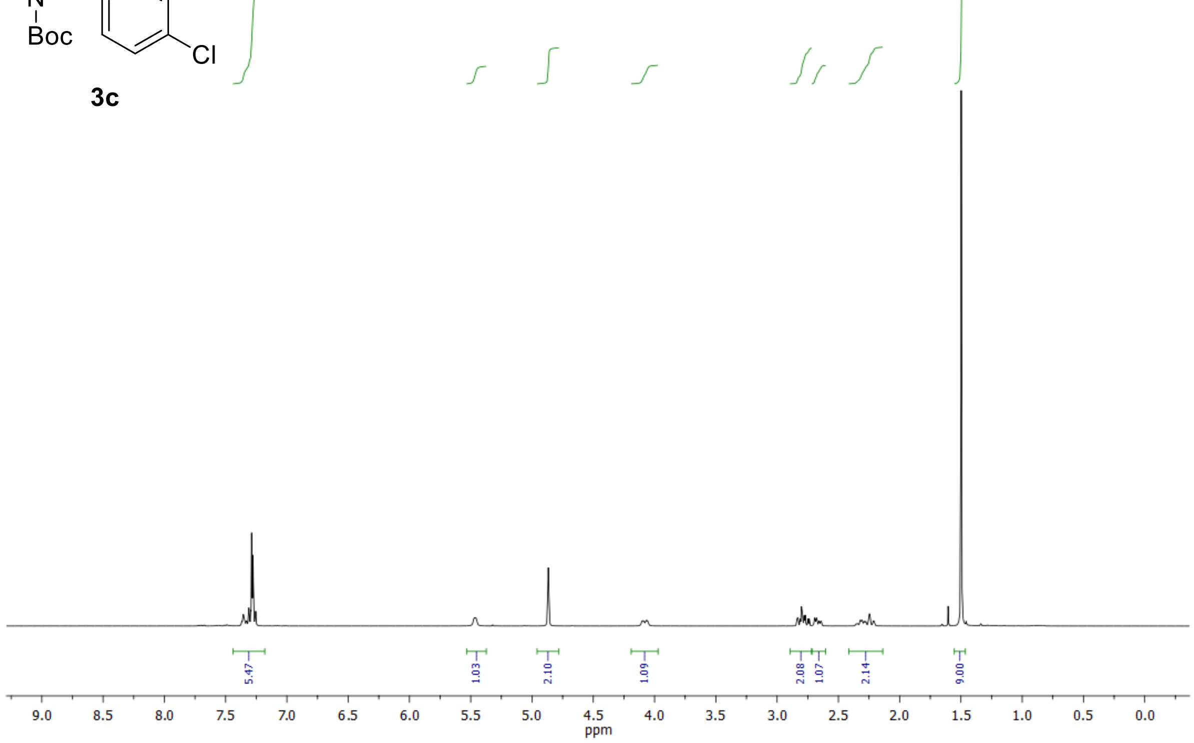

^{13}C{1H} NMR spectrum ($CDCl_3$, 100 MHz):

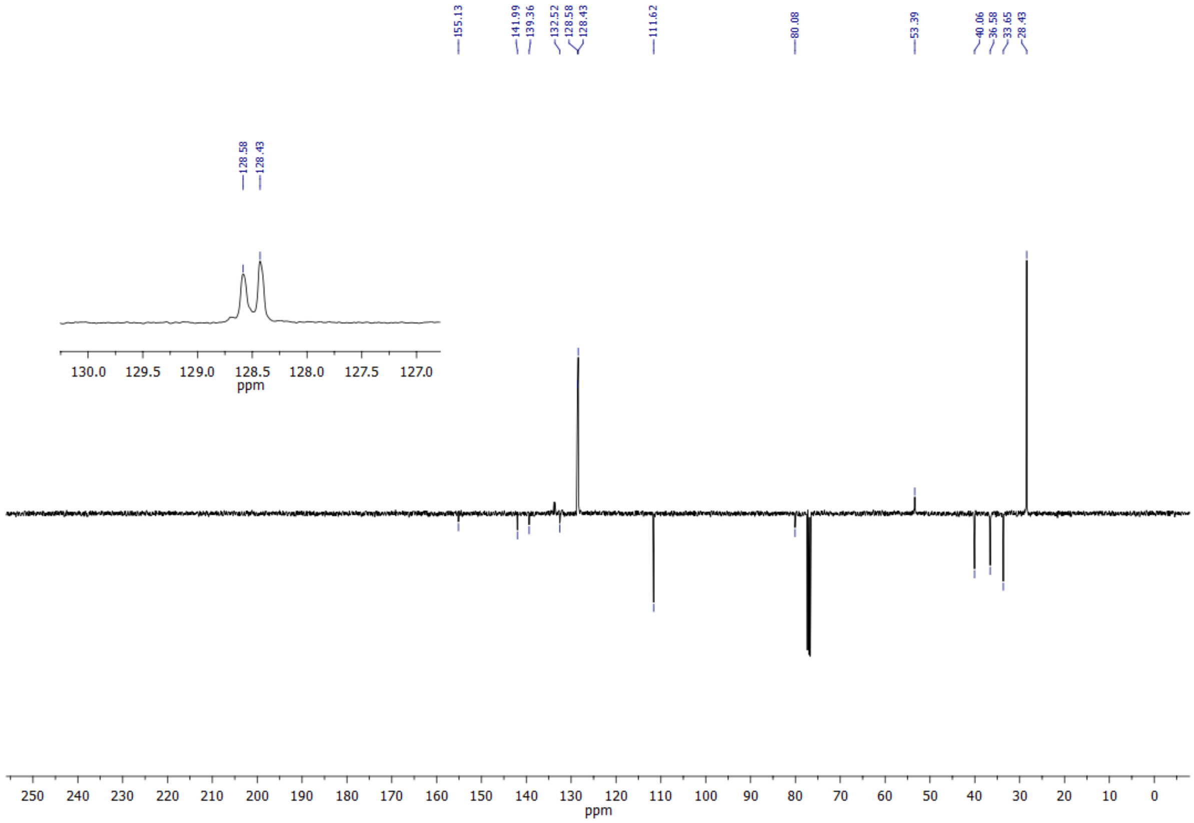

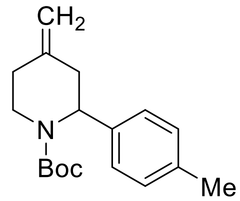

3d

^{1}H NMR spectrum ($CDCl_3$, 400 MHz):

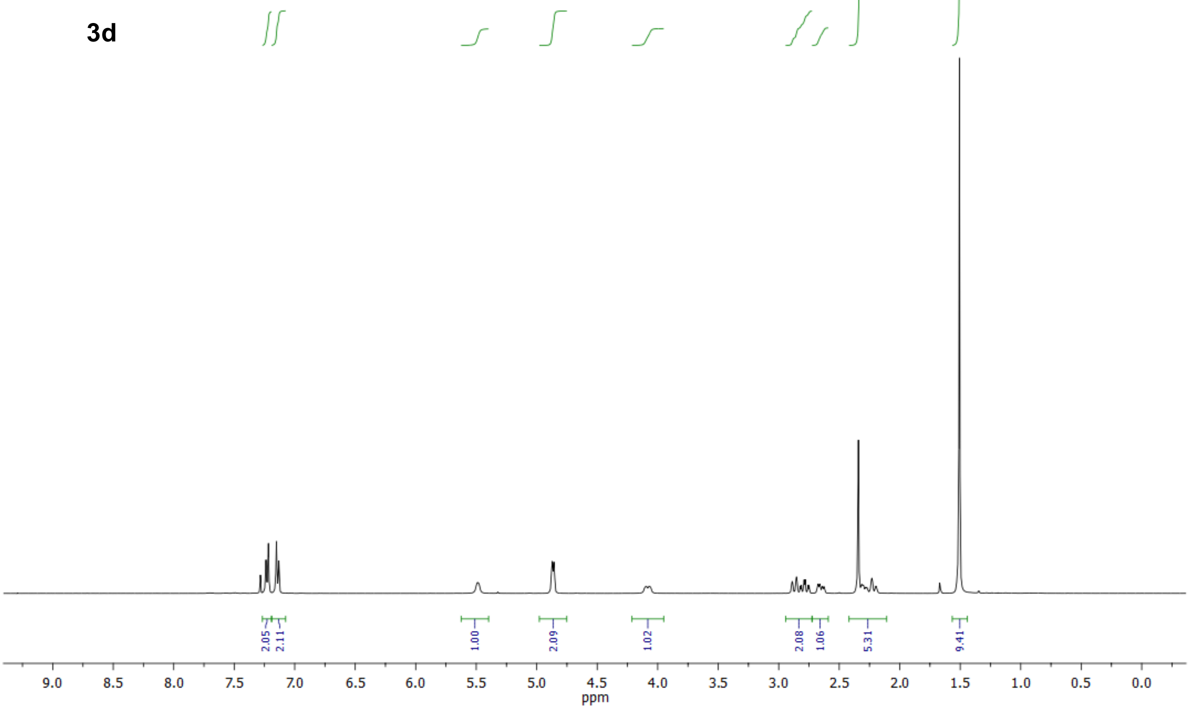

^{13}C{1H} NMR spectrum ($CDCl_3$, 100 MHz):

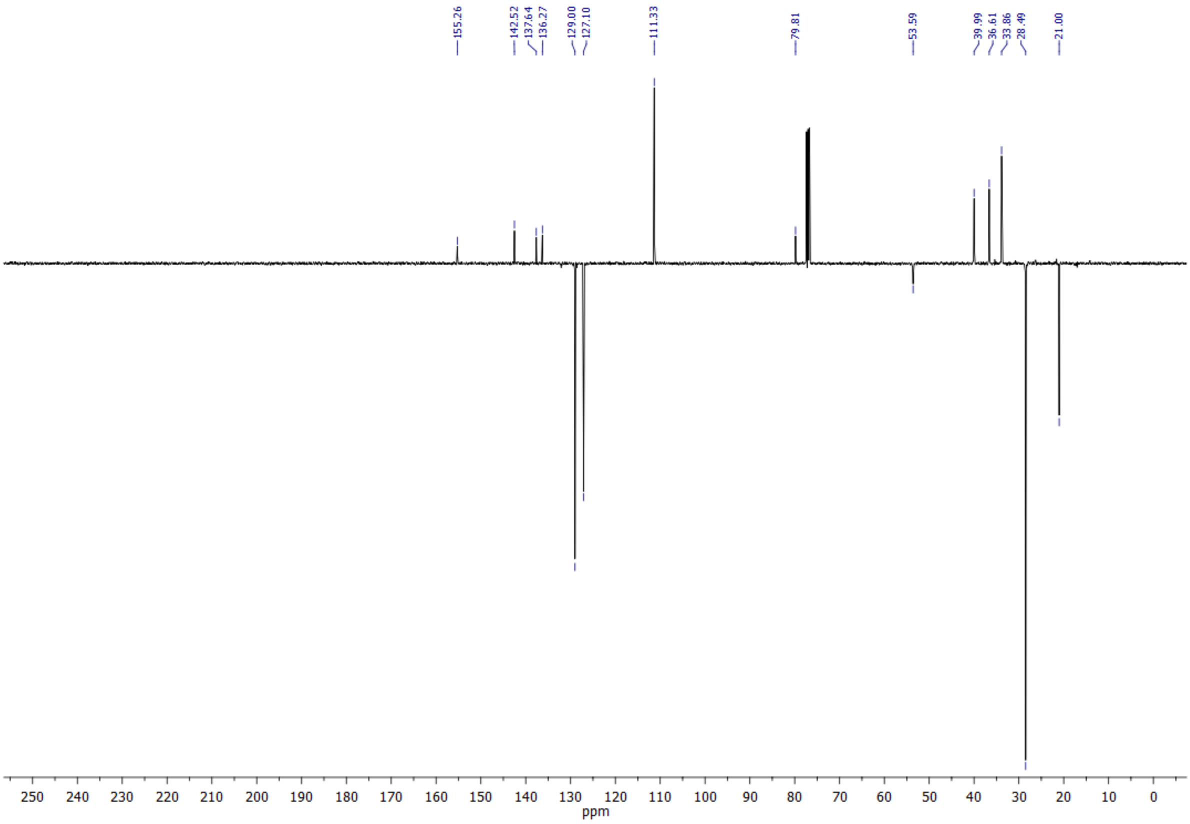

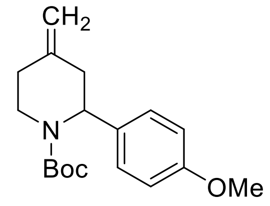

3e

^{1}H NMR spectrum ($CDCl_3$, 400 MHz):

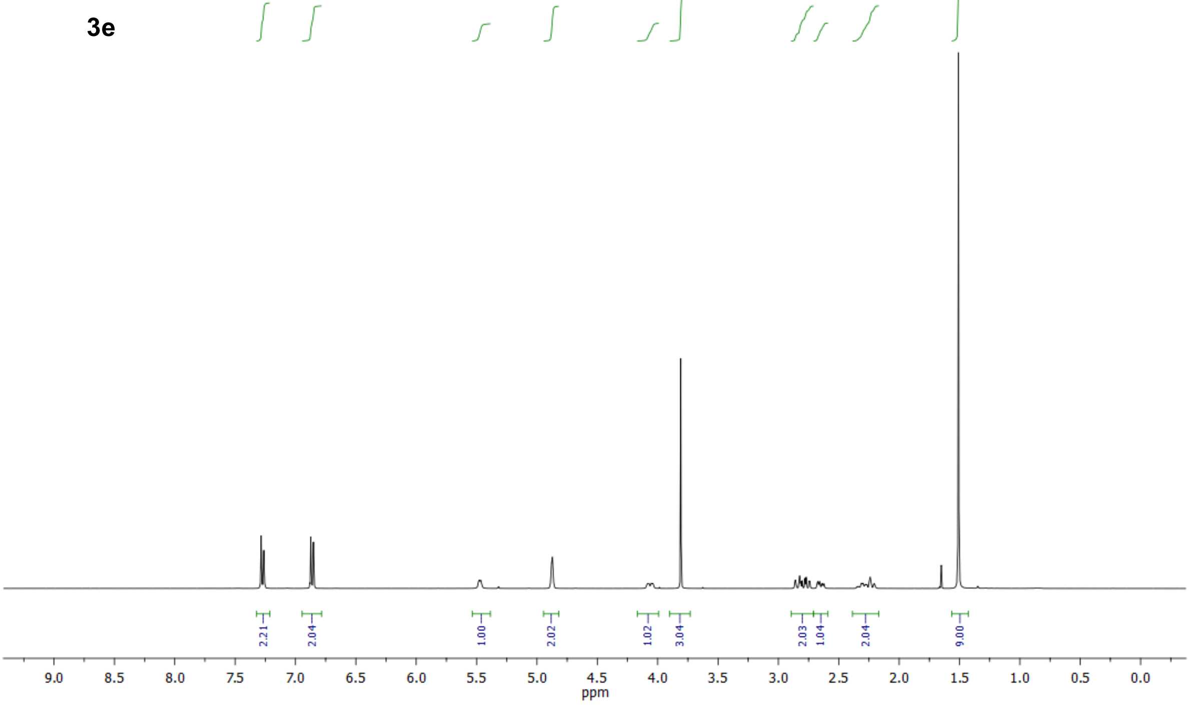

^{13}C{1H} NMR spectrum ($CDCl_3$, 100 MHz):

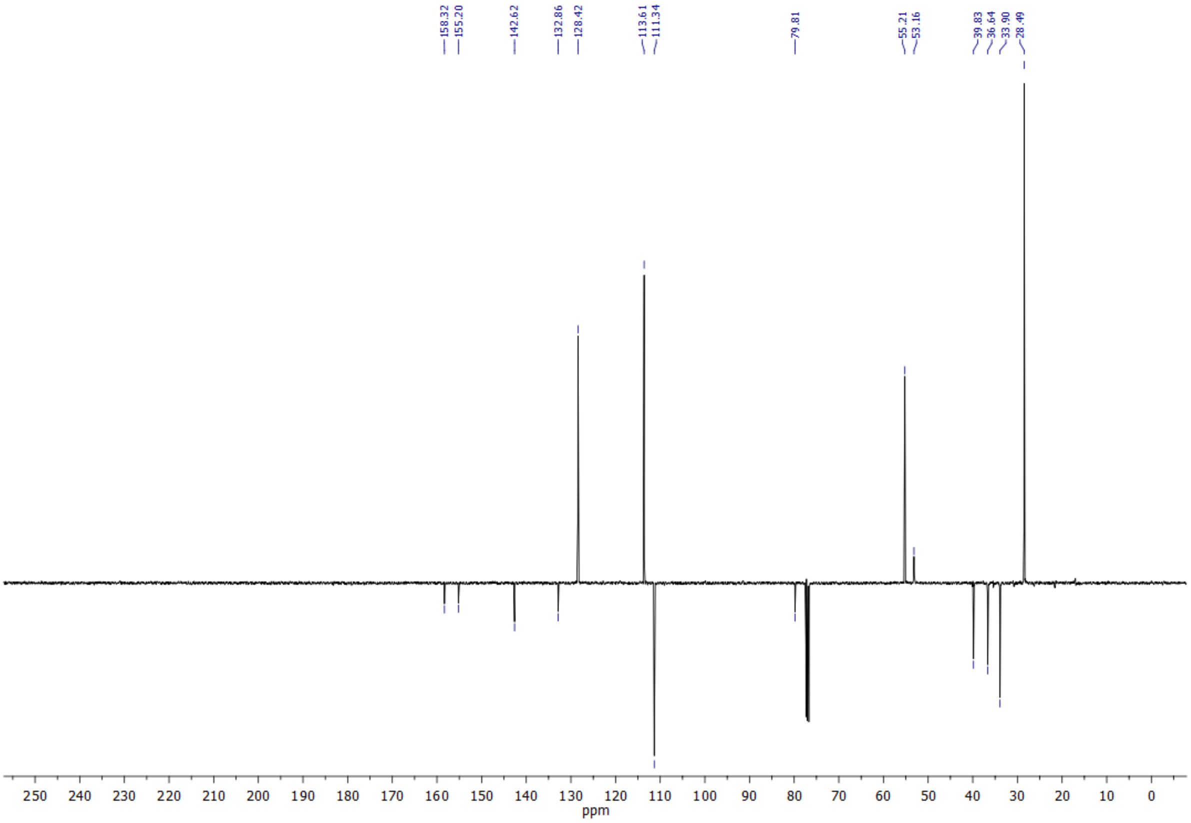

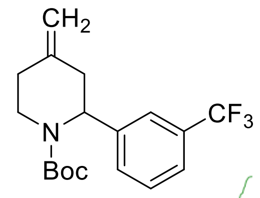

3f

^{1}H NMR spectrum ($CDCl_3$, 400 MHz):

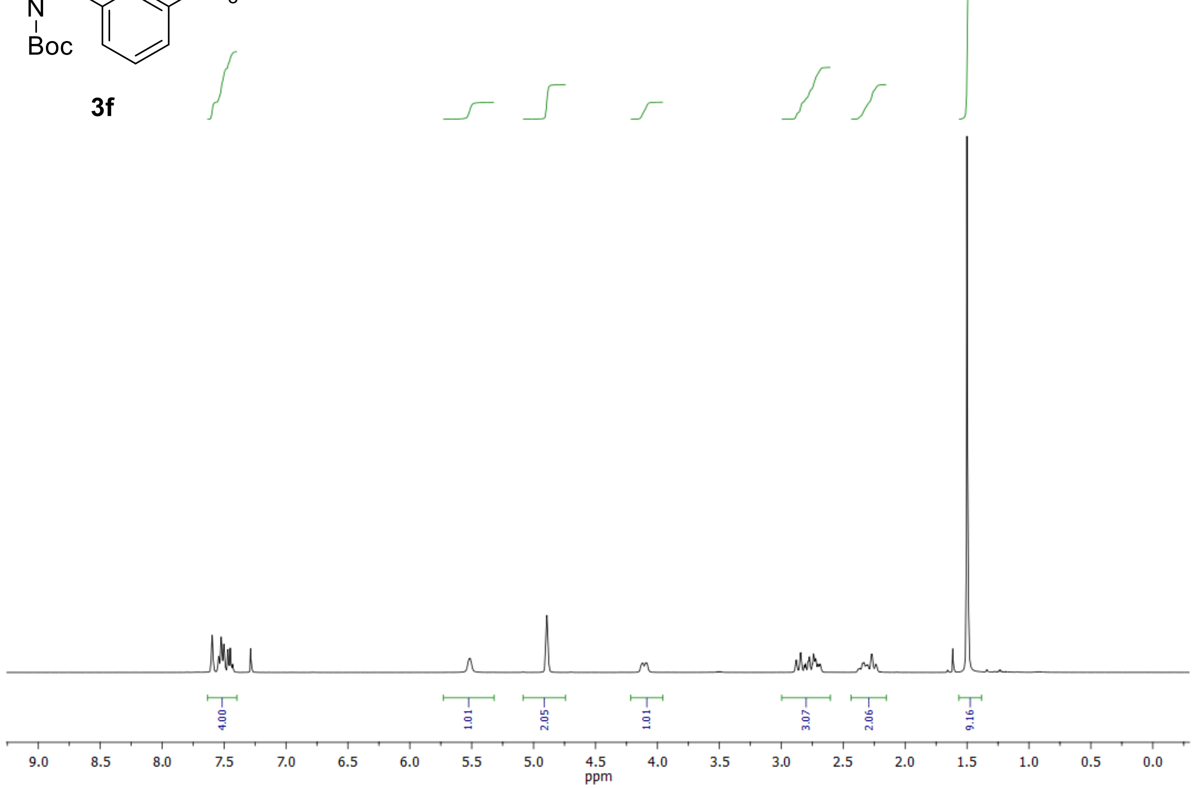

^{13}C{1H} NMR spectrum ($CDCl_3$, 100 MHz):

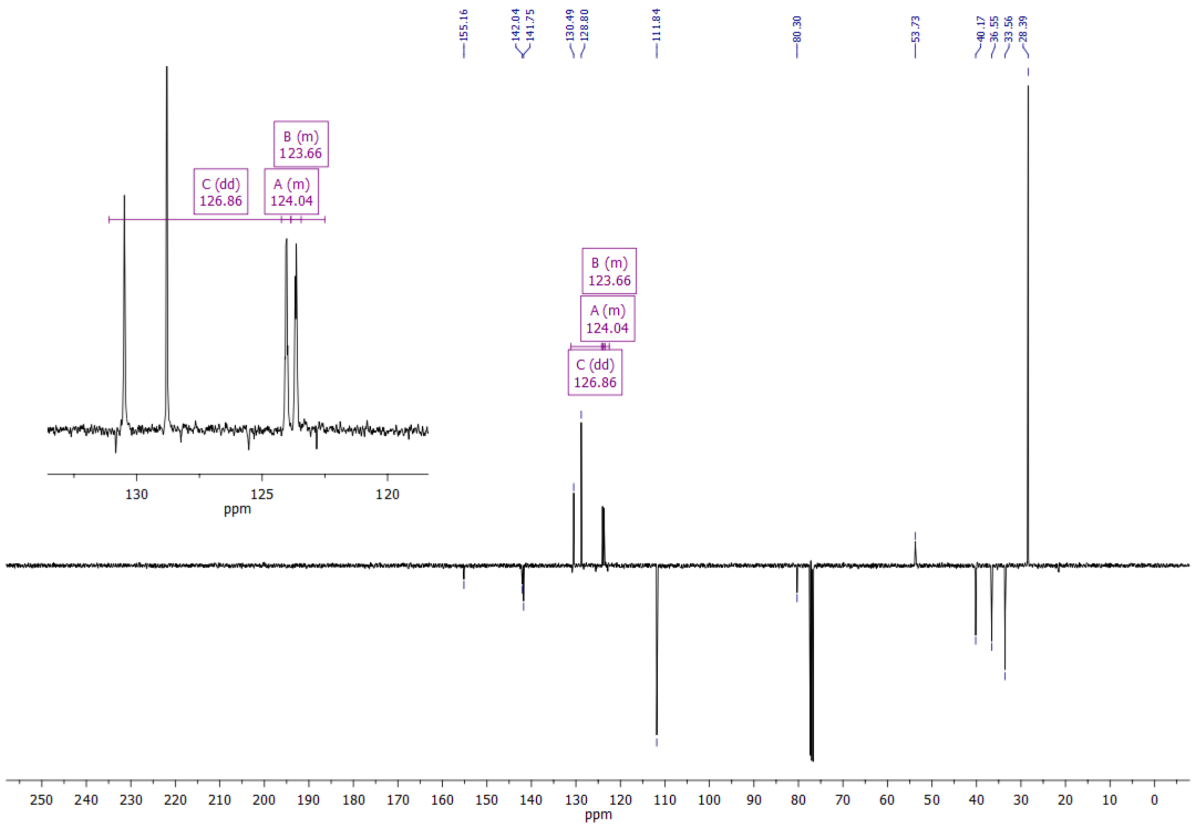

^{19}F NMR spectrum ($CDCl_3$, 377 MHz):

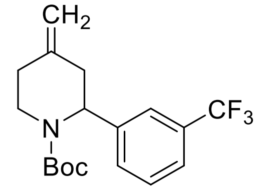

3f (^{19}F-NMR)

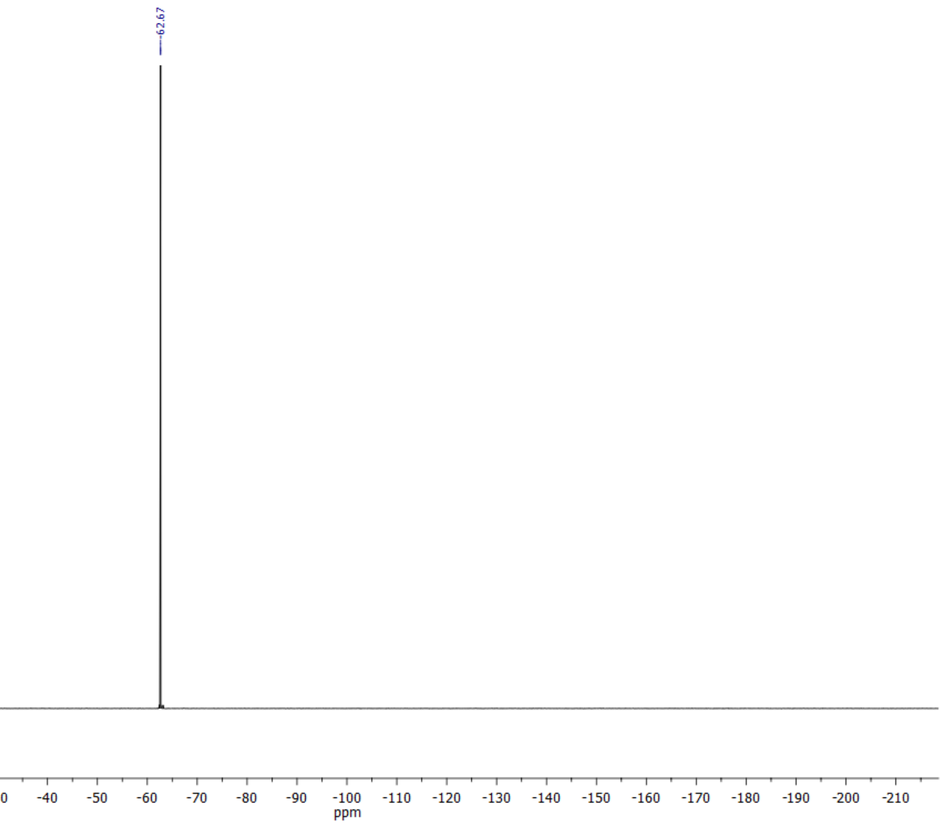

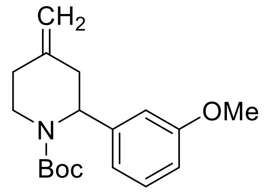

3g

^{1}H NMR spectrum (CDCl$_3$, 400 MHz):

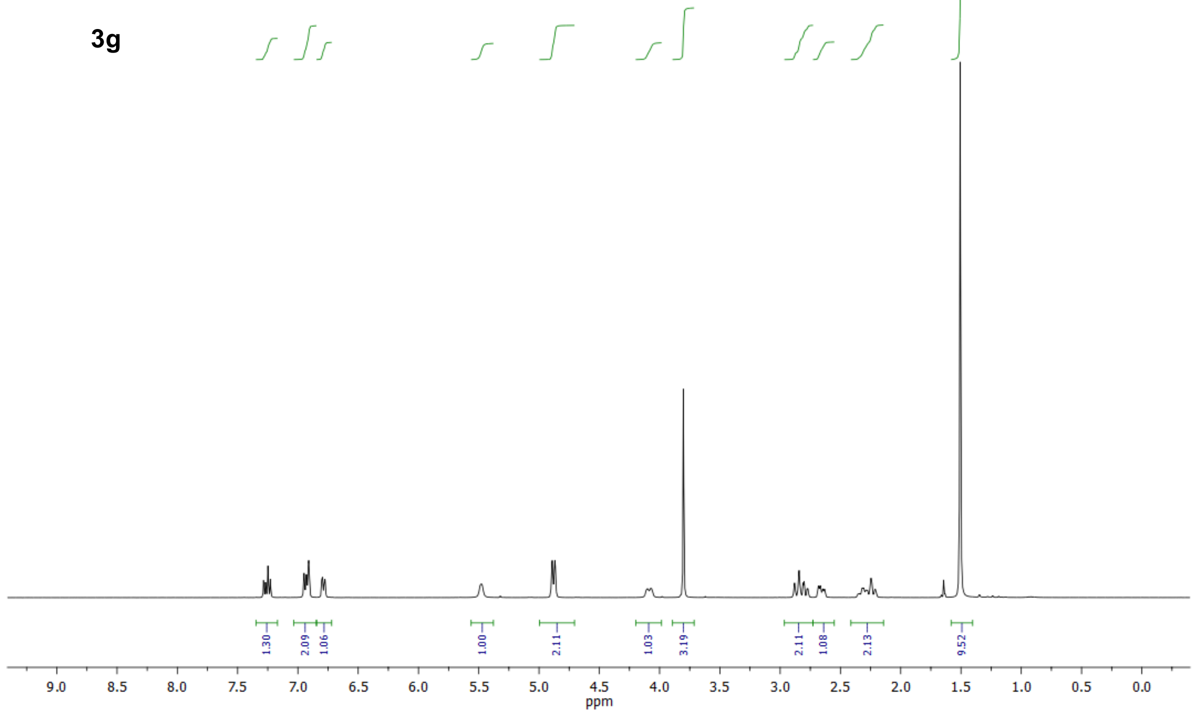

^{13}C{1H} NMR spectrum (CDCl$_3$, 100 MHz):

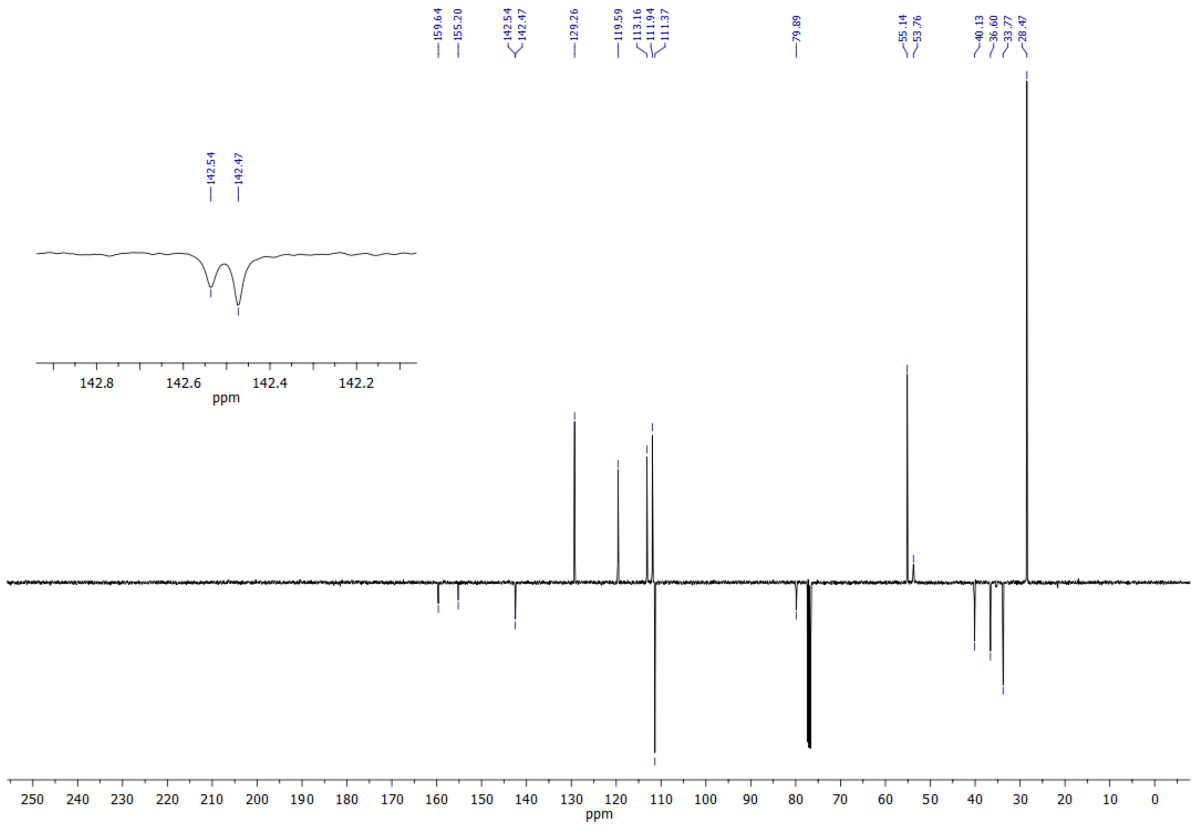

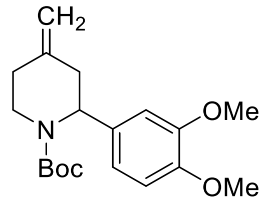

3h

^{1}H NMR spectrum ($CDCl_3$, 400 MHz):

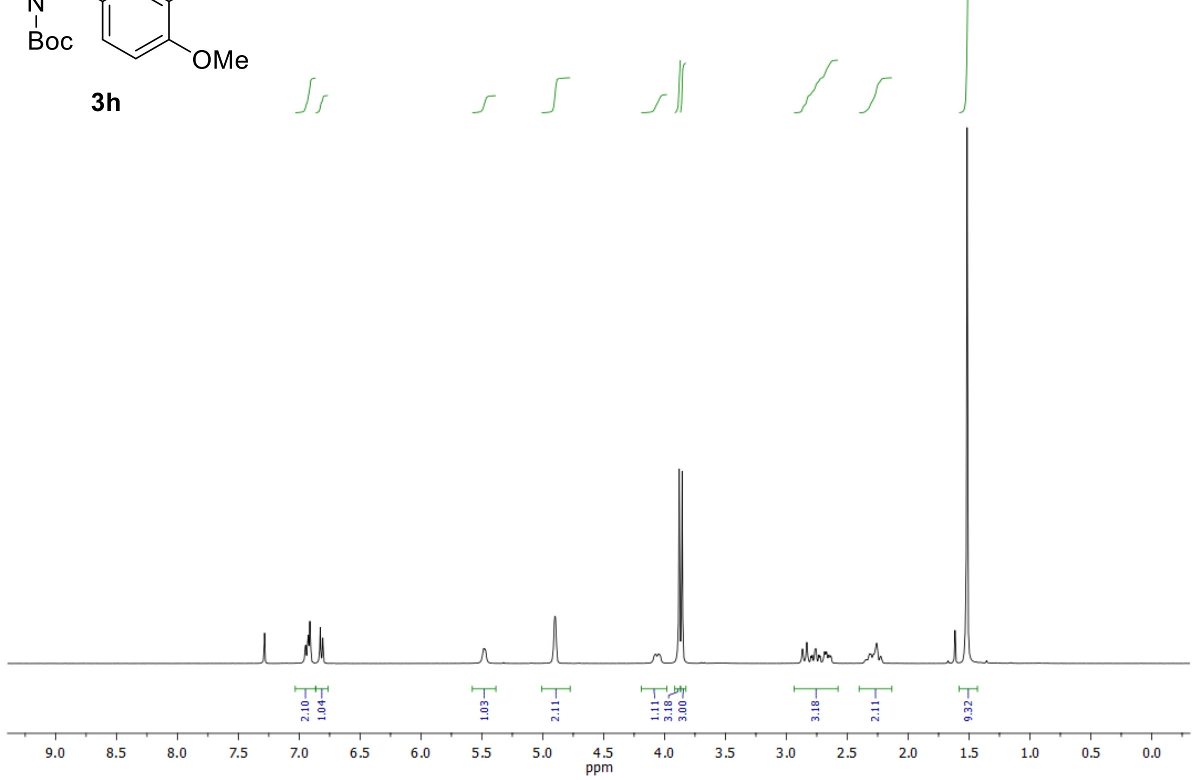

^{13}C{1H} NMR spectrum ($CDCl_3$, 100 MHz):

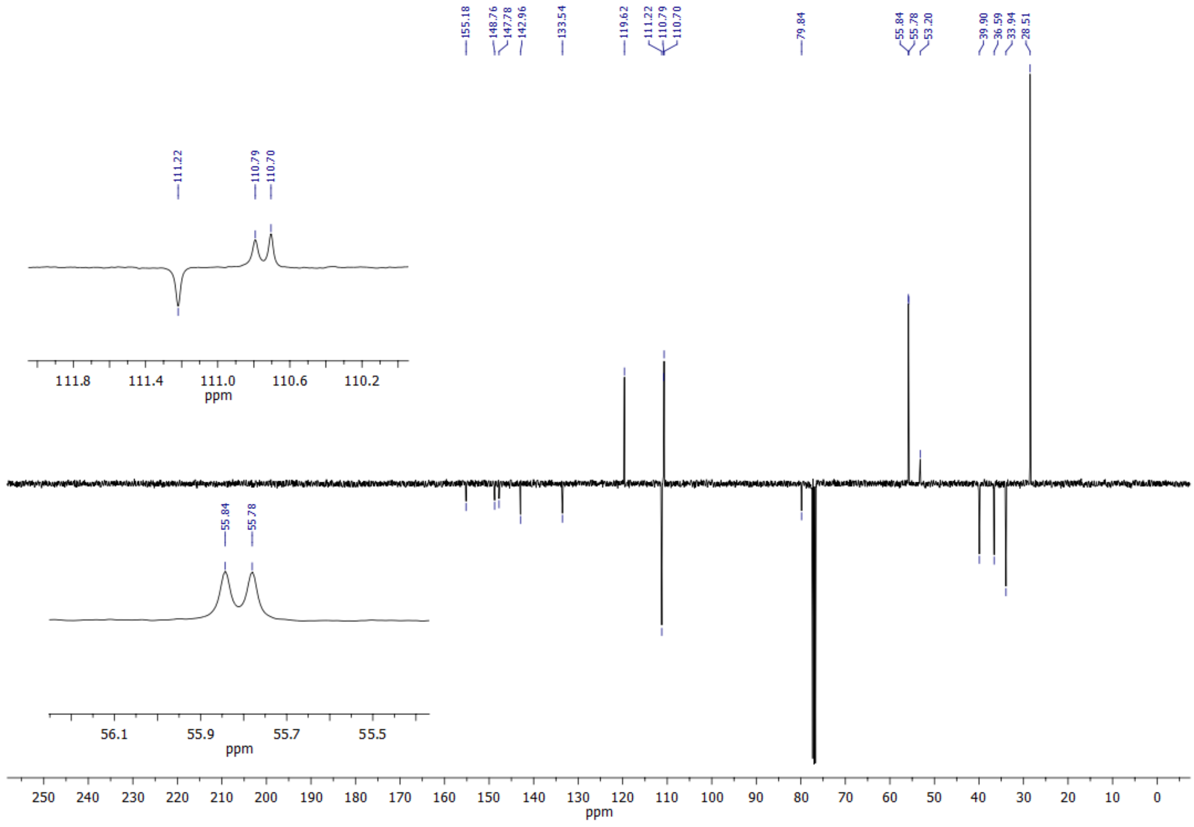

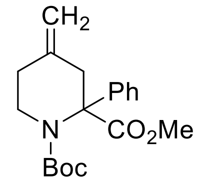

4a

^{1}H NMR spectrum ($CDCl_3$, 400 MHz):

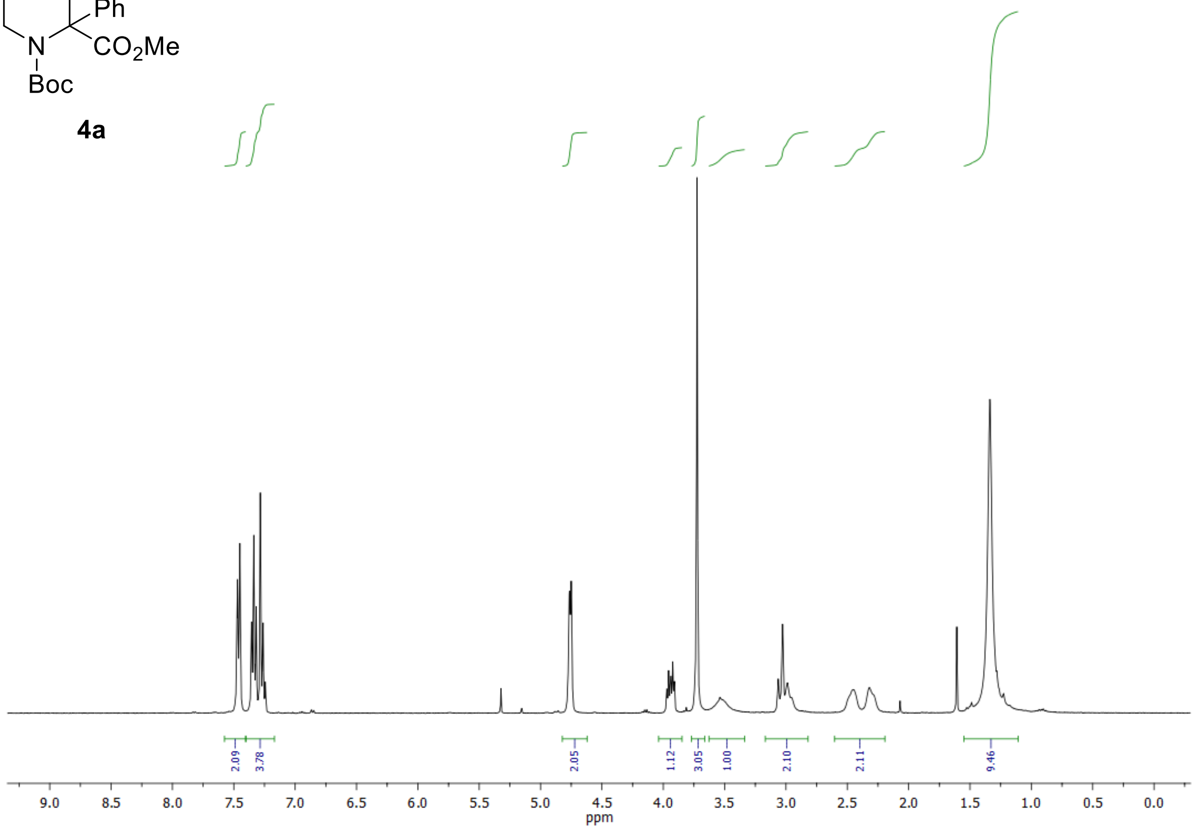

^{13}C{1H} NMR spectrum ($CDCl_3$, 100 MHz):

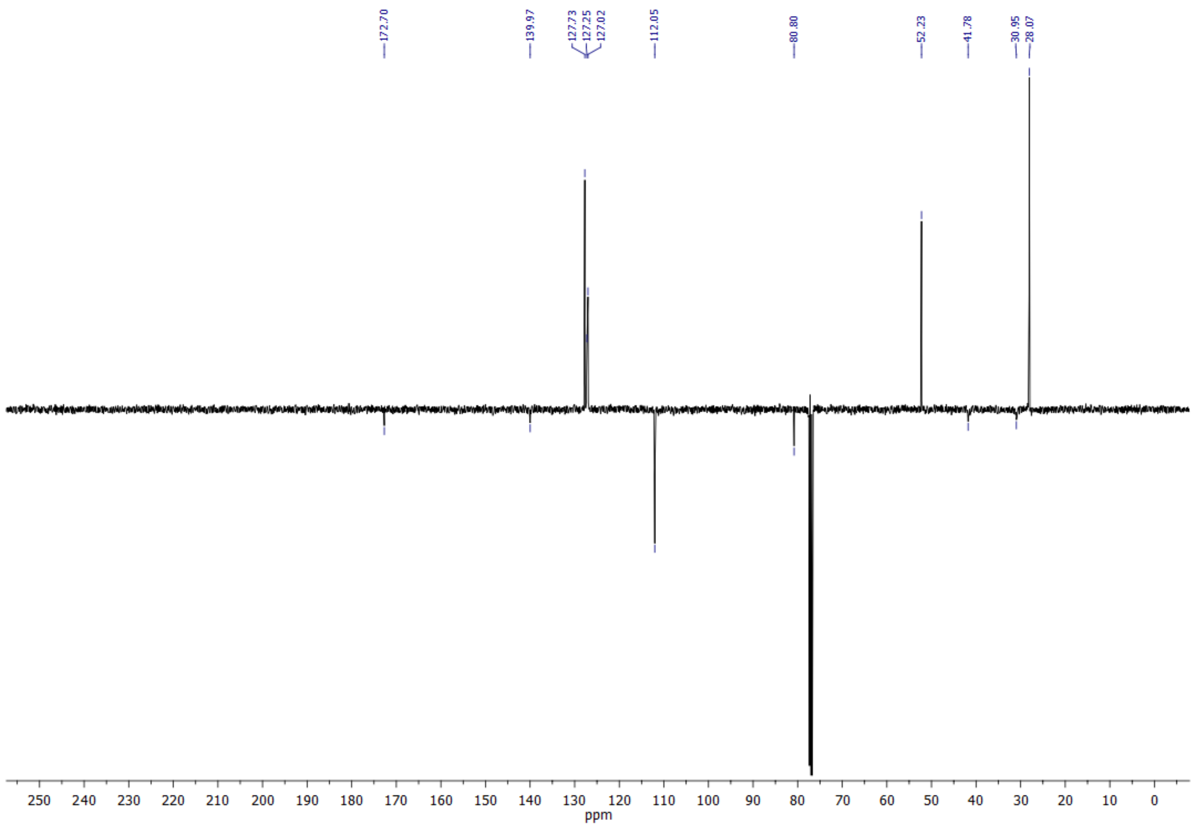

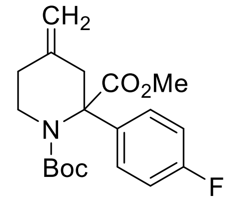

4b

^{1}H NMR spectrum ($CDCl_3$, 400 MHz):

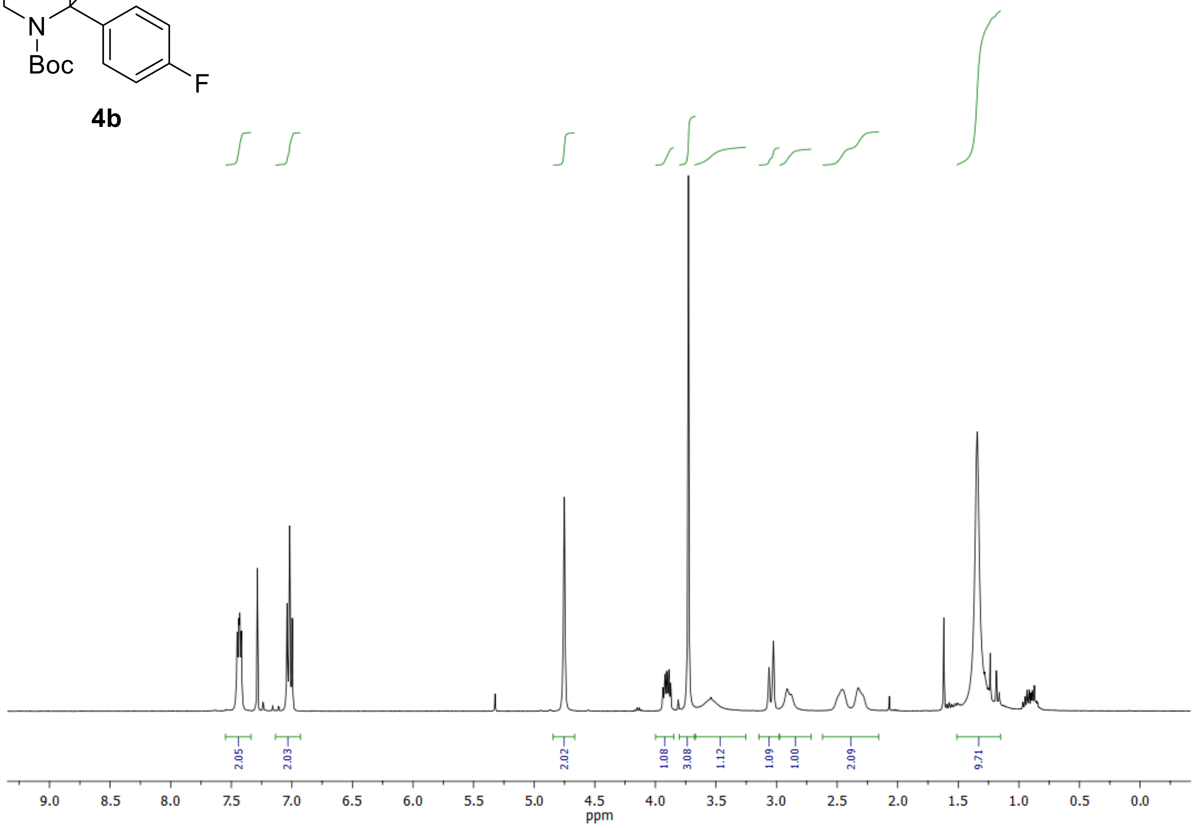

$^{13}C\{1H\}$ NMR spectrum ($CDCl_3$, 100 MHz):

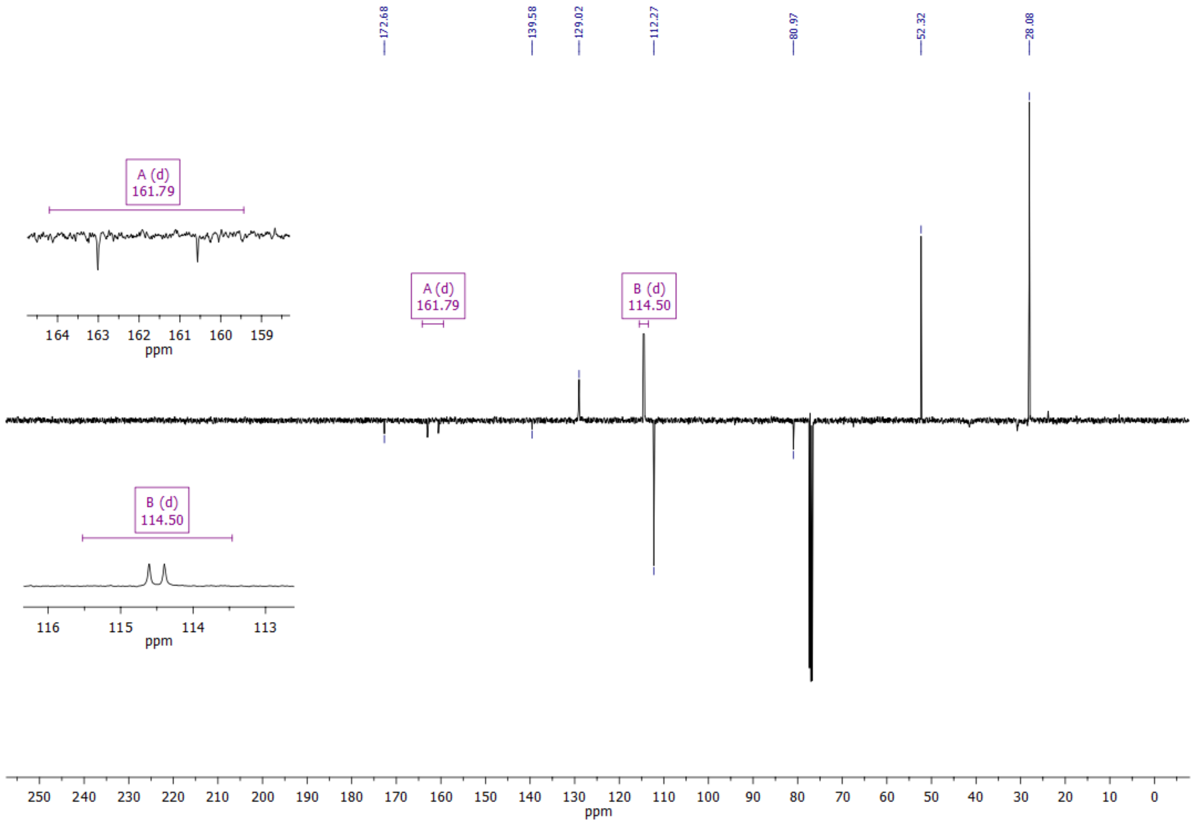

^{19}F NMR spectrum ($CDCl_3$, 377 MHz):

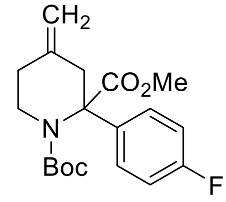

4b (^{19}F-NMR)

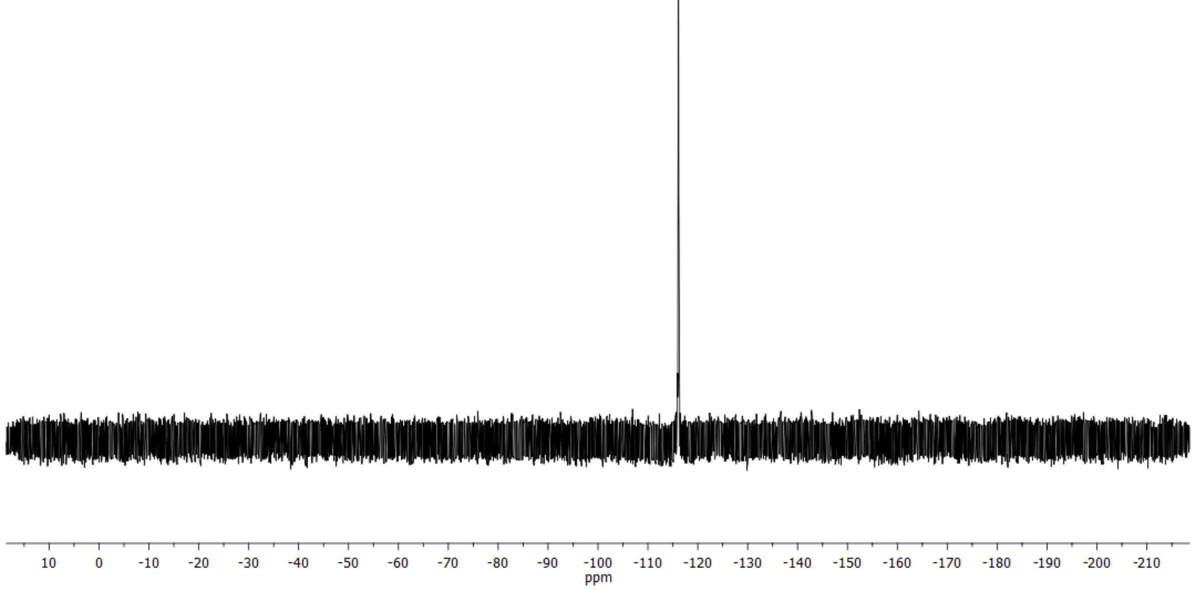

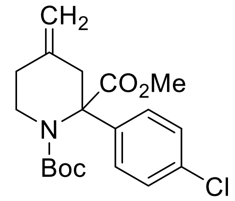

4c

^{1}H NMR spectrum ($CDCl_3$, 400 MHz):

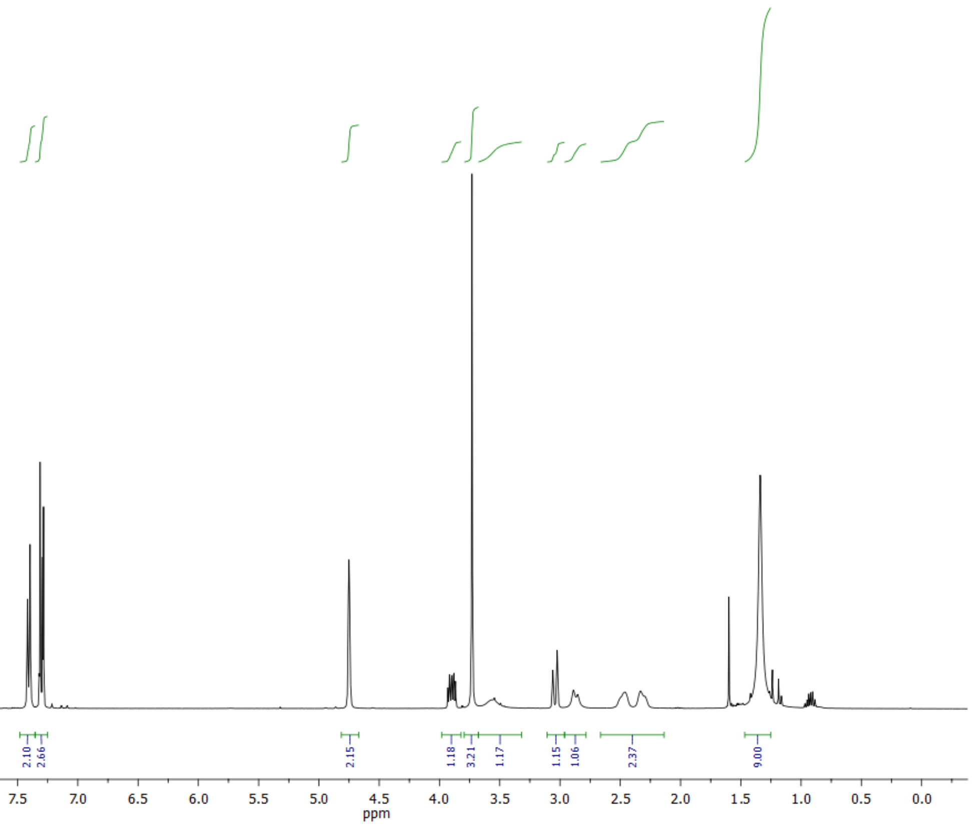

^{13}C{1H} NMR spectrum ($CDCl_3$, 100 MHz):

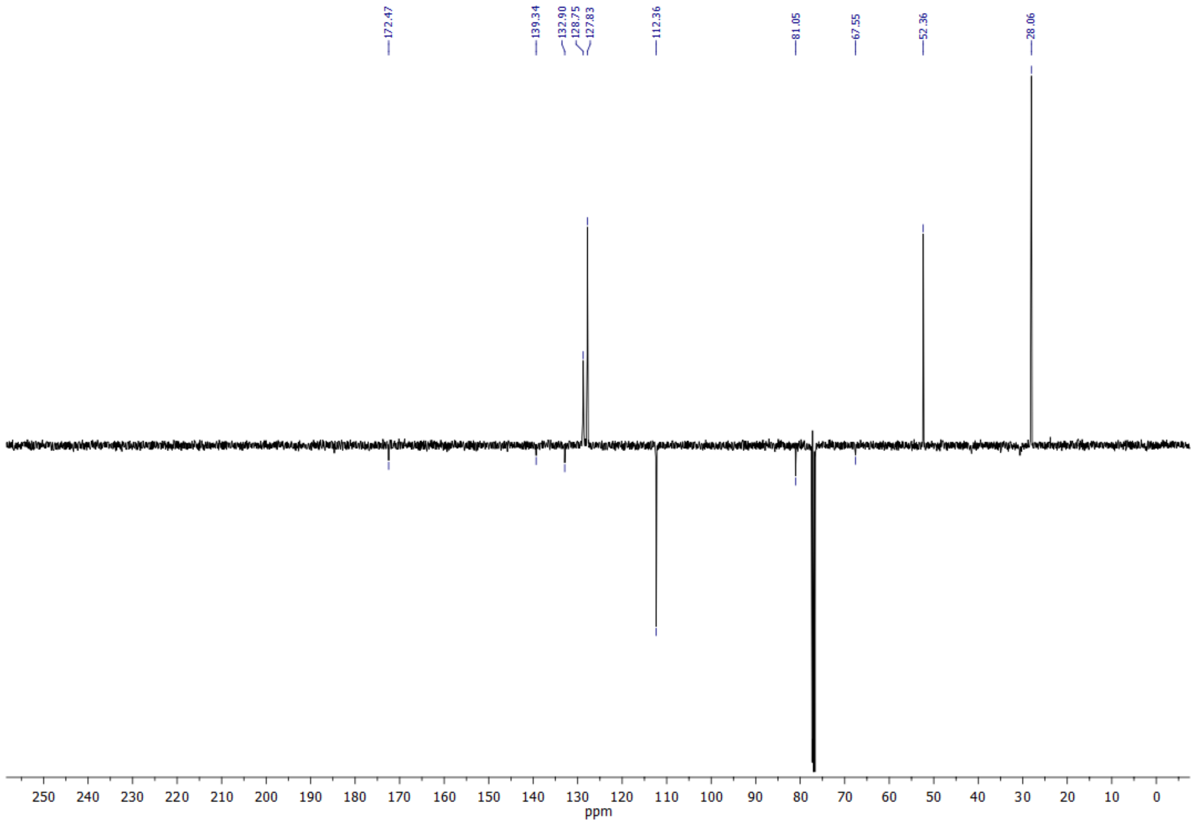

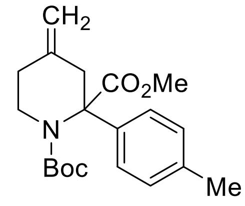

4d

^{1}H NMR spectrum ($CDCl_3$, 400 MHz):

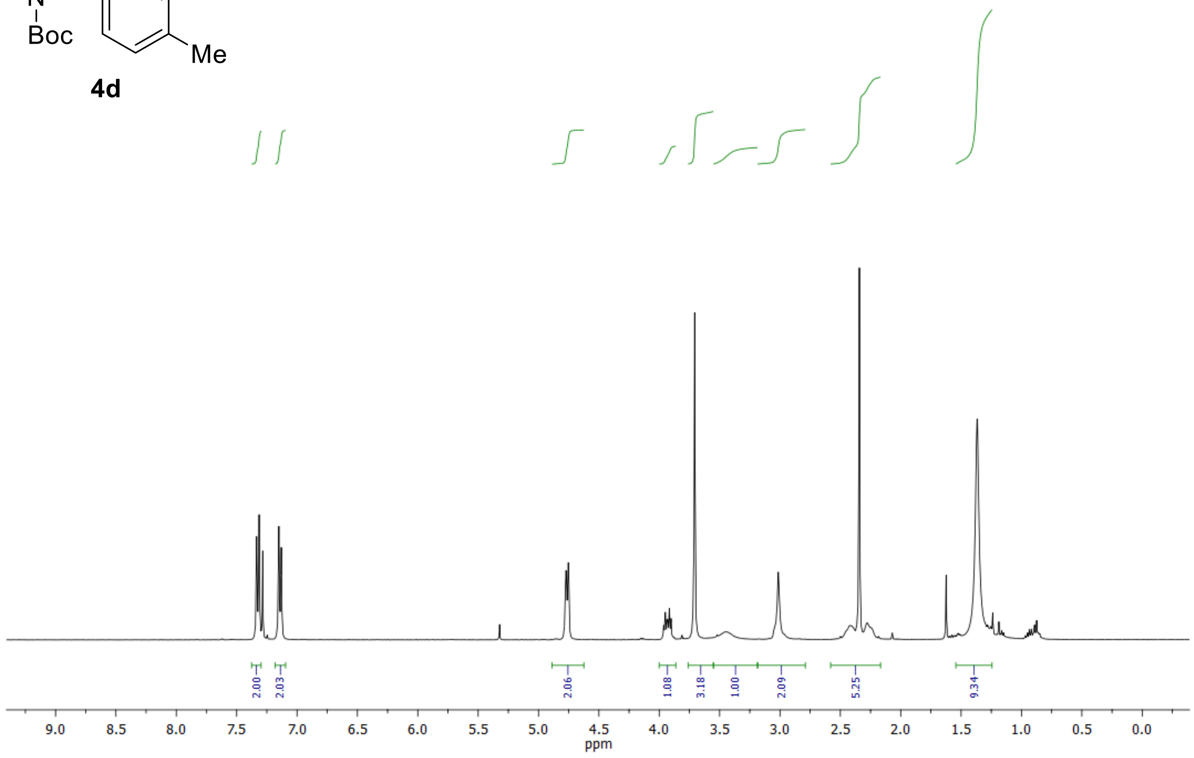

^{13}C{1H} NMR spectrum ($CDCl_3$, 100 MHz):

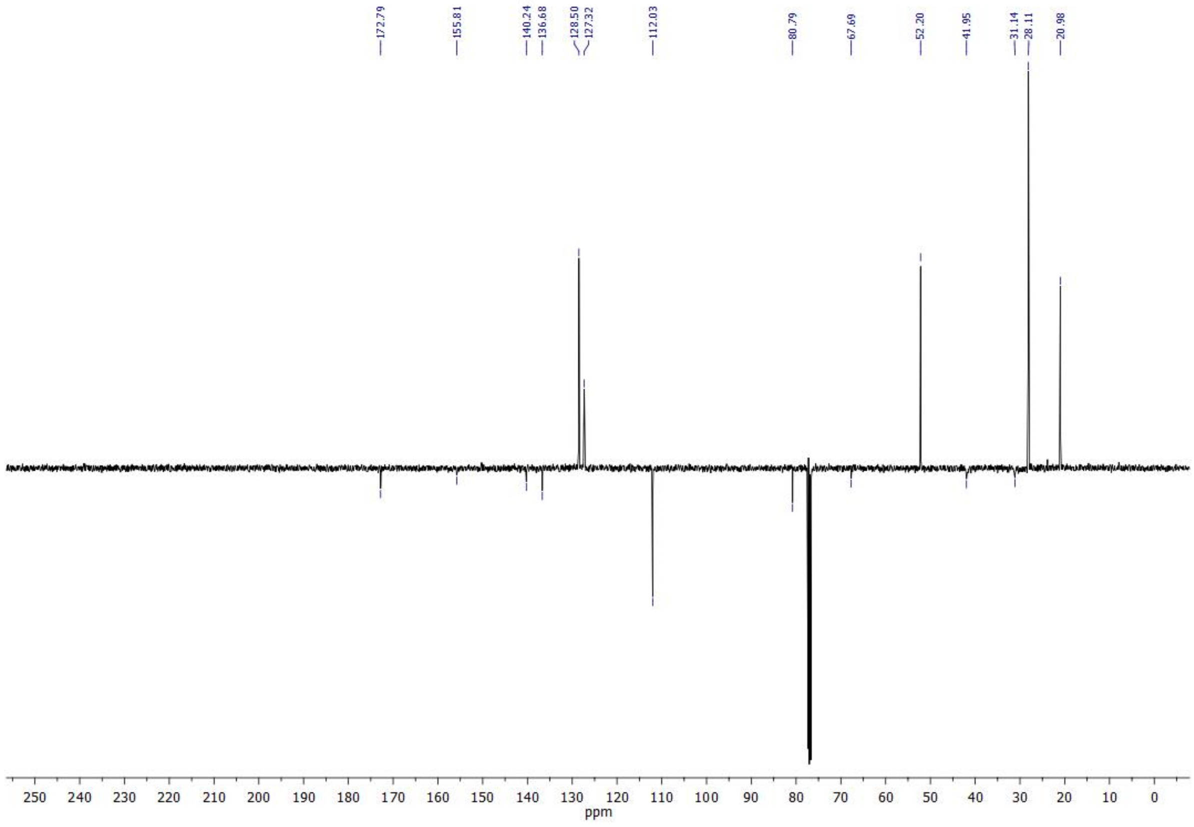

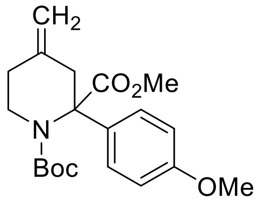

4e

^{1}H NMR spectrum ($CDCl_3$, 400 MHz):

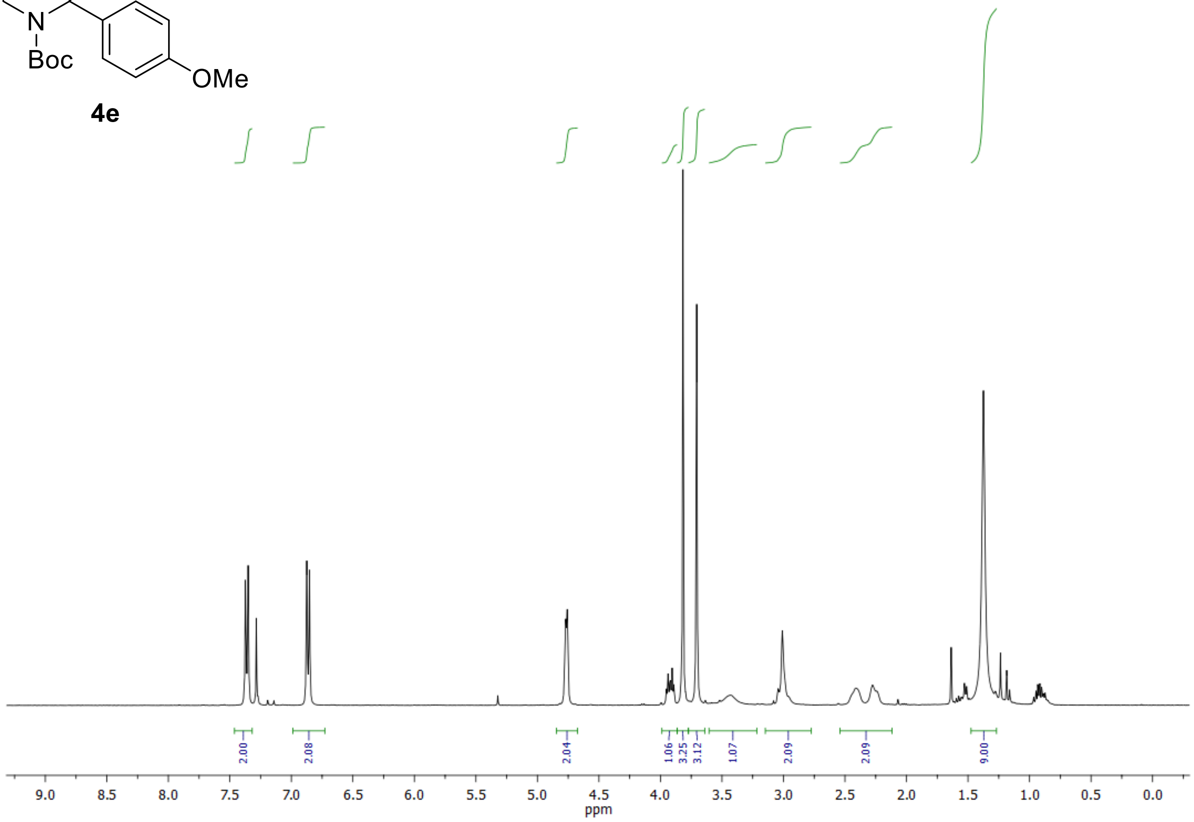

^{13}C{1H} NMR spectrum ($CDCl_3$, 100 MHz):

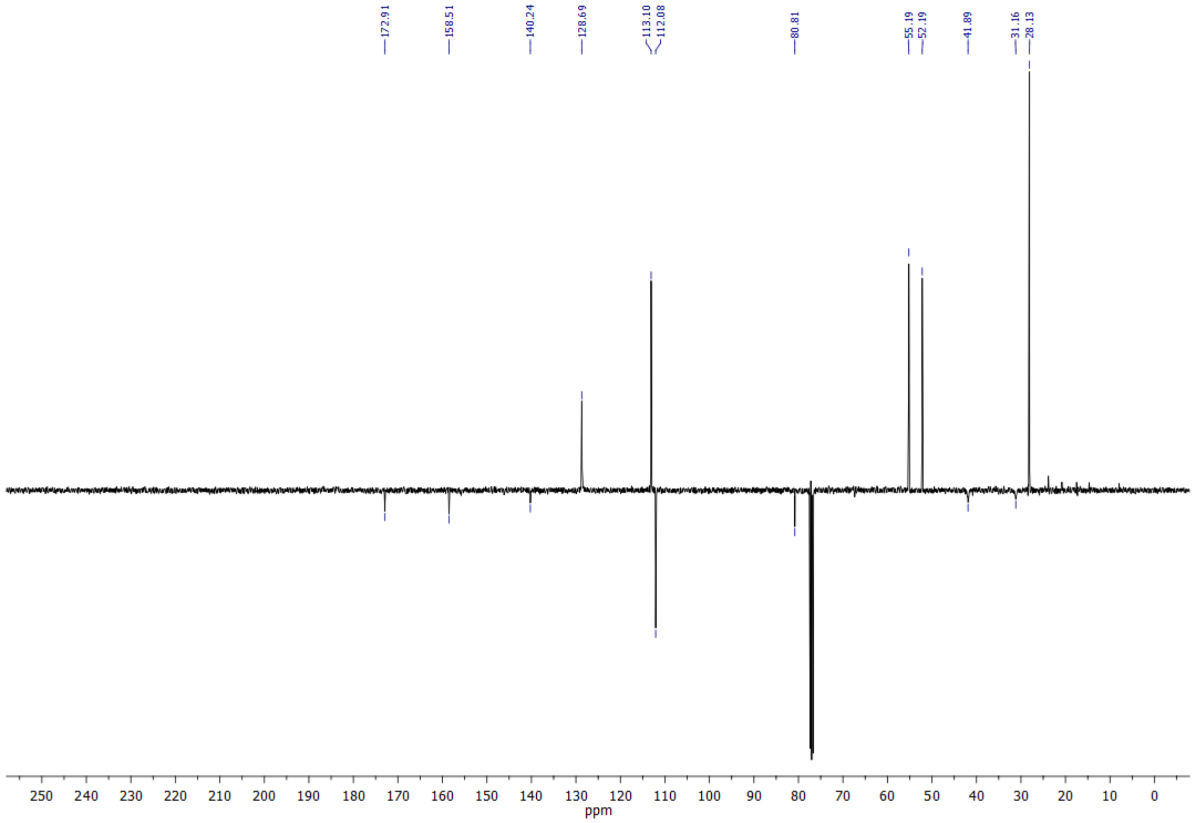

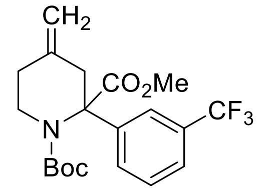

4f

^{1}H NMR spectrum ($CDCl_3$, 400 MHz):

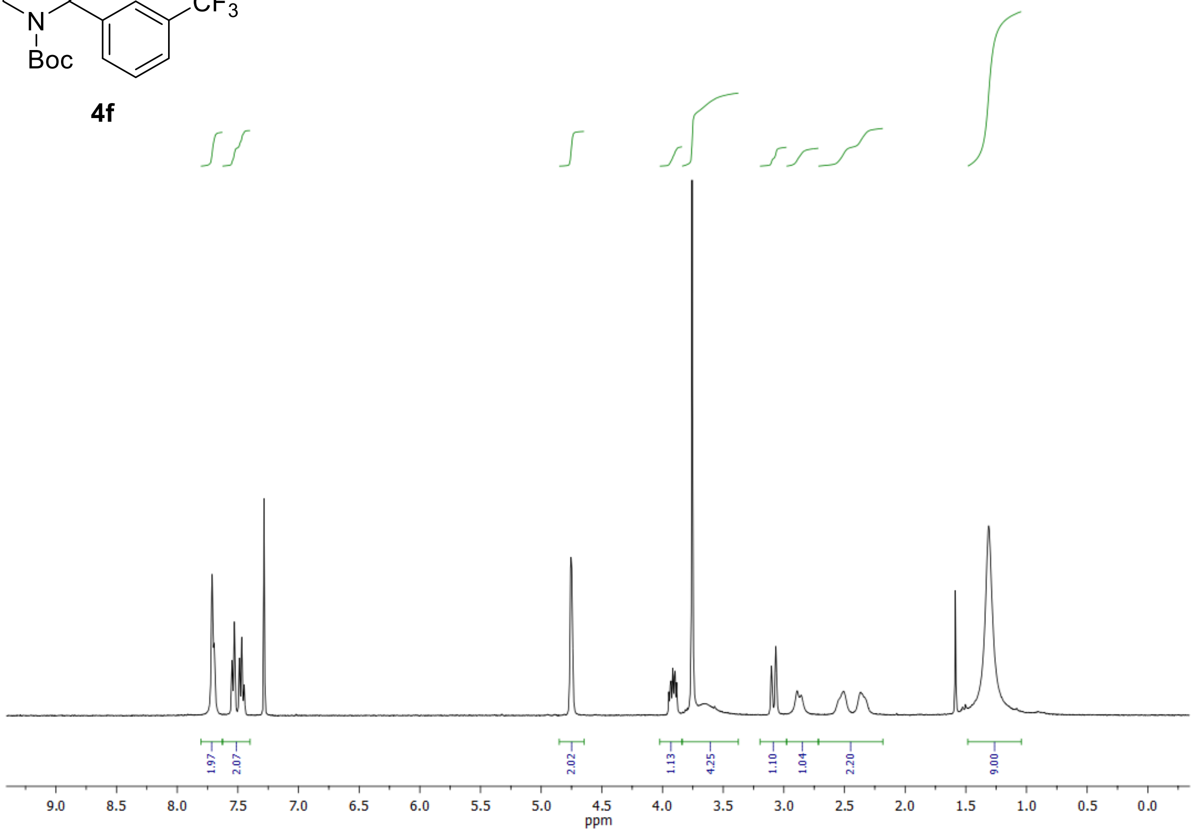

^{13}C{1H} NMR spectrum ($CDCl_3$, 100 MHz):

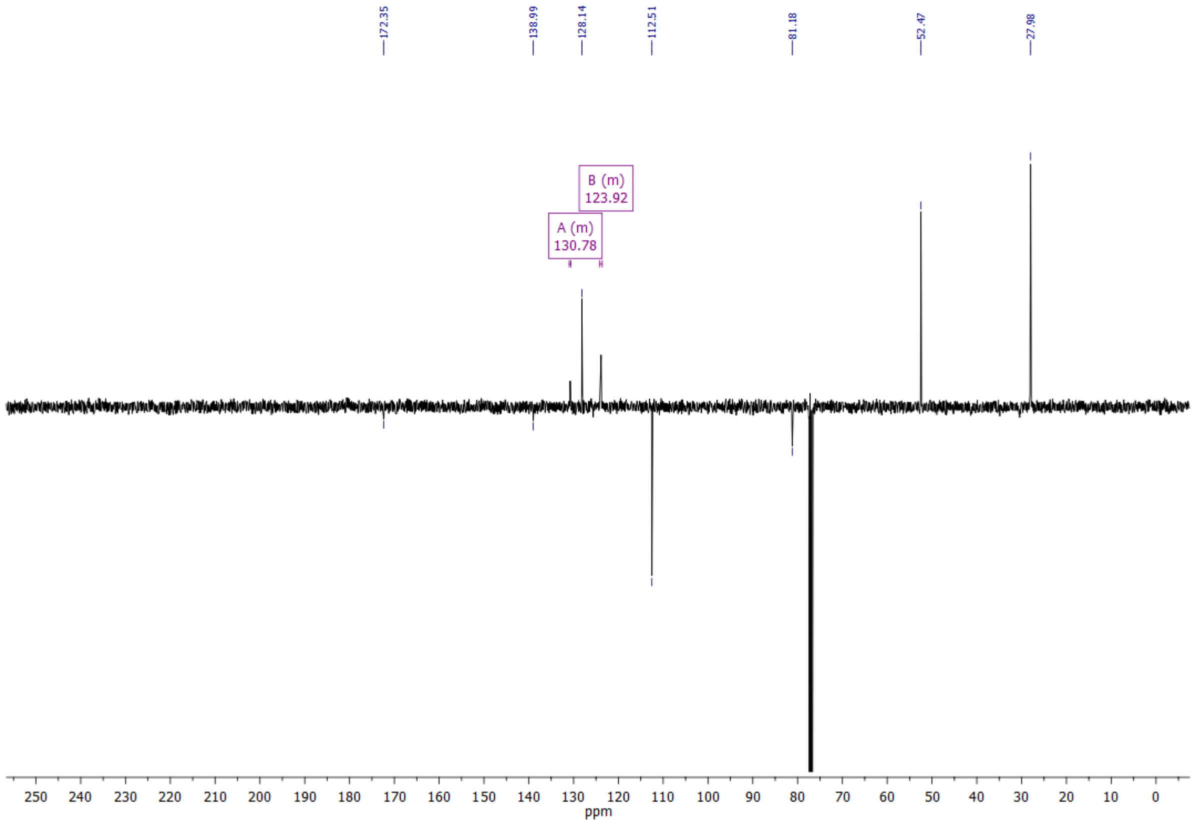

^{19}F NMR spectrum ($CDCl_3$, 377 MHz):

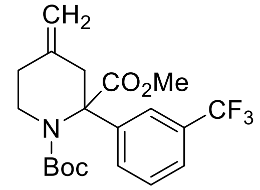

4f (^{19}F-NMR)

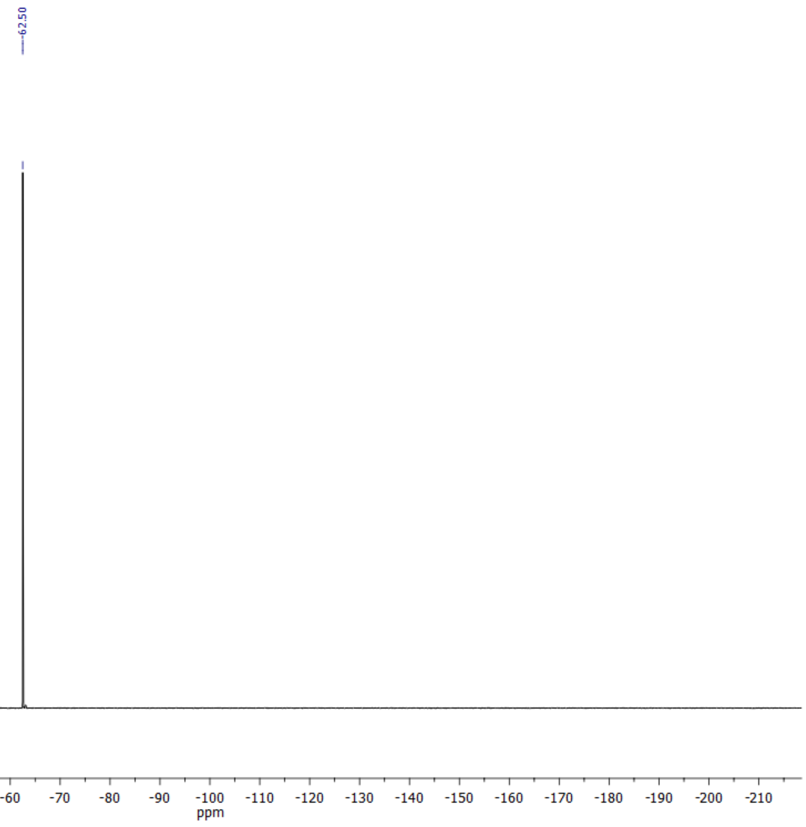

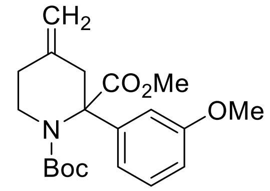

4g

^{1}H NMR spectrum (CDCl$_3$, 400 MHz):

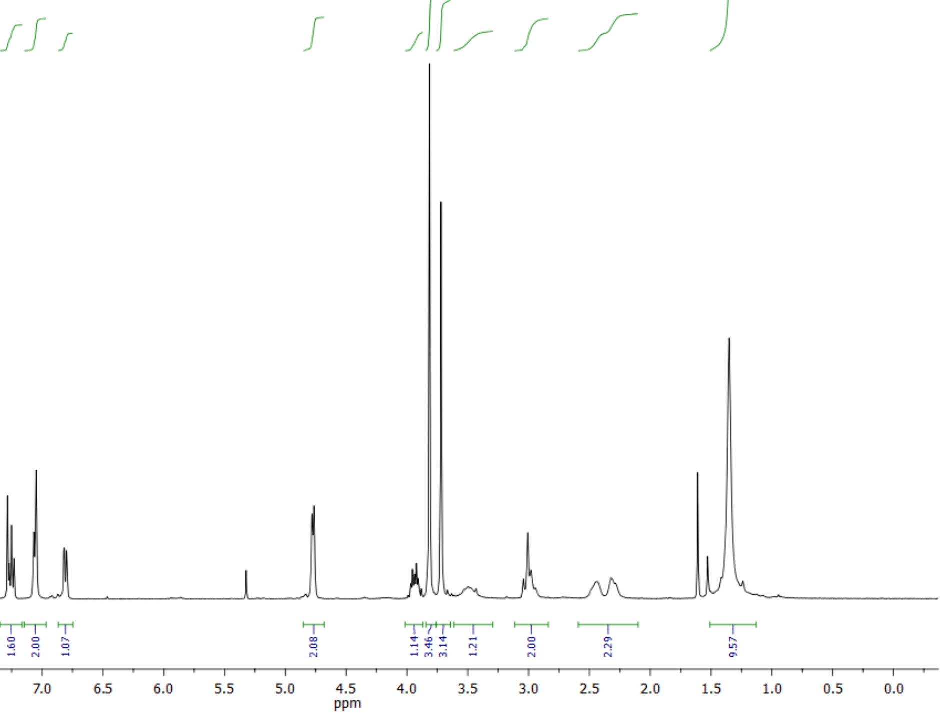

^{13}C{1H} NMR spectrum (CDCl$_3$, 100 MHz):

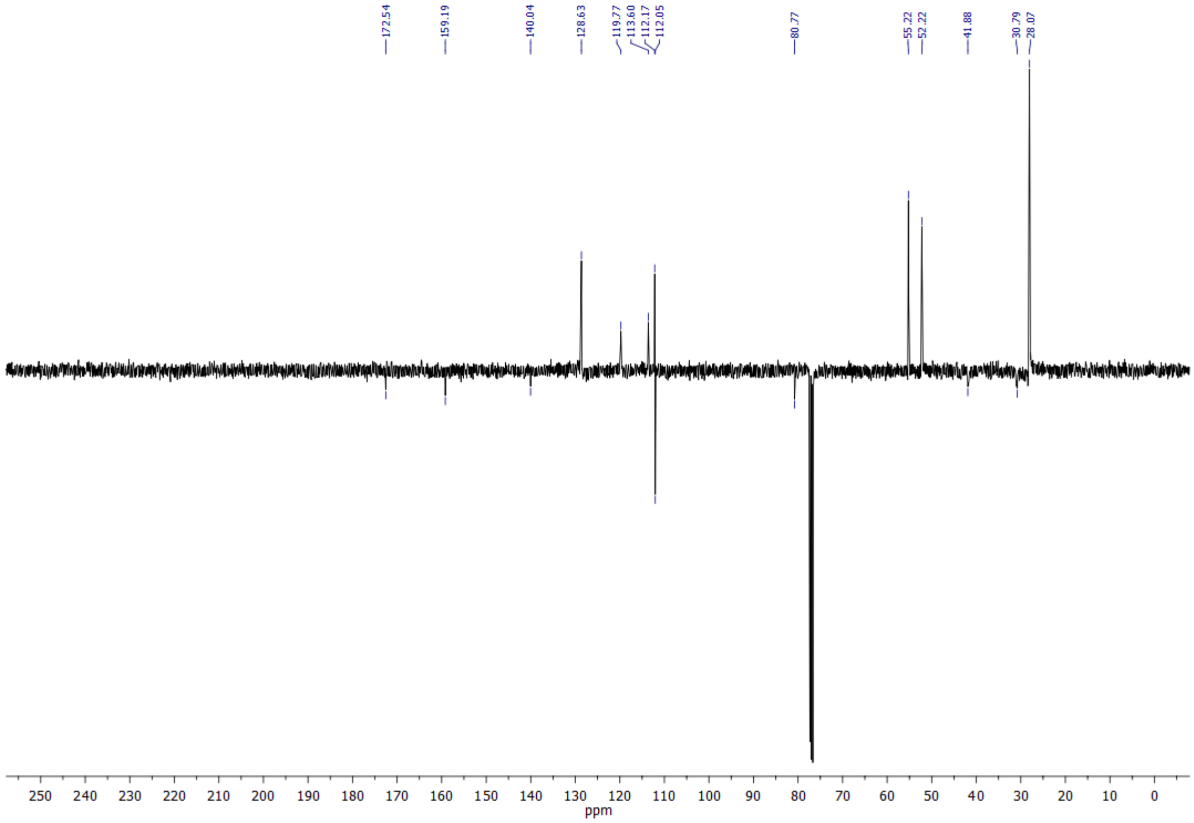

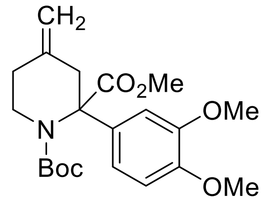

4h

^{1}H NMR spectrum ($CDCl_3$, 400 MHz):

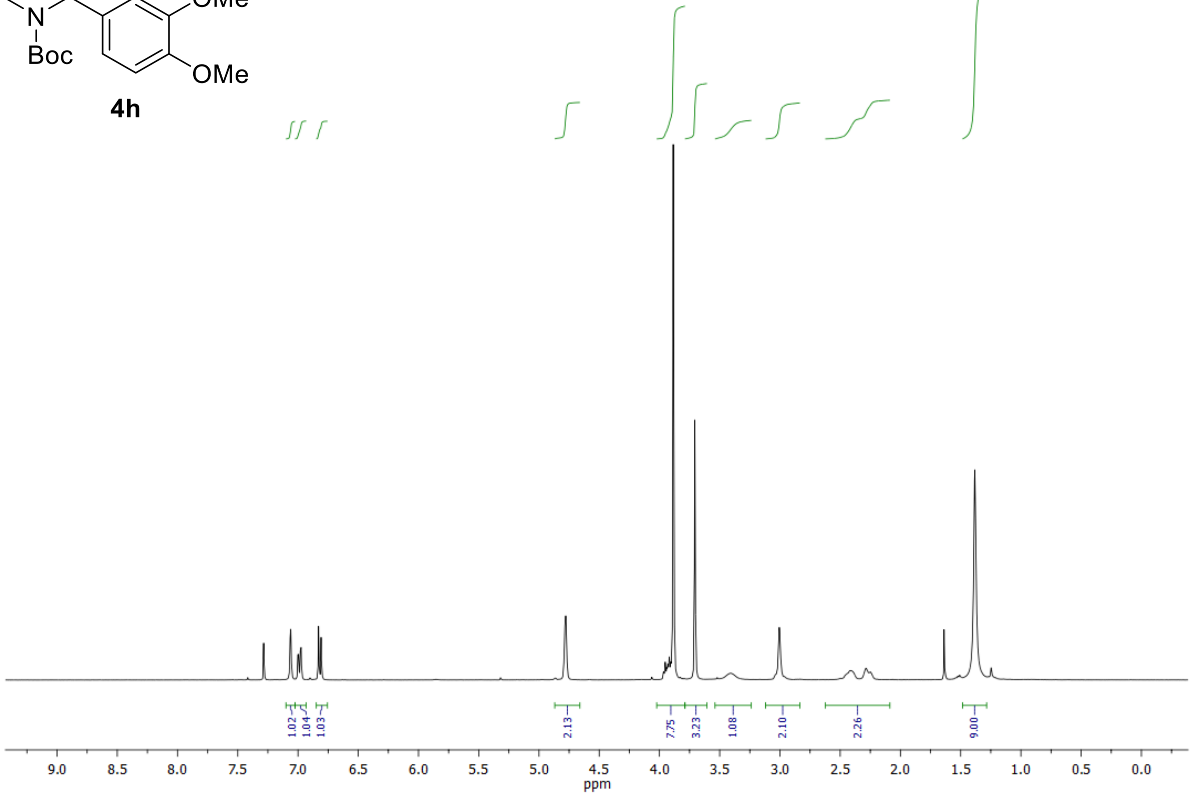

^{13}C{1H} NMR spectrum ($CDCl_3$, 100 MHz):

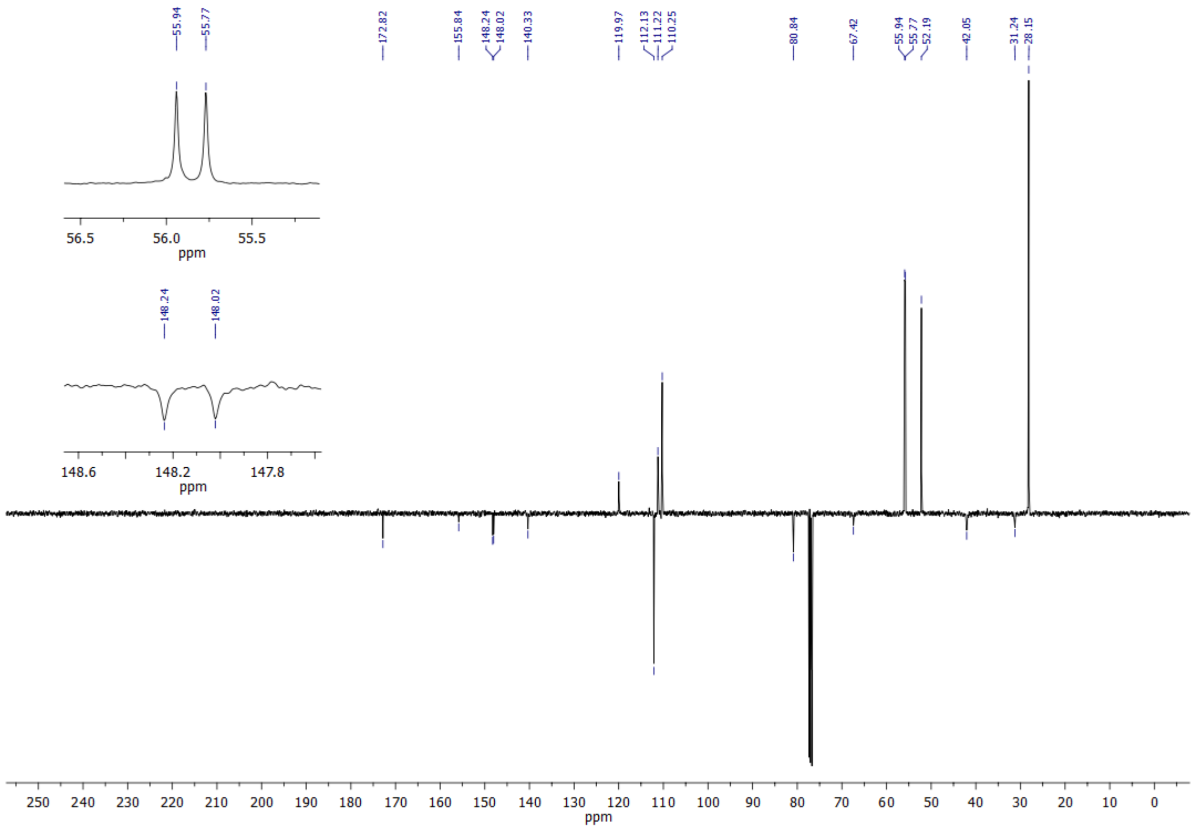

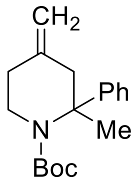

5a

^{1}H NMR spectrum ($CDCl_3$, 400 MHz):

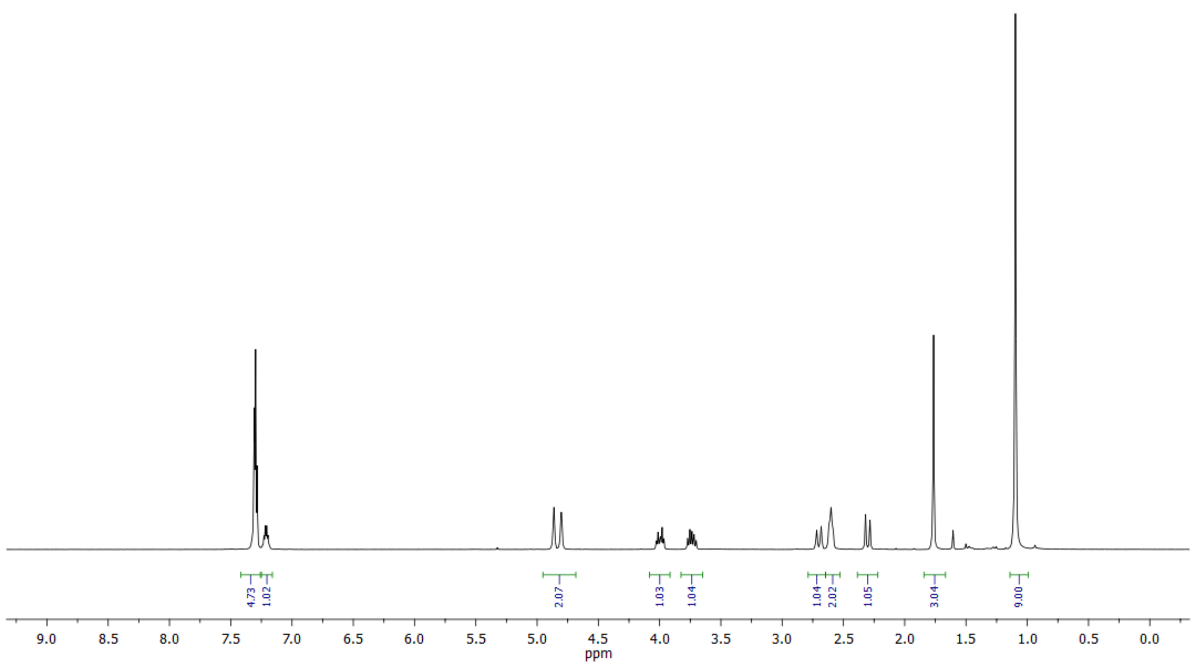

^{13}C{1H} NMR spectrum ($CDCl_3$, 100 MHz):

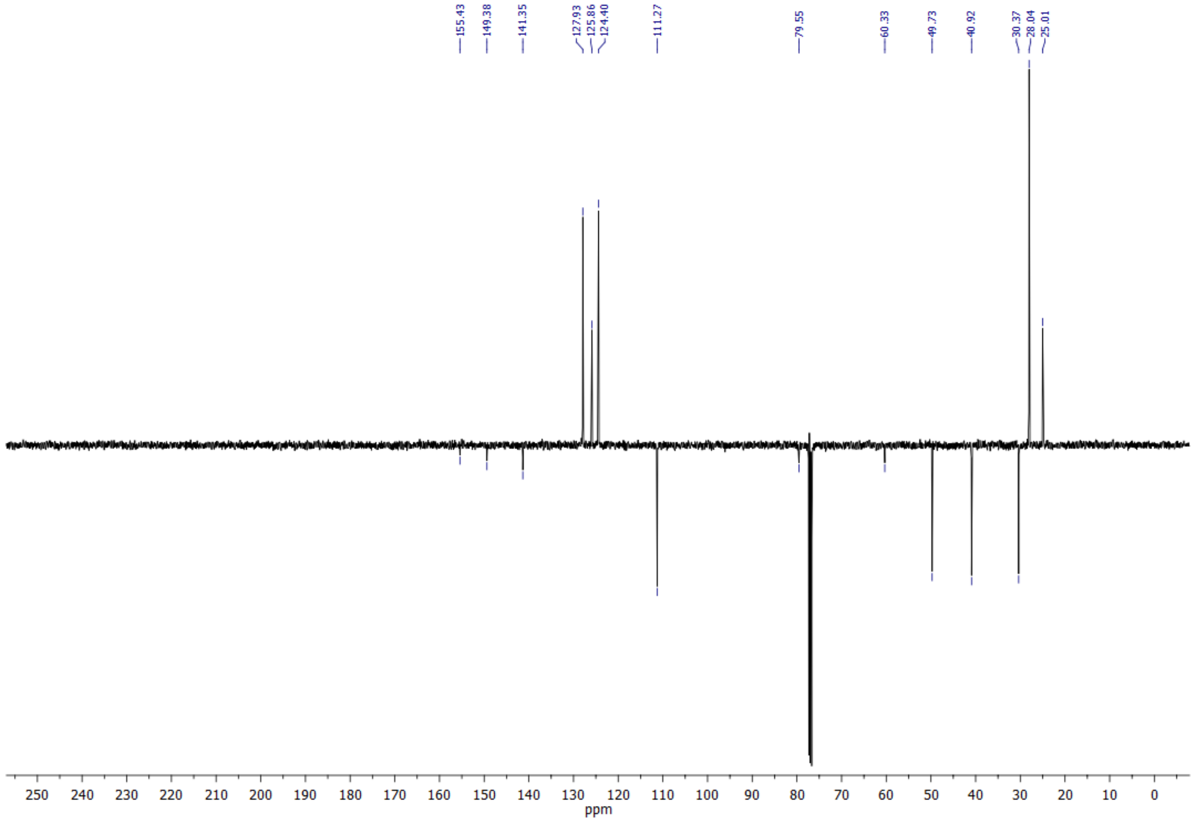

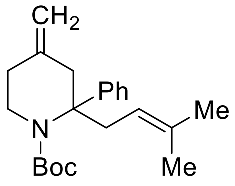

6a

^{1}H NMR spectrum ($CDCl_3$, 400 MHz):

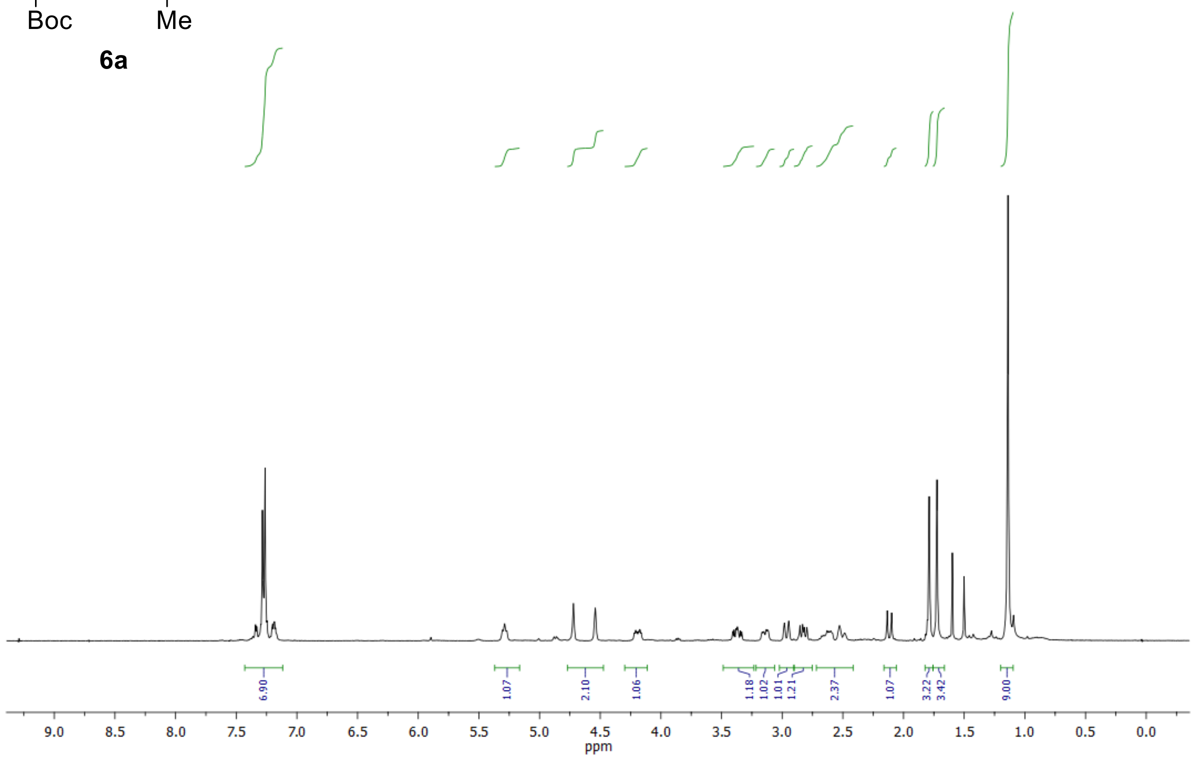

^{13}C{1H} NMR spectrum ($CDCl_3$, 100 MHz):

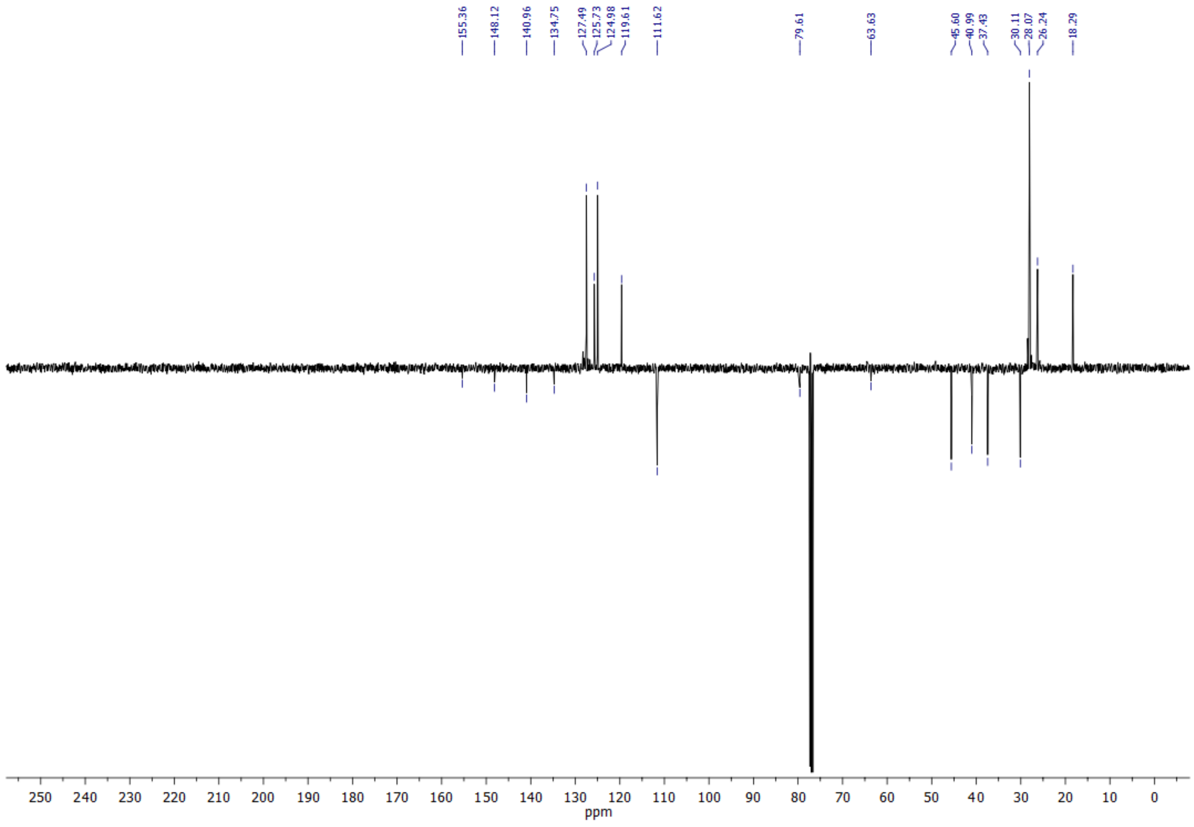

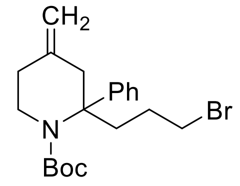

7a

^1H NMR spectrum ($CDCl_3$, 400 MHz):

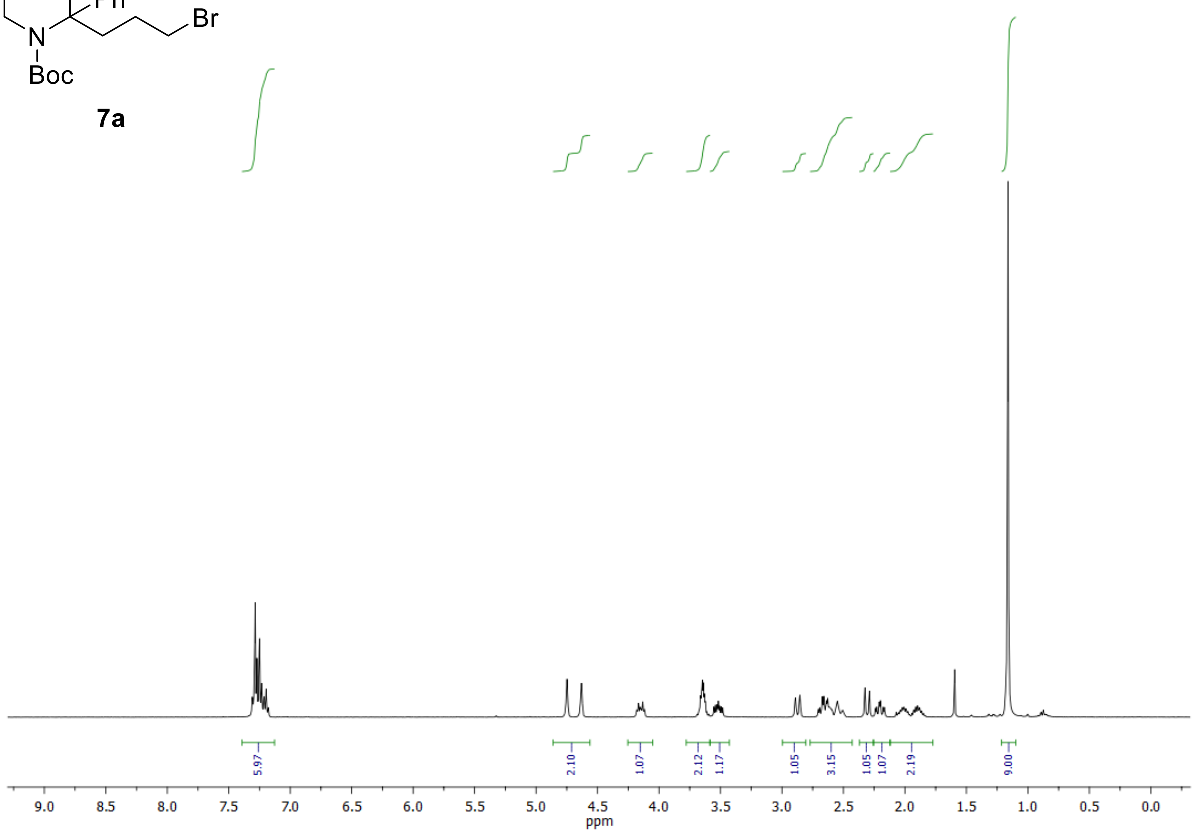

$^{13}C\{1H\}$ NMR spectrum ($CDCl_3$, 100 MHz):

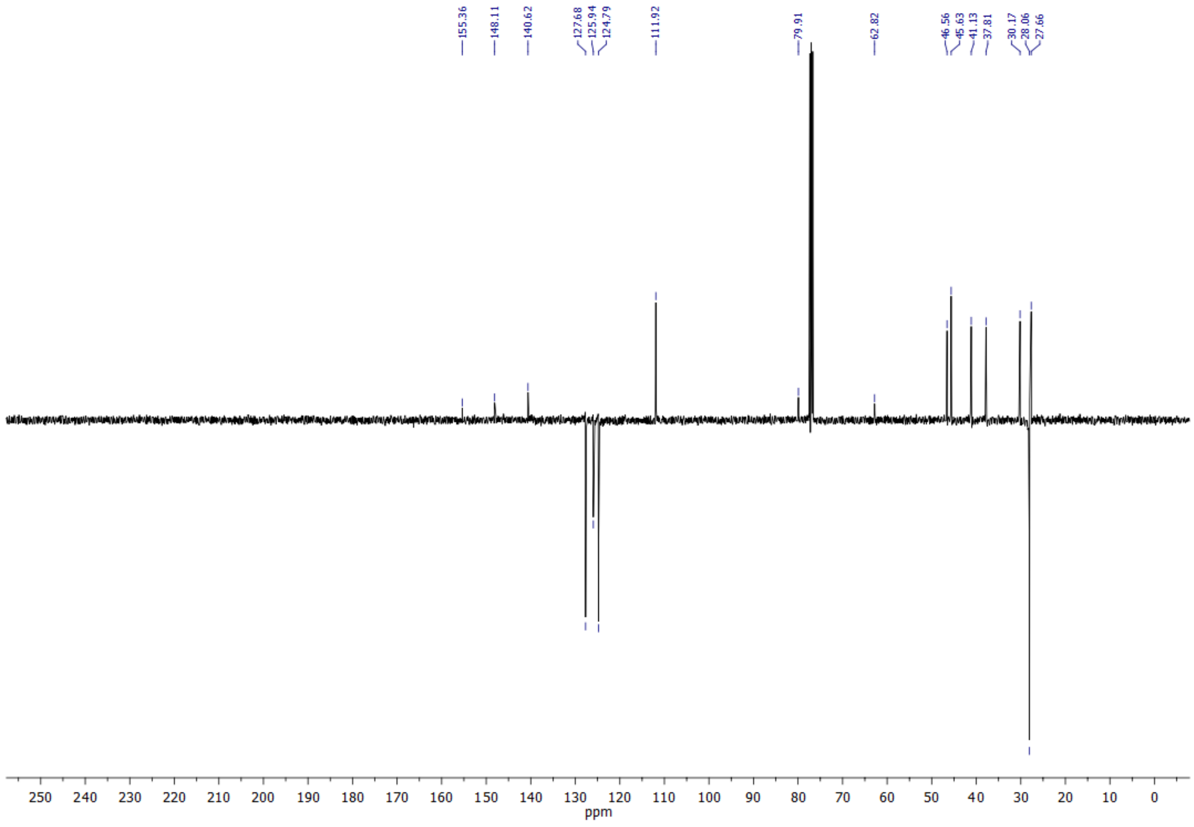

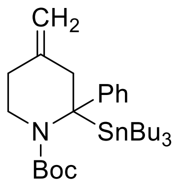

8a

^{1}H NMR spectrum ($CDCl_3$, 400 MHz):

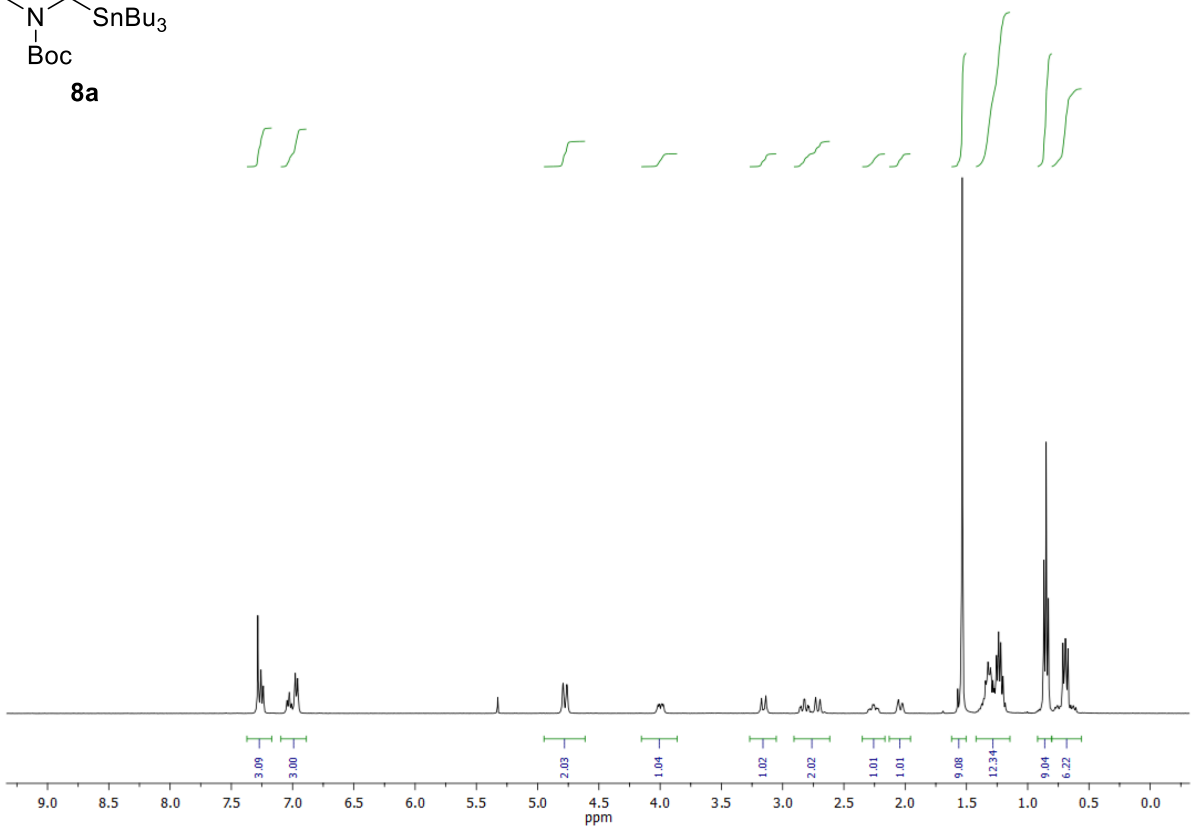

^{13}C{1H} NMR spectrum ($CDCl_3$, 100 MHz):

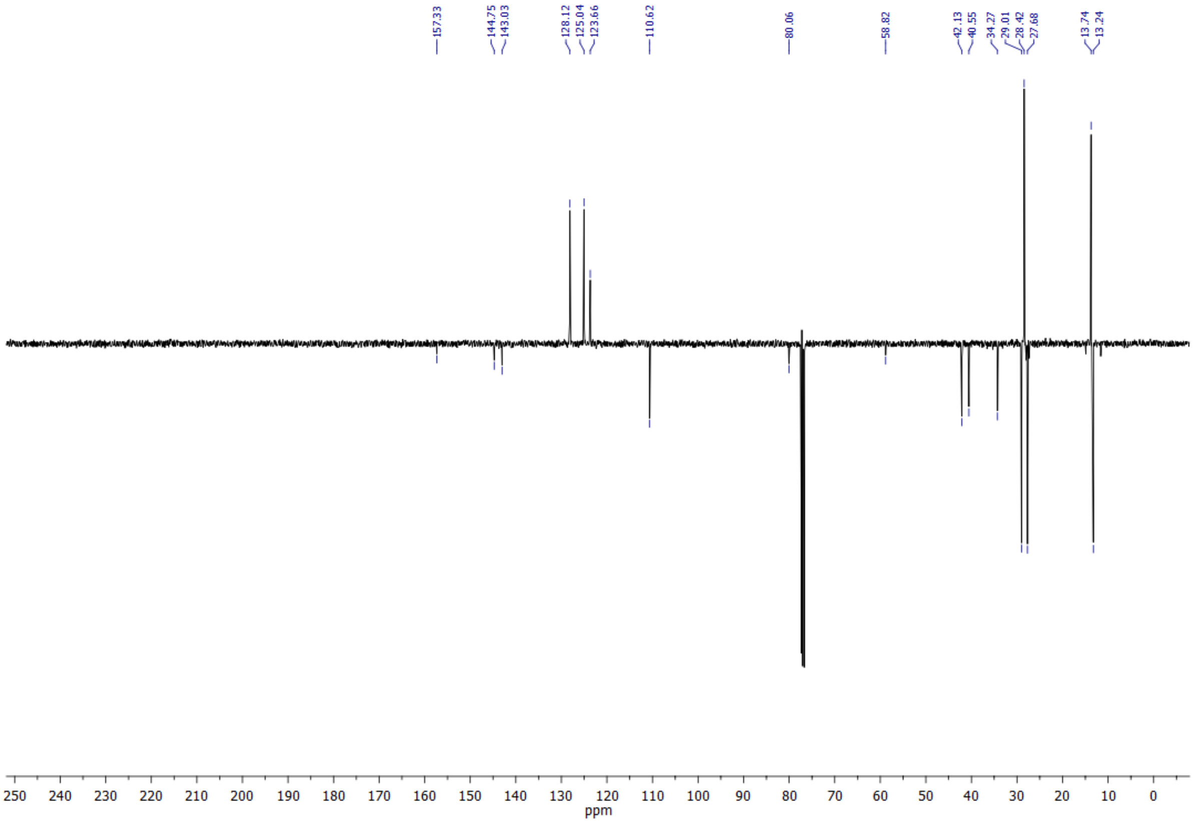

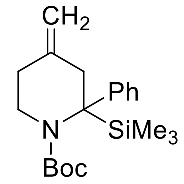

9a

^{1}H NMR spectrum ($CDCl_3$, 400 MHz):

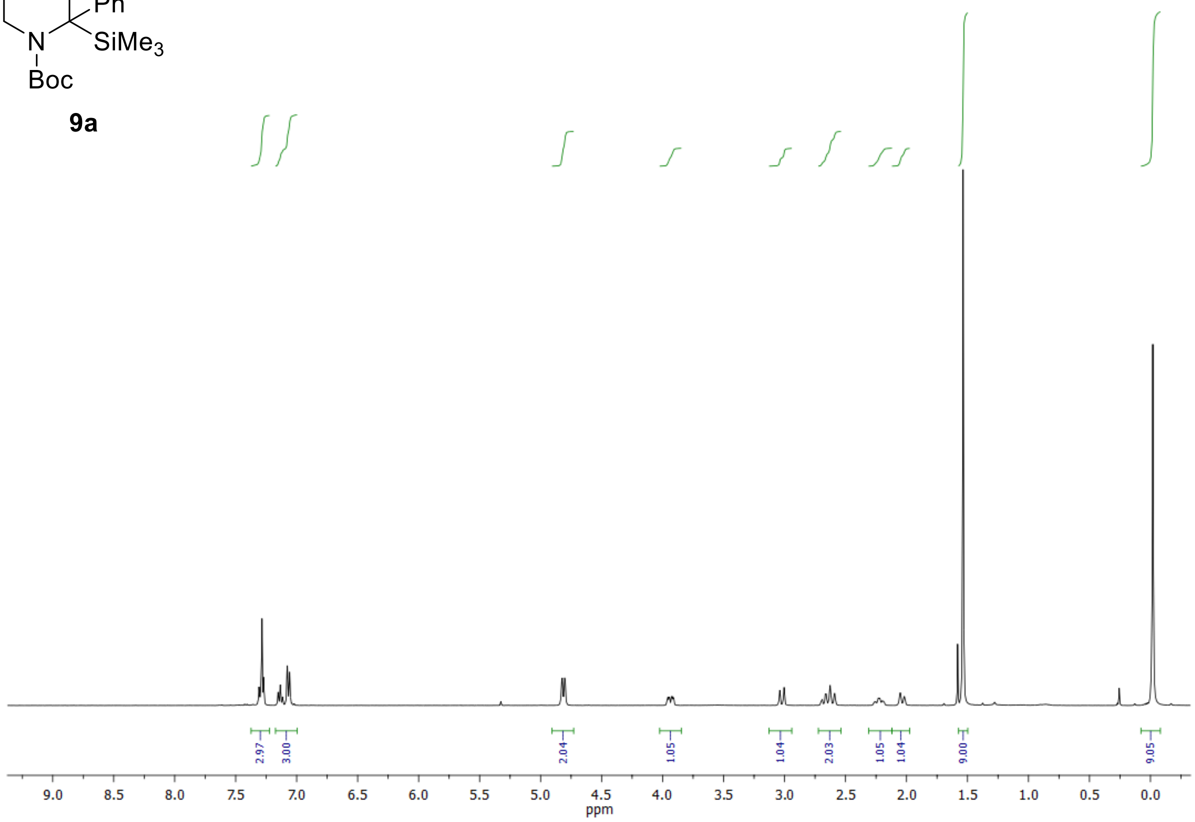

^{13}C{1H} NMR spectrum ($CDCl_3$, 100 MHz):

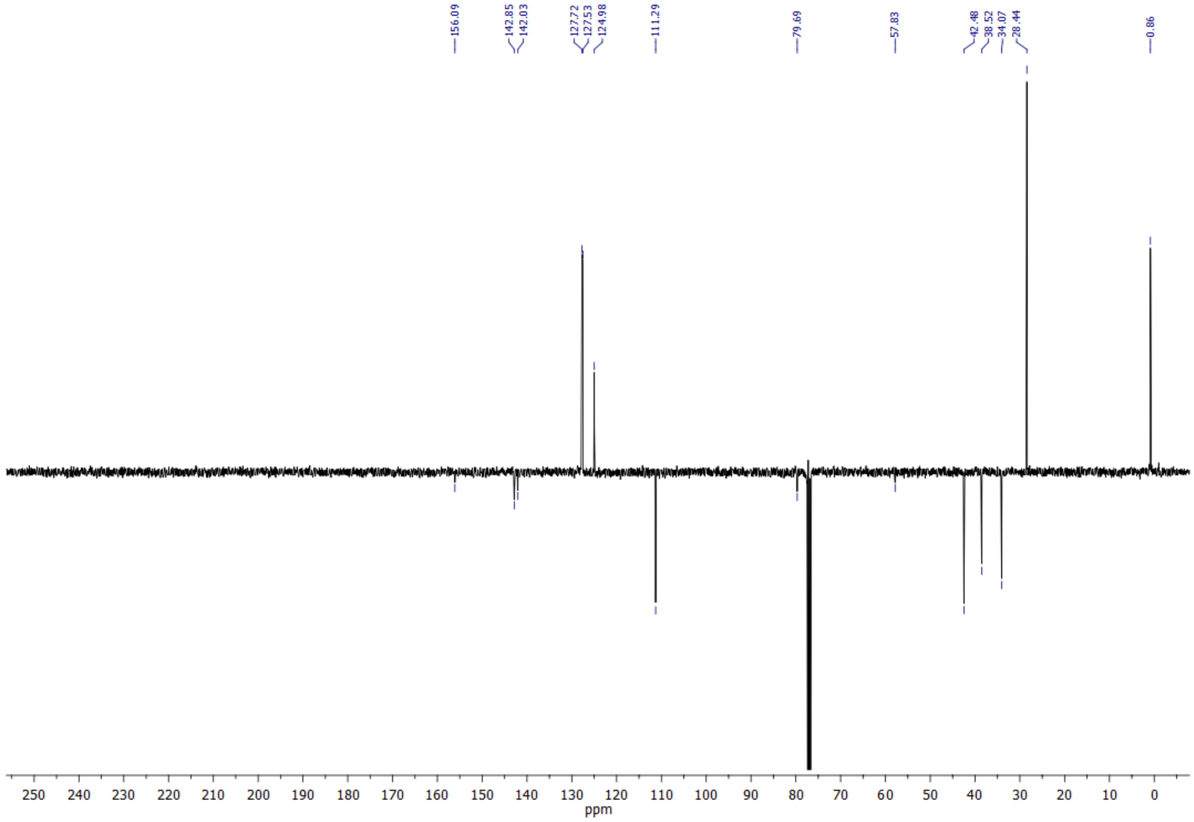

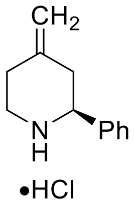

(*S*)-**10**
(in d_6-DMSO)

^{1}H NMR spectrum (d_6-DMSO, 400 MHz):

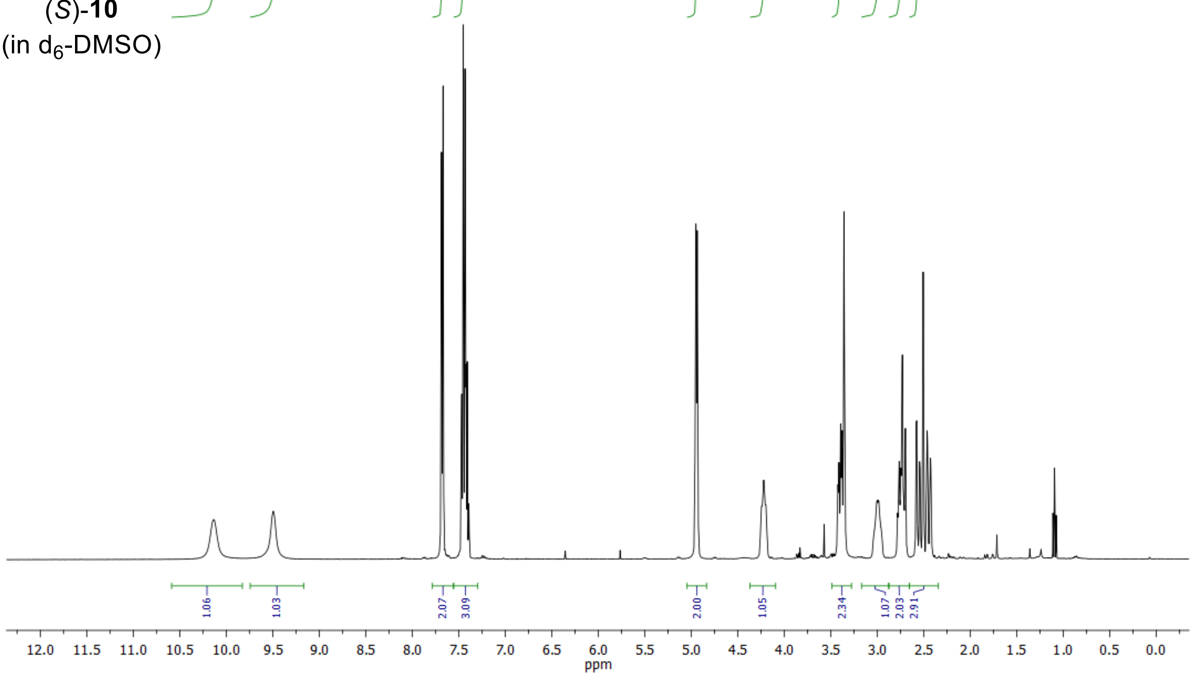

^{13}C{1H} NMR spectrum (d_6-DMSO, 100 MHz):

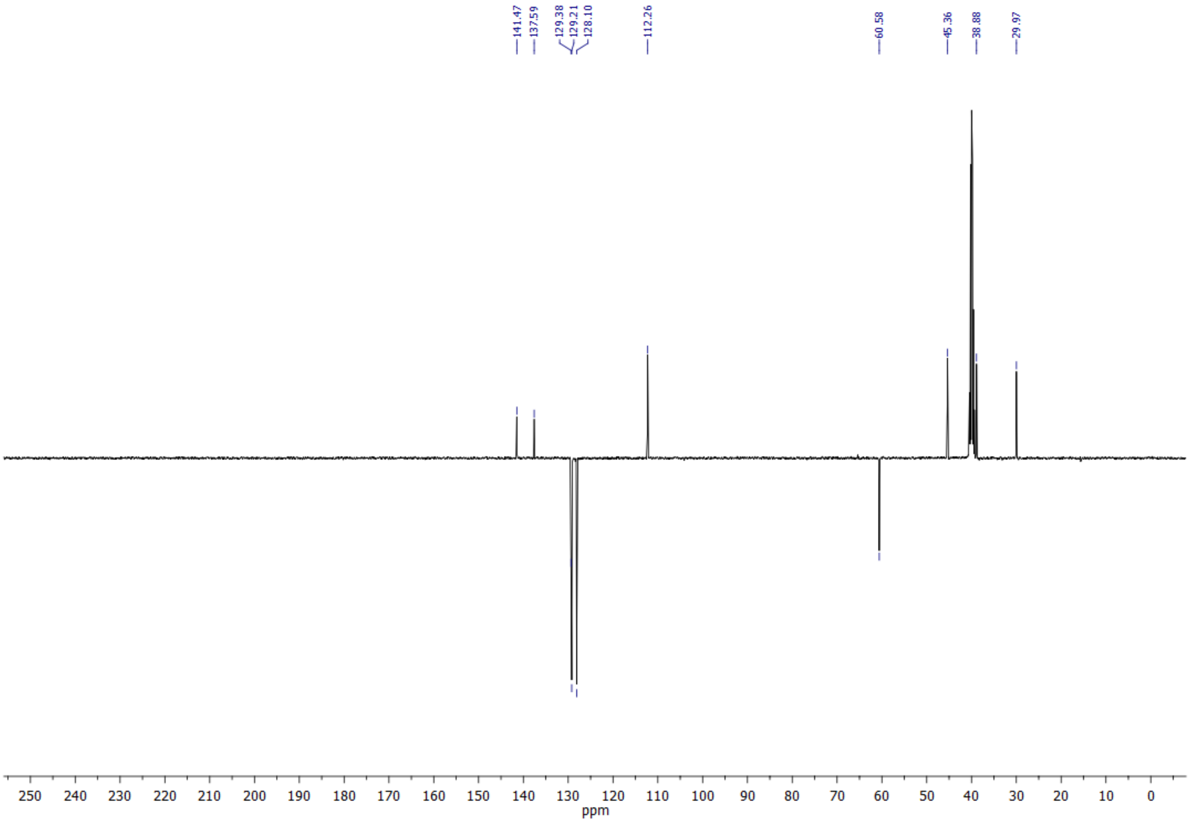

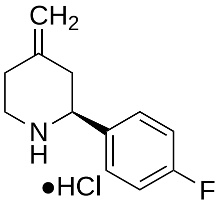

(*S*)-**11**
(in d_6-DMSO)

^{1}H NMR spectrum (d_6-DMSO, 400 MHz):

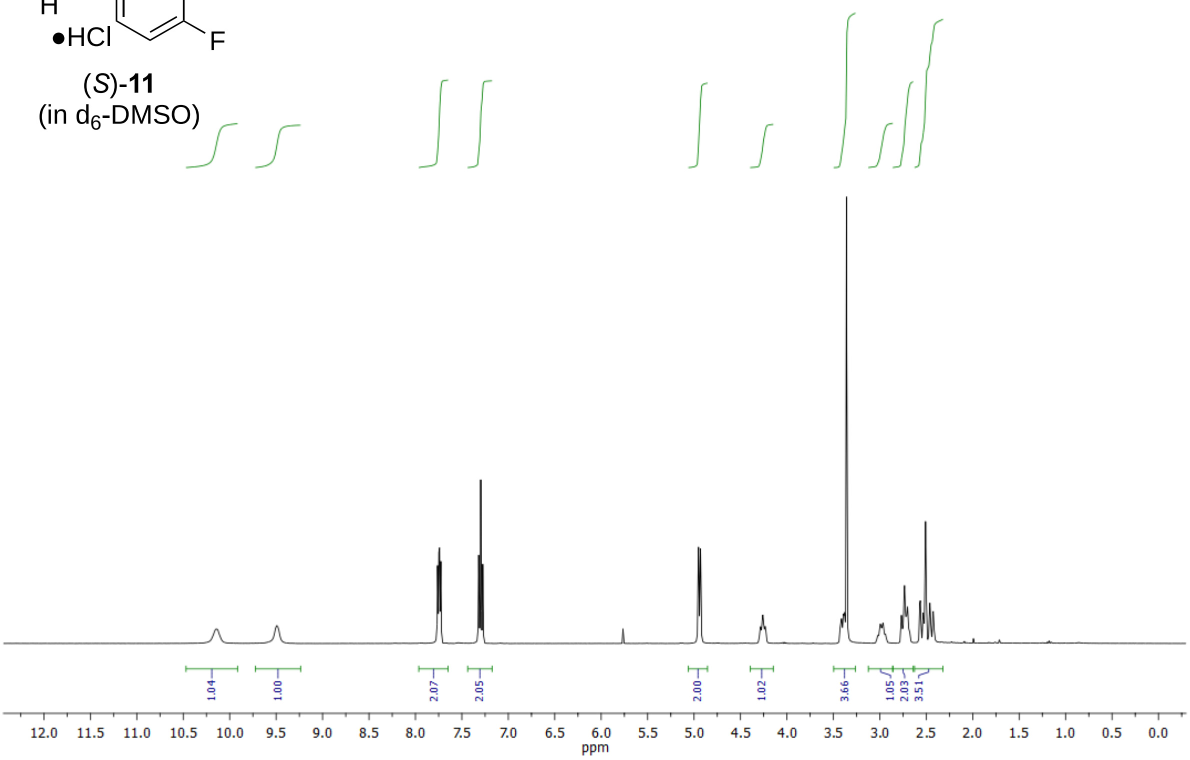

^{13}C{1H} NMR spectrum (d_6-DMSO, 100 MHz):

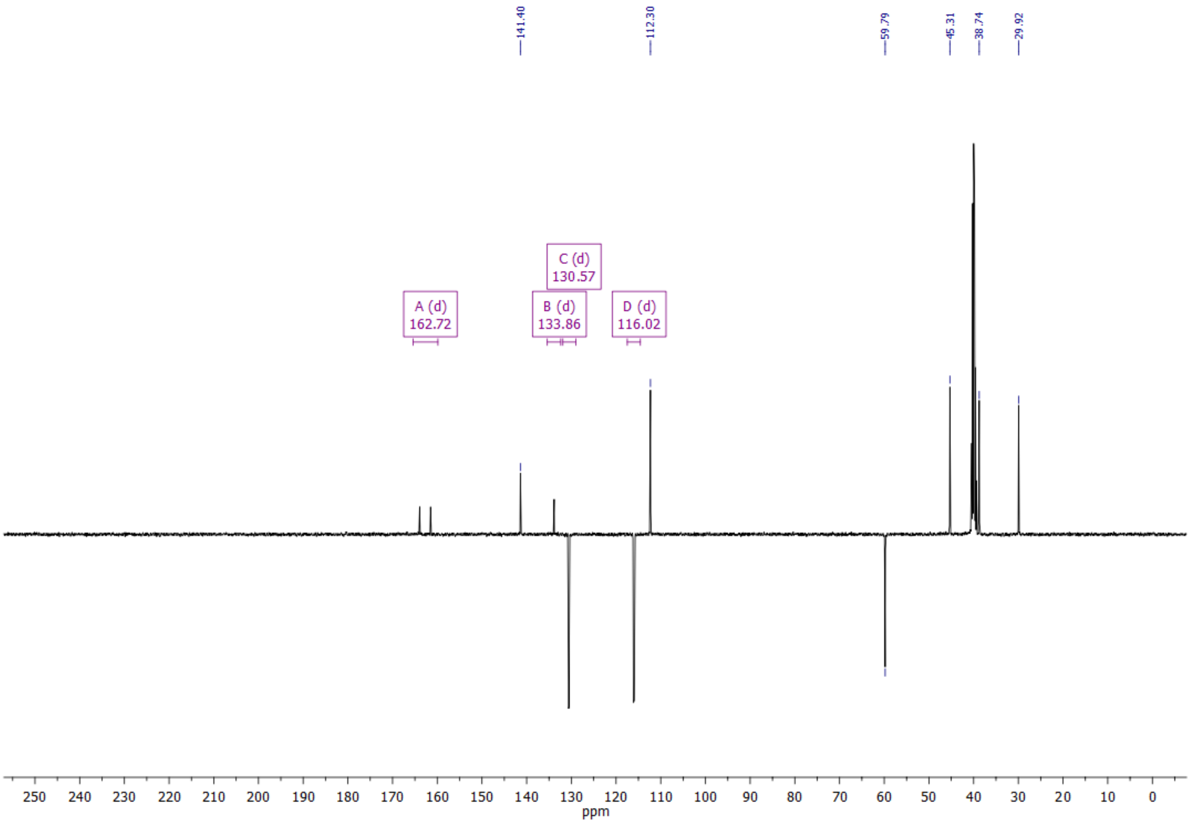

^{19}F NMR spectrum (d_6-DMSO, 377 MHz):

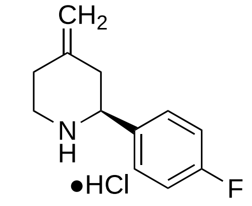

(*S*)-**11**
(^{19}F-NMR in d_6-DMSO)

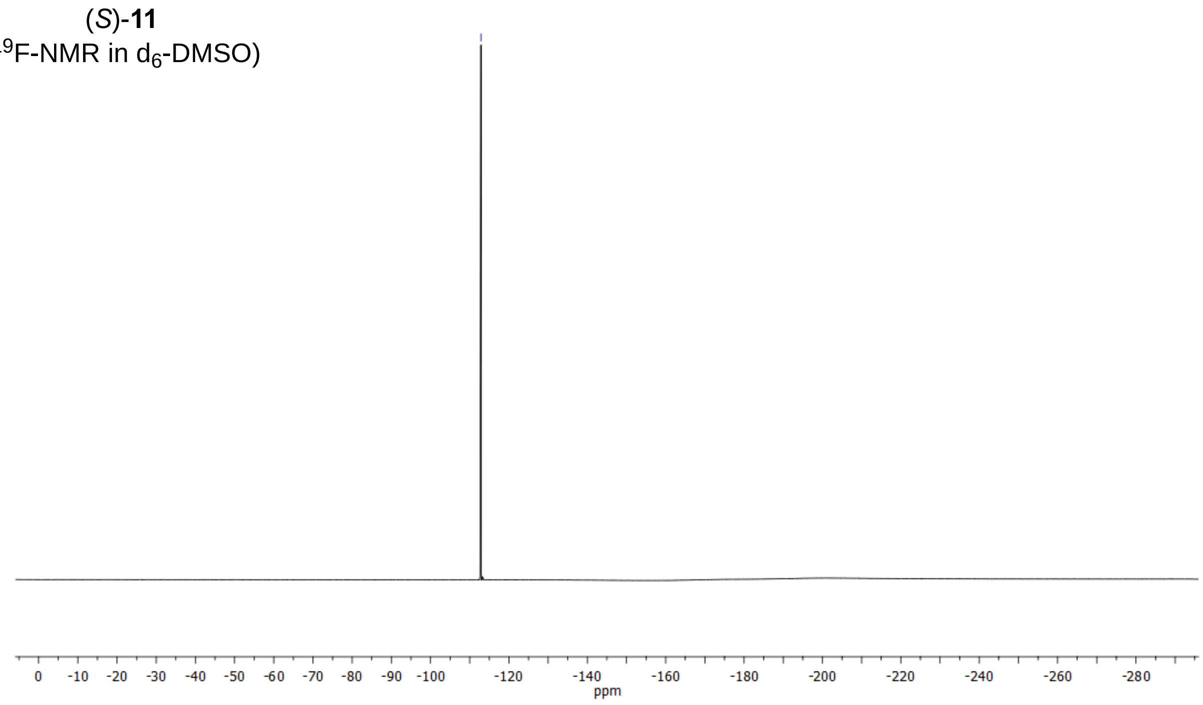

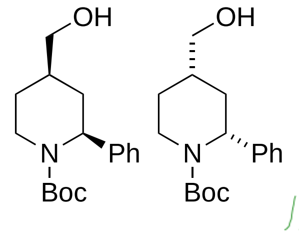

12

^{1}H NMR spectrum ($CDCl_3$, 400 MHz):

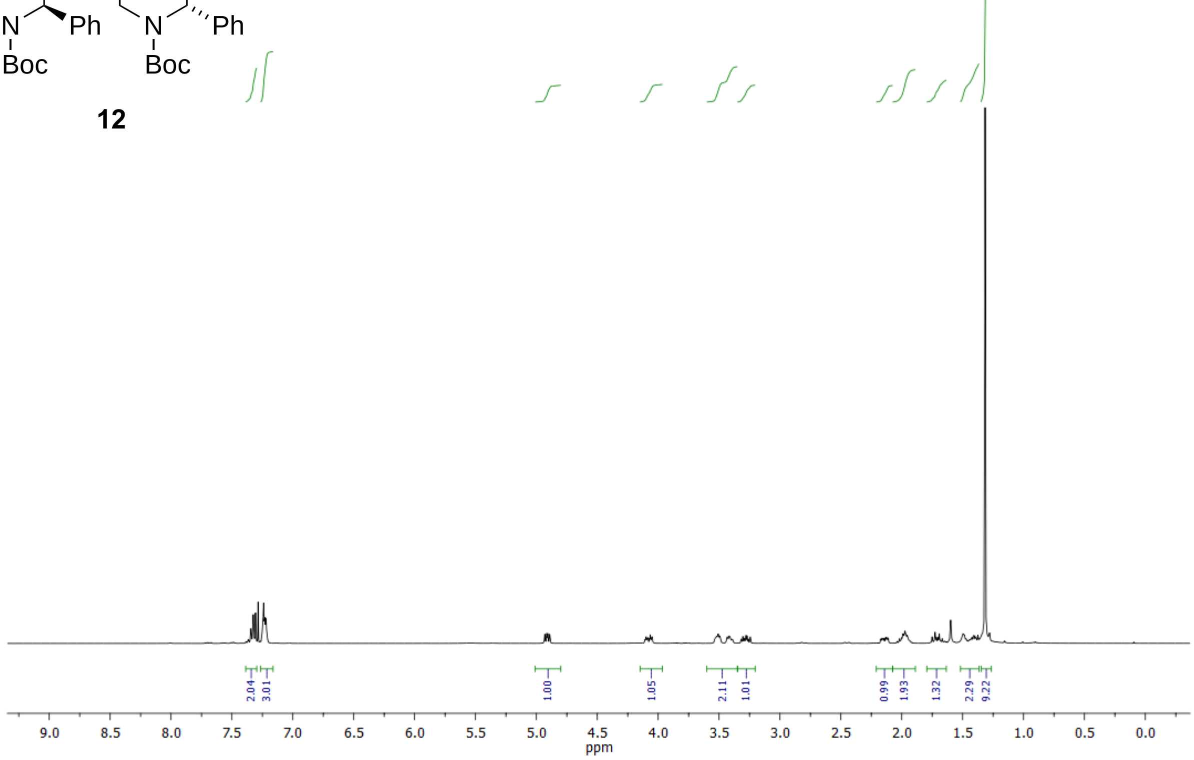

$^{13}C\{1H\}$ NMR spectrum ($CDCl_3$, 100 MHz):

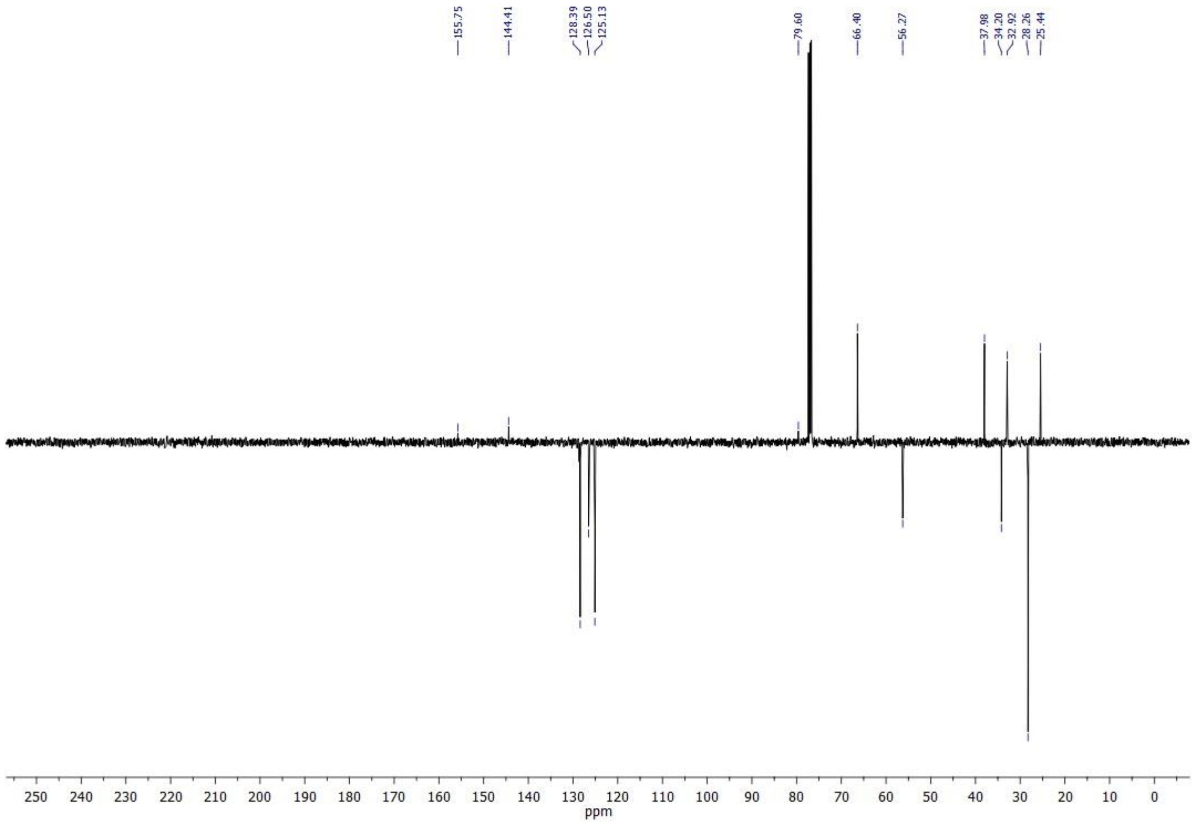

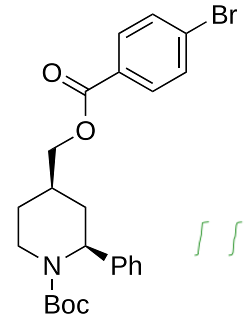

(2*S*,4*R*)-**13**

^{1}H NMR spectrum ($CDCl_3$, 400 MHz):

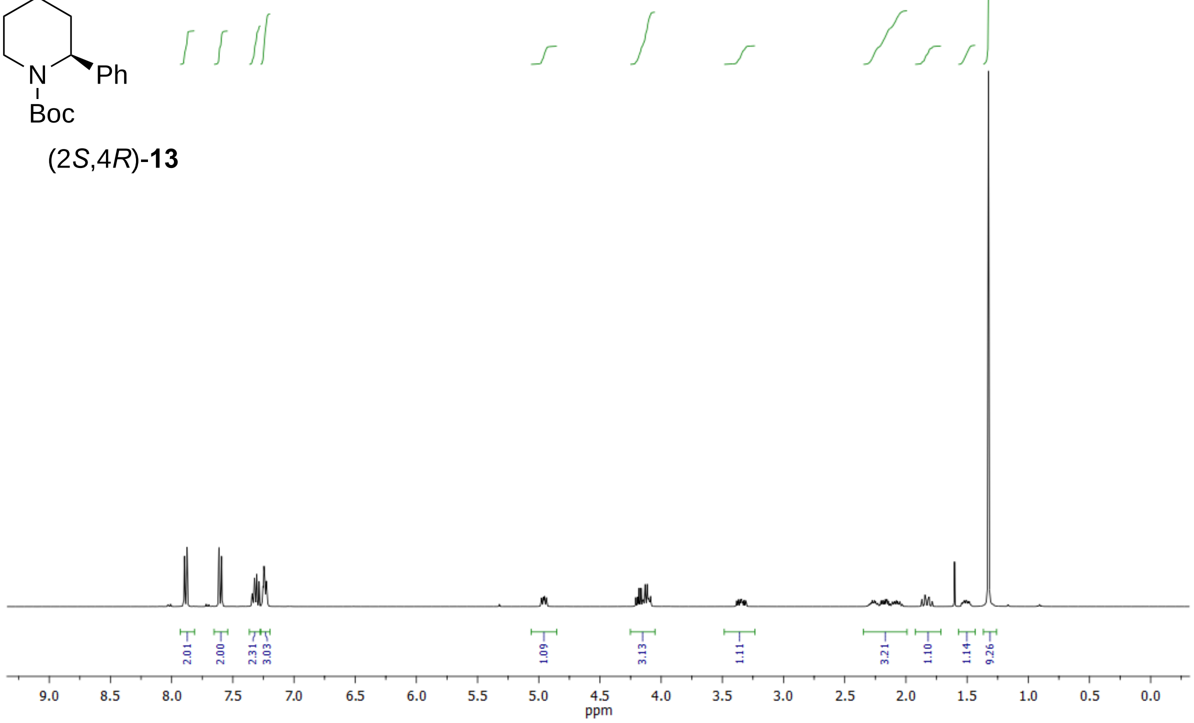

^{13}C{1H} NMR spectrum ($CDCl_3$, 100 MHz):

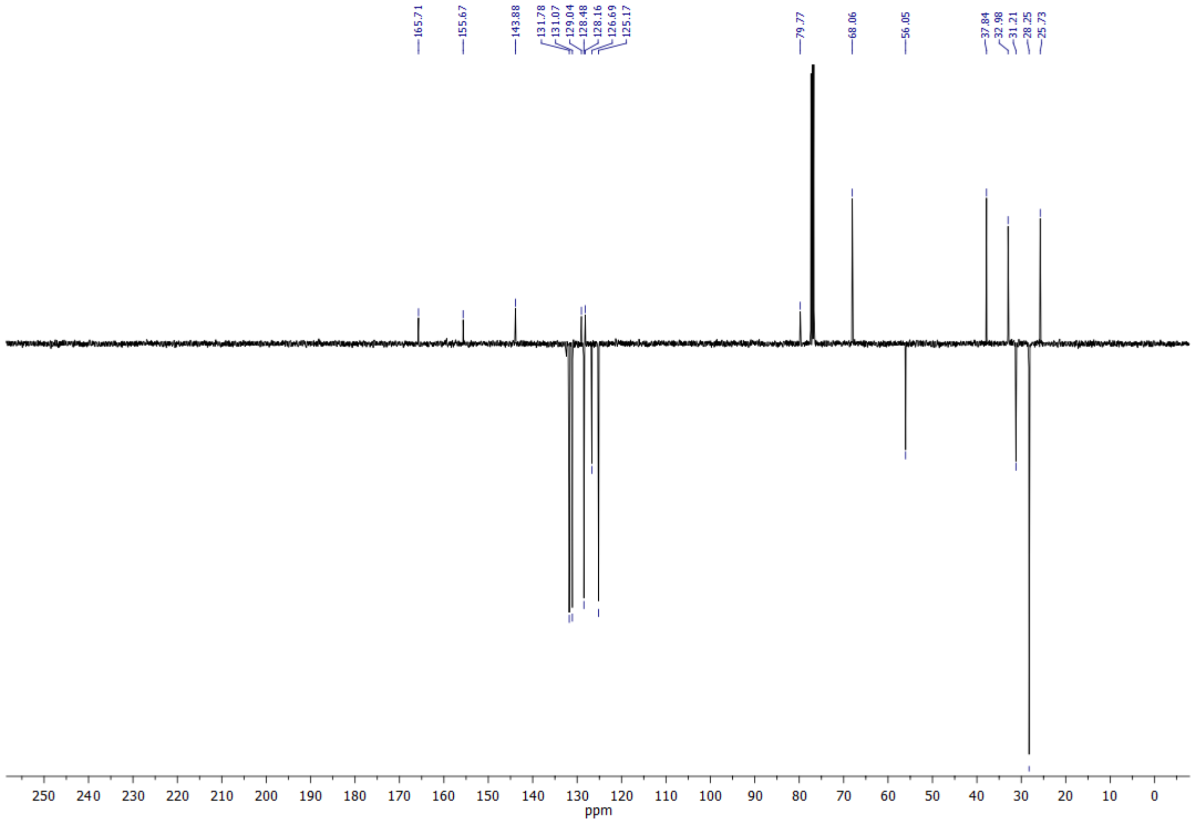

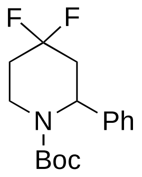

14

^{1}H NMR spectrum ($CDCl_3$, 400 MHz):

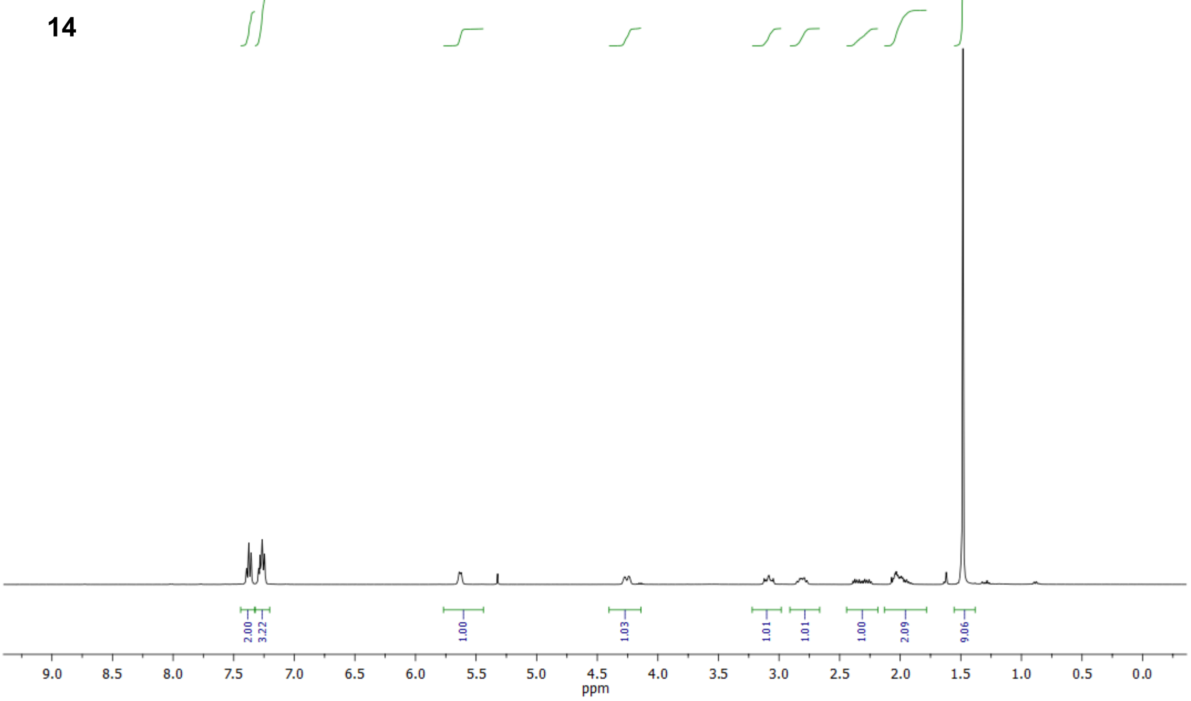

^{13}C{1H} NMR spectrum ($CDCl_3$, 100 MHz):

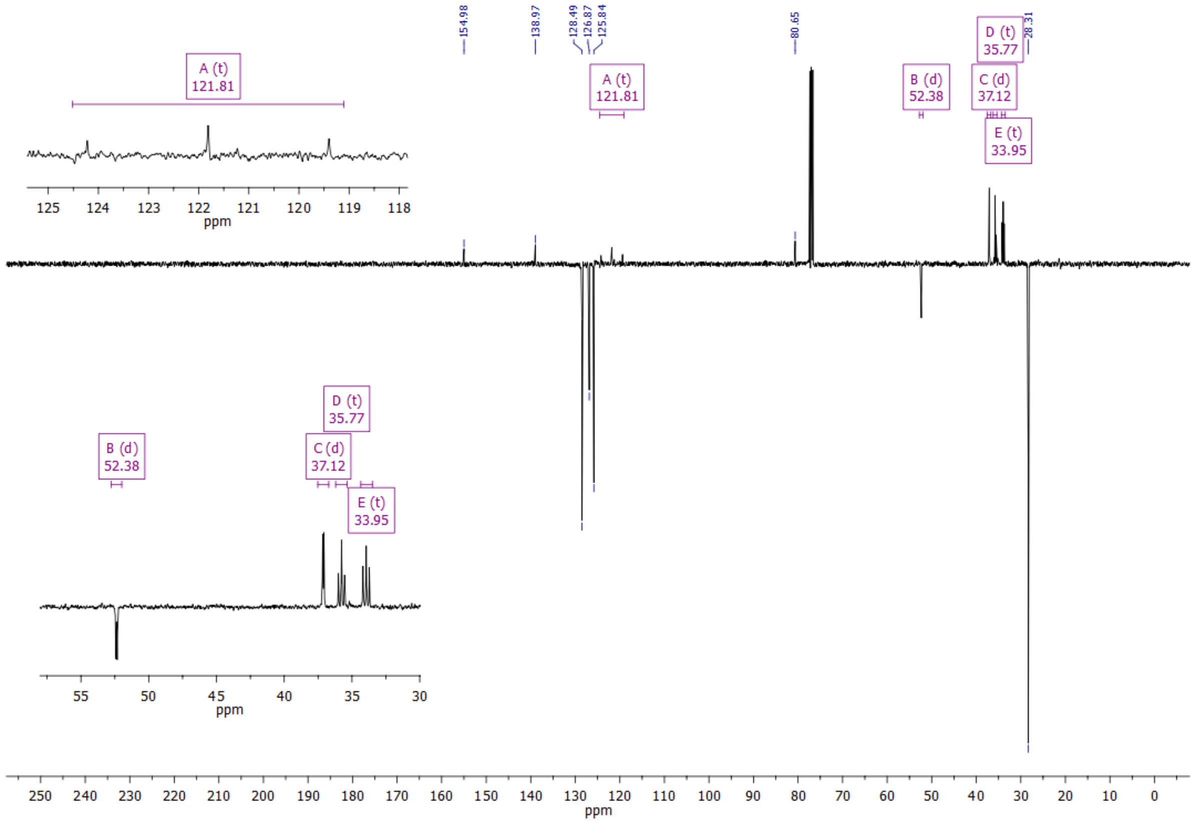

^{19}F NMR spectrum ($CDCl_3$, 377 MHz):

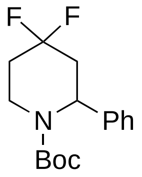

14 (^{19}F-NMR)

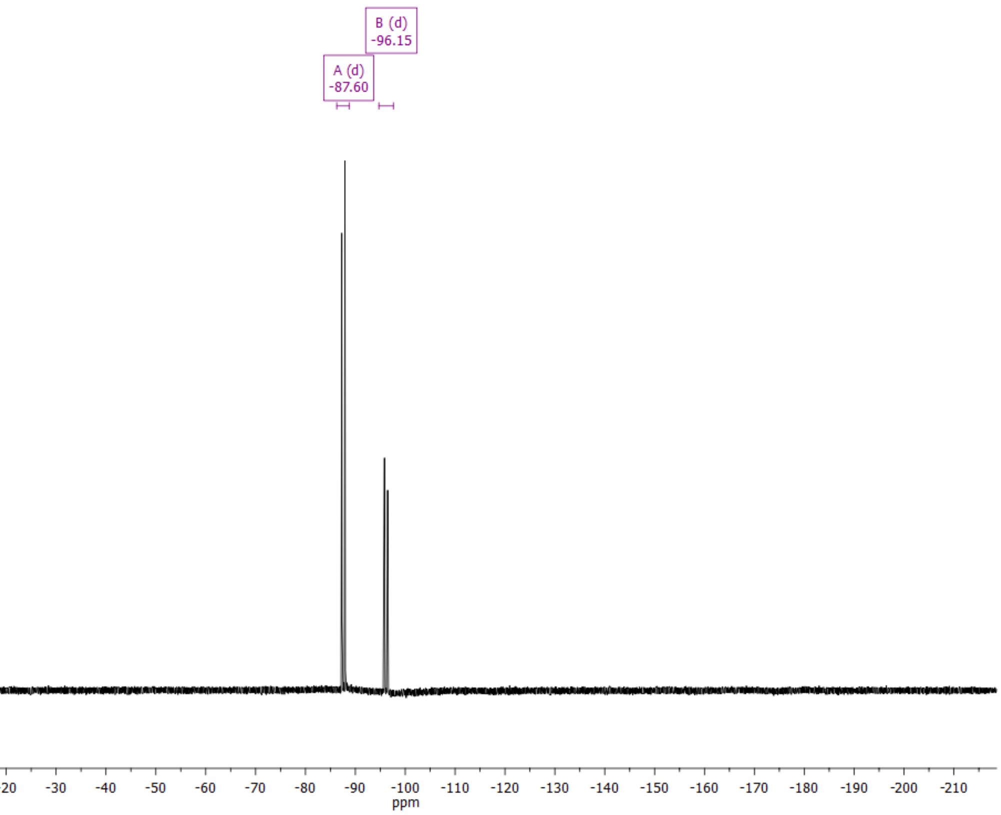

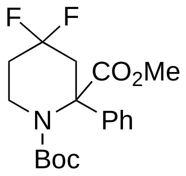

15

^{1}H NMR spectrum ($CDCl_3$, 400 MHz):

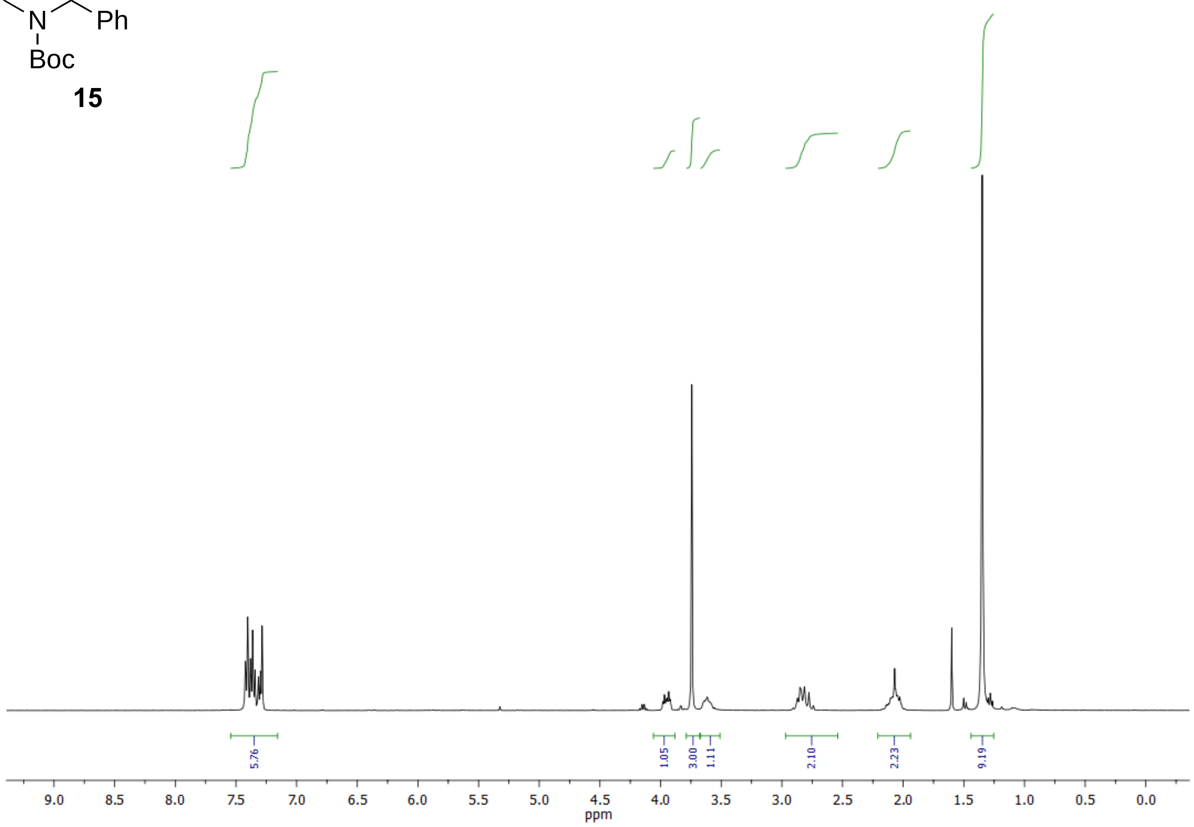

^{13}C{1H} NMR spectrum ($CDCl_3$, 100 MHz):

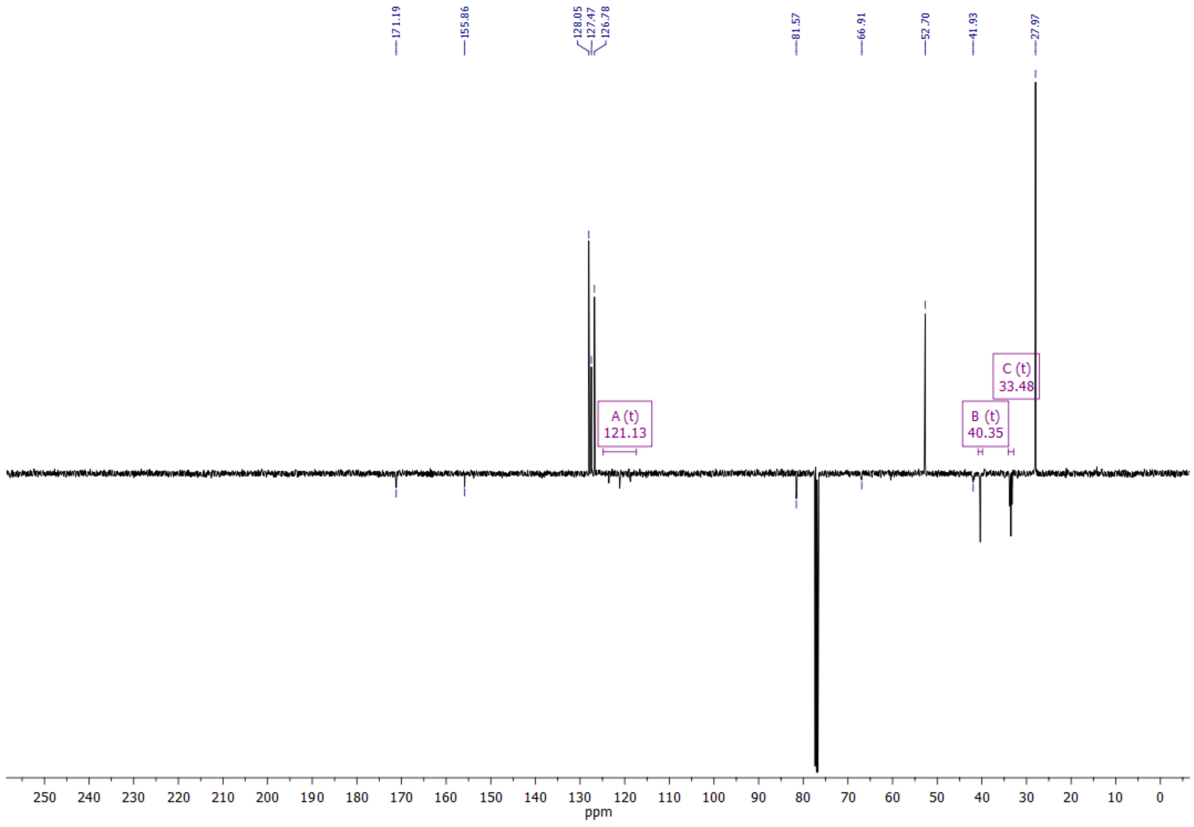

^{19}F NMR spectrum ($CDCl_3$, 377 MHz):

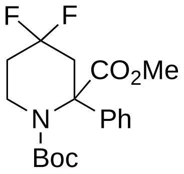

15 (^{19}F-NMR)

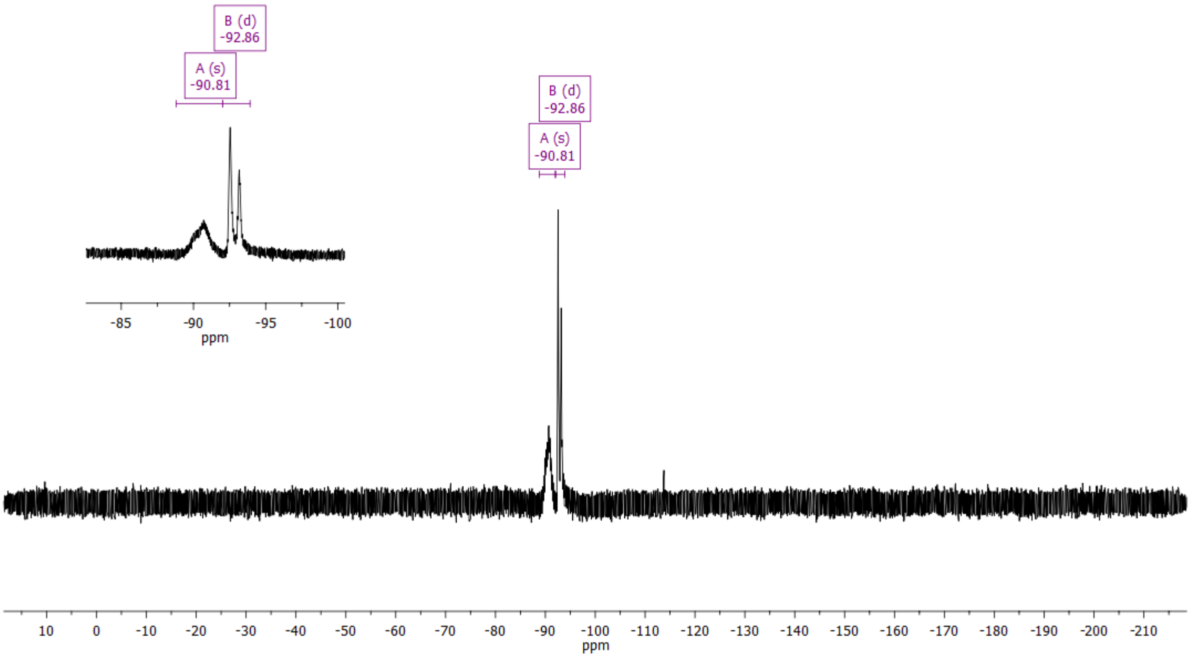

5. HPLC traces

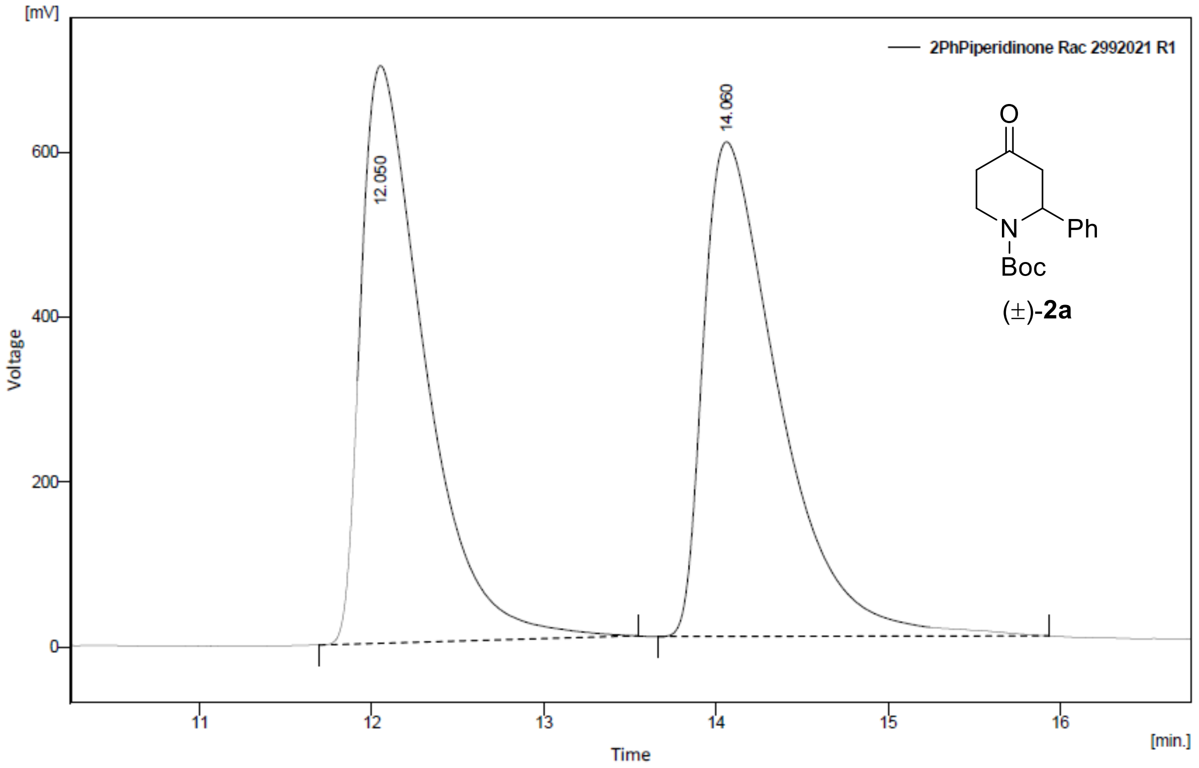

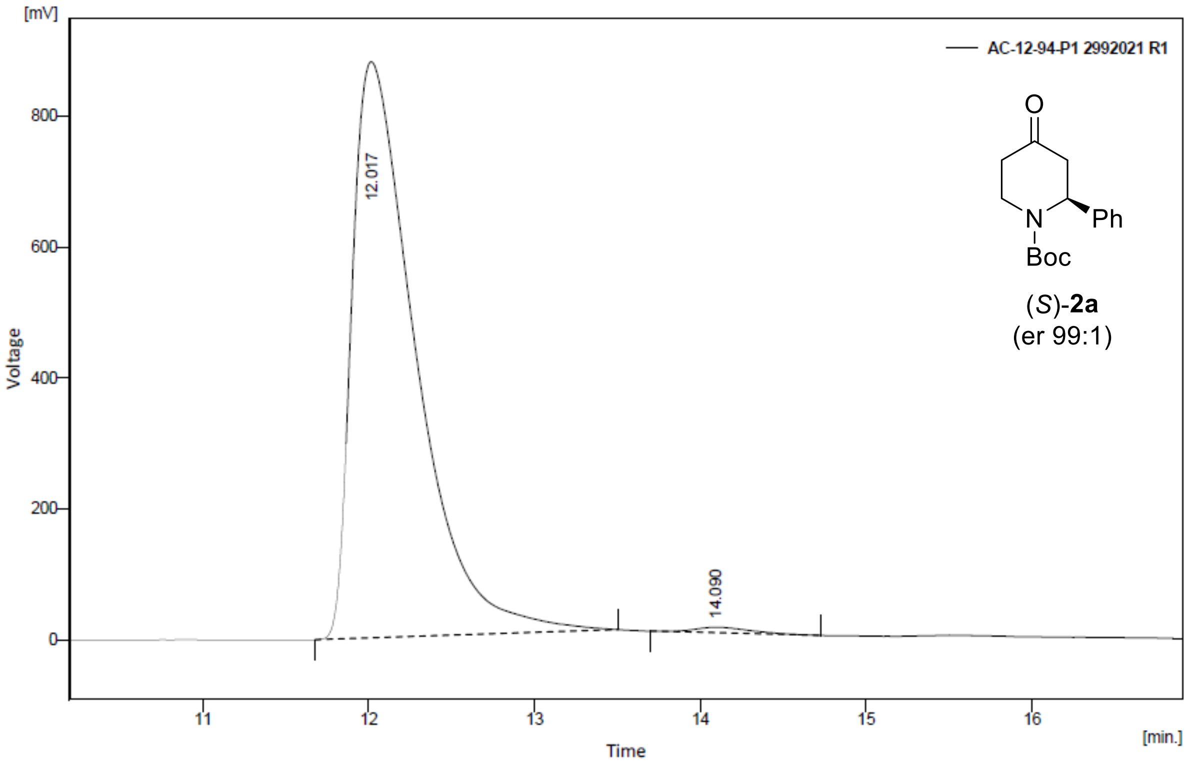

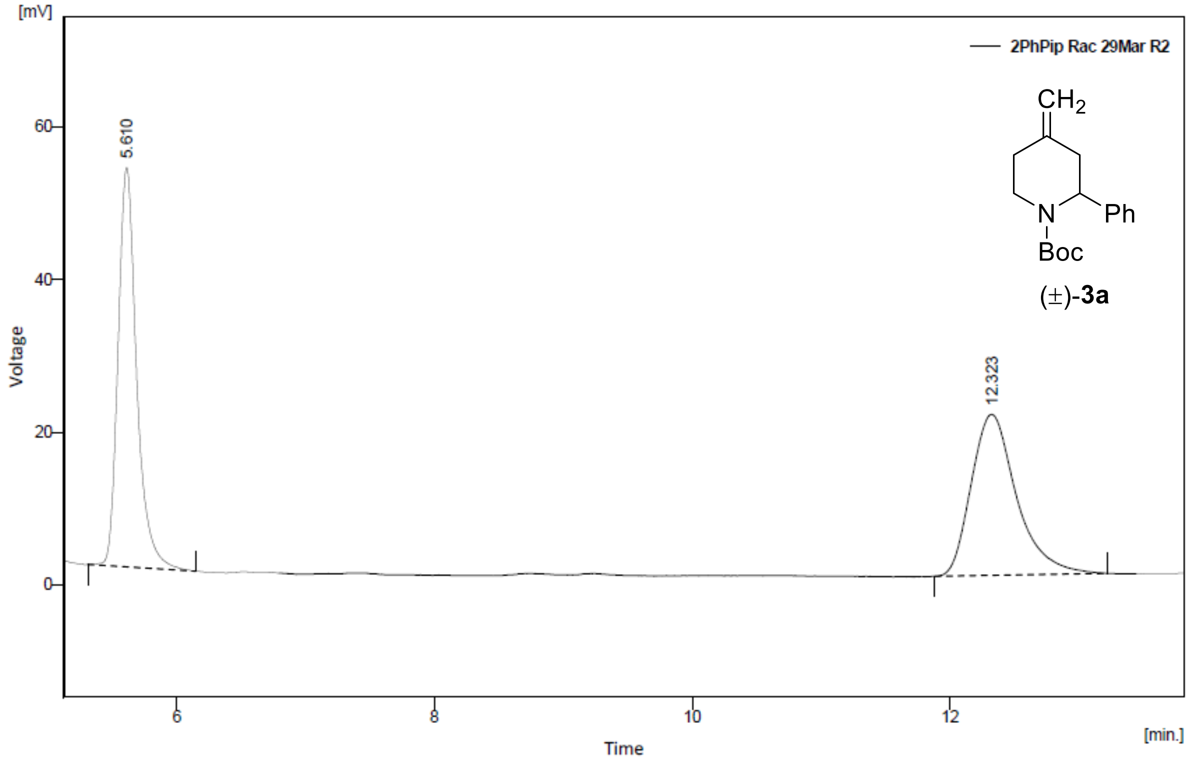
[mV]
2PhPip Rac 29Mar R2
60
40
20
0
Voltage
5.610
12.323
6
8
10
12
[min.]
Time
CH2
N
Ph
Boc
(±)-3a

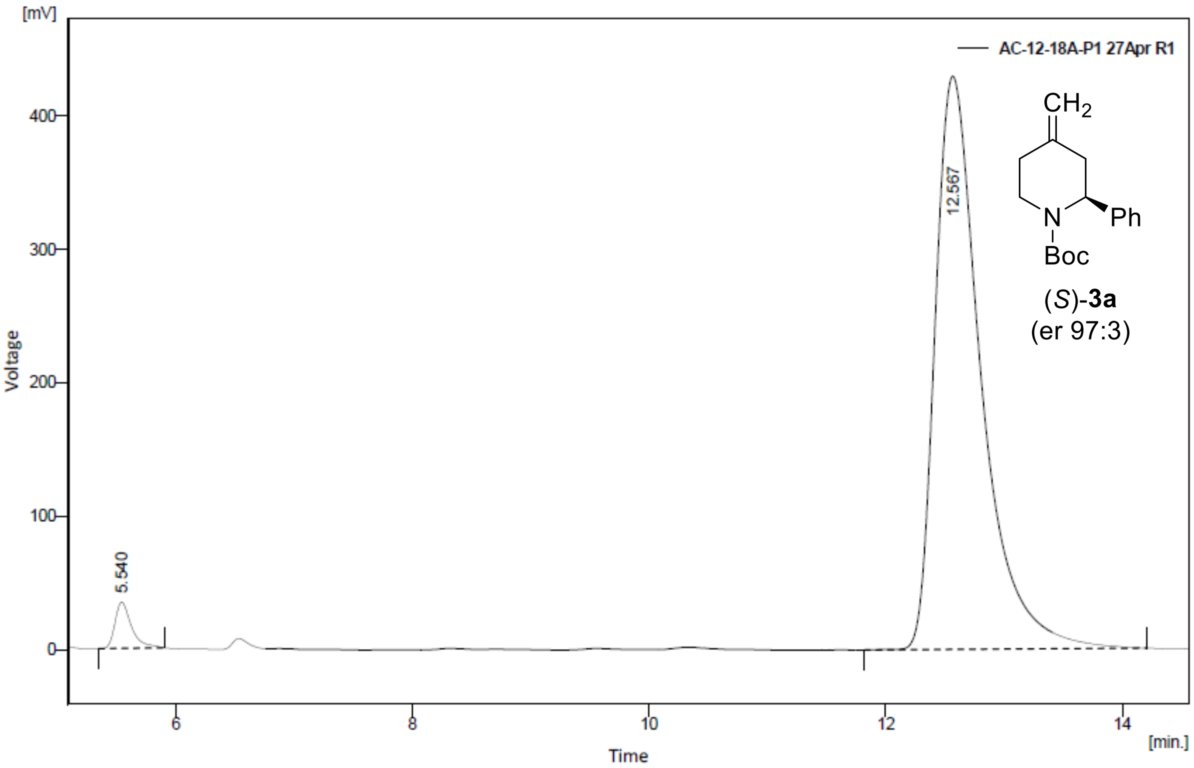
[mV]
AC-12-18A-P1 27Apr R1
400
300
200
100
0
Voltage
5.540
12.567
6
8
10
12
14
[min.]
Time
CH2
N
Ph
Boc
(S)-3a
(er 97:3)

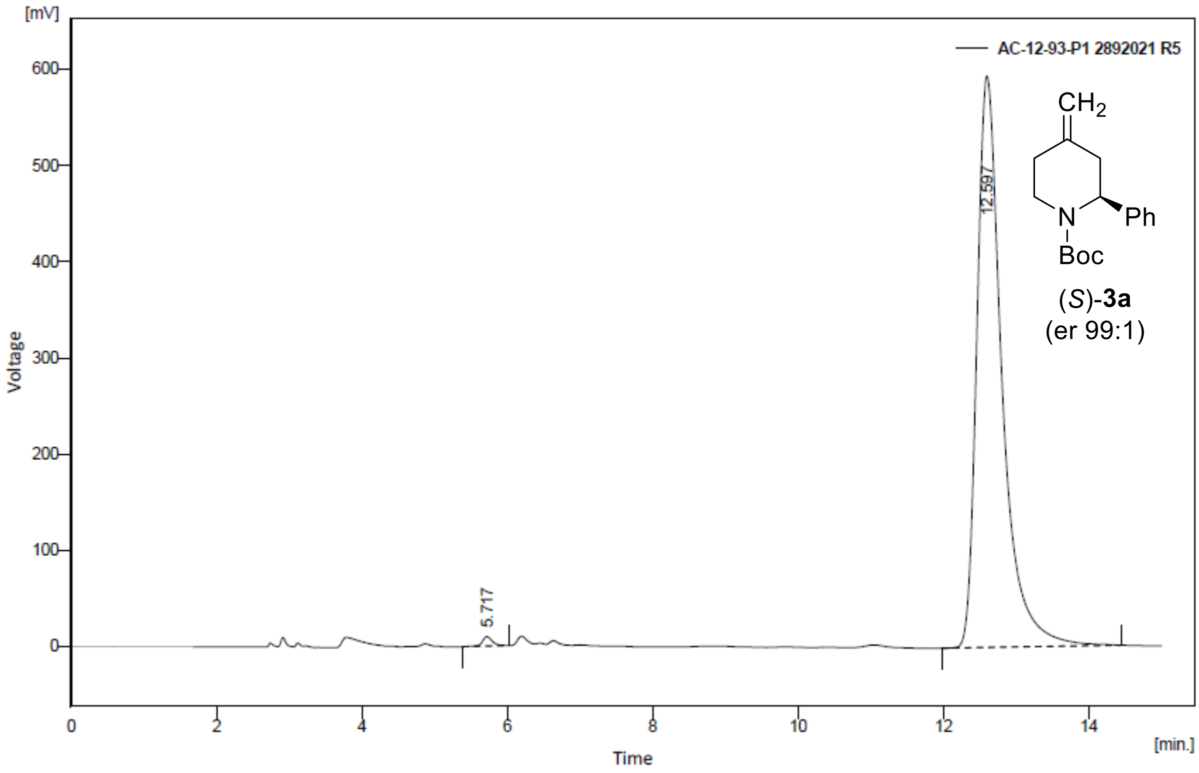
[mV]
600
500
400
300
200
100
0
Voltage
AC-12-93-P1 2892021 R5
12.597
5.717
0
2
4
6
8
10
12
14
[min.]
Time
CH2
N
Ph
Boc
(S)-3a
(er 99:1)

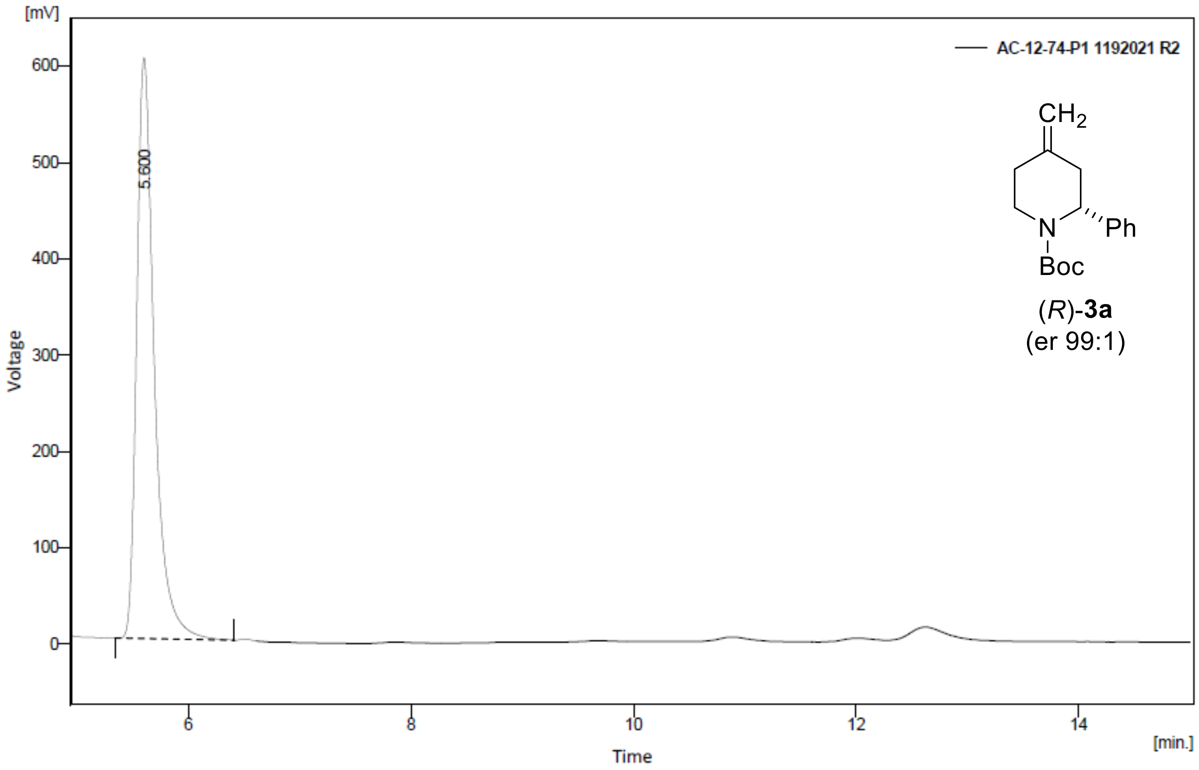
[mV]
600
500
400
300
200
100
0
Voltage
AC-12-74-P1 1192021 R2
5.600
6
8
10
12
14
[min.]
Time
CH2
N
Ph
Boc
(R)-3a
(er 99:1)

HPLC of (±)-**3b** with a Phenomenex Cellulose-2 column:

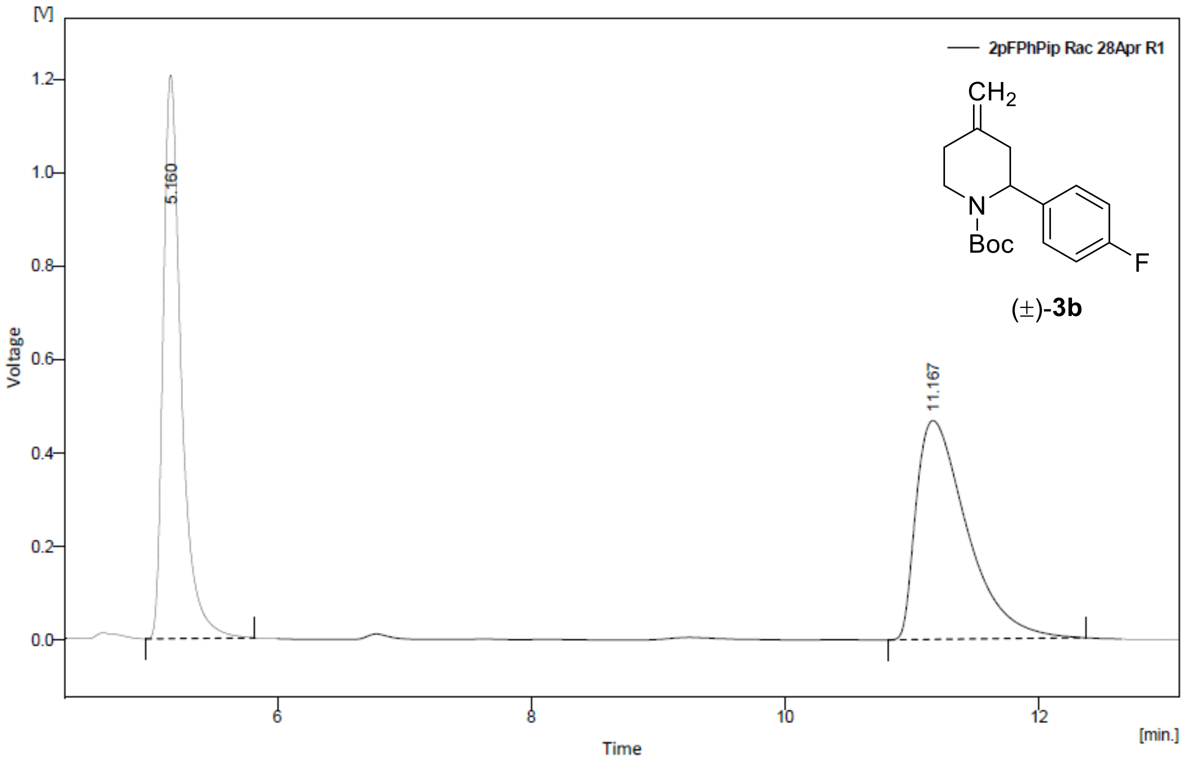

HPLC of (±)-**3b** from Agilent system fitted with a Chiralcel OX-H column:

Chiralcel OX-H Column			
	R_f/min	Area/mAU.s	Area/%
1	5.368	22318.85	48.57
2	6.028	23632.81	51.43
	Total	45951.66	100.00

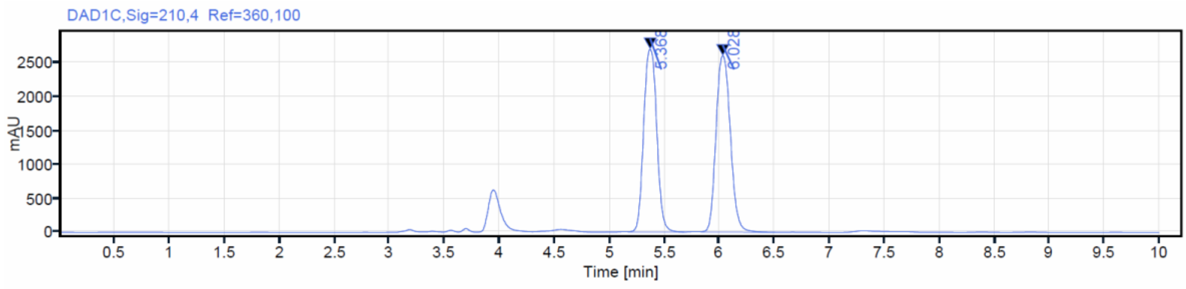

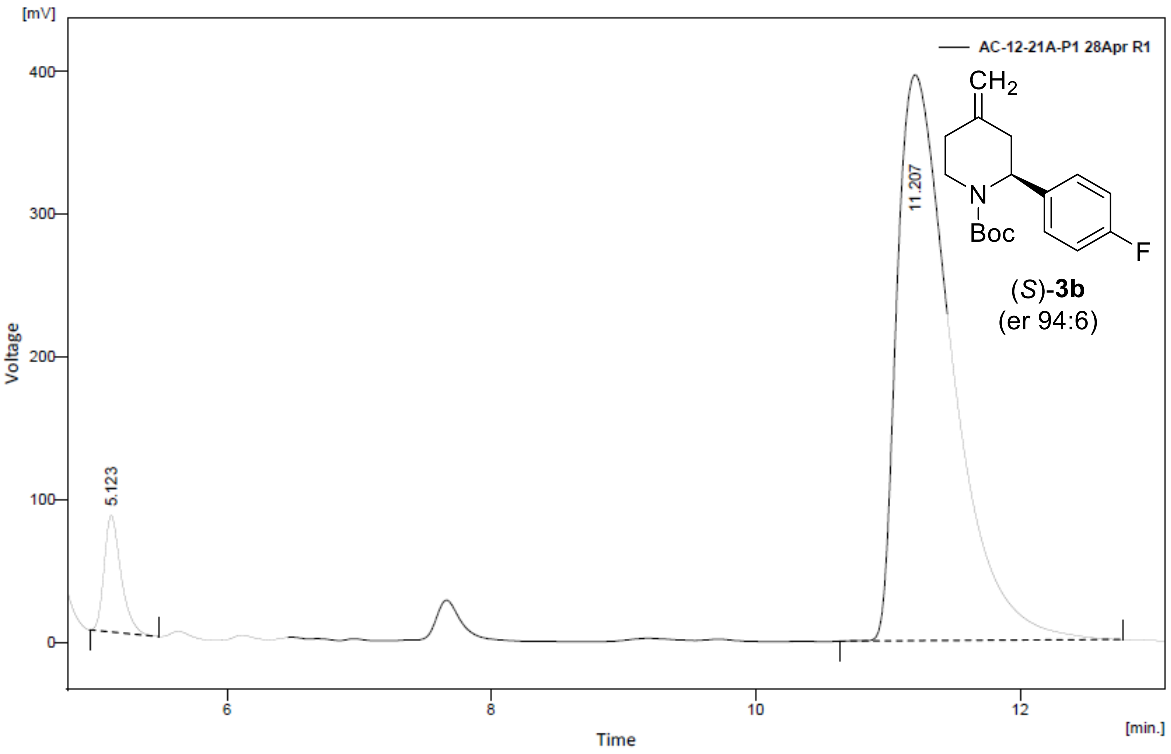

HPLC of (*S*)-**3b** (er 94:6) from Agilent system fitted with a Chiralcel OX-H column:

Chiralcel OX-H Column			
[redacted]			
	R_f/min	Area/mAU.s	Area/%
1	5.356	1818.44	6.33
2	6.068	26888.92	93.67
	Total	28707.36	100.00

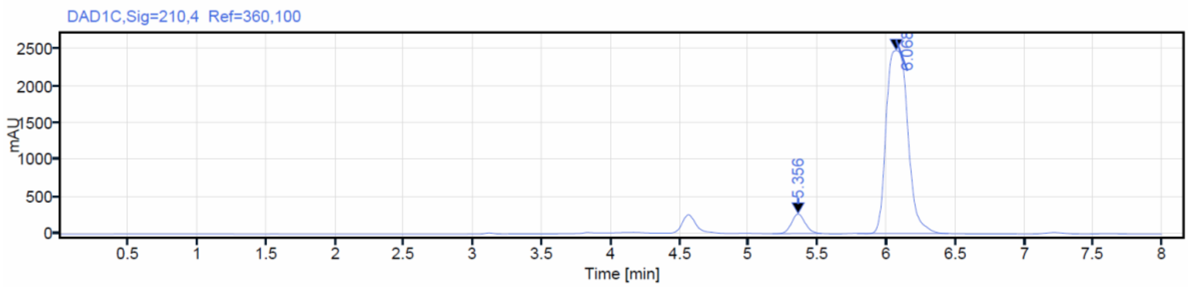

HPLC of (*S*)-**3b** (er 99:1) from Agilent system fitted with a Chiralcel OX-H column:

Chiralcel OX-H Column			
[redacted]			
	R_f/min	Area/mAU.s	Area/%
1	6.087	357.89	0.97
2	6.926	36630.58	99.03
	Total	36988.47	100.00

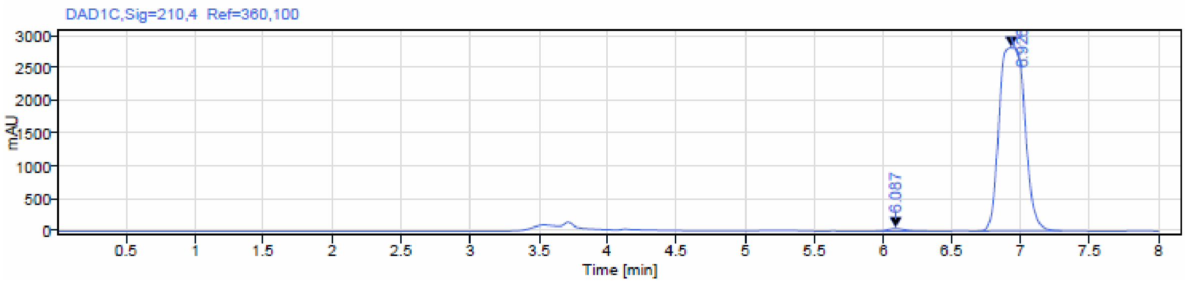

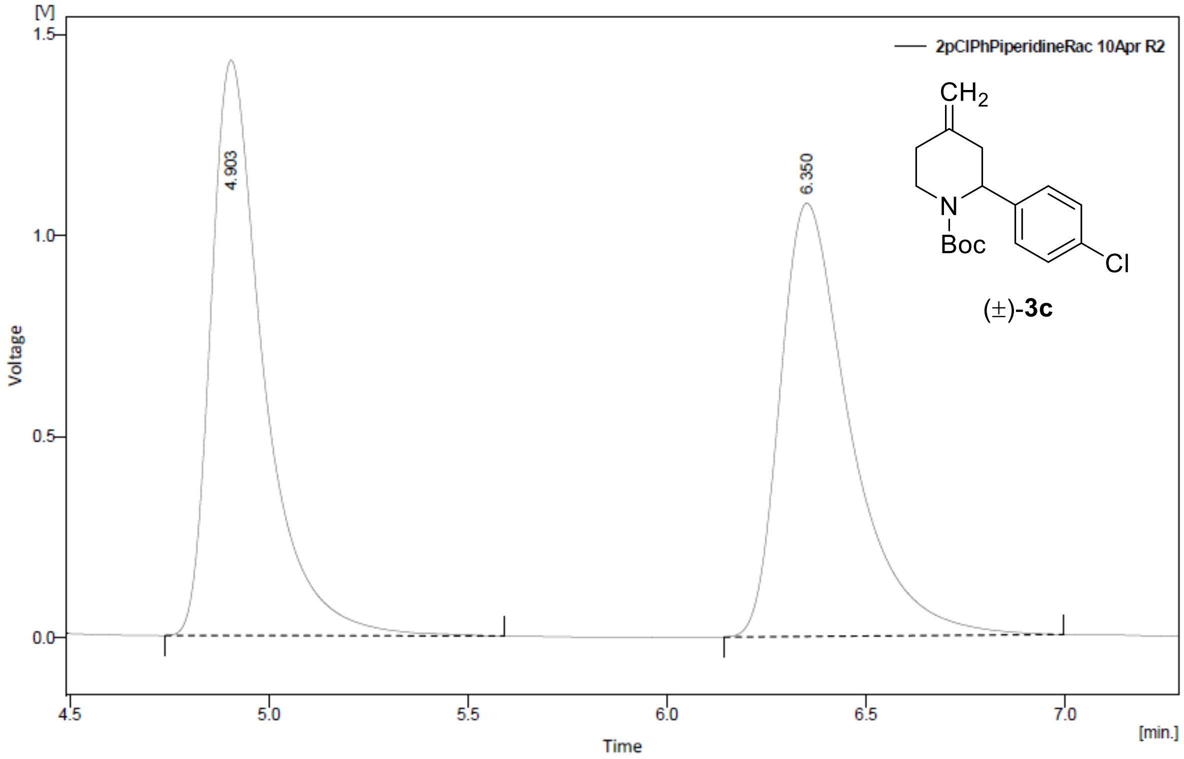

[V]
1.5
1.0
0.5
0.0
Voltage
4.903
6.350
4.5
5.0
5.5
6.0
6.5
7.0
[min.]
Time
2pClPhPiperidineRac 10Apr R2
CH2
N
Boc
Cl
(±)-3c

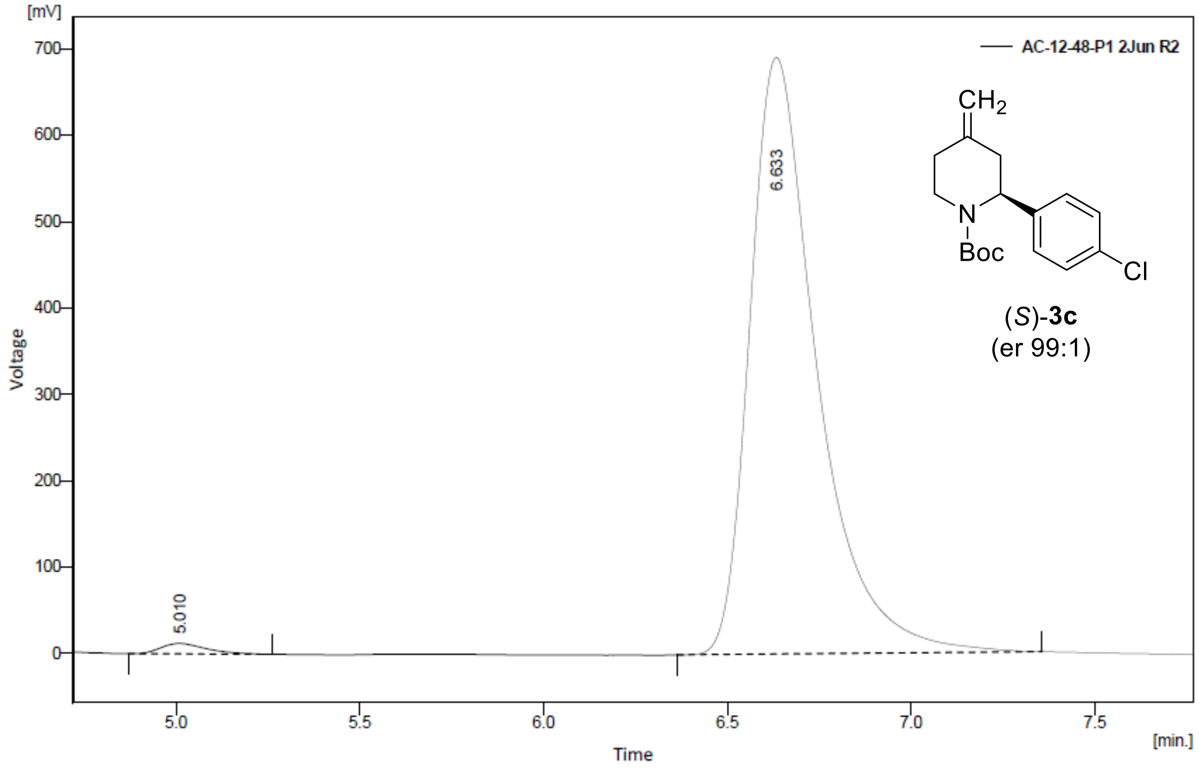

[mV]
700
600
500
400
300
200
100
0
Voltage
5.010
6.633
5.0
5.5
6.0
6.5
7.0
7.5
[min.]
Time
AC-12-48-P1 2Jun R2
CH2
N
Boc
Cl
(S)-3c
(er 99:1)

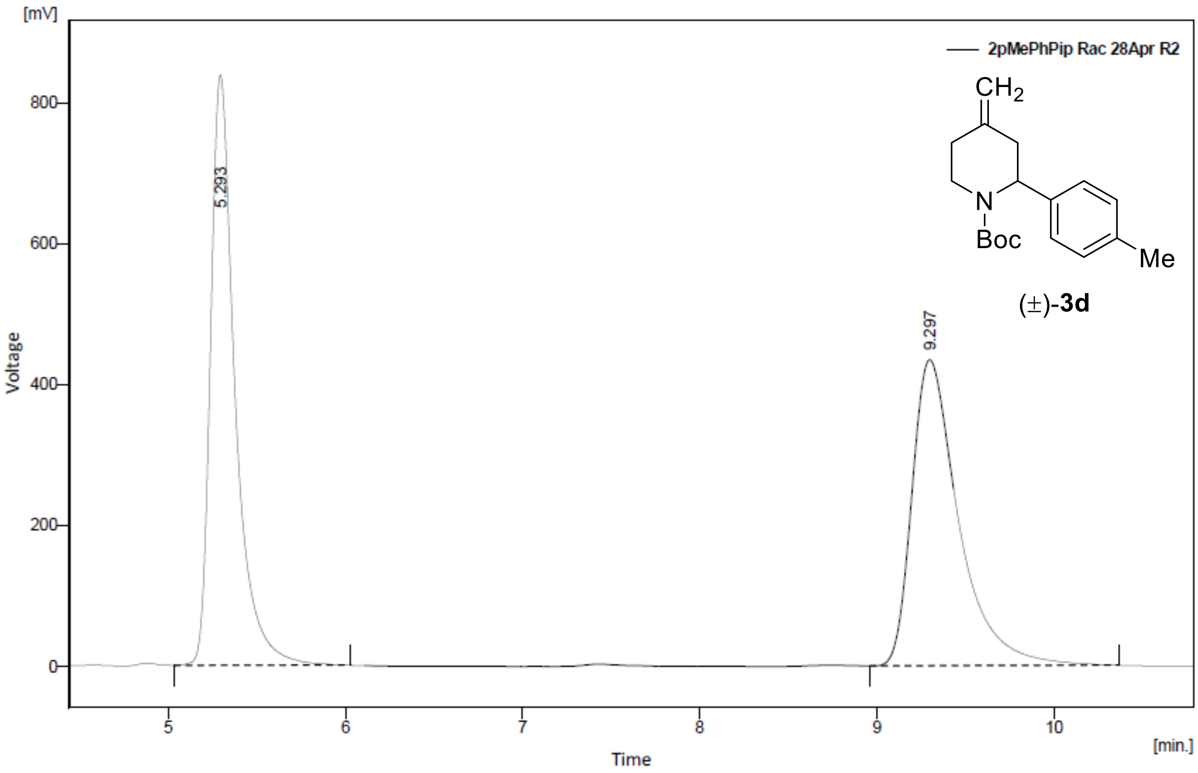
[mV]
800
600
400
200
0
Voltage
5.293
9.297
2pMePhPip Rac 28Apr R2
CH2
N
Boc
Me
(±)-3d
5
6
7
8
9
10
[min.]
Time

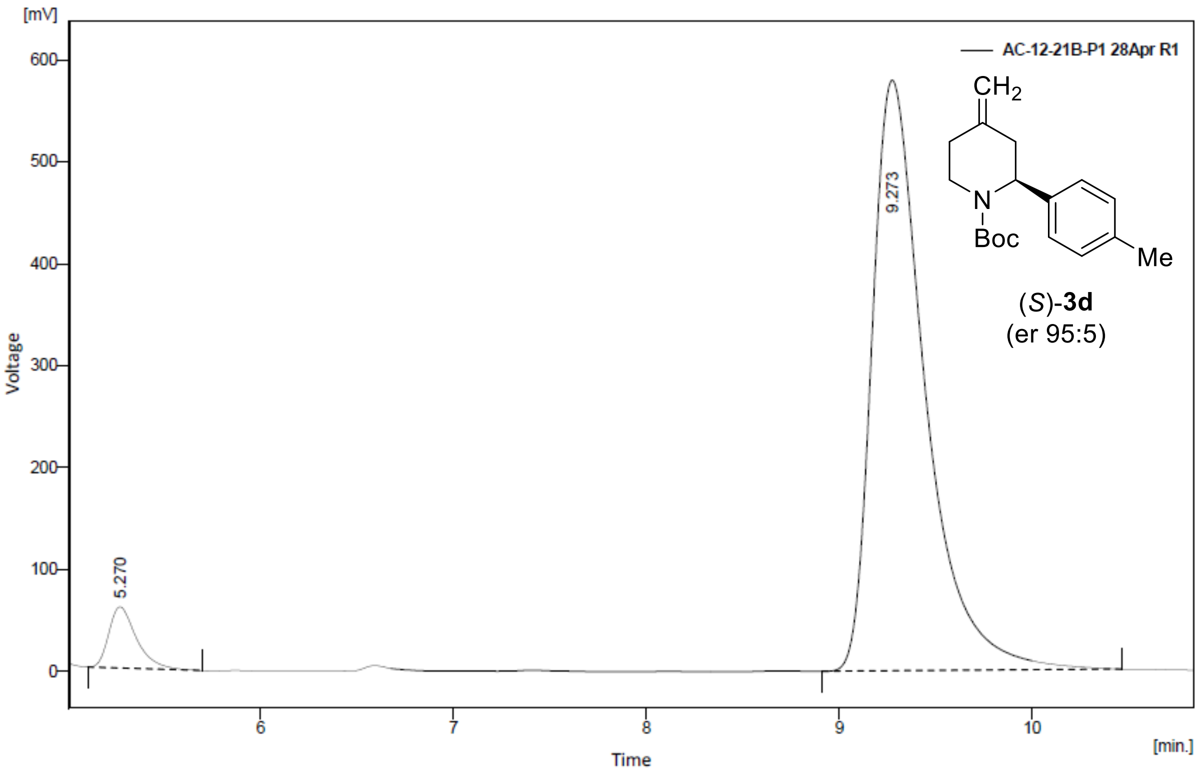
[mV]
600
500
400
300
200
100
0
Voltage
5.270
9.273
AC-12-21B-P1 28Apr R1
CH2
N
Boc
Me
(S)-3d
(er 95:5)
6
7
8
9
10
[min.]
Time

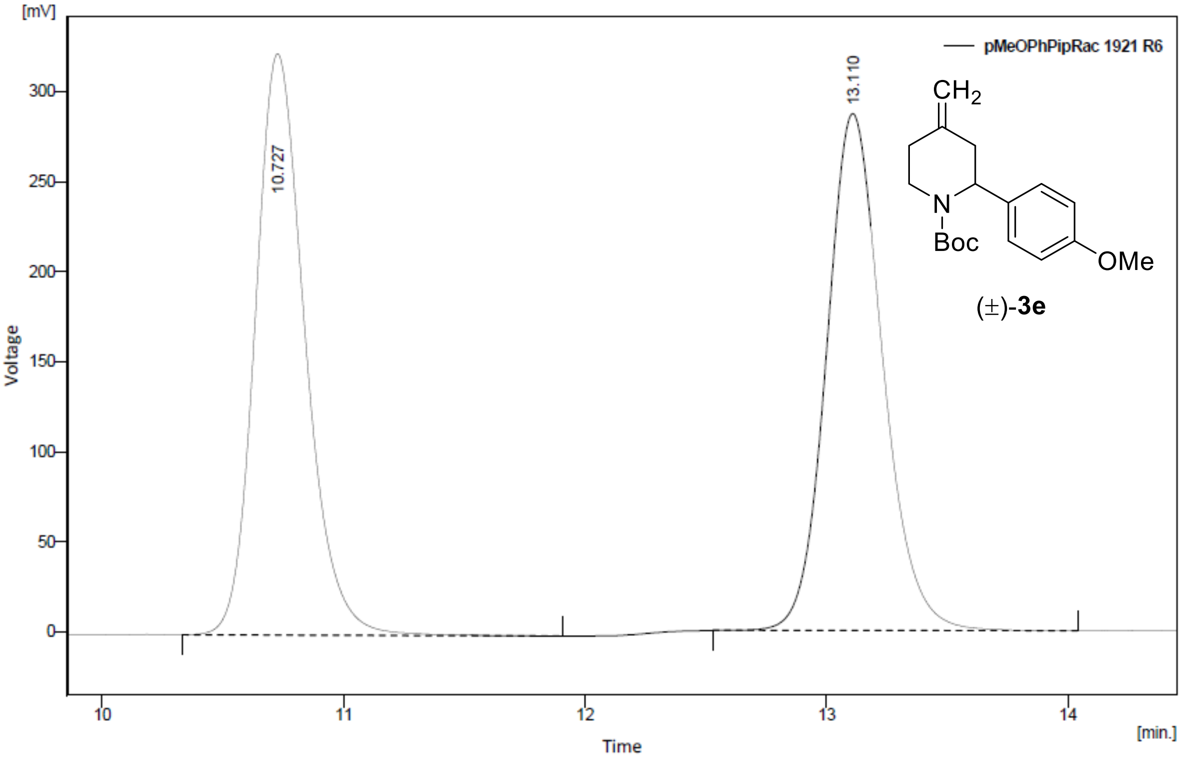

[mV]
Voltage
Time
[min.]
pMeOPhPipRac 1921 R6
10.727
13.110
CH2
N
Boc
OMe
(±)-3e

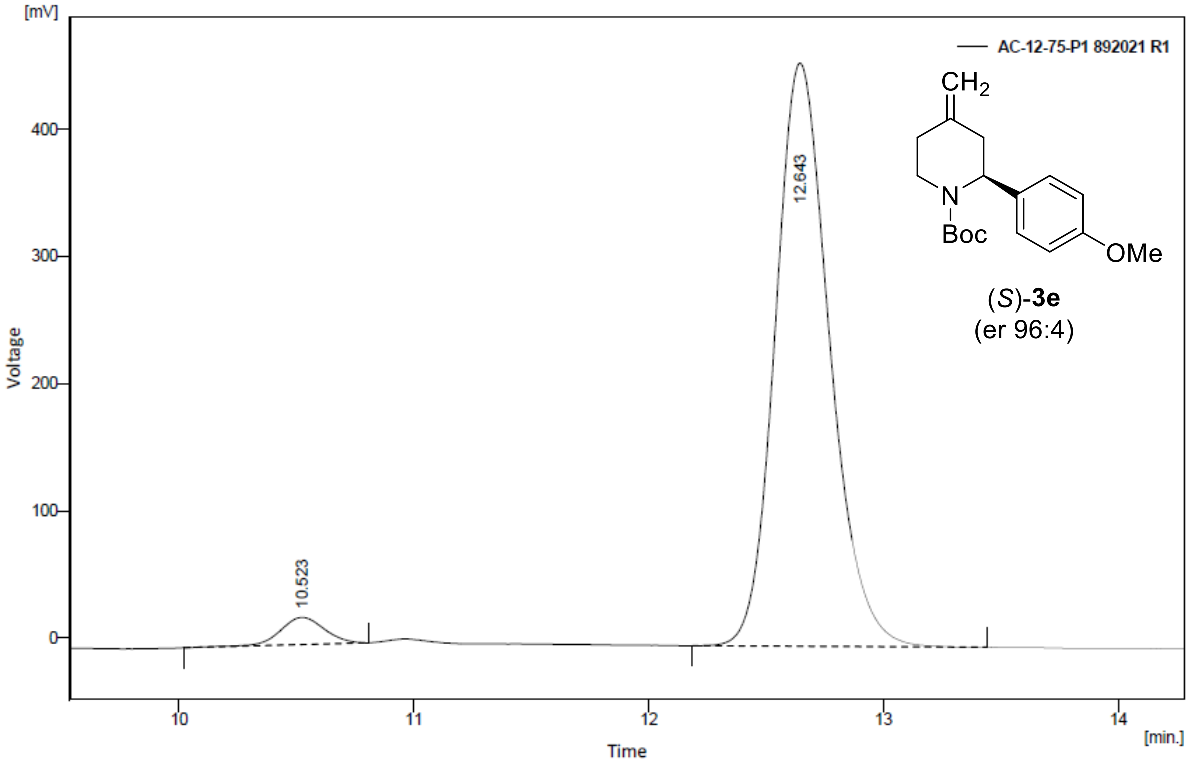

[mV]
Voltage
Time
[min.]
AC-12-75-P1 892021 R1
10.523
12.643
CH2
N
Boc
OMe
(S)-3e
(er 96:4)

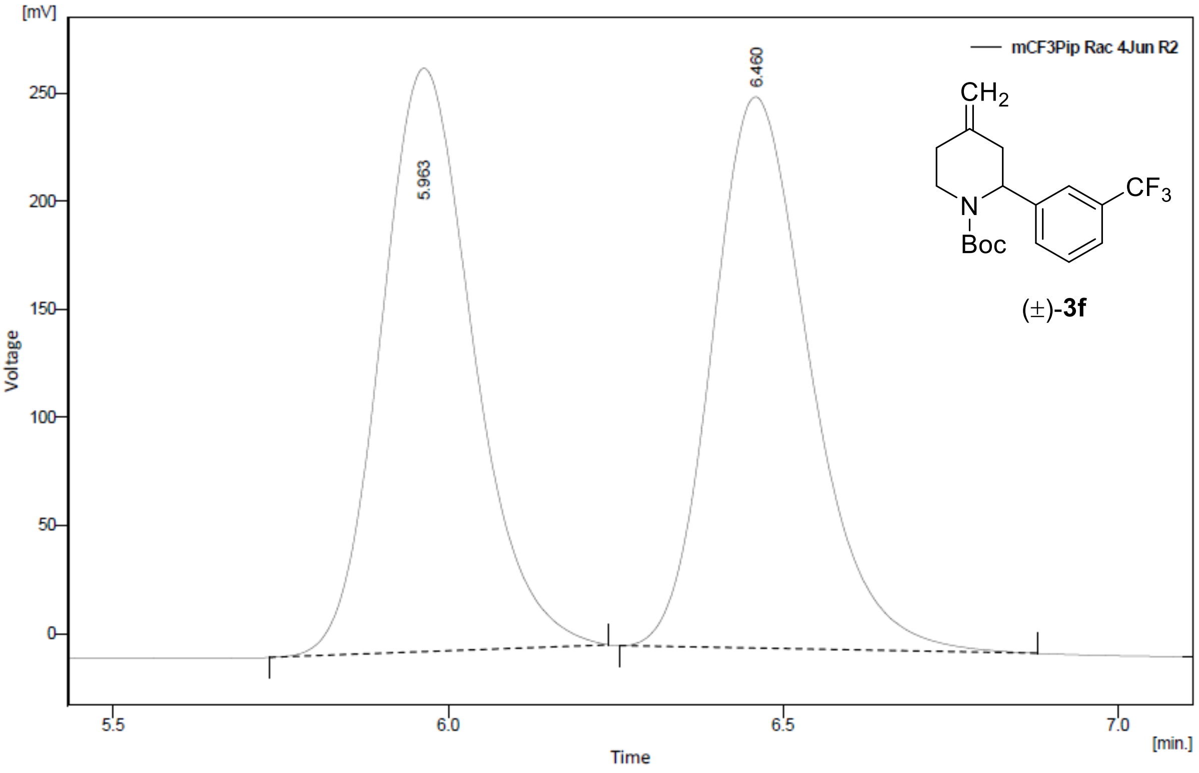
[mV]
250
200
150
100
50
0
Voltage
5.5
6.0
6.5
7.0
[min.]
Time
5.963
6.460
mCF3Pip Rac 4Jun R2
CH2
CF3
N
Boc
(±)-3f

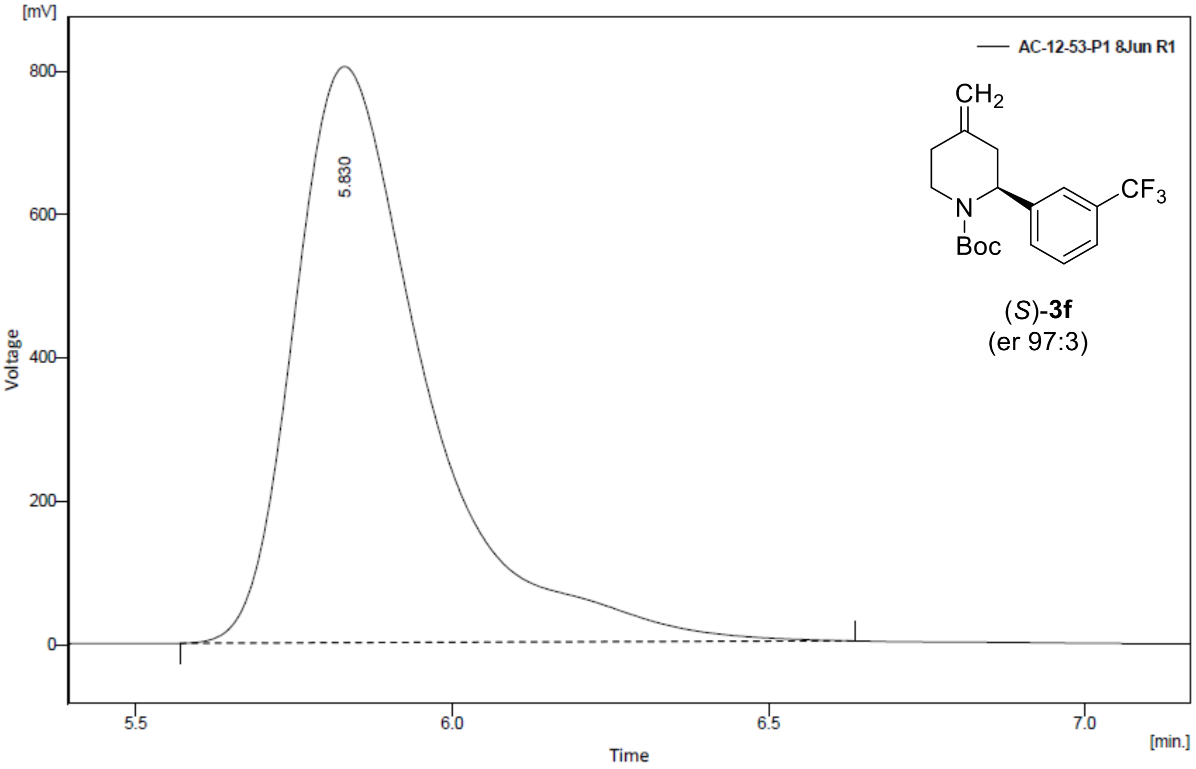
[mV]
800
600
400
200
0
Voltage
5.5
6.0
6.5
7.0
[min.]
Time
5.830
AC-12-53-P1 8Jun R1
CH2
CF3
N
Boc
(S)-3f
(er 97:3)

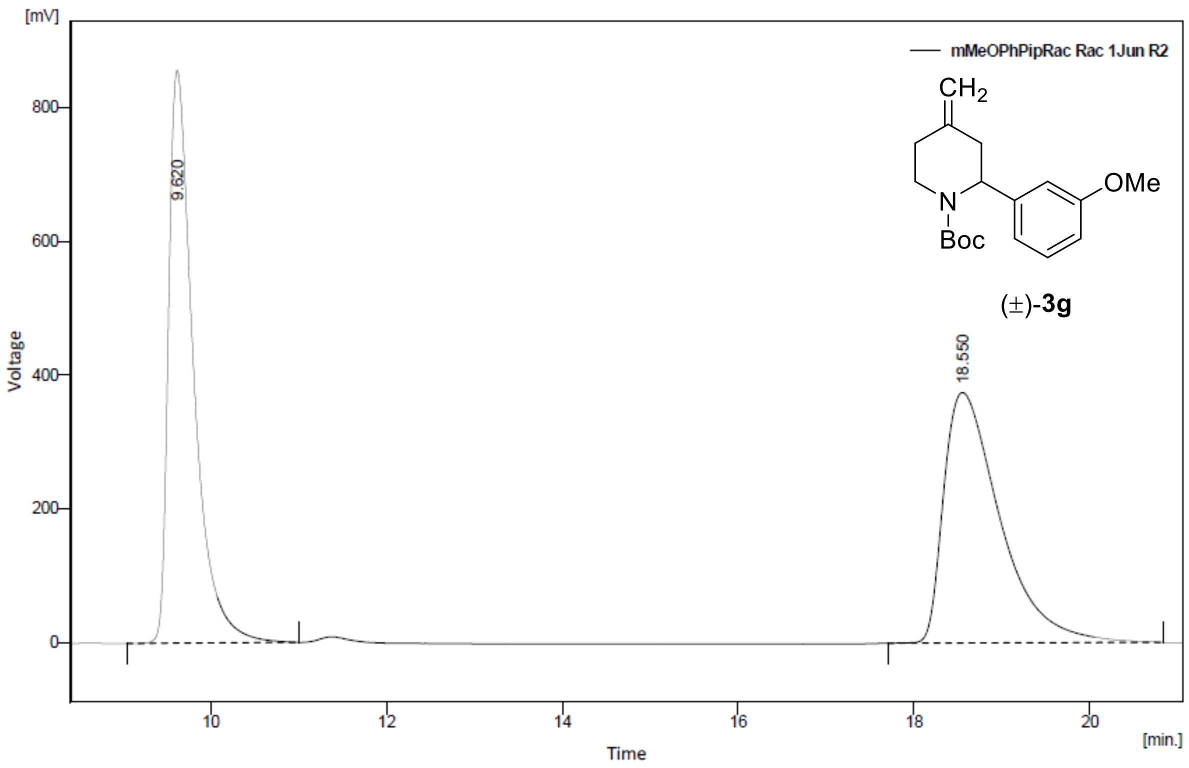
[mV]
800
600
400
200
0
Voltage
9.620
18.550
mMeOPhPipRac Rac 1Jun R2
CH2
OMe
N
Boc
(±)-3g
10
12
14
16
18
20
[min.]
Time

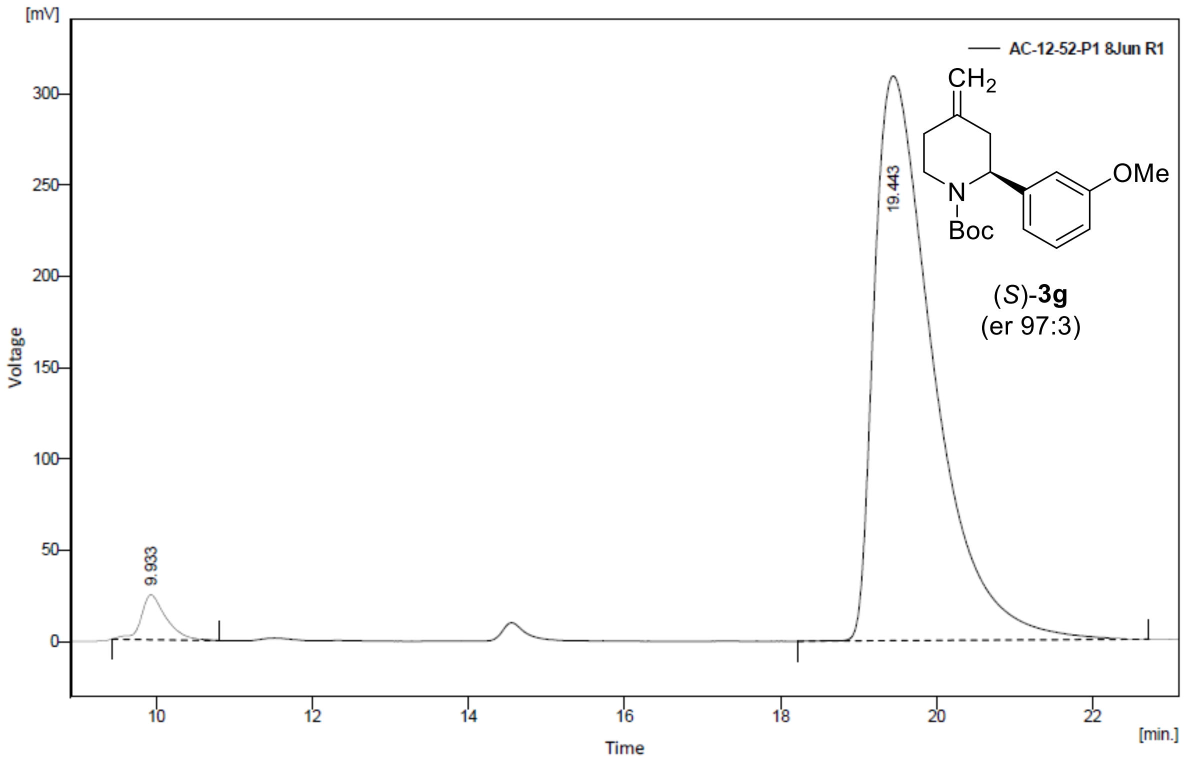
[mV]
300
250
200
150
100
50
0
Voltage
9.933
19.443
AC-12-52-P1 8Jun R1
CH2
OMe
N
Boc
(S)-3g
(er 97:3)
10
12
14
16
18
20
22
[min.]
Time

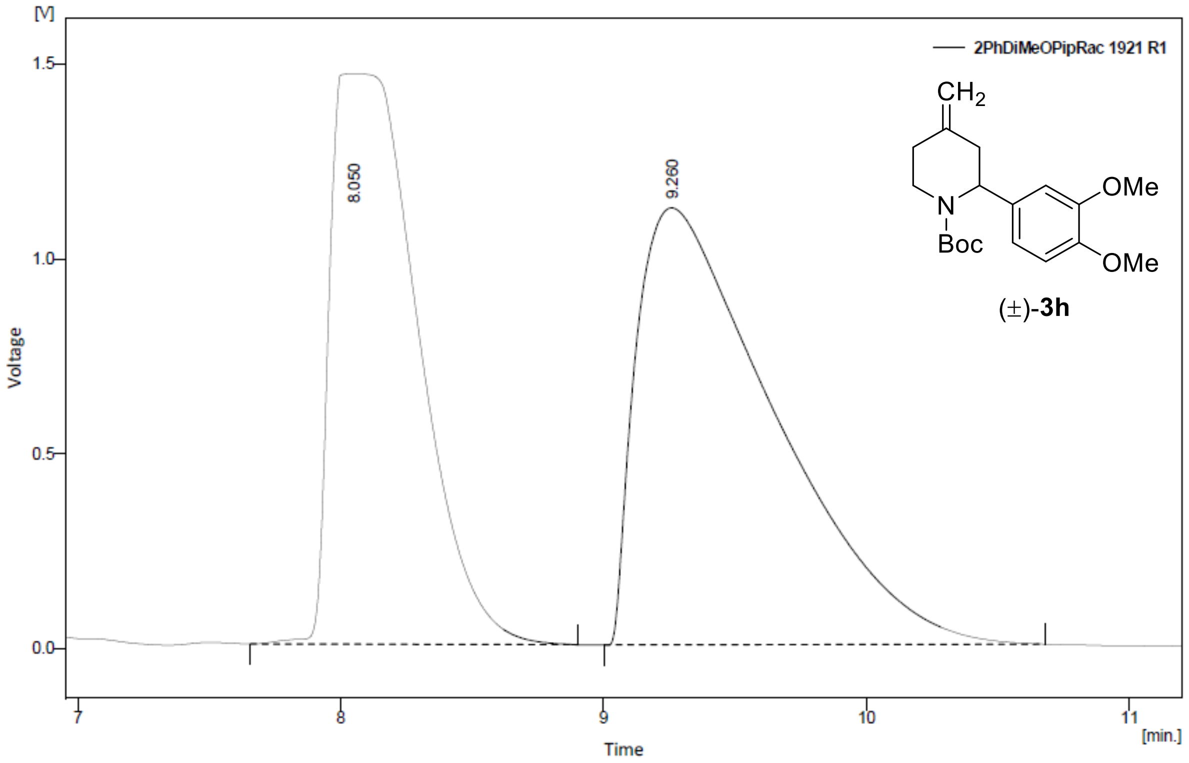

[V]
1.5
1.0
0.5
0.0
Voltage
2PhDiMeOPipRac 1921 R1
8.050
9.260
CH2
N
Boc
OMe
OMe
(±)-3h
7
8
9
10
11
[min.]
Time

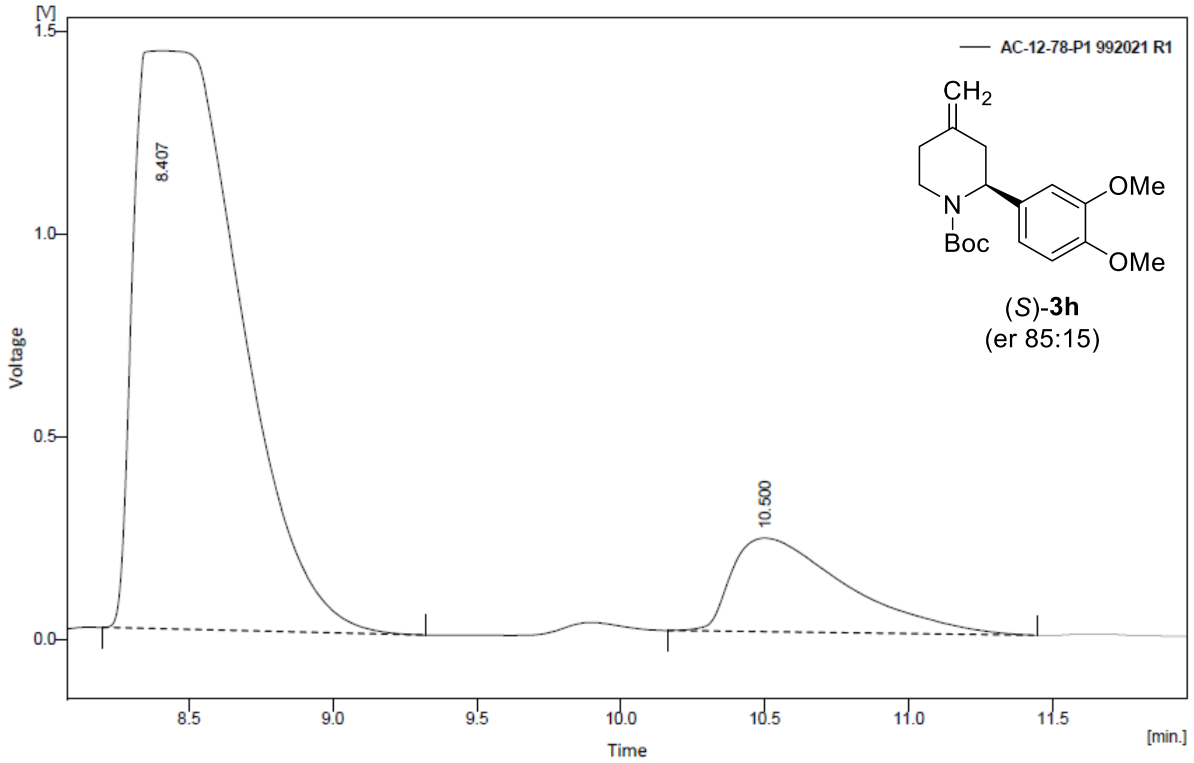

[V]
1.5
1.0
0.5
0.0
Voltage
AC-12-78-P1 992021 R1
8.407
10.500
CH2
N
Boc
OMe
OMe
(S)-3h
(er 85:15)
8.5
9.0
9.5
10.0
10.5
11.0
11.5
[min.]
Time

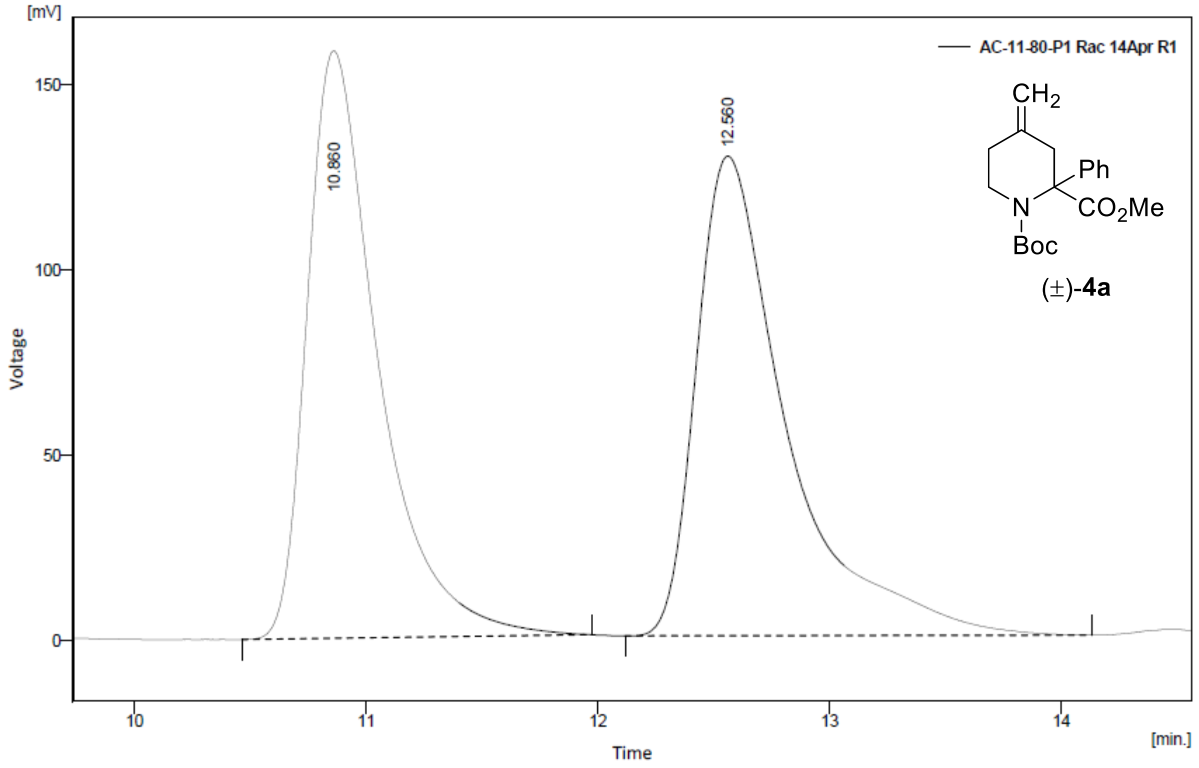
[mV]
150
100
50
0
Voltage
10
11
12
13
14
[min.]
Time
10.860
12.560
AC-11-80-P1 Rac 14Apr R1
CH2
Ph
CO2Me
N
Boc
(±)-4a

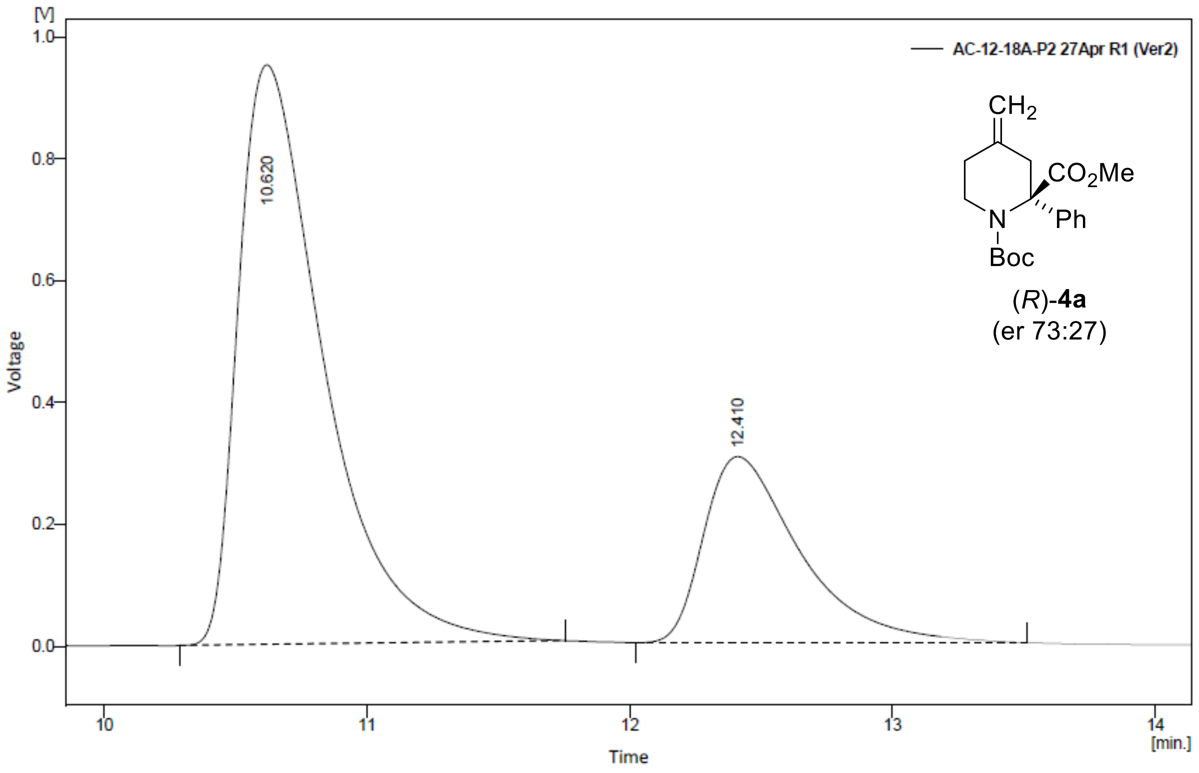
[V]
1.0
0.8
0.6
0.4
0.2
0.0
Voltage
10
11
12
13
14
[min.]
Time
10.620
12.410
AC-12-18A-P2 27Apr R1 (Ver2)
CH2
CO2Me
Ph
N
Boc
(R)-4a
(er 73:27)

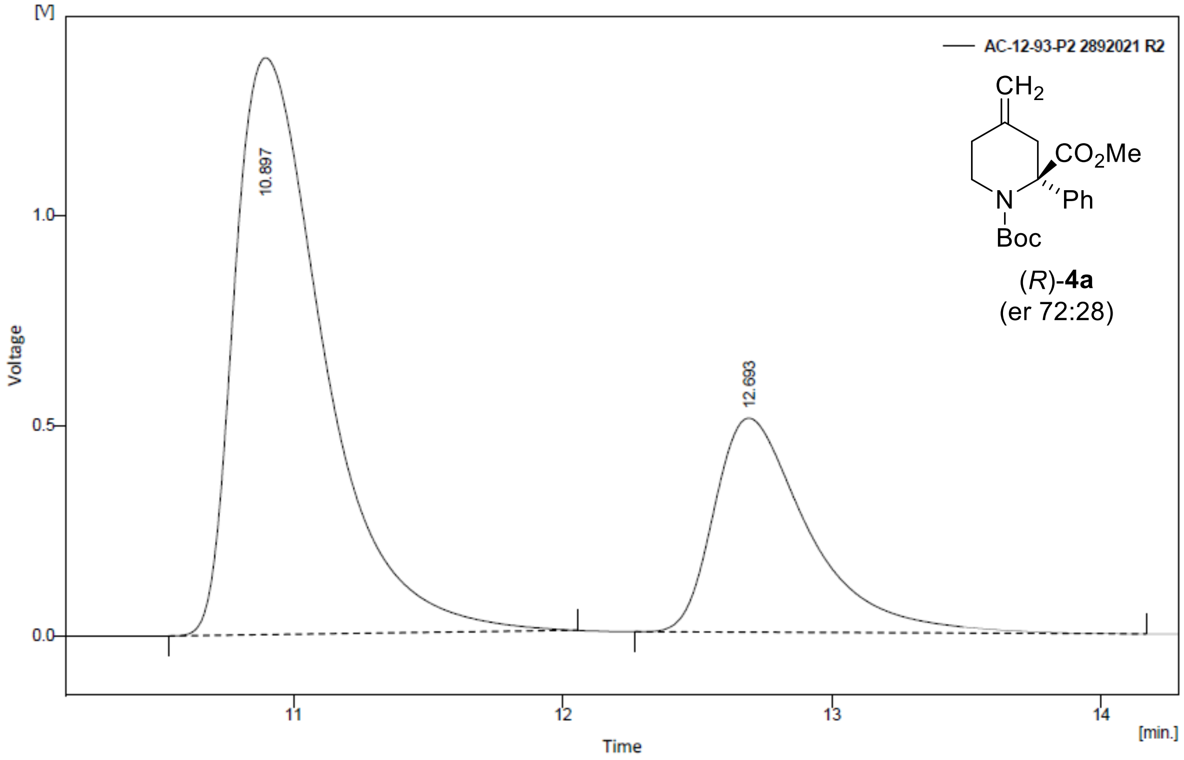
AC-12-93-P2 2892021 R2
CH2
CO2Me
N
Ph
Boc
(R)-4a
(er 72:28)
10.897
12.693
[V]
Voltage
1.0
0.5
0.0
11
12
13
14
Time
[min.]

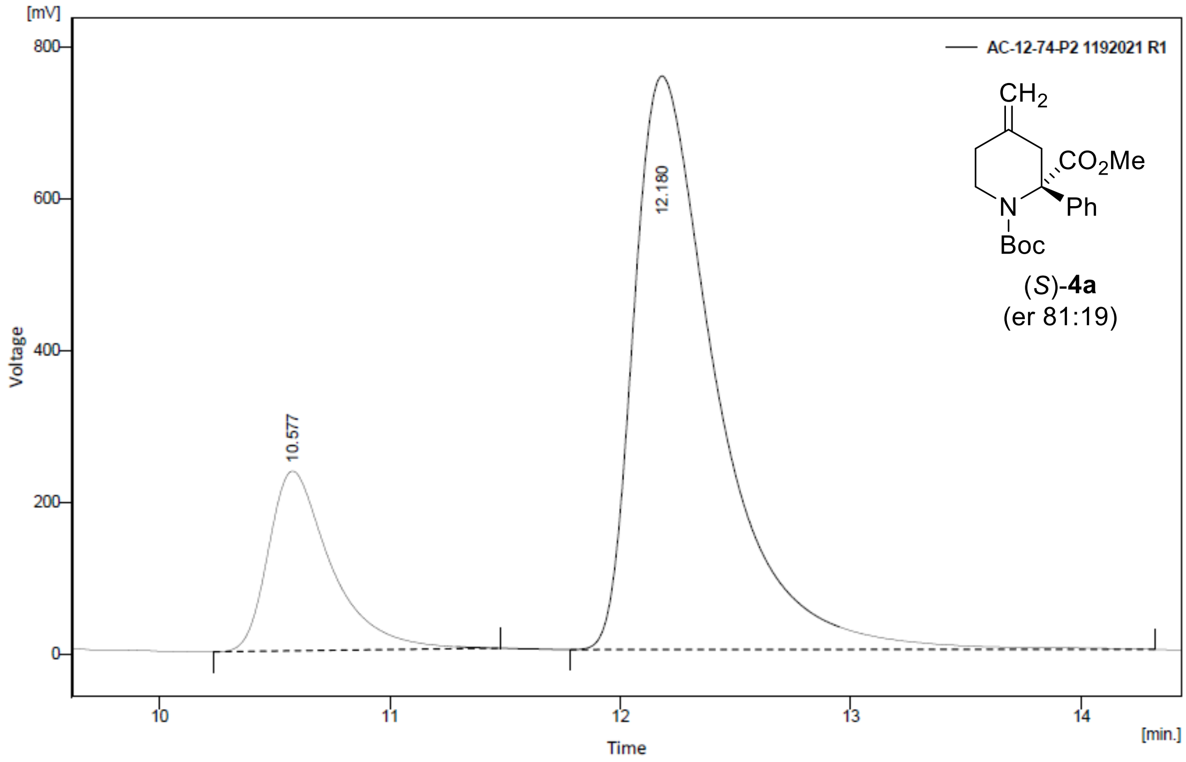
AC-12-74-P2 1192021 R1
CH2
CO2Me
N
Ph
Boc
(S)-4a
(er 81:19)
10.577
12.180
[mV]
Voltage
800
600
400
200
0
10
11
12
13
14
Time
[min.]

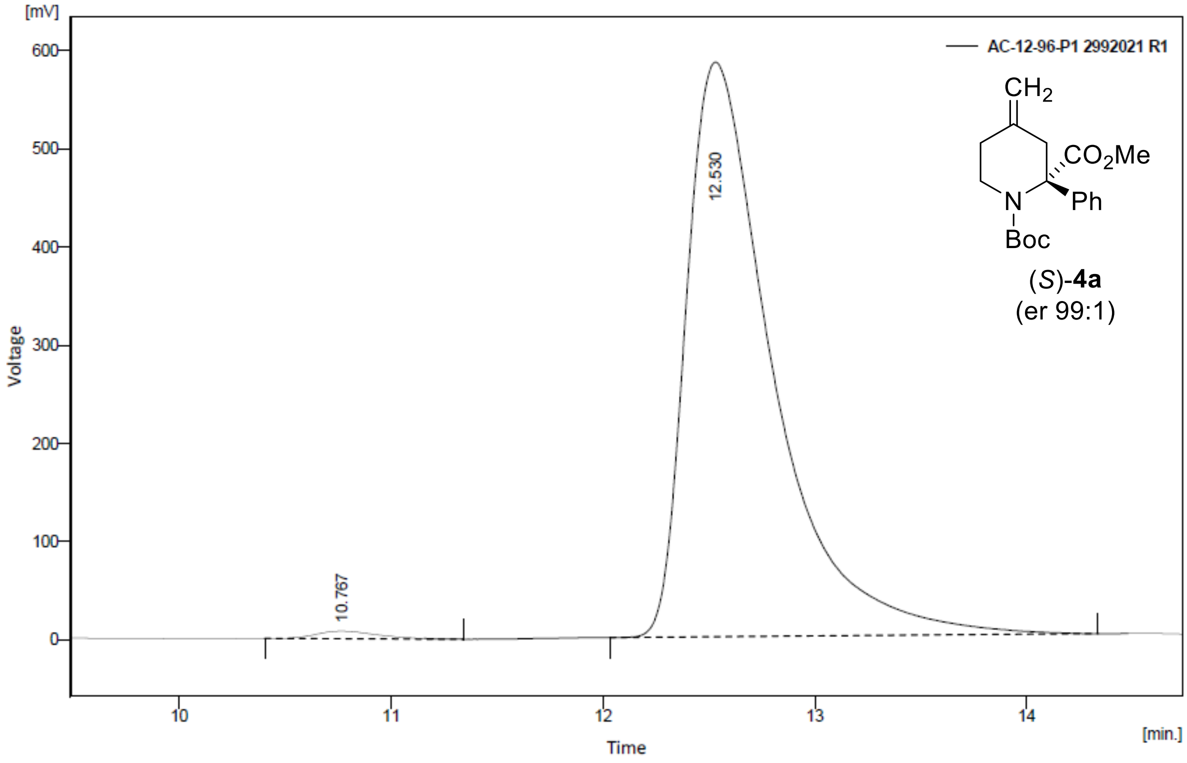

[mV]
600
500
400
300
200
100
0
Voltage
10
11
12
13
14
Time
[min.]
AC-12-96-P1 2992021 R1
10.767
12.530
CH2
CO2Me
Ph
N
Boc
(S)-4a
(er 99:1)

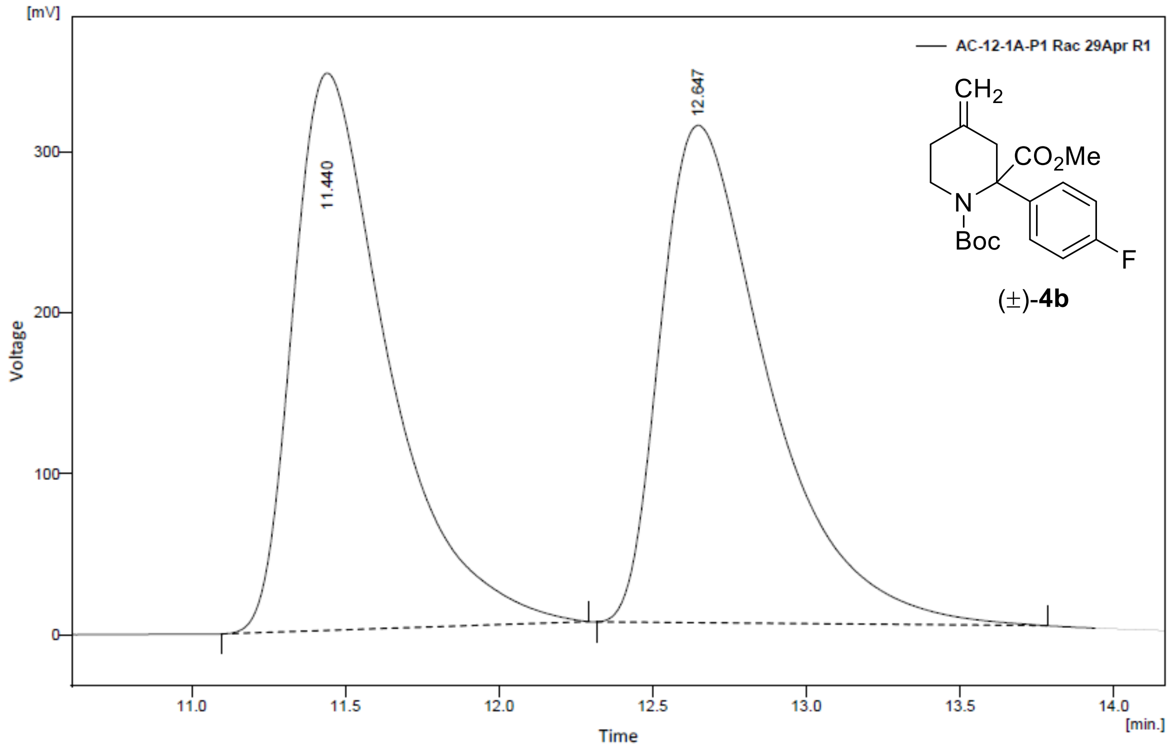

HPLC of (±)-**4b** from Agilent system fitted with a Chiral Art Cellulose-C column:

Chiral Art Cellulose-C Column			
[redacted]			
	R_f/min	Area/mAU.s	Area/%
1	7.670	3502.22	49.52
2	8.372	3570.49	50.48
	Total	7072.71	100.00

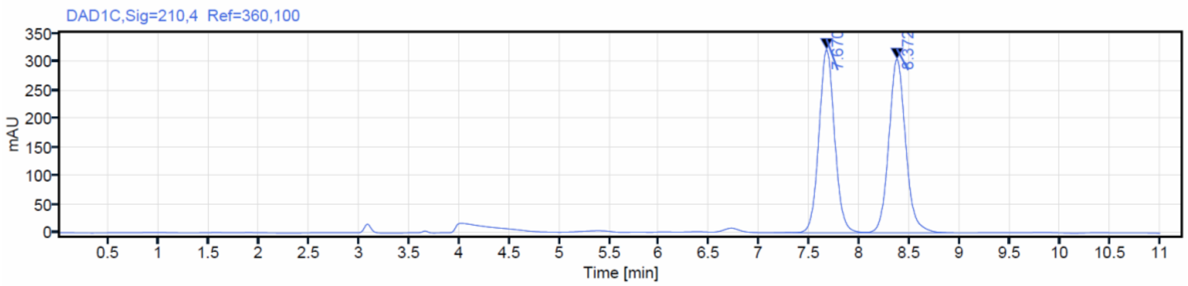

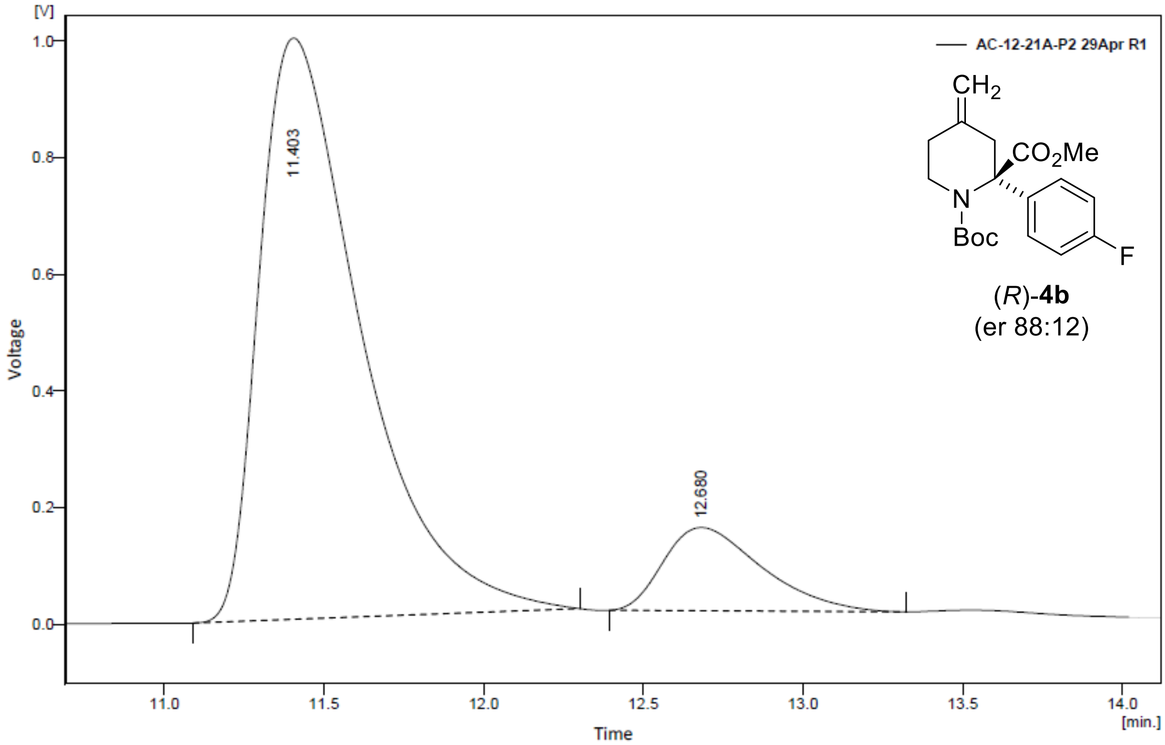

HPLC of (*R*)-**4b** from Agilent system fitted with a Chiral Art Cellulose-C column:

Chiral Art Cellulose-C Column			
	R_f/min	Area/mAU.s	Area/%
1	7.903	13930.66	24.72
2	8.643	42424.82	75.28
	Total	56355.48	100.00

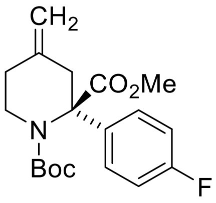

(*R*)-**4b**
(er 75:25)

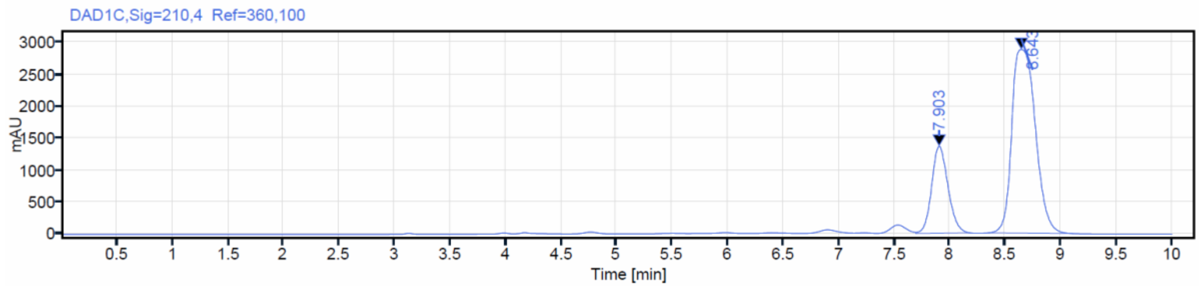

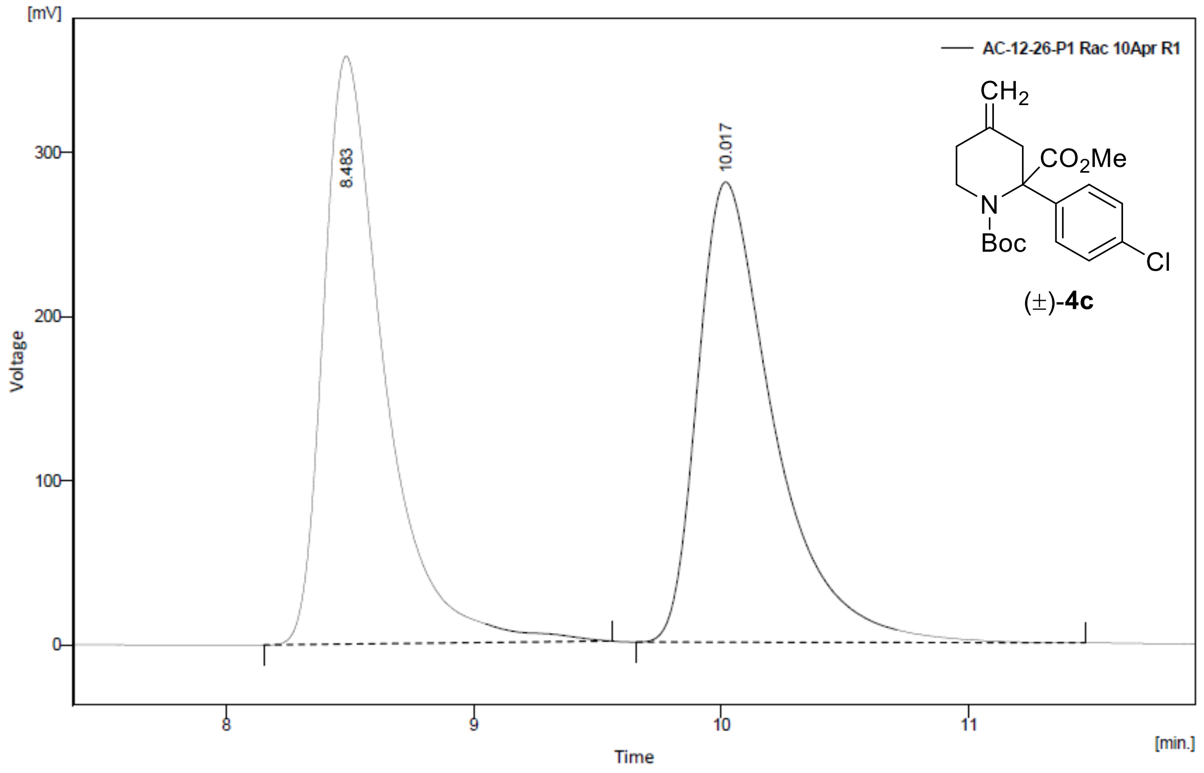
[mV]
300
200
100
0
Voltage
8.483
10.017
AC-12-26-P1 Rac 10Apr R1
CH2
CO2Me
N
Boc
Cl
(±)-4c
8
9
10
11
[min.]
Time

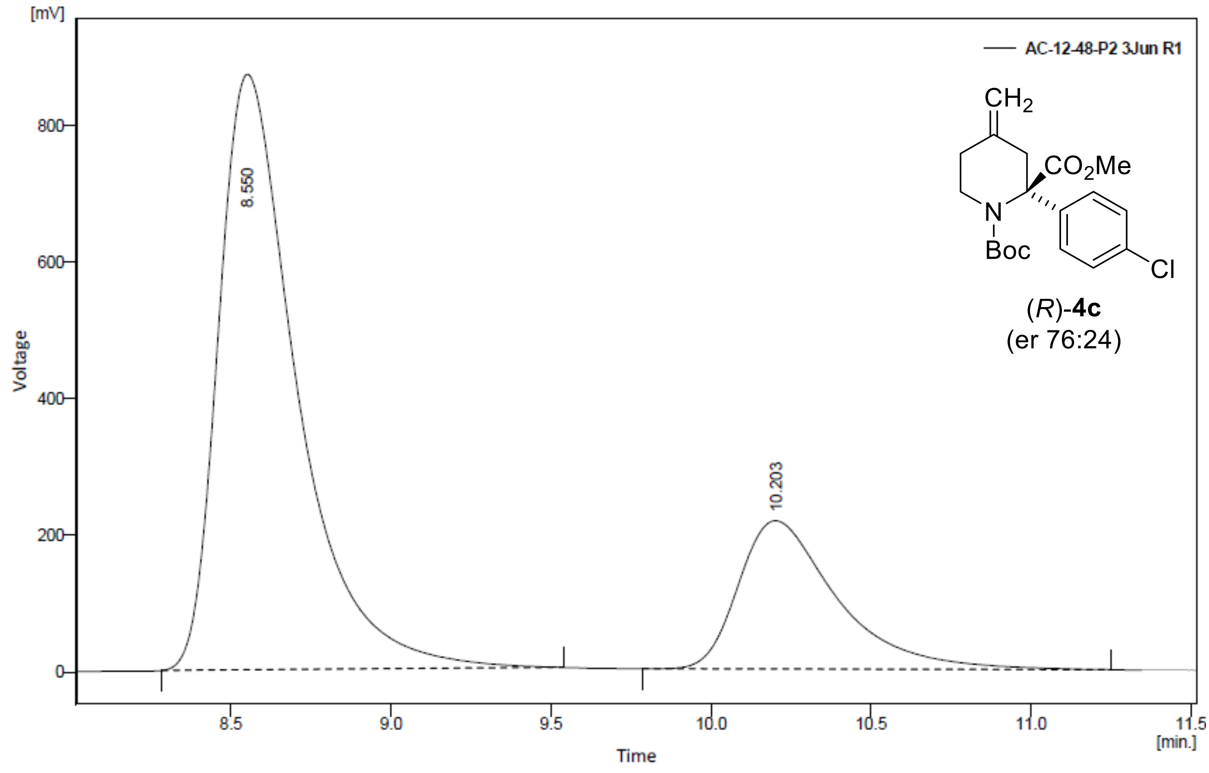
[mV]
800
600
400
200
0
Voltage
8.550
10.203
AC-12-48-P2 3Jun R1
CH2
CO2Me
N
Boc
Cl
(R)-4c
(er 76:24)
8.5
9.0
9.5
10.0
10.5
11.0
11.5
[min.]
Time

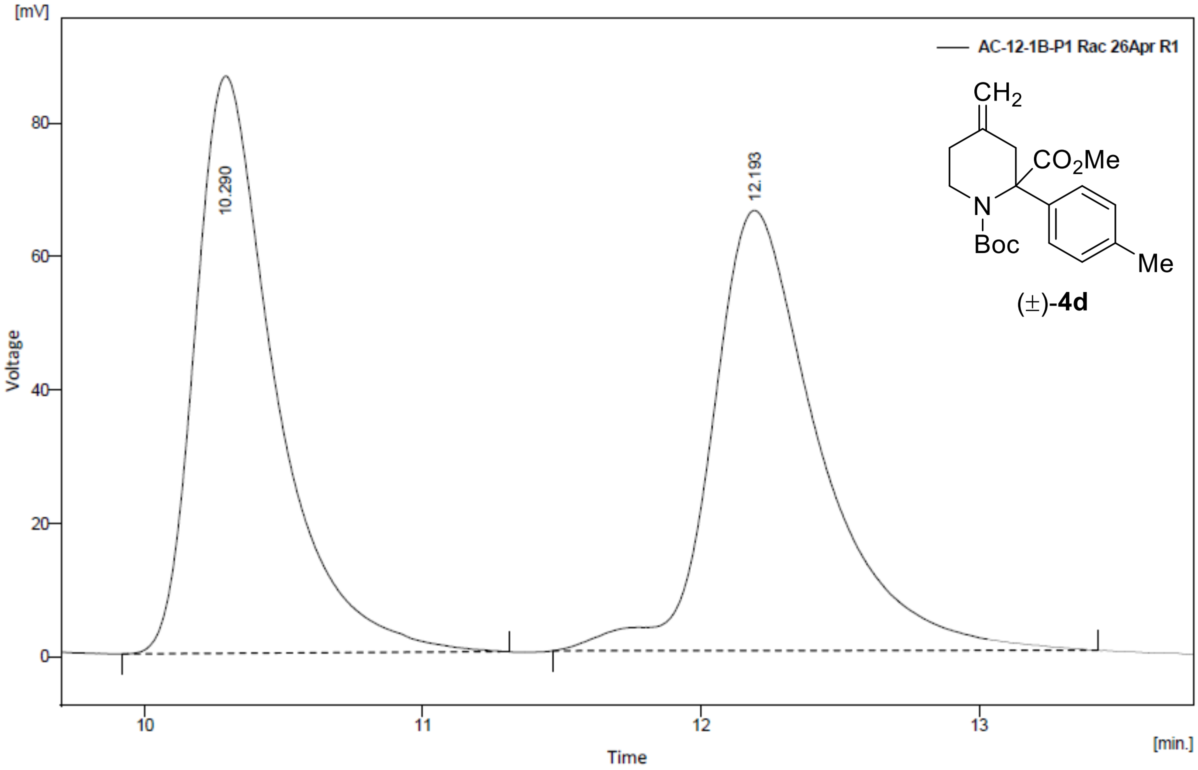
[mV]
AC-12-1B-P1 Rac 26Apr R1
CH2
CO2Me
N
Boc
Me
(±)-4d
10.290
12.193
Voltage
Time
[min.]

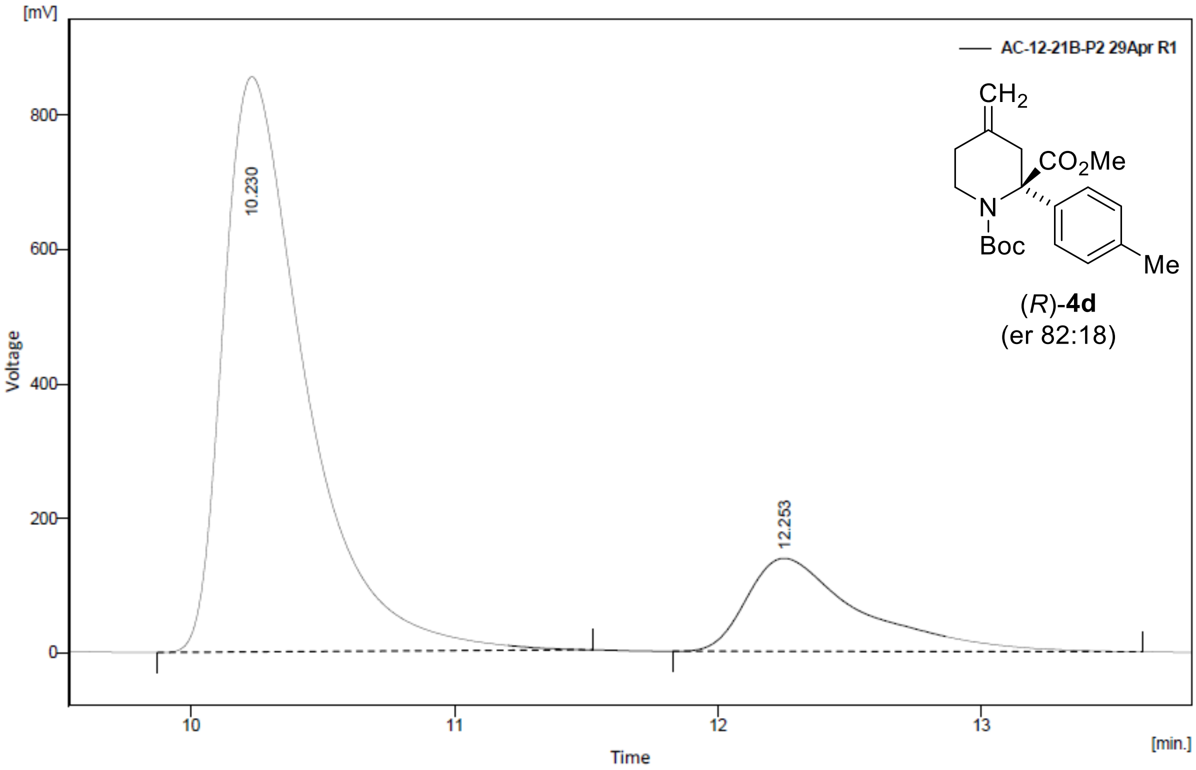
[mV]
AC-12-21B-P2 29Apr R1
CH2
CO2Me
N
Boc
Me
(R)-4d
(er 82:18)
10.230
12.253
Voltage
Time
[min.]

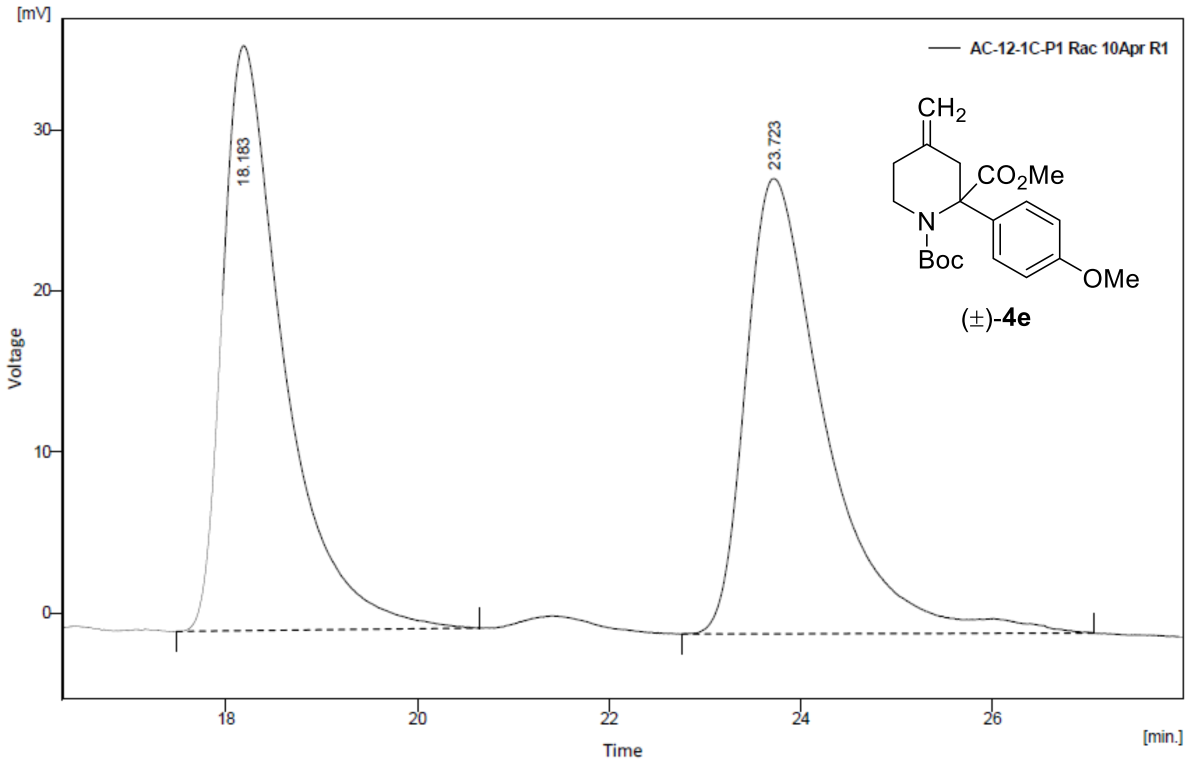
[mV]
AC-12-1C-P1 Rac 10Apr R1
18.183
23.723
Voltage
Time
[min.]
CH2
CO2Me
N
Boc
OMe
(±)-4e

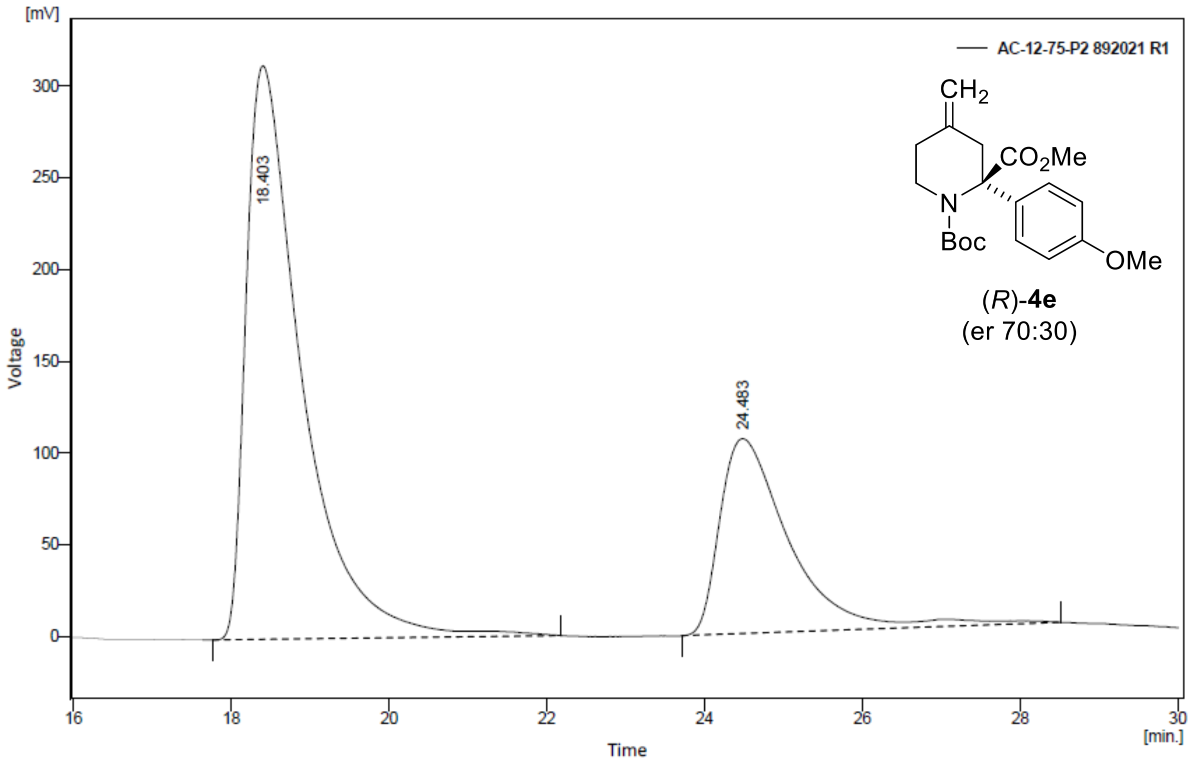
[mV]
AC-12-75-P2 892021 R1
18.403
24.483
Voltage
Time
[min.]
CH2
CO2Me
N
Boc
OMe
(R)-4e
(er 70:30)

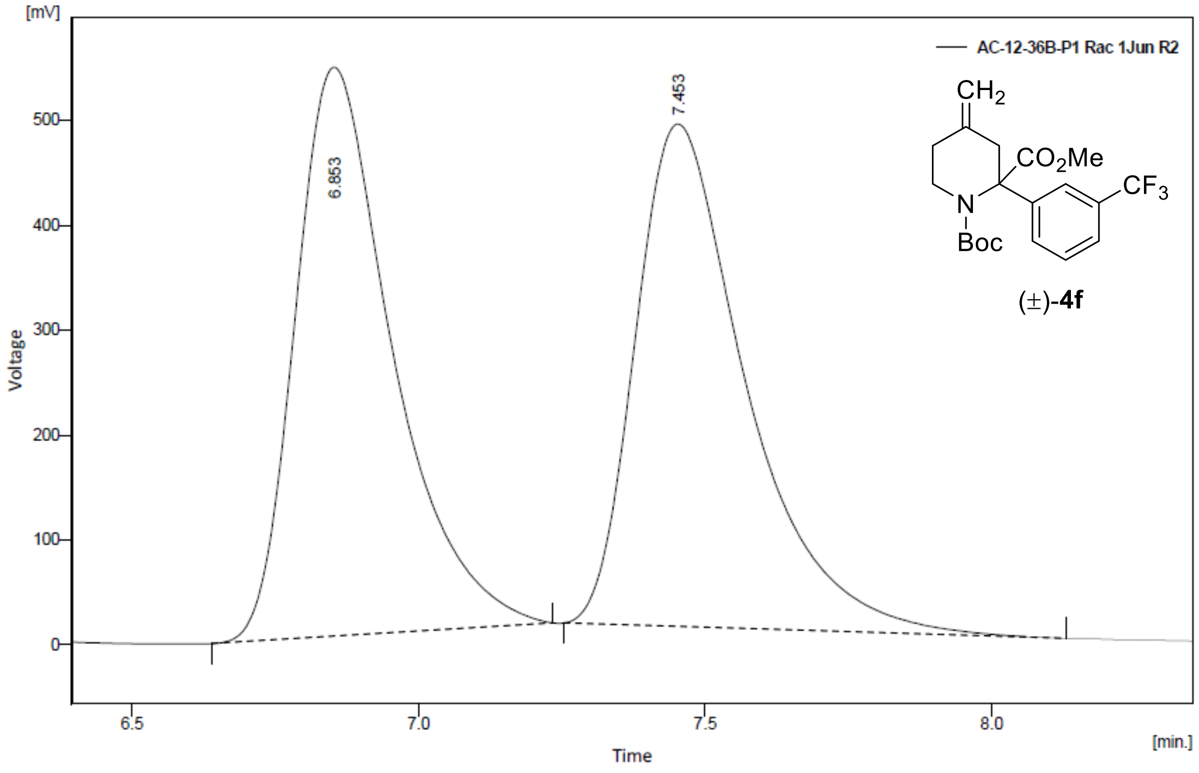
[mV]
Voltage
Time
[min.]
AC-12-36B-P1 Rac 1Jun R2
6.853
7.453
CH2
CO2Me
CF3
N
Boc
(±)-4f

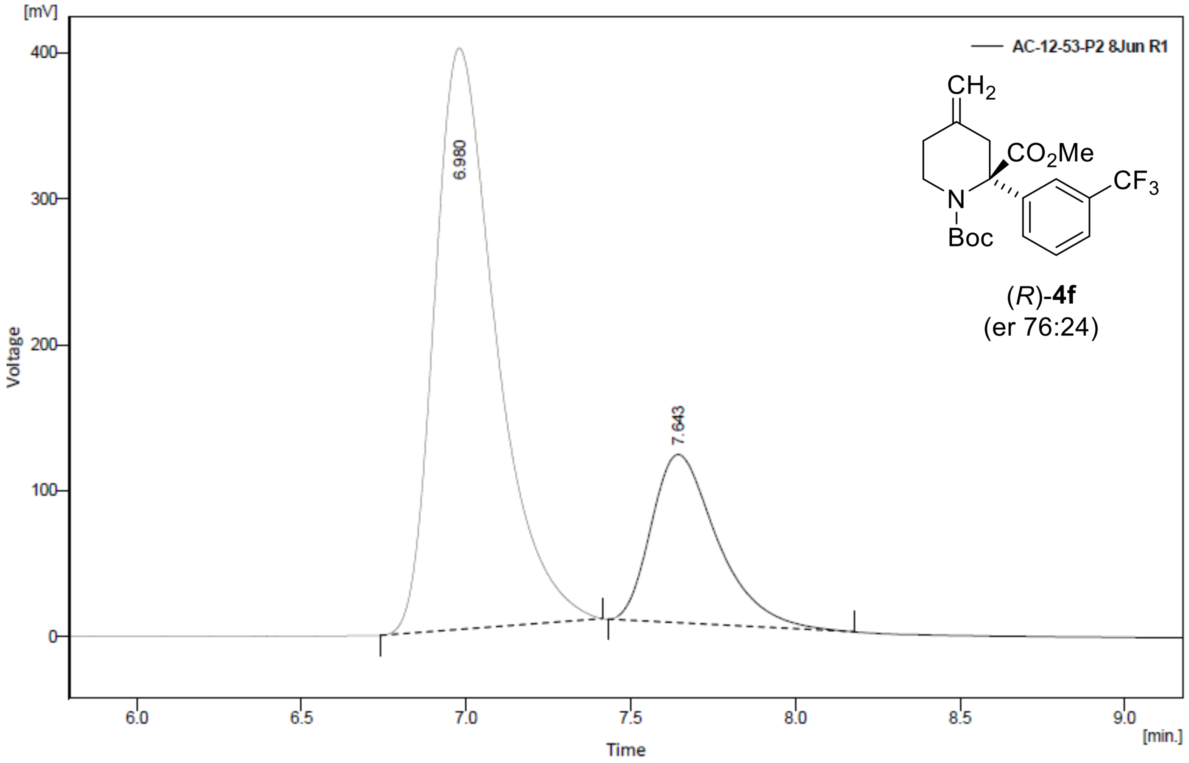
[mV]
Voltage
Time
[min.]
AC-12-53-P2 8Jun R1
6.980
7.643
CH2
CO2Me
CF3
N
Boc
(R)-4f
(er 76:24)

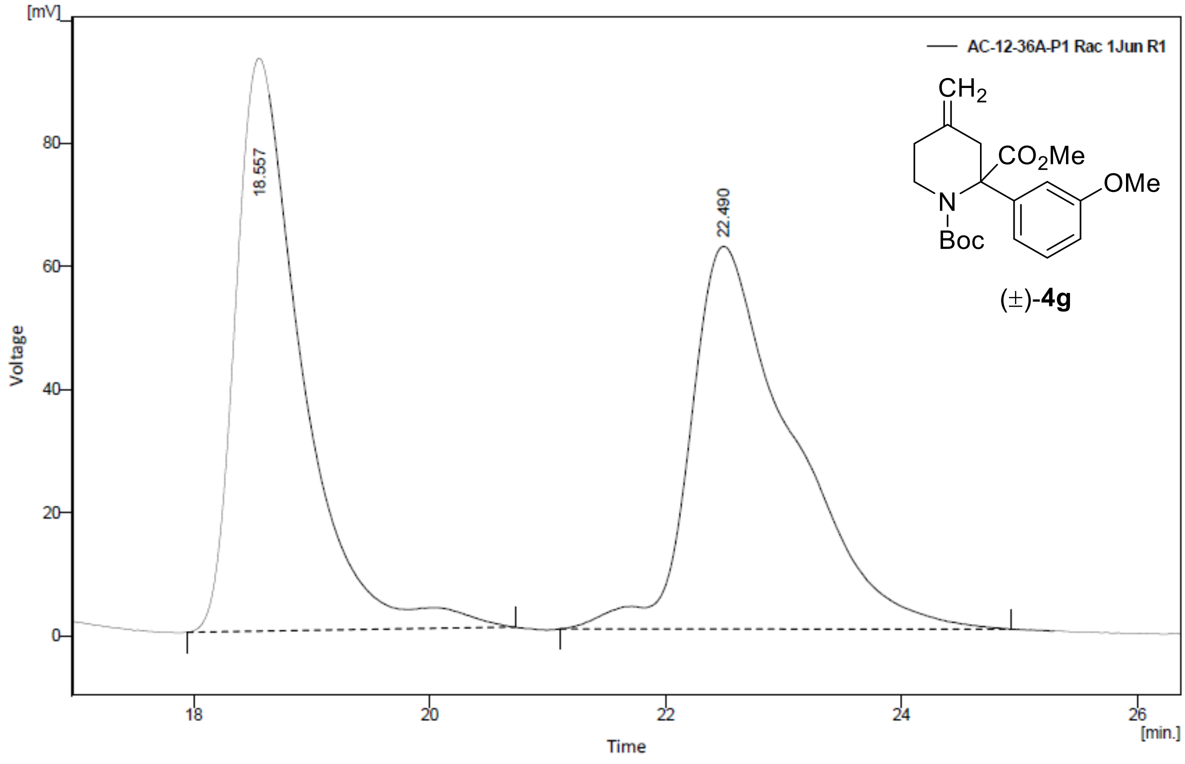
[mV]
Voltage
Time
[min.]
AC-12-36A-P1 Rac 1Jun R1
18.557
22.490
CH2
CO2Me
OMe
N
Boc
(±)-4g

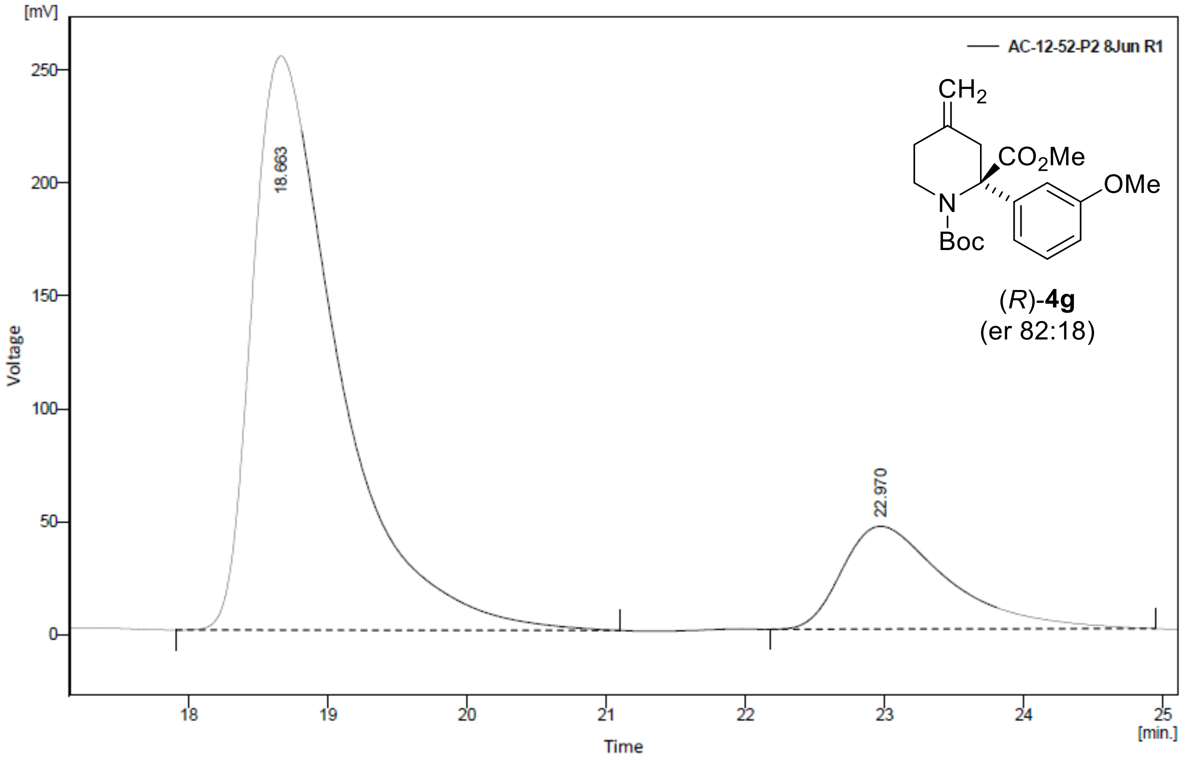
[mV]
Voltage
Time
[min.]
AC-12-52-P2 8Jun R1
18.663
22.970
CH2
CO2Me
OMe
N
Boc
(R)-4g
(er 82:18)

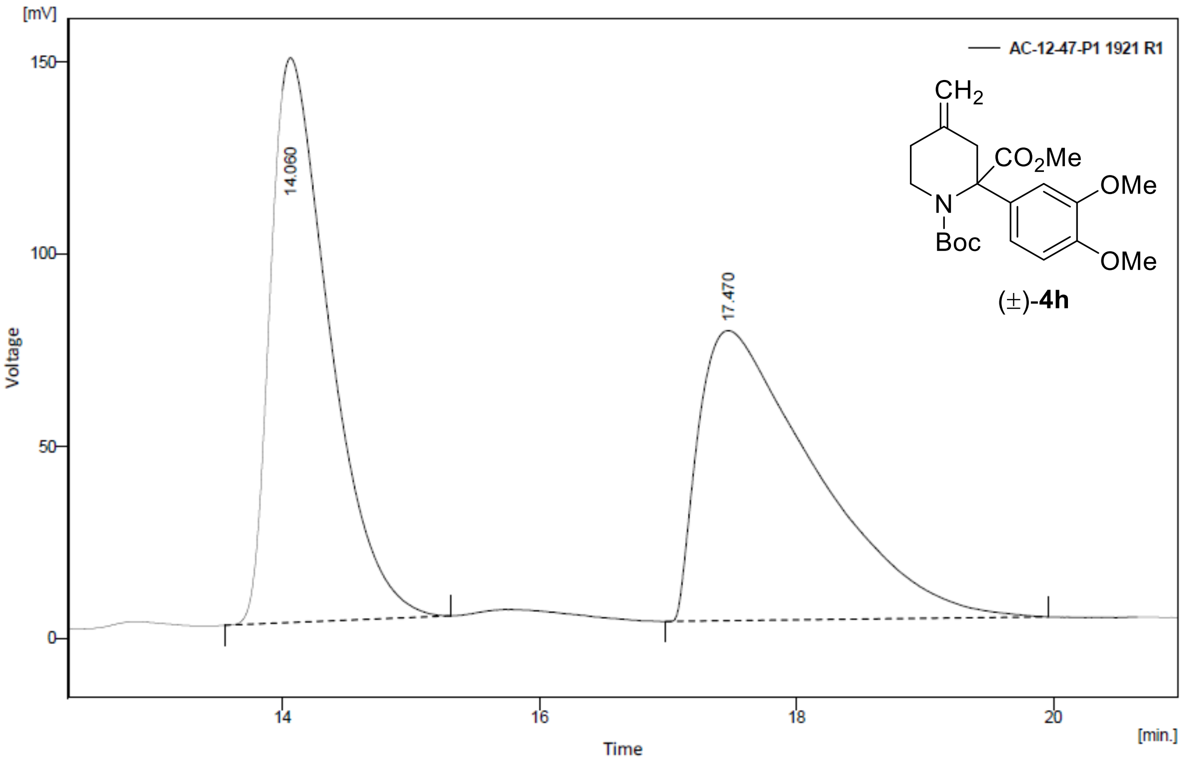

[mV]
Voltage
Time
[min.]
AC-12-47-P1 1921 R1
14.060
17.470
CH2
CO2Me
OMe
OMe
N
Boc
(±)-4h

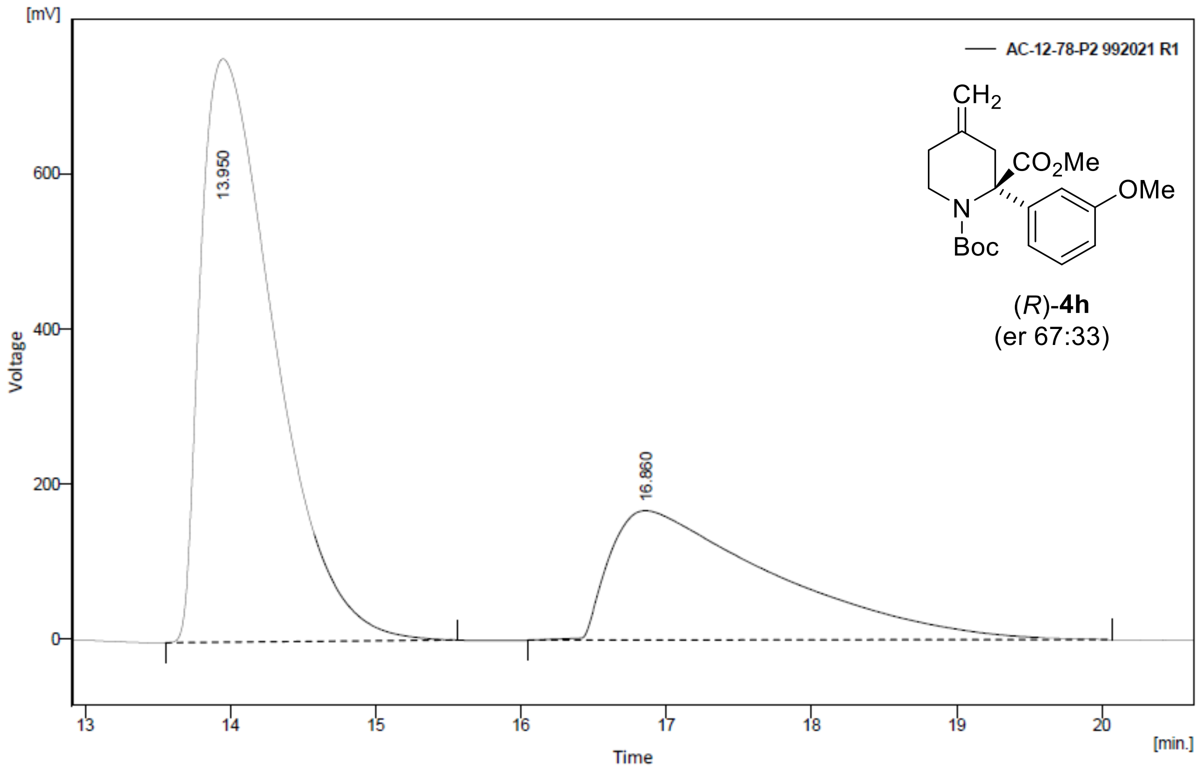

[mV]
Voltage
Time
[min.]
AC-12-78-P2 992021 R1
13.950
16.860
CH2
CO2Me
OMe
N
Boc
(R)-4h
(er 67:33)

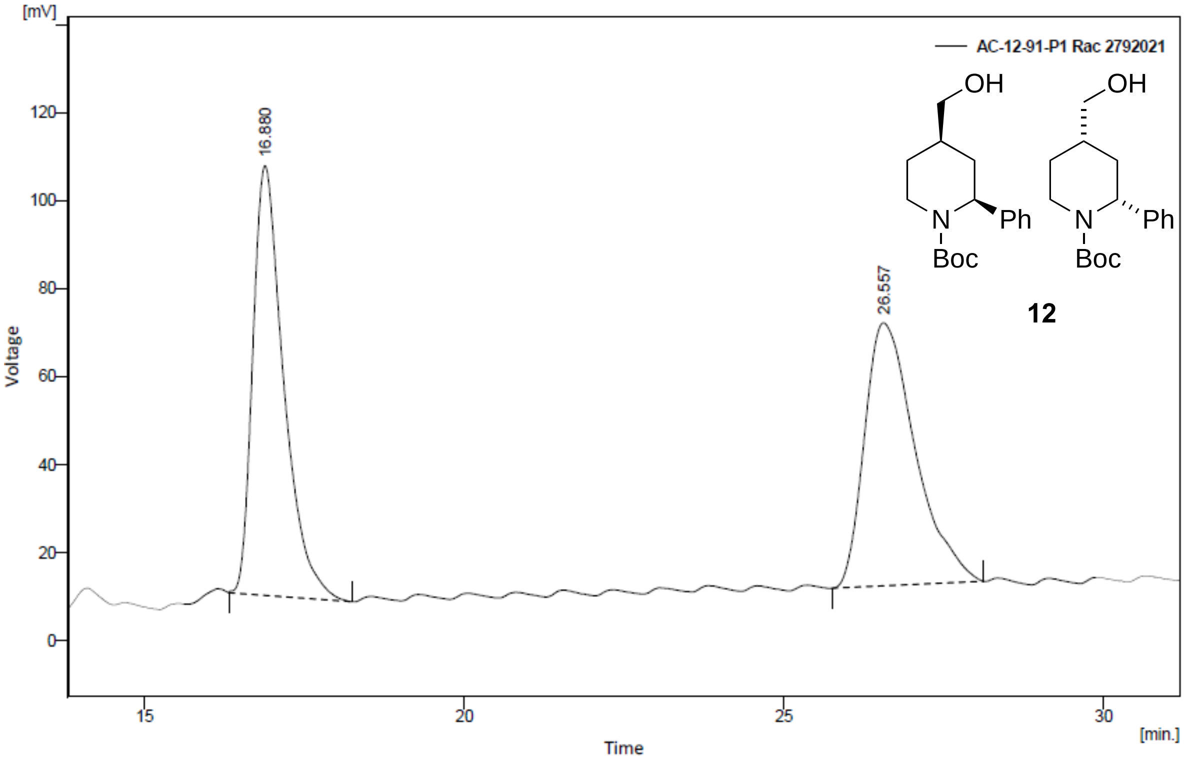
[mV]
AC-12-91-P1 Rac 2792021
16.880
26.557
OH
N
Ph
Boc
12
Voltage
Time
[min.]

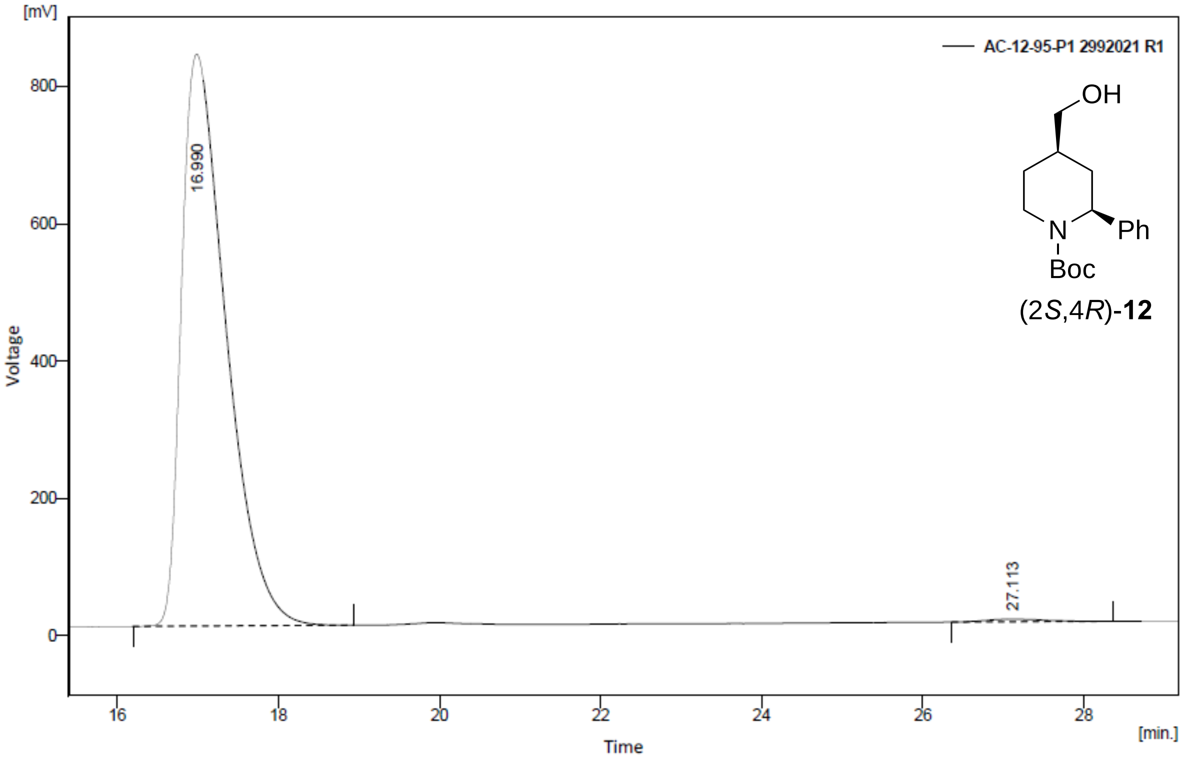
[mV]
AC-12-95-P1 2992021 R1
16.990
27.113
OH
N
Ph
Boc
(2S,4R)-12
Voltage
Time
[min.]

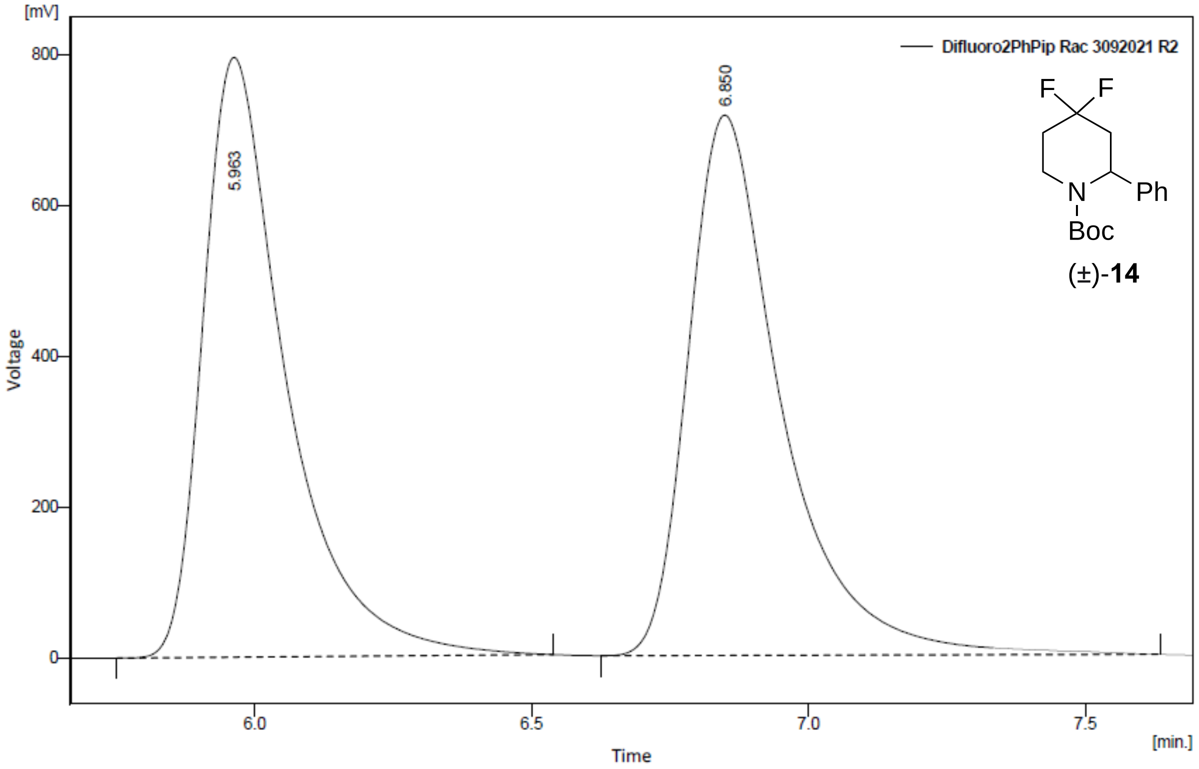
[mV]
800
600
400
200
0
Voltage
5.963
6.850
Difluoro2PhPip Rac 3092021 R2
F
F
N
Ph
Boc
(±)-14
6.0
6.5
7.0
7.5
[min.]
Time

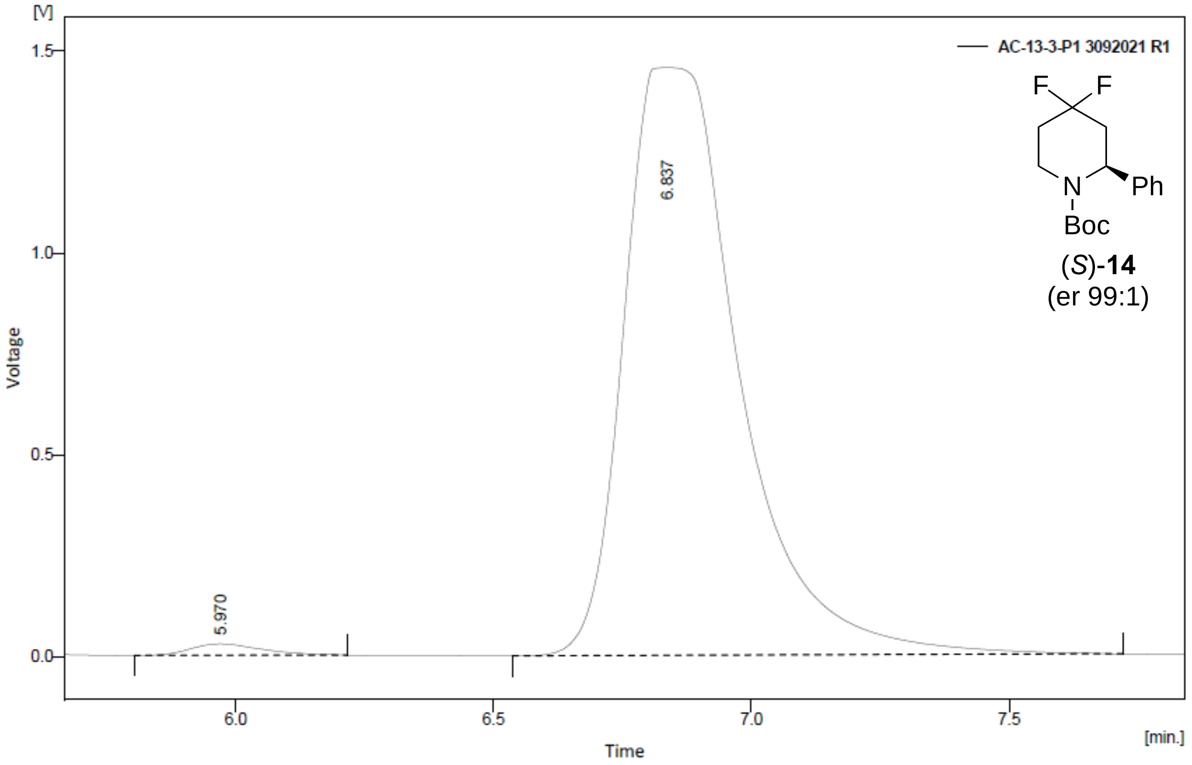
[V]
1.5
1.0
0.5
0.0
Voltage
5.970
6.837
AC-13-3-P1 3092021 R1
F
F
N
Ph
Boc
(S)-14
(er 99:1)
6.0
6.5
7.0
7.5
[min.]
Time

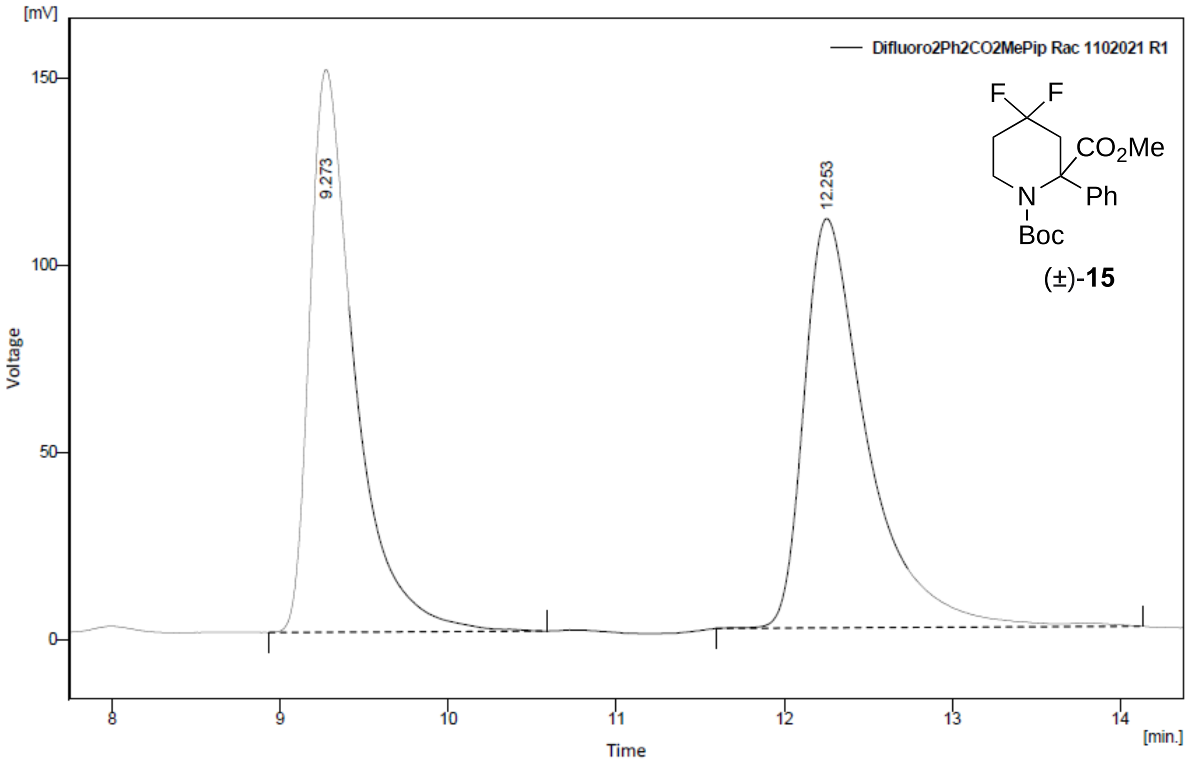
[mV]
Difluoro2Ph2CO2MePip Rac 1102021 R1
150
100
50
0
Voltage
9.273
12.253
8
9
10
11
12
13
14
[min.]
Time
F
F
CO2Me
N
Ph
Boc
(±)-15

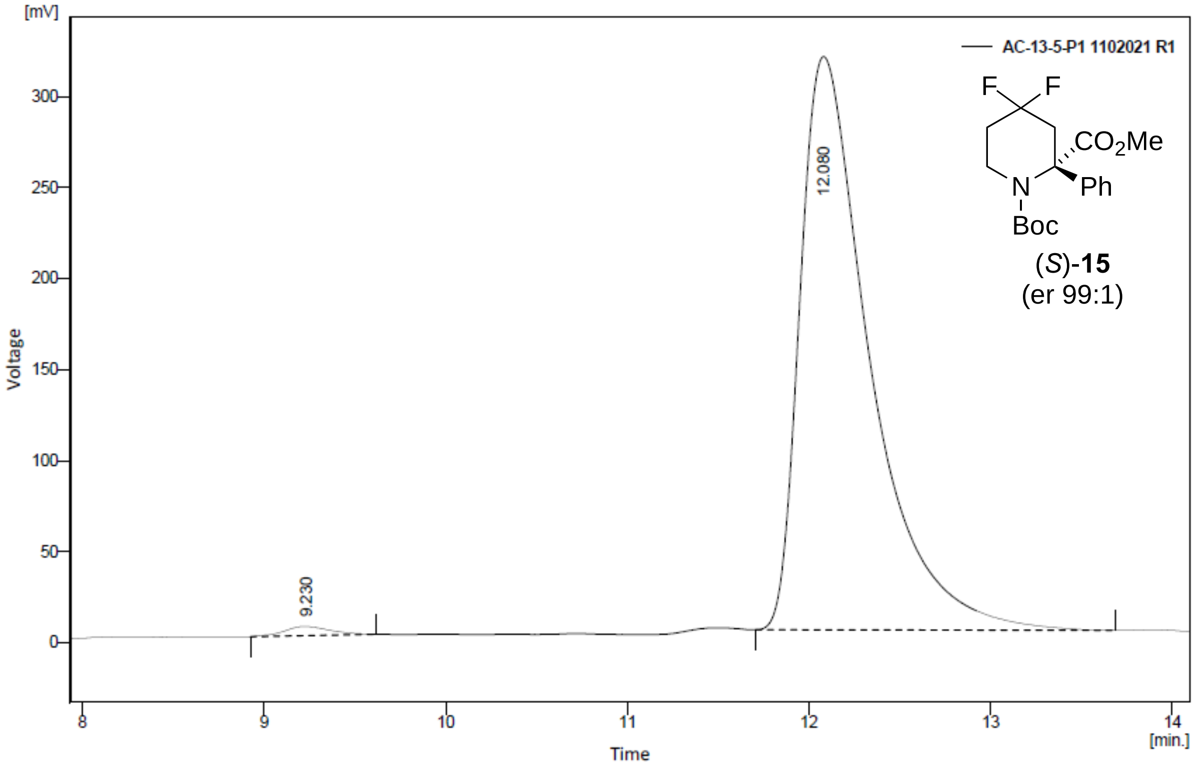
[mV]
AC-13-5-P1 1102021 R1
300
250
200
150
100
50
0
Voltage
9.230
12.080
8
9
10
11
12
13
14
[min.]
Time
F
F
CO2Me
N
Ph
Boc
(S)-15
(er 99:1)

6. X-ray data for ester (2*S*,4*R*)-13

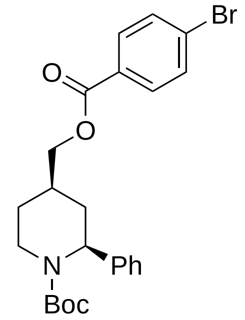

(2*S*,4*R*)-**13**

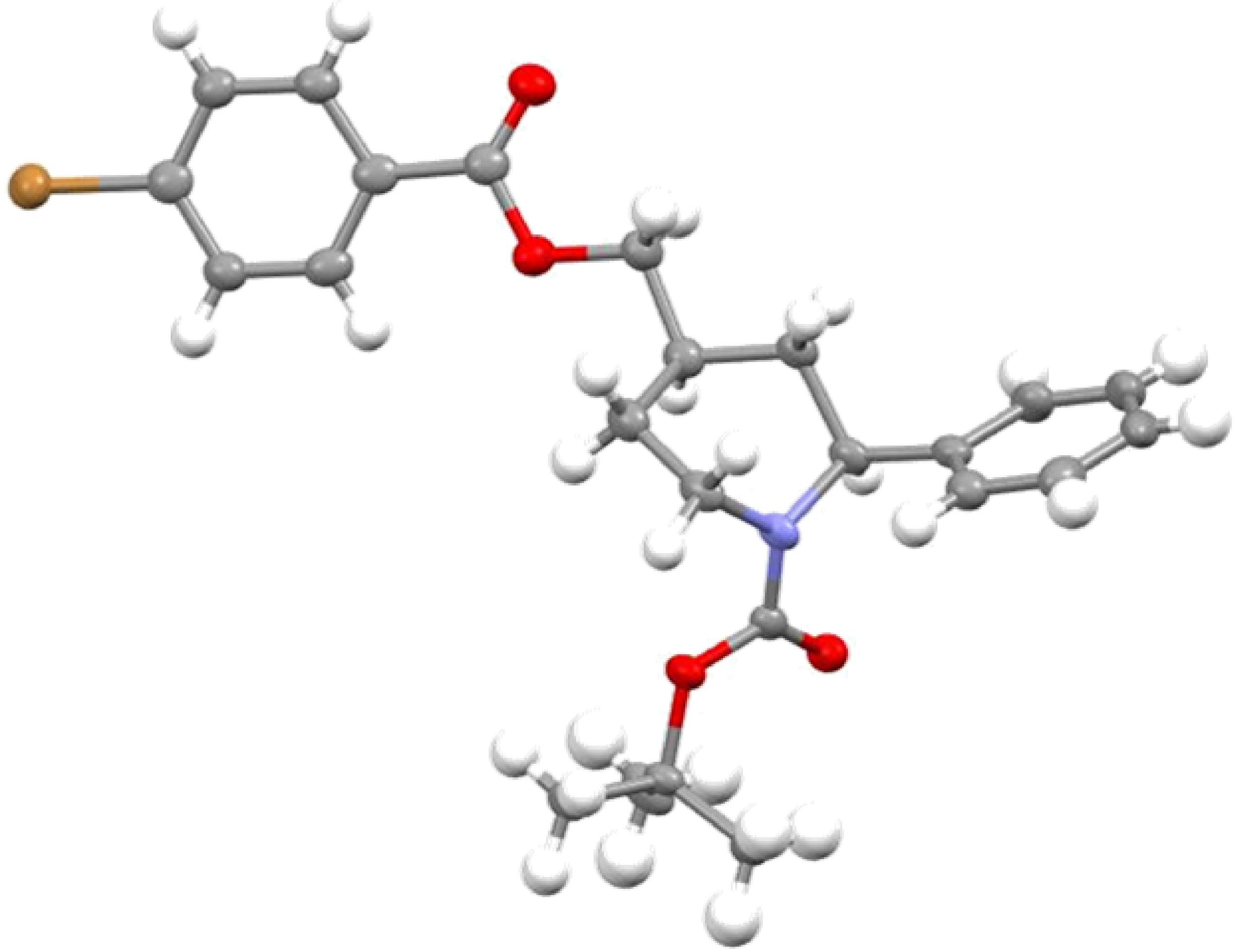

Thermal ellipsoid plot displayed at 50% probability level

CCDC 2155914

Compound (2*S*,4*R*)-**13** was prepared by recrystallization by slow evaporation in the solvent system CH_2Cl_2–hexane

Table S2 Crystal data and structure refinement for OIC323v_0m.

Identification code	OIC323v_0m
Empirical formula	$C_{24}H_{28}BrNO_4$
Formula weight	474.38
Temperature/K	99.99
Crystal system	monoclinic
Space group	C2
a/Å	17.0948(12)
b/Å	10.5262(7)
c/Å	13.2389(9)
α/°	90
β/°	106.728(3)
γ/°	90
Volume/Å^3	2281.4(3)
Z	4
ρ_{calc}g/cm^3	1.381
μ/mm^{-1}	2.694
F(000)	984.0
Crystal size/mm^3	0.2 × 0.19 × 0.14
Radiation	CuKα (λ = 1.54178)
2Θ range for data collection/°	6.972 to 133.964
Index ranges	$-20 \leq h \leq 20$, $-12 \leq k \leq 12$, $-15 \leq l \leq 15$
Reflections collected	41440
Independent reflections	4016 [R_{int} = 0.0517, R_{sigma} = 0.0256]
Data/restraints/parameters	4016/1/274
Goodness-of-fit on F^2	1.031
Final R indexes [I>=2σ (I)]	R_1 = 0.0246, wR_2 = 0.0581
Final R indexes [all data]	R_1 = 0.0258, wR_2 = 0.0588
Largest diff. peak/hole / e Å^{-3}	0.28/-0.27
Flack parameter	0.091(7)

Table S3 Fractional Atomic Coordinates (×10[4]) and Equivalent Isotropic Displacement Parameters (Å[2]×10[3]) for OIC323v_0m. U_{eq} is defined as 1/3 of of the trace of the orthogonalised U_{IJ} tensor.

Atom	*x*	*y*	*z*	U(eq)
Br1	3663.9(2)	-1713.9(4)	9528.9(2)	37.47(11)
O1	4410.2(15)	4379(2)	8341.0(19)	35.1(6)
O2	3544.1(15)	4802(3)	9289(2)	38.1(6)
O3	6231.4(13)	6765(2)	5112.1(17)	30.9(5)
O4	7000.0(13)	5363(2)	6306.5(16)	28.6(5)
N1	6307.0(16)	6864(3)	6854(2)	26.6(6)
C1	5615(2)	7752(3)	6661(3)	27.8(7)
C2	5103.1(19)	7412(3)	7411(2)	28.2(7)
C3	5043.2(19)	5981(3)	7542(2)	29.9(7)
C4	5894(2)	5427(4)	8077(3)	34.2(7)
C5	6560.2(19)	6290(3)	7906(2)	30.3(7)
C6	5864(2)	9138(4)	6779(2)	28.8(7)
C7	6646(2)	9537(4)	7330(3)	33.3(8)
C8	6825(2)	10828(4)	7470(3)	40.3(8)
C9	6231(3)	11714(4)	7057(3)	43.4(9)
C10	5451(3)	11328(4)	6501(3)	47.1(10)
C11	5270(2)	10058(4)	6367(3)	39.3(8)
C12	4448(2)	5727(3)	8170(3)	35.4(8)
C13	3924(2)	4036(4)	8941(3)	31.2(9)
C14	3911(2)	2643(4)	9098(3)	29.6(8)
C15	3458(2)	2178(4)	9753(3)	30.7(7)
C16	3398.2(19)	892(3)	9896(2)	30.5(7)
C17	3787(2)	61(3)	9386(2)	31.5(7)
C18	4255.8(19)	498(4)	8760(2)	30.7(7)
C19	4312(2)	1799(4)	8619(3)	31.7(8)
C20	6492.5(17)	6358(3)	6010(2)	25.0(6)
C21	7401.0(19)	4756(3)	5582(2)	27.7(7)
C22	6776(2)	4172(4)	4650(3)	43.9(9)
C23	7943(2)	5727(4)	5260(3)	40.7(8)
C24	7924.0(19)	3753(3)	6281(3)	29.5(7)

Table S4 Anisotropic Displacement Parameters (Å^2×10^3) for OIC323v_0m. The Anisotropic displacement factor exponent takes the form: -2π^2[h^{2}a*^{2}U$_{11}$+2hka*b*U$_{12}$+…].

Atom	U_{11}	U_{22}	U_{33}	U_{23}	U_{13}	U_{12}
Br1	39.98(18)	38.69(19)	33.44(16)	5.49(18)	10.07(12)	2.9(2)
O1	39.0(13)	33.2(15)	36.3(13)	-3.4(11)	16.2(11)	-4.0(11)
O2	39.5(14)	41.5(15)	34.6(13)	-3.6(11)	13.0(11)	3.5(11)
O3	29.4(11)	37.5(14)	25.9(11)	6.7(10)	8.0(9)	8.9(10)
O4	27.1(11)	33.9(13)	25.3(10)	3.4(9)	8.4(9)	10.5(9)
N1	19.4(13)	32.7(16)	24.6(13)	-0.9(11)	1.3(10)	7.5(11)
C1	20.9(16)	35.1(18)	24.6(16)	0.2(13)	2.1(13)	8.2(13)
C2	20.9(15)	33.9(19)	27.6(15)	-1.5(13)	3.4(12)	4.3(13)
C3	28.1(16)	33.4(19)	25.9(15)	-2.3(13)	4.0(13)	1.8(13)
C4	35.8(18)	38(2)	26.7(16)	4.3(14)	6.0(13)	9.4(15)
C5	24.5(15)	40(2)	20.1(14)	-2.0(14)	-2.6(12)	10.2(14)
C6	29.6(17)	35(2)	24.8(15)	3.0(14)	12.1(13)	9.1(15)
C7	33.1(18)	38(2)	29.0(17)	0.6(15)	8.7(14)	3.9(15)
C8	47(2)	41(2)	37.0(18)	-6.1(16)	18.5(16)	-7.9(17)
C9	63(3)	27.9(19)	52(2)	1.7(16)	38(2)	6.0(17)
C10	51(2)	39(2)	63(2)	17.1(19)	34(2)	17.8(18)
C11	30.9(18)	44(2)	46(2)	12.7(16)	15.3(15)	11.3(15)
C12	37.1(18)	31.5(19)	39.8(18)	-4.3(15)	14.5(15)	0.4(15)
C13	27.9(18)	39(2)	24.1(17)	-4.7(15)	2.9(14)	-2.4(16)
C14	22.1(17)	43(2)	19.6(16)	-4.5(14)	-0.9(13)	-2.1(14)
C15	25.5(16)	40(2)	24.7(16)	-3.6(14)	4.7(13)	0.7(14)
C16	26.2(16)	40(2)	22.6(15)	1.2(13)	3.2(12)	-0.2(14)
C17	25.1(16)	42(2)	21.8(15)	-0.4(13)	-1.7(12)	-0.5(14)
C18	27.0(16)	38(2)	26.1(16)	-1.1(13)	5.5(13)	3.1(14)
C19	23.4(16)	47(2)	23.1(16)	-2.4(15)	3.8(13)	-3.5(15)
C20	18.5(14)	27.4(17)	28.4(16)	-0.6(13)	5.5(12)	0.9(12)
C21	25.8(15)	32.5(17)	25.0(15)	-1.6(13)	7.7(12)	7.9(13)
C22	37.6(19)	47(2)	36.6(18)	-9.5(17)	-5.7(15)	13.4(18)
C23	37.6(19)	39(2)	52(2)	12.0(17)	22.6(17)	10.9(16)
C24	26.2(16)	32.4(17)	27.8(15)	0.6(12)	4.2(13)	7.7(12)

Table S5 Bond Lengths for OIC323v_0m.

Atom	Atom	Length/Å	Atom	Atom	Length/Å
Br1	C17	1.896(4)	C6	C7	1.392(5)
O1	C12	1.442(5)	C6	C11	1.395(5)
O1	C13	1.353(5)	C7	C8	1.393(5)
O2	C13	1.207(5)	C8	C9	1.372(6)
O3	C20	1.221(4)	C9	C10	1.386(6)
O4	C20	1.345(4)	C10	C11	1.372(6)
O4	C21	1.475(4)	C13	C14	1.482(5)
N1	C1	1.472(4)	C14	C15	1.406(5)
N1	C5	1.464(4)	C14	C19	1.383(5)
N1	C20	1.355(4)	C15	C16	1.374(5)
C1	C2	1.542(5)	C16	C17	1.387(5)
C1	C6	1.515(5)	C17	C18	1.385(5)
C2	C3	1.523(5)	C18	C19	1.390(5)
C3	C4	1.537(4)	C21	C22	1.511(5)
C3	C12	1.512(5)	C21	C23	1.522(5)
C4	C5	1.525(5)	C21	C24	1.514(4)

Table S6 Bond Angles for OIC323v_0m.

Atom	Atom	Atom	Angle/°	Atom	Atom	Atom	Angle/°
C13	O1	C12	114.6(3)	O1	C13	C14	112.5(4)
C20	O4	C21	122.0(2)	O2	C13	O1	122.4(4)
C5	N1	C1	115.5(3)	O2	C13	C14	125.1(4)
C20	N1	C1	118.3(3)	C15	C14	C13	117.7(4)
C20	N1	C5	123.2(3)	C19	C14	C13	122.8(4)
N1	C1	C2	108.5(3)	C19	C14	C15	119.5(4)
N1	C1	C6	114.0(3)	C16	C15	C14	120.3(3)
C6	C1	C2	110.6(3)	C15	C16	C17	119.3(3)
C3	C2	C1	111.8(3)	C16	C17	Br1	119.4(3)
C2	C3	C4	110.0(3)	C18	C17	Br1	119.1(3)
C12	C3	C2	108.4(3)	C18	C17	C16	121.5(3)
C12	C3	C4	112.1(3)	C17	C18	C19	118.8(3)
C5	C4	C3	110.7(3)	C14	C19	C18	120.6(3)
N1	C5	C4	111.0(2)	O3	C20	O4	125.6(3)
C7	C6	C1	122.9(3)	O3	C20	N1	123.9(3)
C7	C6	C11	118.5(4)	O4	C20	N1	110.5(3)
C11	C6	C1	118.5(3)	O4	C21	C22	110.8(3)
C6	C7	C8	120.3(3)	O4	C21	C23	109.0(3)
C9	C8	C7	120.1(4)	O4	C21	C24	102.0(2)
C8	C9	C10	120.1(4)	C22	C21	C23	112.9(3)
C11	C10	C9	120.1(3)	C22	C21	C24	111.7(3)
C10	C11	C6	121.0(4)	C24	C21	C23	109.9(3)
O1	C12	C3	109.1(3)				

Table S7 Torsion Angles for OIC323v_0m.

A	B	C	D	Angle/˚	A	B	C	D	Angle/˚
Br1	C17	C18	C19	177.0(2)	C7	C6	C11	C10	-0.1(5)
O1	C13	C14	C15	176.9(3)	C7	C8	C9	C10	0.0(5)
O1	C13	C14	C19	-4.0(5)	C8	C9	C10	C11	0.5(6)
O2	C13	C14	C15	-3.8(6)	C9	C10	C11	C6	-0.4(6)
O2	C13	C14	C19	175.2(3)	C11	C6	C7	C8	0.5(5)
N1	C1	C2	C3	-38.4(4)	C12	O1	C13	O2	1.1(5)
N1	C1	C6	C7	-19.4(5)	C12	O1	C13	C14	-179.6(3)
N1	C1	C6	C11	164.3(3)	C12	C3	C4	C5	-144.4(3)
C1	N1	C5	C4	65.7(4)	C13	O1	C12	C3	177.0(3)
C1	N1	C20	O3	14.6(5)	C13	C14	C15	C16	177.5(3)
C1	N1	C20	O4	-165.6(3)	C13	C14	C19	C18	-177.6(4)
C1	C2	C3	C4	64.2(3)	C14	C15	C16	C17	-0.1(5)
C1	C2	C3	C12	-172.9(3)	C15	C14	C19	C18	1.5(5)
C1	C6	C7	C8	-175.8(3)	C15	C16	C17	Br1	-177.1(2)
C1	C6	C11	C10	176.4(3)	C15	C16	C17	C18	2.0(5)
C2	C1	C6	C7	103.2(3)	C16	C17	C18	C19	-2.1(5)
C2	C1	C6	C11	-73.1(4)	C17	C18	C19	C14	0.3(5)
C2	C3	C4	C5	-23.7(4)	C19	C14	C15	C16	-1.5(5)
C2	C3	C12	O1	-177.5(3)	C20	O4	C21	C22	-63.5(4)
C3	C4	C5	N1	-36.4(4)	C20	O4	C21	C23	61.4(4)
C4	C3	C12	O1	-55.9(4)	C20	O4	C21	C24	177.5(3)
C5	N1	C1	C2	-24.8(4)	C20	N1	C1	C2	135.9(3)
C5	N1	C1	C6	98.9(4)	C20	N1	C1	C6	-100.4(4)
C5	N1	C20	O3	173.8(3)	C20	N1	C5	C4	-94.1(4)
C5	N1	C20	O4	-6.4(4)	C21	O4	C20	O3	9.4(5)
C6	C1	C2	C3	-164.2(3)	C21	O4	C20	N1	-170.4(3)
C6	C7	C8	C9	-0.5(5)					

Table S8 Hydrogen Atom Coordinates (Å×10[4]) and Isotropic Displacement Parameters (Å[2]×10[3]) for OIC323v_0m.

Atom	*x*	*y*	*z*	U(eq)
H1	5264.26	7619.31	5921.71	33
H2A	5355.13	7802.75	8109.5	34
H2B	4547	7769.29	7128.51	34
H3	4826.76	5587.04	6828.66	36
H4A	5966.3	5331.59	8842.88	41
H4B	5939.45	4574.98	7782.93	41
H5A	6681.6	6969.41	8446.08	36
H5B	7065.29	5790.89	7987.42	36
H7	7060.92	8926.7	7611.82	40
H8	7359.41	11095.23	7851.6	48
H9	6353.67	12594.01	7153.66	52
H10	5041.06	11943.28	6210.96	56
H11	4732.97	9801.27	5988.24	47
H12A	4628.33	6173.84	8856.64	43
H12B	3899.62	6048.77	7781.26	43
H15	3192.43	2754.69	10097.66	37
H16	3093.45	576.65	10339.07	37
H18	4533.52	-82.28	8434.33	37
H19	4629.29	2111.21	8188.97	38
H22A	6412.9	3615.13	4902.92	66
H22B	7055.29	3676	4231.82	66
H22C	6454.24	4847.3	4212.02	66
H23A	7601.71	6372.53	4806.86	61
H23B	8278.71	5300.73	4873.74	61
H23C	8299.18	6134.83	5891.68	61
H24A	8317.06	4164.2	6880.03	44
H24B	8218.59	3268.42	5874.34	44
H24C	7574.77	3178.06	6541.82	44

Crystal structure determination of OIC323v_0m

Crystal Data for $C_{24}H_{28}BrNO_4$ (M =474.38 g/mol): monoclinic, space group C2 (no. 5), a = 17.0948(12) Å, b = 10.5262(7) Å, c = 13.2389(9) Å, β = 106.728(3)°, V = 2281.4(3) Å^3, Z = 4, T = 99.99 K, μ(CuKα) = 2.694 mm^{-1}, $Dcalc$ = 1.381 g/cm^3, 41440 reflections measured (6.972° ≤ 2Θ ≤ 133.964°), 4016 unique (R_{int} = 0.0517, R_{sigma} = 0.0256) which were used in all calculations. The final R_1 was 0.0246 (I > 2σ(I)) and wR_2 was 0.0588 (all data).

7. Variable temperature ^{1}H NMR spectra for alkene 3a

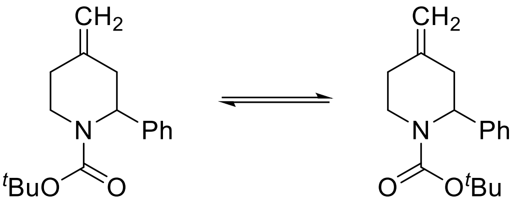

Coalescence of signals in the ^{1}H NMR spectrum of alkene **3a** in D_8-THF was followed by taking spectra at various temperatures. The ^{1}H NMR spectra in the region 6.0–3.7 ppm are shown below:

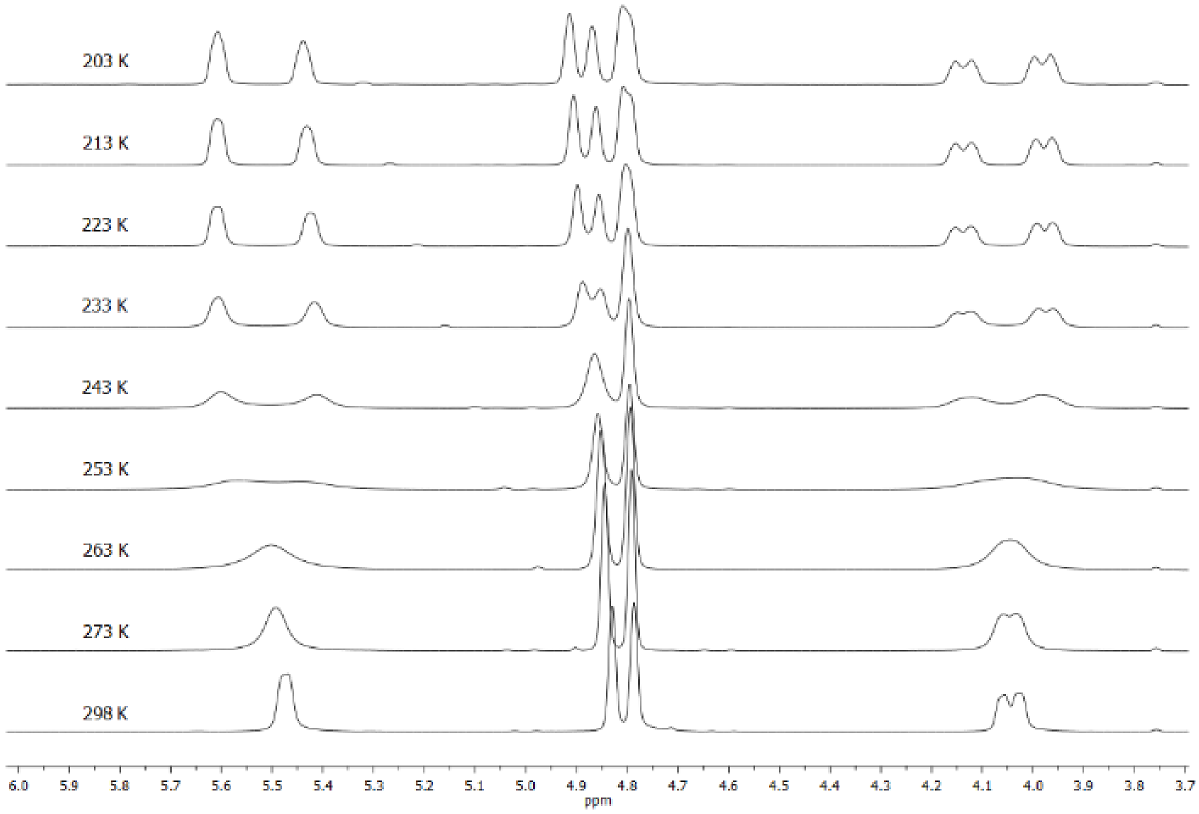

The ratio of rotamers is ~1.2 : 1.

Assuming an approximately equal rate and using line shape analysis (DNMR with the software iNMR), the following rate data can be estimated:

Table S-9. Estimated rate constants from VT-NMR spectroscopy of carbamate **3a**.

T/K	1/T	*k*	ln(*k*/T)
203	0.004926	0.1	-7.61579107
213	0.004695	1	-5.36129217
223	0.004484	3	-4.30855948
233	0.004292	12	-2.9661318
243	0.004115	30	-2.09186406
253	0.003953	110	-0.83290912
263	0.003802	210	-0.2250465
273	0.003663	550	0.700446483
283	0.003534	1500	1.667773489
298	0.003356	3500	2.463424761

Eyring plot:

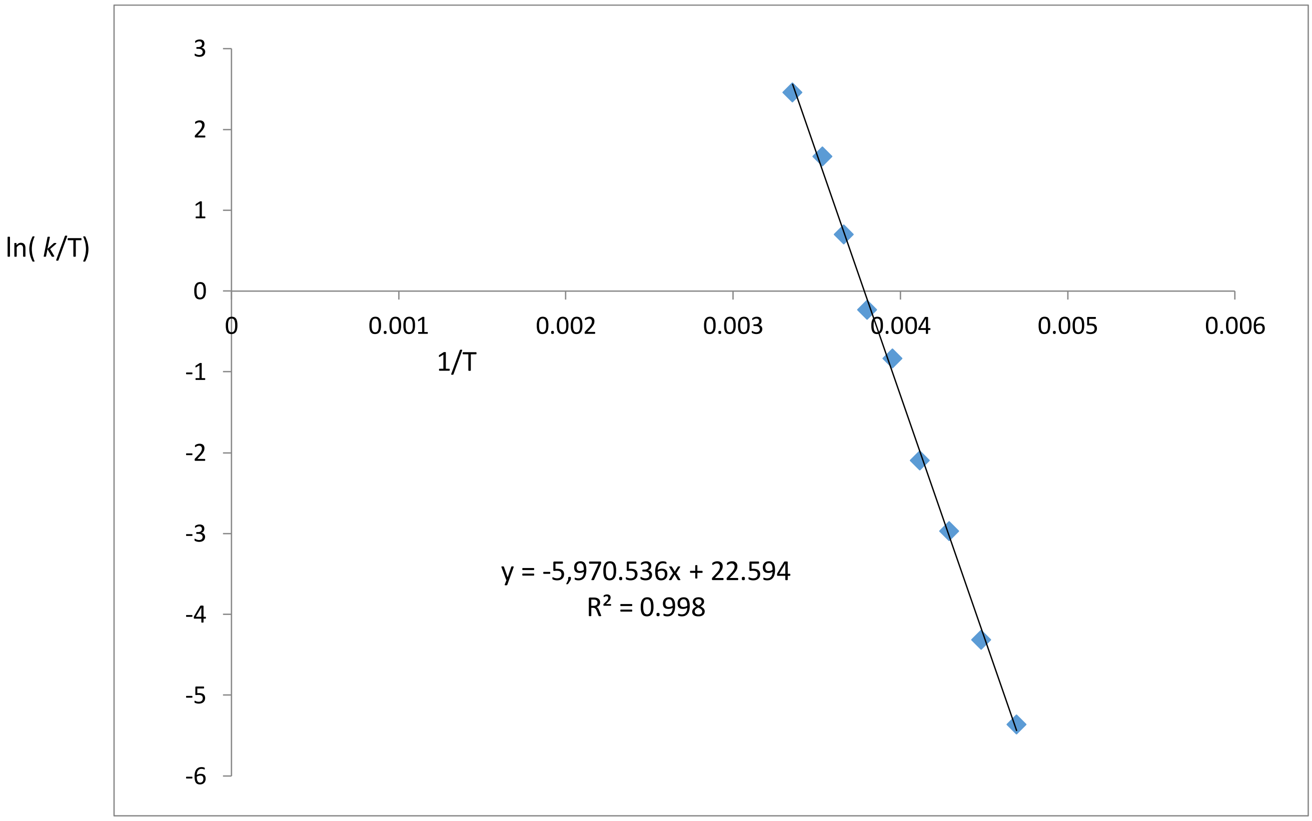

By using the Eyring equation, this gave the following data:

slope –5970.54, intercept 22.59

Approximate activation parameters for Boc rotation in THF:

$\Delta H^{\ddagger} \approx 49.6$ kJ/mol

$\Delta S^{\ddagger} \approx -9.7$ J/K·mol.

Hence the barrier to rotation $\Delta G^{\ddagger} \approx 51.5$ kJ/mol at –78 °C.

The half-life for rotation is about 11 sec at –78 °C.

8. DFT Data

DFT calculations on the rotamers of piperidine **3a** were carried out using the methods described in the general information. Two conformations of piperidine **3a** were considered where the phenyl group could be axial or equatorial. The minimal energy structures for the rotamers of piperidine **3a** with an axial phenyl group are shown in Figure 1a,c. Of these two structures the rotamer in which the carbonyl group of the Boc group is pointing towards the benzylic position (C-2) was lower in thermal energy by 170 $Jmol^{-1}$. From these structures the lowest energy transition state for rotation of the Boc group was obtained (Figure 1b) where the Gibbs energy of activation was calculated to be ~77 $kJmol^{-1}$ at –78 °C.

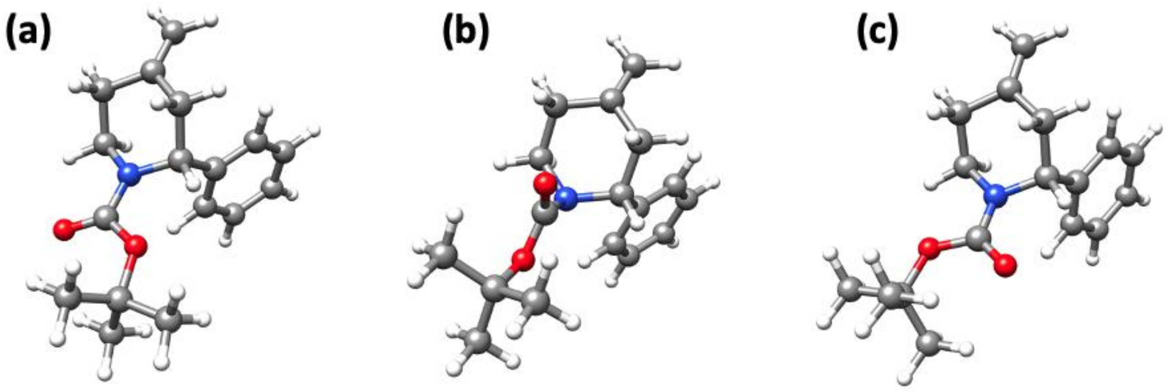

Figure 1

In contrast, the minimal energy structures for the rotamers of piperidine **3a** with an equatorial phenyl group are shown in Figure 2a,c. As expected, based on $A_{1,3}$ strain considerations these structures were higher in energy then their corresponding axial rotamers, however the rotamer in which the carbonyl group of the Boc group is pointing towards the benzylic position (C-2) was now higher in thermal energy by 6.3 $kJmol^{-1}$. The Gibbs energy of activation for rotation of the Boc group through the lowest energy transition state (Figure 2b) was calculated to be ~34 $kJmol^{-1}$ at –78 °C. Although further optimization of the rotamers in Figure 2a,c gave lower energy structures, the lowest energy transition state obtained for rotation of the Boc group from these structures was much higher in energy when compared to the other transition states calculated. Therefore, these structures were not considered any further.

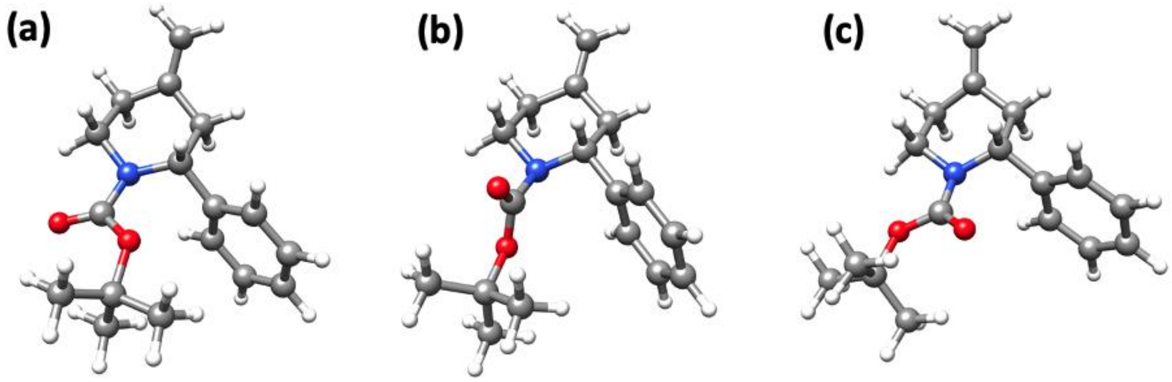

Figure 2

With the phenyl group axial in piperidine **3a** we found when comparing this to the lowest energy equatorial transition state for rotation of the Boc group the Gibbs energy of activation was calculated to be ~55 $kJmol^{-1}$ at –78 °C (with $\Delta H \approx 43$ $kJmol^{-1}$ and $\Delta S \approx -63$ $JK^{-1}mol^{-1}$). This value matched well with the results obtained from VT-NMR and indicated that the rate of ring flipping between the axial and equatorial positions of the phenyl group was fast along with the rotation of the Boc group under the reaction conditions.

Below are summaries for optimised structures and transition states obtained from the calculations performed. Thermochemistry data at 298 K, 223 K and 195 K has also been provided as well.

Left Rotamer Axial

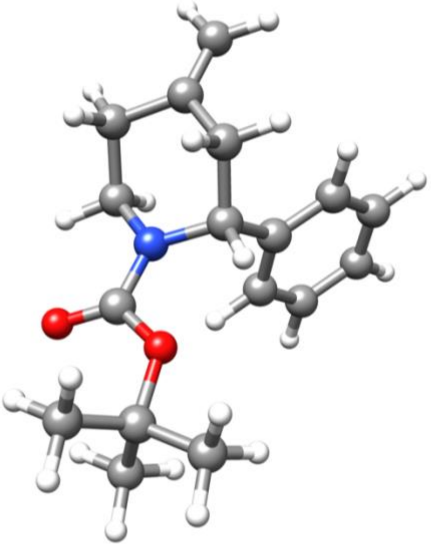

Route : # opt freq b3lyp scrf=(solvent=thf) geom=connectivity def2tzvp empiricaldispersion=gd3bj int=ultrafine pop=(regular,mk)

SMILES : C=C2CCN(C(=O)OC(C)(C)C)C(c1ccccc1)C2

Formula : $C_{17}H_{23}NO_2$

Charge : 0

Multiplicity : 1

Dipole : 2.6574 Debye

Energy : -867.300243526 a.u.

Gibbs Energy : -866.979530 a.u.

Number of imaginary frequencies : 0

Cartesian Coordinates (XYZ format)

43

C	-1.41379400	-1.48908400	1.39336000
C	-0.64103300	-0.33635800	0.71920700
C	-0.64043300	-1.61694300	-1.40517300
C	-1.42477200	-2.76659000	-0.76582800
H	0.07834400	0.04029900	1.44046200
H	-0.66022700	-2.14732100	1.83644400
H	-2.02984700	-1.11639700	2.20891000
H	-1.33666300	-0.92697700	-1.89147400
H	0.05359000	-1.98355700	-2.15380300
H	-2.06760000	-3.23185000	-1.51396400
H	-0.70914400	-3.52084300	-0.42095200
C	-2.22815300	-2.27127500	0.40337600
C	-3.53518400	-2.47520300	0.53201400
H	-4.08764600	-2.08981900	1.38040800
H	-4.09436900	-3.03763500	-0.20609400
N	0.12622500	-0.89038000	-0.39924400

C	1.46011600	-0.68835500	-0.58630300
O	2.08870400	-1.12864600	-1.53596800
O	1.98784600	0.05600000	0.40436800
C	3.40317300	0.45464200	0.40959200
C	4.29797600	-0.77654600	0.49413500
H	4.23784500	-1.37079000	-0.41346500
H	5.33172900	-0.45977600	0.64053400
H	4.00809800	-1.39535600	1.34477300
C	3.70726000	1.31568200	-0.81116700
H	4.71556700	1.72258500	-0.72194900
H	3.64273800	0.73672500	-1.72840600
H	3.00670400	2.15039000	-0.86663100
C	3.50575500	1.28083200	1.68585500
H	3.24637200	0.67627200	2.55560000
H	4.52525000	1.64658700	1.80927200
H	2.83200700	2.13709900	1.64260800
C	-1.49911400	0.84544000	0.27335000
C	-1.05462200	1.67357100	-0.75863900
C	-2.69588100	1.17320200	0.90936700
C	-1.78234300	2.79118500	-1.14659700
H	-0.13103400	1.44075100	-1.27060300
C	-3.42752600	2.29198100	0.52474100
H	-3.07455100	0.55773200	1.71192700
C	-2.97553700	3.10604600	-0.50599500
H	-1.41600800	3.41567900	-1.95144400
H	-4.35587600	2.52235700	1.03176100
H	-3.54656600	3.97431500	-0.80817700

Frequencies

Mode	IR frequency	IR intensity	Raman intensity
1	14.16070000	0.02060000	0.00000000
2	19.09830000	0.17990000	0.00000000
3	34.18630000	0.01750000	0.00000000
4	59.89320000	4.61870000	0.00000000
5	68.74770000	0.27250000	0.00000000
6	100.20840000	0.47870000	0.00000000
7	107.13920000	0.23520000	0.00000000
8	124.02560000	1.12160000	0.00000000
9	160.17790000	0.17590000	0.00000000
10	199.02450000	1.17170000	0.00000000
11	204.41360000	0.22210000	0.00000000
12	219.00280000	8.03990000	0.00000000
13	241.93030000	0.04140000	0.00000000
14	271.52830000	0.06880000	0.00000000
15	275.83610000	3.09010000	0.00000000
16	306.93210000	0.37410000	0.00000000
17	330.05430000	3.81040000	0.00000000
18	340.62590000	12.67090000	0.00000000

19	351.09040000	1.95050000	0.00000000
20	384.15790000	10.95380000	0.00000000
21	410.14060000	1.13510000	0.00000000
22	413.73130000	3.57770000	0.00000000
23	415.40020000	0.07740000	0.00000000
24	436.04500000	1.64770000	0.00000000
25	459.74400000	3.71200000	0.00000000
26	469.28450000	15.86460000	0.00000000
27	483.41550000	1.45780000	0.00000000
28	511.69800000	0.80150000	0.00000000
29	542.05550000	9.22950000	0.00000000
30	623.94120000	7.78990000	0.00000000
31	638.25710000	1.03310000	0.00000000
32	664.64230000	21.36010000	0.00000000
33	712.81940000	54.31570000	0.00000000
34	723.29660000	18.21150000	0.00000000
35	743.44520000	12.23450000	0.00000000
36	766.15980000	7.17110000	0.00000000
37	771.72180000	17.59310000	0.00000000
38	776.37560000	26.19100000	0.00000000
39	786.15690000	12.90970000	0.00000000
40	812.77920000	16.82260000	0.00000000
41	840.54860000	5.75630000	0.00000000
42	857.51940000	0.67370000	0.00000000
43	867.35760000	47.70610000	0.00000000
44	917.36150000	27.17080000	0.00000000
45	928.21160000	39.30960000	0.00000000
46	929.49550000	3.79850000	0.00000000
47	930.79810000	0.14670000	0.00000000
48	939.92590000	13.87540000	0.00000000
49	963.59740000	20.37220000	0.00000000
50	972.60480000	0.10580000	0.00000000
51	979.39580000	0.37410000	0.00000000
52	994.50530000	1.08140000	0.00000000
53	1007.37460000	21.82210000	0.00000000
54	1014.22440000	79.32760000	0.00000000
55	1025.10860000	4.61930000	0.00000000
56	1029.52630000	60.32650000	0.00000000
57	1052.70300000	0.67880000	0.00000000
58	1055.51190000	9.62660000	0.00000000
59	1059.16300000	0.95600000	0.00000000
60	1070.39220000	10.22900000	0.00000000
61	1115.02270000	21.65620000	0.00000000

62	1126.69820000	183.51340000	0.00000000
63	1169.18920000	70.52510000	0.00000000
64	1181.97660000	6.71480000	0.00000000
65	1185.39030000	416.03820000	0.00000000
66	1198.72450000	56.29080000	0.00000000
67	1211.07760000	5.65180000	0.00000000
68	1223.71120000	17.14680000	0.00000000
69	1248.58540000	4.27730000	0.00000000
70	1269.20150000	96.09440000	0.00000000
71	1269.83470000	86.09100000	0.00000000
72	1284.04360000	36.02090000	0.00000000
73	1292.64790000	97.78100000	0.00000000
74	1305.65780000	12.79180000	0.00000000
75	1325.22680000	20.97300000	0.00000000
76	1347.77640000	160.69840000	0.00000000
77	1354.84960000	7.78100000	0.00000000
78	1364.27420000	0.18930000	0.00000000
79	1378.87620000	16.93550000	0.00000000
80	1396.25500000	31.37290000	0.00000000
81	1396.87640000	28.31070000	0.00000000
82	1401.19940000	16.17020000	0.00000000
83	1423.04640000	18.01430000	0.00000000
84	1444.24550000	393.77190000	0.00000000
85	1456.82030000	25.68590000	0.00000000
86	1466.68250000	0.20610000	0.00000000
87	1473.28380000	4.81720000	0.00000000
88	1482.76110000	11.02400000	0.00000000
89	1484.26350000	1.61930000	0.00000000
90	1484.35710000	1.59170000	0.00000000
91	1486.94800000	25.93270000	0.00000000
92	1492.10110000	29.79960000	0.00000000
93	1497.60620000	2.46160000	0.00000000
94	1499.56730000	19.29340000	0.00000000
95	1517.34740000	32.81390000	0.00000000
96	1535.33650000	19.18150000	0.00000000
97	1625.44980000	0.39220000	0.00000000
98	1646.23830000	11.23140000	0.00000000
99	1701.82210000	797.65440000	0.00000000
100	1717.00020000	17.29430000	0.00000000
101	3011.92360000	20.09000000	0.00000000
102	3026.63370000	28.21360000	0.00000000
103	3029.12930000	76.73210000	0.00000000
104	3041.44740000	15.45400000	0.00000000

105	3043.10580000	39.69190000	0.00000000
106	3049.98520000	20.64360000	0.00000000
107	3086.55710000	44.81620000	0.00000000
108	3102.06480000	8.04560000	0.00000000
109	3104.19370000	30.62440000	0.00000000
110	3109.71890000	25.64920000	0.00000000
111	3111.08380000	45.13500000	0.00000000
112	3117.60990000	64.10510000	0.00000000
113	3120.69660000	8.14510000	0.00000000
114	3131.75470000	14.54570000	0.00000000
115	3144.88970000	0.65150000	0.00000000
116	3147.99200000	24.36750000	0.00000000
117	3152.11910000	6.65550000	0.00000000
118	3169.80960000	0.56760000	0.00000000
119	3177.90180000	11.67750000	0.00000000
120	3190.57040000	32.40370000	0.00000000
121	3198.42780000	21.89150000	0.00000000
122	3210.45420000	8.79120000	0.00000000
123	3211.23580000	26.50140000	0.00000000

Right Rotamer Axial

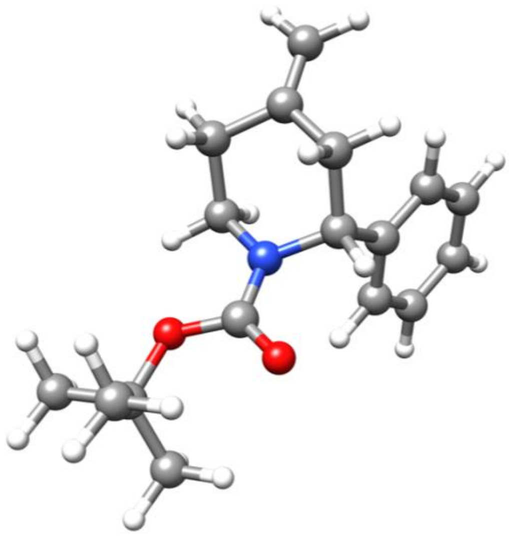

Route : # opt freq b3lyp scrf=(solvent=thf) geom=connectivity def2tzvp empiricaldispersion=gd3bj int=ultrafine pop=(regular,mk)

SMILES : C=C2CCN(C(=O)OC(C)(C)C)C(c1ccccc1)C2

Formula : $C_{17}H_{23}NO_2$

Charge : 0

Multiplicity : 1

Dipole : 2.7968 Debye

Energy : -867.300166083 a.u.

Gibbs Energy : -866.983065 a.u.

Number of imaginary frequencies : 0

Cartesian Coordinates (XYZ format)

43

C	-1.42919500	-1.73875000	1.22136800
C	-0.97148400	-0.29229000	0.94757600
C	-0.01570300	-1.00933700	-1.21443600
C	-0.45123400	-2.46108100	-0.98477300
H	-0.61807700	0.13212800	1.88510700
H	-0.63129600	-2.20655900	1.80643300
H	-2.32517600	-1.75190300	1.83972300
H	-0.79692400	-0.47575500	-1.76281400
H	0.89675500	-0.96334300	-1.79584200
H	-0.69064500	-2.93122000	-1.93943700
H	0.39287400	-3.00406200	-0.54533000
C	-1.62615500	-2.52125600	-0.04786400
C	-2.74194100	-3.18733600	-0.32735200
H	-3.57875700	-3.20563200	0.36050000

H	-2.85300700	-3.74048700	-1.25225500
N	0.19345100	-0.33402400	0.06151300
C	1.39189700	0.12881600	0.51006900
O	1.54994100	0.66046700	1.59884800
O	2.36740100	-0.06550700	-0.39718200
C	3.74644400	0.38828000	-0.16084500
C	3.78107200	1.90425400	-0.00283400
H	3.28543300	2.21736600	0.91203500
H	4.81921600	2.23843400	0.02692200
H	3.29386100	2.38296800	-0.85377000
C	4.33967300	-0.33763400	1.04131800
H	5.40093500	-0.09677900	1.11858100
H	3.84470000	-0.04375600	1.96278000
H	4.24410400	-1.41714300	0.91431600
C	4.45505600	-0.03220700	-1.44265400
H	4.00414000	0.45514500	-2.30769500
H	5.50673300	0.25084100	-1.39392500
H	4.39240100	-1.11239500	-1.57849800
C	-2.03735700	0.64445800	0.39114200
C	-1.63956100	1.86550100	-0.15840700
C	-3.39964100	0.36027300	0.44997800
C	-2.57314800	2.77429900	-0.63647000
H	-0.58549300	2.10354700	-0.21396600
C	-4.33953500	1.26900400	-0.02859900
H	-3.74489900	-0.57577400	0.86200700
C	-3.93158300	2.47844100	-0.57473100
H	-2.24046600	3.71467300	-1.05727500
H	-5.39277900	1.02405400	0.02311600
H	-4.66215900	3.18353000	-0.94955000

Frequencies

Mode	IR frequency	IR intensity	Raman intensity
1	2.45010000	0.07770000	0.00000000
2	4.31770000	0.05220000	0.00000000
3	26.95830000	0.06870000	0.00000000
4	53.99790000	5.22180000	0.00000000
5	73.90260000	0.66050000	0.00000000
6	98.47940000	0.52890000	0.00000000
7	103.36100000	0.49950000	0.00000000
8	122.69100000	0.67640000	0.00000000
9	157.64960000	0.79010000	0.00000000
10	199.43650000	0.32700000	0.00000000
11	203.57420000	0.15290000	0.00000000
12	236.83350000	0.94040000	0.00000000
13	243.56010000	12.01890000	0.00000000
14	250.64830000	1.38880000	0.00000000
15	268.76940000	0.06440000	0.00000000
16	288.92720000	0.56290000	0.00000000
17	331.96410000	18.47730000	0.00000000

18	337.81910000	6.86540000	0.00000000
19	349.67860000	1.18140000	0.00000000
20	380.21470000	3.05640000	0.00000000
21	407.79230000	1.55030000	0.00000000
22	414.28380000	0.31150000	0.00000000
23	418.26190000	1.31320000	0.00000000
24	452.47170000	5.63920000	0.00000000
25	459.53920000	2.92150000	0.00000000
26	474.37770000	13.05360000	0.00000000
27	486.88570000	1.12660000	0.00000000
28	512.08290000	0.38750000	0.00000000
29	562.59240000	11.62690000	0.00000000
30	621.37590000	15.77720000	0.00000000
31	637.50680000	0.78950000	0.00000000
32	652.22480000	16.04450000	0.00000000
33	712.09610000	61.92540000	0.00000000
34	720.48630000	8.98440000	0.00000000
35	737.42060000	20.79820000	0.00000000
36	755.92130000	5.29830000	0.00000000
37	774.52910000	33.13490000	0.00000000
38	781.99990000	14.18240000	0.00000000
39	782.51300000	4.49550000	0.00000000
40	812.43830000	14.82340000	0.00000000
41	839.78000000	4.26130000	0.00000000
42	856.20450000	0.27180000	0.00000000
43	870.43770000	63.66860000	0.00000000
44	916.96660000	28.57370000	0.00000000
45	927.49790000	34.75110000	0.00000000
46	929.11700000	3.70030000	0.00000000
47	929.61990000	0.27020000	0.00000000
48	934.04640000	10.78130000	0.00000000
49	963.14540000	9.97930000	0.00000000
50	971.41980000	0.10420000	0.00000000
51	977.05430000	0.19100000	0.00000000
52	992.55190000	0.65020000	0.00000000
53	1003.39320000	19.11090000	0.00000000
54	1013.07010000	60.33330000	0.00000000
55	1023.33920000	83.27710000	0.00000000
56	1026.77890000	50.37930000	0.00000000
57	1052.09840000	1.18770000	0.00000000
58	1053.76880000	9.23390000	0.00000000
59	1058.26650000	2.11370000	0.00000000
60	1066.97280000	1.01460000	0.00000000

61	1110.95620000	29.30920000	0.00000000
62	1131.09720000	182.01100000	0.00000000
63	1167.27670000	29.86450000	0.00000000
64	1180.89360000	10.63670000	0.00000000
65	1183.79790000	546.75020000	0.00000000
66	1206.41480000	14.33350000	0.00000000
67	1209.89080000	1.83440000	0.00000000
68	1222.95910000	21.70410000	0.00000000
69	1247.87770000	12.23390000	0.00000000
70	1265.99330000	179.91710000	0.00000000
71	1268.80260000	22.79600000	0.00000000
72	1284.58240000	42.07890000	0.00000000
73	1287.88590000	54.44890000	0.00000000
74	1304.88460000	10.82540000	0.00000000
75	1331.01430000	10.30370000	0.00000000
76	1348.34690000	151.03810000	0.00000000
77	1354.74240000	3.42020000	0.00000000
78	1364.77030000	1.78950000	0.00000000
79	1379.64700000	14.17490000	0.00000000
80	1395.82840000	28.40310000	0.00000000
81	1396.54330000	44.12880000	0.00000000
82	1400.73470000	13.64270000	0.00000000
83	1421.46170000	19.49900000	0.00000000
84	1447.57240000	417.17350000	0.00000000
85	1456.00790000	35.17910000	0.00000000
86	1465.84070000	0.15340000	0.00000000
87	1471.82940000	8.96120000	0.00000000
88	1483.18840000	11.06640000	0.00000000
89	1483.81090000	2.04970000	0.00000000
90	1483.90290000	0.21040000	0.00000000
91	1485.98480000	22.98670000	0.00000000
92	1490.06320000	26.06210000	0.00000000
93	1496.67560000	2.24090000	0.00000000
94	1501.85860000	21.90430000	0.00000000
95	1516.36830000	42.17840000	0.00000000
96	1535.33810000	17.56570000	0.00000000
97	1626.21340000	1.69160000	0.00000000
98	1646.81110000	8.02510000	0.00000000
99	1696.53820000	732.21270000	0.00000000
100	1715.10180000	21.48700000	0.00000000
101	3010.87800000	22.43100000	0.00000000
102	3026.86340000	33.27280000	0.00000000
103	3034.47150000	59.24340000	0.00000000

104	3040.86340000	16.23940000	0.00000000
105	3043.25430000	40.90710000	0.00000000
106	3049.84740000	22.12720000	0.00000000
107	3084.59440000	45.71090000	0.00000000
108	3100.16260000	5.36240000	0.00000000
109	3102.25590000	6.58280000	0.00000000
110	3102.87160000	43.41560000	0.00000000
111	3103.63150000	27.14860000	0.00000000
112	3110.68590000	46.57890000	0.00000000
113	3117.08330000	61.35300000	0.00000000
114	3131.29930000	14.58860000	0.00000000
115	3144.86150000	2.56480000	0.00000000
116	3148.03850000	24.01270000	0.00000000
117	3169.12410000	0.72100000	0.00000000
118	3171.33050000	6.95070000	0.00000000
119	3177.04790000	9.38000000	0.00000000
120	3187.88440000	31.52680000	0.00000000
121	3195.71500000	26.22850000	0.00000000
122	3210.00930000	20.13710000	0.00000000
123	3216.75610000	13.57740000	0.00000000

Left Rotamer Equatorial

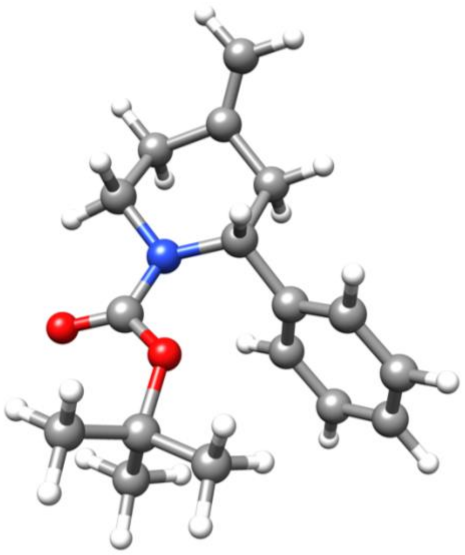

Route : # opt freq b3lyp scrf=(solvent=thf) geom=connectivity def2tzvp empiricaldispersion=gd3bj int=ultrafine pop=(regular,mk)

SMILES : C=C2CCN(C(=O)OC(C)(C)C)C(c1ccccc1)C2

Formula : $C_{17}H_{23}NO_2$

Charge : 0

Multiplicity : 1

Dipole : 2.5409 Debye

Energy : -867.295403418 a.u.

Gibbs Energy : -866.973483 a.u.

Number of imaginary frequencies : 0

Cartesian Coordinates (XYZ format)

43

C	-3.48872700	-1.12642600	-0.70406700
C	-2.21300100	-1.82479900	-0.25297200
C	-1.20184600	0.37630100	0.45404900
C	-2.55702100	1.02659800	0.07748100
H	-2.37627500	-2.24226400	0.74907100
H	-1.96921600	-2.64631500	-0.91789200
H	-3.38330600	-0.82964300	-1.75290600
H	-4.31940300	-1.82927200	-0.63355700
H	-1.19082200	0.22463900	1.53929200
H	-2.70022300	1.88941800	0.72736800
H	-2.46712300	1.40022900	-0.94727300
C	-3.71674100	0.08624100	0.14153200
C	-4.79553100	0.28776500	0.89105500
H	-5.61404100	-0.42198500	0.90465200
H	-4.89726700	1.17200900	1.50880200
N	-1.04378500	-0.92912700	-0.21792600

C	0.16123200	-1.60270300	-0.21045300
O	0.31523600	-2.69921000	-0.72139800
O	1.11600100	-0.92159500	0.43025100
C	2.54168700	-1.27502000	0.31660400
C	2.80194100	-2.60426600	1.01455800
H	2.31071200	-3.42283800	0.49547700
H	3.87629100	-2.79365400	1.03898300
H	2.43918400	-2.56703100	2.04294200
C	2.96054400	-1.29115900	-1.14881900
H	4.04180000	-1.42259900	-1.20863700
H	2.48108900	-2.10242300	-1.69043900
H	2.70423200	-0.34306800	-1.62259400
C	3.22675700	-0.13153200	1.05333400
H	2.88111000	-0.08059400	2.08627100
H	4.30548900	-0.29056800	1.05559100
H	3.01075100	0.81990600	0.56950200
C	-0.13038700	1.38542100	0.09725000
C	0.29184400	1.55838500	-1.21931600
C	0.37490500	2.22786000	1.08116100
C	1.22354000	2.53647300	-1.53885200
H	-0.09501500	0.90787800	-1.99327800
C	1.29904600	3.21740800	0.76369700
H	0.05529600	2.09910100	2.10832800
C	1.73192600	3.37151900	-0.54762900
H	1.55448700	2.64838300	-2.56360100
H	1.68863800	3.85930000	1.54352100
H	2.45920200	4.13333600	-0.79663800

Frequencies

Mode	IR frequency	IR intensity	Raman intensity
1	22.03290000	0.49000000	0.00000000
2	40.45610000	0.35270000	0.00000000
3	60.34950000	4.19230000	0.00000000
4	67.42860000	0.04790000	0.00000000
5	75.24240000	0.02880000	0.00000000
6	91.43500000	2.34870000	0.00000000
7	105.79890000	1.29440000	0.00000000
8	140.09510000	0.13080000	0.00000000
9	171.77960000	0.22190000	0.00000000
10	182.85150000	4.51620000	0.00000000
11	209.75920000	0.66660000	0.00000000
12	220.32100000	4.68400000	0.00000000
13	242.00230000	0.48270000	0.00000000
14	250.10050000	0.49620000	0.00000000
15	274.24460000	2.82900000	0.00000000
16	278.34080000	2.58680000	0.00000000
17	285.78990000	4.94000000	0.00000000
18	324.31710000	4.34990000	0.00000000

19	355.29400000	1.26240000	0.00000000
20	363.42850000	15.26280000	0.00000000
21	383.48180000	8.23190000	0.00000000
22	417.77920000	0.13190000	0.00000000
23	419.33730000	0.94310000	0.00000000
24	430.35130000	5.60970000	0.00000000
25	464.78630000	2.25110000	0.00000000
26	467.36990000	3.01530000	0.00000000
27	468.12900000	2.65560000	0.00000000
28	532.31110000	1.15100000	0.00000000
29	556.61320000	12.24140000	0.00000000
30	610.52550000	30.36150000	0.00000000
31	635.96070000	0.05190000	0.00000000
32	669.28950000	14.98480000	0.00000000
33	696.67420000	8.89860000	0.00000000
34	718.08190000	68.61840000	0.00000000
35	737.25580000	2.17330000	0.00000000
36	761.86530000	12.81390000	0.00000000
37	776.55150000	26.42290000	0.00000000
38	790.94940000	53.49620000	0.00000000
39	809.08940000	14.24560000	0.00000000
40	838.47520000	3.18560000	0.00000000
41	854.60230000	9.64790000	0.00000000
42	861.27550000	38.69870000	0.00000000
43	862.27150000	4.26100000	0.00000000
44	918.72080000	8.18220000	0.00000000
45	927.67090000	21.97020000	0.00000000
46	930.26610000	41.29340000	0.00000000
47	931.57400000	0.30360000	0.00000000
48	931.92810000	0.61000000	0.00000000
49	974.20350000	0.21100000	0.00000000
50	978.59230000	9.65010000	0.00000000
51	985.23920000	0.53180000	0.00000000
52	1000.82130000	1.53800000	0.00000000
53	1014.62050000	20.69160000	0.00000000
54	1022.29350000	34.67290000	0.00000000
55	1025.47420000	2.34010000	0.00000000
56	1031.78840000	14.50270000	0.00000000
57	1053.34540000	10.27100000	0.00000000
58	1054.47130000	1.65170000	0.00000000
59	1063.09100000	1.76090000	0.00000000
60	1077.26260000	13.52930000	0.00000000
61	1104.62530000	13.24600000	0.00000000

62	1140.60920000	58.77210000	0.00000000
63	1157.04250000	74.94480000	0.00000000
64	1178.42140000	3.56200000	0.00000000
65	1183.80470000	521.78070000	0.00000000
66	1200.04040000	3.28940000	0.00000000
67	1212.99860000	4.80630000	0.00000000
68	1228.47050000	23.15160000	0.00000000
69	1253.98090000	12.77100000	0.00000000
70	1270.69200000	29.04240000	0.00000000
71	1276.87350000	192.76640000	0.00000000
72	1283.58290000	22.50890000	0.00000000
73	1291.21070000	137.40570000	0.00000000
74	1309.59700000	1.26230000	0.00000000
75	1326.49350000	82.02740000	0.00000000
76	1345.78170000	118.00340000	0.00000000
77	1357.19860000	13.63800000	0.00000000
78	1359.34030000	45.87040000	0.00000000
79	1368.71930000	51.34900000	0.00000000
80	1396.88200000	25.97130000	0.00000000
81	1398.39860000	58.45590000	0.00000000
82	1400.89160000	34.90900000	0.00000000
83	1423.33380000	56.91950000	0.00000000
84	1425.50890000	97.32290000	0.00000000
85	1455.12610000	5.25360000	0.00000000
86	1467.88220000	0.35040000	0.00000000
87	1468.89350000	5.69880000	0.00000000
88	1484.21540000	9.74030000	0.00000000
89	1484.72180000	3.13500000	0.00000000
90	1485.98300000	2.50370000	0.00000000
91	1488.75970000	24.51830000	0.00000000
92	1491.77480000	9.38220000	0.00000000
93	1498.32810000	2.64370000	0.00000000
94	1508.22840000	10.80060000	0.00000000
95	1517.58540000	22.63130000	0.00000000
96	1532.75530000	19.40900000	0.00000000
97	1629.03240000	1.17970000	0.00000000
98	1647.55880000	17.81460000	0.00000000
99	1701.25430000	687.48360000	0.00000000
100	1720.61590000	36.47150000	0.00000000
101	2980.70690000	63.78030000	0.00000000
102	2997.90540000	40.47240000	0.00000000
103	3019.11770000	21.95580000	0.00000000
104	3026.58810000	55.49470000	0.00000000

105	3042.55910000	17.28790000	0.00000000
106	3045.05090000	32.60710000	0.00000000
107	3052.07840000	26.89620000	0.00000000
108	3089.03990000	44.64920000	0.00000000
109	3097.63280000	23.93710000	0.00000000
110	3101.62980000	15.20980000	0.00000000
111	3108.32090000	14.45010000	0.00000000
112	3113.61750000	64.72460000	0.00000000
113	3127.15570000	25.90220000	0.00000000
114	3131.09830000	16.62430000	0.00000000
115	3143.06140000	2.17170000	0.00000000
116	3145.32450000	10.00950000	0.00000000
117	3146.45510000	24.56730000	0.00000000
118	3163.19280000	8.92470000	0.00000000
119	3169.18020000	0.04220000	0.00000000
120	3178.12170000	15.35040000	0.00000000
121	3185.15890000	37.21380000	0.00000000
122	3195.23570000	21.47190000	0.00000000
123	3210.88940000	19.60330000	0.00000000

Right Rotamer Equatorial

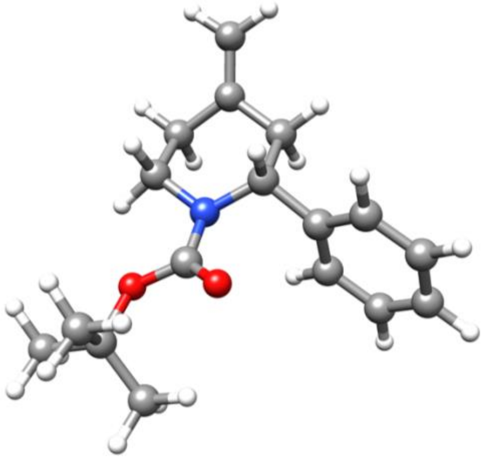

Route : # opt freq b3lyp scrf=(solvent=thf) geom=connectivity def2tzvp empiricaldispersion=gd3bj int=ultrafine pop=(regular,mk)

SMILES : C=C2CCN(C(=O)OC(C)(C)C)C(c1ccccc1)C2

Formula : $C_{17}H_{23}NO_2$

Charge : 0

Multiplicity : 1

Dipole : 2.9888 Debye

Energy : -867.292815979 a.u.

Gibbs Energy : -866.972580 a.u.

Number of imaginary frequencies : 0

Cartesian Coordinates (XYZ format)

43

C	0.69356000	-3.12724700	0.82557000
C	-0.38893300	-2.28343000	0.16357100
C	1.31705100	-0.53271400	-0.46519200
C	2.39173300	-1.46824800	0.13656000
H	-0.57884900	-2.68122800	-0.84156100
H	-1.31163300	-2.35356900	0.72745600
H	0.78393000	-2.83075200	1.87597600
H	0.38930200	-4.17395800	0.79572400
H	1.31650200	-0.67042800	-1.55249600
H	3.32020100	-1.29871200	-0.40805100
H	2.55828200	-1.15619400	1.17229700
C	1.99935900	-2.91035400	0.12938100
C	2.70512600	-3.87044200	-0.45903100
H	2.38045300	-4.90392500	-0.43772500
H	3.63324300	-3.65429600	-0.97448600
N	-0.02290200	-0.85747700	0.06168500
C	-1.03217400	0.02194900	-0.27927000
O	-0.86568400	1.03798600	-0.92472200

O	-2.21460200	-0.38196900	0.21799200
C	-3.45268400	0.37928200	-0.01347400
C	-3.34573400	1.75787300	0.62865700
H	-2.60592800	2.37241500	0.12320300
H	-4.31494400	2.25588500	0.57360800
H	-3.07069500	1.66332600	1.68023200
C	-3.75728500	0.45217800	-1.50565000
H	-4.74820500	0.88593700	-1.64757400
H	-3.02924100	1.06479000	-2.03011100
H	-3.75920200	-0.54903200	-1.93943900
C	-4.49743400	-0.46664700	0.70396400
H	-4.25573600	-0.55680500	1.76344300
H	-5.47852000	-0.00066700	0.61034100
H	-4.54526200	-1.46614600	0.27046900
C	1.74491100	0.88688300	-0.15881000
C	1.57269400	1.43081000	1.11254900
C	2.41717400	1.63060600	-1.12264300
C	2.04444800	2.70330800	1.40617300
H	1.05081400	0.85983000	1.86997600
C	2.89874300	2.90205400	-0.83059200
H	2.55331300	1.21731100	-2.11485400
C	2.71099700	3.44502000	0.43516300
H	1.89269300	3.11764700	2.39494700
H	3.41426900	3.47035600	-1.59440900
H	3.07850700	4.43713200	0.66352200

Frequencies

Mode	IR frequency	IR intensity	Raman intensity
1	24.69350000	0.10800000	0.00000000
2	26.31540000	0.55800000	0.00000000
3	40.91610000	2.70950000	0.00000000
4	47.35990000	0.19540000	0.00000000
5	67.71640000	0.71750000	0.00000000
6	87.87200000	0.67590000	0.00000000
7	111.06730000	0.76730000	0.00000000
8	119.97730000	1.90210000	0.00000000
9	167.22320000	1.38040000	0.00000000
10	186.44590000	4.95200000	0.00000000
11	205.49770000	0.22030000	0.00000000
12	223.87930000	2.17900000	0.00000000
13	237.21130000	3.30500000	0.00000000
14	245.56130000	1.21050000	0.00000000
15	270.96990000	3.02940000	0.00000000
16	276.67470000	9.89850000	0.00000000
17	284.50800000	1.54640000	0.00000000
18	318.90600000	0.24690000	0.00000000
19	341.94190000	31.04130000	0.00000000
20	352.76330000	2.03610000	0.00000000

21	396.36240000	0.75060000	0.00000000
22	416.53490000	0.34250000	0.00000000
23	420.56820000	0.13120000	0.00000000
24	430.68880000	5.98750000	0.00000000
25	460.72750000	1.46960000	0.00000000
26	467.31630000	5.87210000	0.00000000
27	470.21020000	0.35600000	0.00000000
28	530.60720000	17.14970000	0.00000000
29	562.32420000	2.84640000	0.00000000
30	612.39060000	28.56850000	0.00000000
31	636.92230000	0.12210000	0.00000000
32	650.33180000	4.92440000	0.00000000
33	697.91400000	14.86230000	0.00000000
34	714.97420000	58.91570000	0.00000000
35	736.89210000	0.57690000	0.00000000
36	766.89960000	25.43960000	0.00000000
37	774.85390000	24.21280000	0.00000000
38	797.90750000	32.73050000	0.00000000
39	808.71450000	20.01440000	0.00000000
40	837.83760000	0.14980000	0.00000000
41	849.41790000	13.28300000	0.00000000
42	858.28190000	0.15660000	0.00000000
43	871.92140000	36.78450000	0.00000000
44	918.78150000	13.56530000	0.00000000
45	925.35860000	12.41350000	0.00000000
46	928.59880000	40.56900000	0.00000000
47	930.43060000	0.63160000	0.00000000
48	932.23090000	7.73380000	0.00000000
49	971.86720000	0.09570000	0.00000000
50	975.09020000	0.64380000	0.00000000
51	977.79900000	4.68880000	0.00000000
52	990.10390000	1.42600000	0.00000000
53	1014.37660000	24.49070000	0.00000000
54	1024.47490000	0.07730000	0.00000000
55	1026.45450000	78.56160000	0.00000000
56	1032.44980000	3.43130000	0.00000000
57	1052.70280000	5.52420000	0.00000000
58	1052.75120000	6.82180000	0.00000000
59	1061.08100000	4.90780000	0.00000000
60	1077.74770000	22.65070000	0.00000000
61	1103.55630000	5.36740000	0.00000000
62	1135.28950000	166.84240000	0.00000000
63	1155.67910000	56.74680000	0.00000000

64	1178.24010000	8.60050000	0.00000000
65	1183.38440000	620.40370000	0.00000000
66	1198.97590000	4.27780000	0.00000000
67	1215.09890000	6.69930000	0.00000000
68	1233.48060000	17.65410000	0.00000000
69	1256.07470000	177.29880000	0.00000000
70	1261.09690000	108.54890000	0.00000000
71	1269.10750000	21.52440000	0.00000000
72	1282.91490000	33.21200000	0.00000000
73	1288.50690000	51.31480000	0.00000000
74	1310.36990000	5.79970000	0.00000000
75	1327.58190000	89.12910000	0.00000000
76	1345.45610000	148.09720000	0.00000000
77	1356.31530000	12.78570000	0.00000000
78	1363.26810000	78.76170000	0.00000000
79	1368.88900000	36.09440000	0.00000000
80	1396.96380000	25.65920000	0.00000000
81	1399.18350000	42.10230000	0.00000000
82	1402.21040000	47.98790000	0.00000000
83	1413.62820000	54.69410000	0.00000000
84	1423.25140000	34.17920000	0.00000000
85	1455.70760000	2.87750000	0.00000000
86	1466.96780000	0.17000000	0.00000000
87	1468.72570000	6.83170000	0.00000000
88	1483.84510000	0.94980000	0.00000000
89	1484.24420000	1.69830000	0.00000000
90	1485.12210000	10.27140000	0.00000000
91	1487.79990000	17.28970000	0.00000000
92	1490.83210000	9.69190000	0.00000000
93	1497.71830000	2.16780000	0.00000000
94	1508.45160000	4.25300000	0.00000000
95	1516.52380000	22.63140000	0.00000000
96	1532.32320000	17.66470000	0.00000000
97	1628.98830000	1.63260000	0.00000000
98	1647.39650000	14.55970000	0.00000000
99	1720.15480000	204.23450000	0.00000000
100	1723.67770000	393.41920000	0.00000000
101	2982.18940000	61.65630000	0.00000000
102	2997.20510000	42.27880000	0.00000000
103	3018.44020000	22.65970000	0.00000000
104	3026.73750000	53.87360000	0.00000000
105	3040.94880000	16.47740000	0.00000000
106	3043.38130000	38.73260000	0.00000000

107	3050.16950000	22.30400000	0.00000000
108	3088.88740000	44.80140000	0.00000000
109	3097.26630000	24.35050000	0.00000000
110	3102.22170000	7.57100000	0.00000000
111	3103.59850000	25.57260000	0.00000000
112	3110.93270000	46.31660000	0.00000000
113	3116.99510000	62.99780000	0.00000000
114	3131.07570000	16.43210000	0.00000000
115	3145.82610000	1.70870000	0.00000000
116	3148.63810000	25.17380000	0.00000000
117	3154.61640000	12.32440000	0.00000000
118	3162.51830000	9.47720000	0.00000000
119	3167.91860000	0.06890000	0.00000000
120	3176.95450000	16.57950000	0.00000000
121	3183.96230000	42.61260000	0.00000000
122	3194.17770000	22.40660000	0.00000000
123	3210.83250000	19.41290000	0.00000000

Left Rotamer Equatorial Re-Optimized

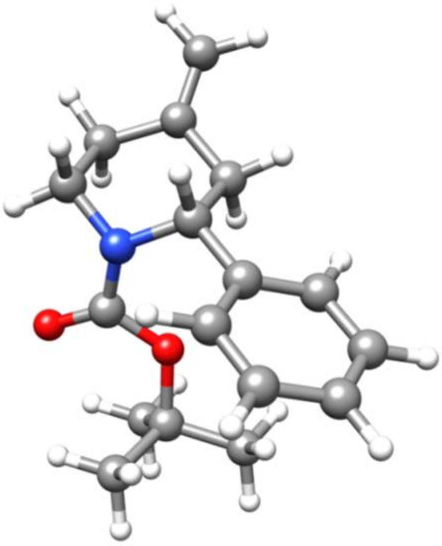

Route : # opt freq b3lyp scrf=(solvent=thf) geom=connectivity def2tzvp empiricaldispersion=gd3bj int=ultrafine pop=(regular,mk)

SMILES : C=C2CCN(C(=O)OC(C)(C)C)C(c1ccccc1)C2

Formula : $C_{17}H_{23}NO_2$

Charge : 0

Multiplicity : 1

Dipole : 2.5348 Debye

Energy : -867.296096363 a.u.

Gibbs Energy : -866.973549 a.u.

Number of imaginary frequencies : 0

Cartesian Coordinates (XYZ format)

43

C	-3.42792700	0.76307600	-0.13179500
C	-2.50772000	0.71741200	-1.36170600
C	-1.10390400	-0.98441500	-0.34106000
C	-1.92485700	-0.98128300	0.95867600
H	-2.85530800	-0.03633100	-2.07026200
H	-2.47878800	1.67844000	-1.86418500
H	-3.10952100	1.60888400	0.48775800
H	-4.45917100	0.94767400	-0.43563800
H	-1.65861800	-1.60513300	-1.05280800
H	-1.95643000	-1.97925200	1.39492200
H	-1.43732400	-0.31335700	1.67577100
C	-3.32848800	-0.51090400	0.66533200
C	-4.39886000	-1.20642800	1.03967000
H	-5.40170000	-0.87049000	0.80464300
H	-4.30648800	-2.12956400	1.59933900

N	-1.15215500	0.36095500	-0.94429700
C	-0.33327600	1.40274400	-0.58195100
O	-0.47619100	2.53952600	-1.00160100
O	0.62323500	1.01362800	0.26886400
C	1.80121000	1.84771600	0.55608900
C	2.53104300	2.17678700	-0.74086400
H	1.93595900	2.82749900	-1.37669900
H	3.46941300	2.68114600	-0.50613700
H	2.76204100	1.25942200	-1.28392900
C	1.38280800	3.09482500	1.32579700
H	2.27458100	3.63120800	1.65409500
H	0.78089700	3.75558100	0.70800600
H	0.80885400	2.81550500	2.21077900
C	2.65061700	0.93327900	1.42909400
H	2.91260400	0.02369100	0.89057400
H	3.56629400	1.44926800	1.71941300
H	2.10752300	0.65438500	2.33250900
C	0.28140200	-1.59314400	-0.27587900
C	0.77840100	-2.19852000	0.87263100
C	1.06662700	-1.60242300	-1.42981600
C	2.03842700	-2.79025600	0.87502100
H	0.19725500	-2.19956800	1.78365700
C	2.32642800	-2.18191000	-1.42896000
H	0.69012800	-1.13193200	-2.32944400
C	2.81951100	-2.78075900	-0.27230100
H	2.41096500	-3.24996600	1.78157700
H	2.92472800	-2.16955200	-2.33115300
H	3.80218600	-3.23426000	-0.26868300

Frequencies

Mode	IR frequency	IR intensity	Raman intensity
1	28.92760000	1.68780000	0.00000000
2	33.66180000	0.26290000	0.00000000
3	52.39400000	0.01620000	0.00000000
4	73.37800000	0.12730000	0.00000000
5	81.27800000	3.82970000	0.00000000
6	100.63980000	1.01760000	0.00000000
7	117.69270000	0.82780000	0.00000000
8	130.76300000	0.30520000	0.00000000
9	135.65300000	0.55860000	0.00000000
10	187.00340000	1.84500000	0.00000000
11	205.64790000	0.25920000	0.00000000
12	216.46970000	6.31200000	0.00000000
13	244.46510000	0.27350000	0.00000000
14	272.47360000	1.67510000	0.00000000
15	276.08270000	0.28010000	0.00000000
16	311.79050000	2.17420000	0.00000000
17	324.07560000	5.32540000	0.00000000
18	344.66560000	5.68850000	0.00000000

19	359.04860000	2.33070000	0.00000000
20	382.48320000	4.23330000	0.00000000
21	399.23730000	18.80210000	0.00000000
22	414.63120000	0.30070000	0.00000000
23	423.07500000	1.93150000	0.00000000
24	436.69290000	2.32410000	0.00000000
25	462.29210000	2.97740000	0.00000000
26	479.78690000	7.34260000	0.00000000
27	490.28310000	0.34870000	0.00000000
28	515.42550000	1.71190000	0.00000000
29	546.97880000	2.60960000	0.00000000
30	625.07830000	12.26560000	0.00000000
31	639.21050000	2.19330000	0.00000000
32	647.22560000	41.77350000	0.00000000
33	681.29580000	9.74250000	0.00000000
34	714.00020000	60.06290000	0.00000000
35	733.12650000	1.53520000	0.00000000
36	766.24960000	9.73520000	0.00000000
37	767.72950000	35.79680000	0.00000000
38	785.12650000	43.02070000	0.00000000
39	797.24860000	8.07860000	0.00000000
40	830.39170000	5.46250000	0.00000000
41	859.06850000	0.88910000	0.00000000
42	862.97590000	29.93470000	0.00000000
43	869.18240000	23.18460000	0.00000000
44	917.93730000	20.19720000	0.00000000
45	927.42040000	48.56600000	0.00000000
46	931.24170000	0.74710000	0.00000000
47	931.52860000	0.32190000	0.00000000
48	943.84080000	17.10030000	0.00000000
49	959.83050000	8.52640000	0.00000000
50	972.90110000	0.25900000	0.00000000
51	982.71660000	0.51040000	0.00000000
52	998.51860000	50.00810000	0.00000000
53	999.80300000	1.31620000	0.00000000
54	1014.44060000	36.50940000	0.00000000
55	1024.82300000	16.59360000	0.00000000
56	1026.51500000	4.95270000	0.00000000
57	1053.45150000	1.12090000	0.00000000
58	1056.07420000	8.34450000	0.00000000
59	1062.43420000	1.05710000	0.00000000
60	1070.55200000	22.97860000	0.00000000
61	1115.92820000	6.30940000	0.00000000

62	1146.64670000	67.03450000	0.00000000
63	1165.76320000	34.20700000	0.00000000
64	1179.84000000	8.71060000	0.00000000
65	1186.00370000	150.44880000	0.00000000
66	1187.95800000	313.59630000	0.00000000
67	1207.18430000	1.89560000	0.00000000
68	1235.90590000	2.35240000	0.00000000
69	1252.92010000	8.70100000	0.00000000
70	1269.98280000	29.15830000	0.00000000
71	1273.40950000	216.20630000	0.00000000
72	1286.60070000	77.69050000	0.00000000
73	1290.07530000	14.20440000	0.00000000
74	1305.84850000	6.55730000	0.00000000
75	1323.87380000	131.63150000	0.00000000
76	1339.68420000	18.23590000	0.00000000
77	1349.74850000	11.16020000	0.00000000
78	1361.47370000	4.32780000	0.00000000
79	1378.98850000	10.07320000	0.00000000
80	1385.96590000	24.98240000	0.00000000
81	1396.30010000	26.20770000	0.00000000
82	1400.88380000	30.49720000	0.00000000
83	1424.32940000	18.14900000	0.00000000
84	1450.02680000	169.98470000	0.00000000
85	1456.02750000	87.39860000	0.00000000
86	1466.88460000	0.26860000	0.00000000
87	1472.37730000	11.17010000	0.00000000
88	1483.97780000	2.75310000	0.00000000
89	1485.26470000	3.54080000	0.00000000
90	1485.74810000	31.92310000	0.00000000
91	1487.28650000	14.59100000	0.00000000
92	1488.53000000	7.19280000	0.00000000
93	1496.43380000	60.74000000	0.00000000
94	1497.36110000	3.74290000	0.00000000
95	1517.01030000	36.46110000	0.00000000
96	1536.30970000	17.17490000	0.00000000
97	1628.06460000	2.59340000	0.00000000
98	1649.22470000	16.25480000	0.00000000
99	1698.38340000	733.11260000	0.00000000
100	1709.47210000	35.27680000	0.00000000
101	3003.85100000	14.34550000	0.00000000
102	3013.06260000	19.91580000	0.00000000
103	3029.01150000	29.50320000	0.00000000
104	3042.18020000	18.95250000	0.00000000

105	3044.06500000	29.14180000	0.00000000
106	3051.08670000	28.35480000	0.00000000
107	3056.85940000	46.99690000	0.00000000
108	3084.32270000	60.91580000	0.00000000
109	3095.48150000	32.96890000	0.00000000
110	3100.89770000	15.64060000	0.00000000
111	3106.35960000	17.53710000	0.00000000
112	3113.58970000	65.95530000	0.00000000
113	3126.77850000	25.71940000	0.00000000
114	3131.10850000	16.52930000	0.00000000
115	3140.86230000	7.58300000	0.00000000
116	3146.48660000	18.27510000	0.00000000
117	3151.20010000	10.25770000	0.00000000
118	3167.03100000	2.30600000	0.00000000
119	3173.98620000	2.78900000	0.00000000
120	3182.79940000	29.33180000	0.00000000
121	3193.05860000	34.53630000	0.00000000
122	3203.57790000	11.08460000	0.00000000
123	3210.43890000	20.99850000	0.00000000

Right Rotamer Equatorial Re-Optimized

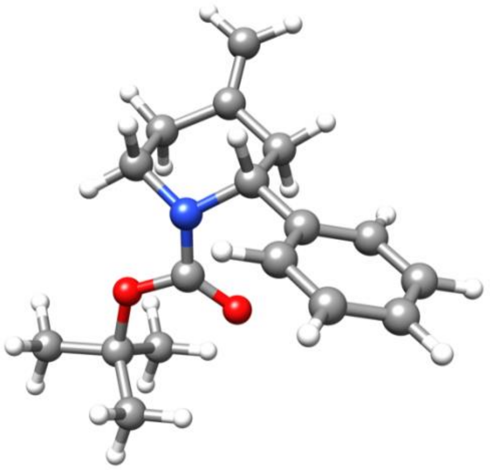

Route : # opt freq b3lyp scrf=(solvent=thf) geom=connectivity def2tzvp empiricaldispersion=gd3bj int=ultrafine pop=(regular,mk)

SMILES : C=C2CCN(C(=O)OC(C)(C)C)C(c1ccccc1)C2

Formula : $C_{17}H_{23}NO_2$

Charge : 0

Multiplicity : 1

Dipole : 2.9756 Debye

Energy : -867.293496677 a.u.

Gibbs Energy : -866.972175 a.u.

Number of imaginary frequencies : 0

Cartesian Coordinates (XYZ format)

43

C	0.93684500	2.86530400	-0.29797400
C	0.81602900	1.76876900	-1.36730000
C	-1.25170500	0.93962800	-0.39911000
C	-1.20229200	1.96156500	0.74803400
H	0.22391800	2.12797200	-2.21077200
H	1.79051600	1.47304300	-1.73773000
H	1.62114100	2.49973800	0.47610800
H	1.37605600	3.76716000	-0.72665600
H	-1.65979500	1.46481300	-1.26840200
H	-2.20890200	2.26631400	1.03354900
H	-0.73459300	1.48469200	1.61491000
C	-0.40537600	3.16746400	0.31563300
C	-0.88112500	4.40532800	0.42009300
H	-0.30708200	5.26159900	0.08684000
H	-1.85625300	4.60211700	0.84938900
N	0.12683000	0.60517800	-0.80285600
C	0.82929600	-0.31495500	-0.06204300

O	0.34274300	-1.01535100	0.80467200
O	2.11828700	-0.35760400	-0.45134600
C	3.09505400	-1.23845700	0.20686800
C	3.21863100	-0.87544000	1.68264100
H	2.31025000	-1.11764200	2.22735400
H	4.04931900	-1.43101600	2.12021600
H	3.42663900	0.19014600	1.79260000
C	2.71147400	-2.69870700	-0.00420900
H	3.51471900	-3.33808400	0.36522800
H	1.79468100	-2.94525000	0.52407900
H	2.57531600	-2.90267200	-1.06735400
C	4.38581300	-0.91069800	-0.53354100
H	4.63938200	0.14321100	-0.41407300
H	5.20362900	-1.51163700	-0.13538700
H	4.28252500	-1.12645000	-1.59741700
C	-2.13920400	-0.27048800	-0.19993300
C	-2.89552000	-0.46762000	0.95020000
C	-2.25298100	-1.19178800	-1.24193300
C	-3.73780200	-1.56958900	1.06469500
H	-2.82232800	0.22615600	1.77563000
C	-3.08665300	-2.29491300	-1.12953800
H	-1.66889200	-1.04611800	-2.14229300
C	-3.83514200	-2.48883100	0.02816300
H	-4.31436700	-1.70999300	1.97024200
H	-3.15621400	-3.00259800	-1.94598700
H	-4.48776500	-3.34753900	0.11883000

Frequencies

Mode	IR frequency	IR intensity	Raman intensity
1	23.52920000	0.86060000	0.00000000
2	31.55340000	0.04650000	0.00000000
3	37.25000000	0.21230000	0.00000000
4	60.17330000	0.80580000	0.00000000
5	73.59420000	3.05600000	0.00000000
6	108.10230000	0.33190000	0.00000000
7	111.05750000	0.64480000	0.00000000
8	123.61660000	0.74090000	0.00000000
9	140.54420000	1.10310000	0.00000000
10	185.30660000	5.22700000	0.00000000
11	206.13720000	0.23730000	0.00000000
12	219.08660000	4.51060000	0.00000000
13	242.66420000	0.93550000	0.00000000
14	271.37990000	4.80190000	0.00000000
15	274.94820000	1.67040000	0.00000000
16	284.78600000	0.62620000	0.00000000
17	330.75580000	22.81410000	0.00000000
18	343.11230000	1.92000000	0.00000000
19	353.82390000	2.21580000	0.00000000

20	385.32510000	7.97110000	0.00000000
21	411.79360000	1.80540000	0.00000000
22	414.64180000	0.26080000	0.00000000
23	422.31010000	0.82930000	0.00000000
24	439.11680000	7.98860000	0.00000000
25	461.83480000	4.17680000	0.00000000
26	480.80350000	5.65960000	0.00000000
27	487.05510000	5.08070000	0.00000000
28	516.51440000	1.76920000	0.00000000
29	577.06850000	4.96460000	0.00000000
30	607.22140000	16.47530000	0.00000000
31	635.15070000	4.36030000	0.00000000
32	645.97080000	18.95470000	0.00000000
33	677.77590000	15.28260000	0.00000000
34	711.02380000	53.15290000	0.00000000
35	733.61280000	2.53530000	0.00000000
36	756.17190000	59.25040000	0.00000000
37	777.47780000	15.72890000	0.00000000
38	787.02190000	14.56080000	0.00000000
39	798.95370000	20.25550000	0.00000000
40	830.90030000	1.85930000	0.00000000
41	854.75980000	15.49630000	0.00000000
42	855.54980000	0.17470000	0.00000000
43	879.35530000	33.51600000	0.00000000
44	916.74590000	10.40400000	0.00000000
45	926.46280000	56.96680000	0.00000000
46	929.85120000	0.25050000	0.00000000
47	930.84380000	0.07860000	0.00000000
48	942.26360000	2.43720000	0.00000000
49	959.51390000	3.16650000	0.00000000
50	972.51400000	0.02710000	0.00000000
51	972.52580000	0.45110000	0.00000000
52	988.25100000	1.75980000	0.00000000
53	997.12620000	65.90640000	0.00000000
54	1012.75200000	77.29580000	0.00000000
55	1024.50400000	12.40710000	0.00000000
56	1026.45330000	3.18130000	0.00000000
57	1053.59530000	1.84050000	0.00000000
58	1055.34780000	11.37710000	0.00000000
59	1060.35430000	3.40630000	0.00000000
60	1069.60270000	13.73580000	0.00000000
61	1114.64110000	7.91520000	0.00000000
62	1137.86690000	121.45220000	0.00000000

63	1171.15400000	260.14910000	0.00000000
64	1179.00560000	334.78710000	0.00000000
65	1179.81250000	1.80020000	0.00000000
66	1189.30290000	141.34050000	0.00000000
67	1205.96970000	1.20690000	0.00000000
68	1234.58850000	24.64630000	0.00000000
69	1249.55960000	170.38020000	0.00000000
70	1254.34400000	8.47540000	0.00000000
71	1269.15080000	22.67220000	0.00000000
72	1282.57170000	20.05520000	0.00000000
73	1287.81270000	2.39920000	0.00000000
74	1304.58890000	3.68430000	0.00000000
75	1332.88260000	147.68440000	0.00000000
76	1340.57410000	86.60810000	0.00000000
77	1349.75960000	27.69000000	0.00000000
78	1360.77760000	13.84420000	0.00000000
79	1378.58430000	11.40640000	0.00000000
80	1388.07240000	44.03590000	0.00000000
81	1397.16380000	25.88730000	0.00000000
82	1399.74230000	23.27340000	0.00000000
83	1422.72260000	14.20020000	0.00000000
84	1437.57990000	177.90540000	0.00000000
85	1453.67010000	2.54020000	0.00000000
86	1467.14920000	0.30860000	0.00000000
87	1471.97050000	9.88210000	0.00000000
88	1483.76800000	1.77690000	0.00000000
89	1484.11350000	0.08590000	0.00000000
90	1486.49260000	20.09070000	0.00000000
91	1487.22960000	17.61690000	0.00000000
92	1487.52550000	6.93800000	0.00000000
93	1497.68180000	2.15860000	0.00000000
94	1503.00940000	40.47690000	0.00000000
95	1516.68140000	33.86080000	0.00000000
96	1536.02380000	15.97230000	0.00000000
97	1628.15060000	2.19580000	0.00000000
98	1649.04180000	11.04700000	0.00000000
99	1708.98980000	93.98130000	0.00000000
100	1721.51060000	522.20050000	0.00000000
101	3010.03340000	31.23760000	0.00000000
102	3012.95550000	1.51420000	0.00000000
103	3031.85190000	24.62170000	0.00000000
104	3041.11590000	16.75480000	0.00000000
105	3042.93640000	38.75630000	0.00000000

106	3049.80900000	22.89250000	0.00000000
107	3056.72830000	50.14370000	0.00000000
108	3083.42390000	59.95990000	0.00000000
109	3094.06990000	32.25330000	0.00000000
110	3101.74440000	7.83900000	0.00000000
111	3103.76870000	28.79600000	0.00000000
112	3110.68470000	46.28800000	0.00000000
113	3117.17590000	59.89480000	0.00000000
114	3130.94940000	16.70940000	0.00000000
115	3145.25100000	3.32390000	0.00000000
116	3148.68050000	23.76070000	0.00000000
117	3161.71780000	13.81560000	0.00000000
118	3165.42600000	3.61030000	0.00000000
119	3172.37410000	1.91930000	0.00000000
120	3181.23130000	32.79490000	0.00000000
121	3192.15740000	37.40260000	0.00000000
122	3202.48040000	11.94490000	0.00000000
123	3210.25360000	21.35260000	0.00000000

Axial Clockwise Transition State

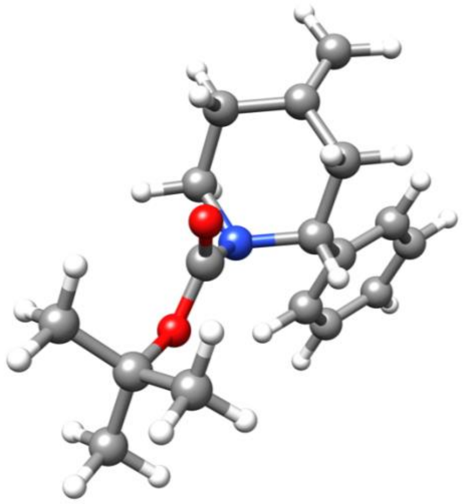

Route : # opt freq b3lyp scrf=(solvent=thf) geom=connectivity def2tzvp
empiricaldispersion=gd3bj int=ultrafine pop=(regular,mk)

SMILES : C=C2CCN(C(=O)OC(C)(C)C)C(c1ccccc1)C2

Formula : $C_{17}H_{23}NO_2$

Charge : 0

Multiplicity : 1

Dipole : 2.1747 Debye

Energy : -867.274004513 a.u.

Gibbs Energy : -866.951473 a.u.

Number of imaginary frequencies : -1

Cartesian Coordinates (XYZ format)

43

C	-1.19655600	-1.61359700	1.16879700
C	-0.78851000	-0.18746200	0.73389000
C	-0.25695000	-1.03369400	-1.51850700
C	-0.66570500	-2.46433100	-1.13608200
H	-0.27078400	0.27795600	1.57751900
H	-0.30401600	-2.07299400	1.59930700
H	-1.95220300	-1.58163800	1.95219900
H	-1.10964700	-0.52275100	-1.97127900
H	0.54419200	-1.04447000	-2.25831700
H	-1.08617100	-2.97131100	-2.00634700
H	0.22956600	-3.01039100	-0.82578500
C	-1.65060400	-2.44042700	-0.00095800
C	-2.81989100	-3.07298800	-0.03977300
H	-3.51591800	-3.02983700	0.78944800
H	-3.11611600	-3.65986700	-0.90109600
N	0.17774500	-0.21530700	-0.38021400

C	1.51857100	-0.45113700	0.02554300
O	1.93271100	-1.47441800	0.52292200
O	2.25272700	0.63031700	-0.22577800
C	3.69992800	0.68860300	0.08909900
C	3.90790800	0.53558100	1.59050400
H	3.65072600	-0.46452000	1.92909400
H	4.95627200	0.72432700	1.82534800
H	3.30202800	1.26323500	2.13238600
C	4.44686300	-0.36479800	-0.71917000
H	5.51988100	-0.21711400	-0.59030300
H	4.19331400	-1.37062400	-0.39495200
H	4.21501100	-0.26215300	-1.78026400
C	4.07837800	2.09045400	-0.36871800
H	3.50509800	2.84004700	0.17739400
H	5.13908700	2.26374600	-0.18643200
H	3.88469900	2.21129700	-1.43485300
C	-1.94946100	0.73367800	0.36772200
C	-1.68784500	1.90469500	-0.34732300
C	-3.26119200	0.48389700	0.76598500
C	-2.70485400	2.79758500	-0.65609900
H	-0.67446900	2.10037300	-0.66936800
C	-4.28386300	1.37651000	0.45744000
H	-3.50498600	-0.41264000	1.31518200
C	-4.01162400	2.53647400	-0.25530100
H	-2.47739400	3.69867600	-1.21174600
H	-5.29604900	1.15755300	0.77312200
H	-4.80741600	3.22879600	-0.49821400

Frequencies

Mode	IR frequency	IR intensity	Raman intensity
1	-60.63770000	2.12340000	0.00000000
2	20.26300000	0.01600000	0.00000000
3	37.57220000	0.18260000	0.00000000
4	46.92130000	0.52900000	0.00000000
5	80.77690000	0.23990000	0.00000000
6	101.02050000	0.23940000	0.00000000
7	108.70150000	0.34940000	0.00000000
8	149.70330000	0.91520000	0.00000000
9	170.19180000	4.65830000	0.00000000
10	193.00880000	2.06390000	0.00000000
11	203.49200000	0.14140000	0.00000000
12	214.71160000	1.54780000	0.00000000
13	239.97710000	0.65410000	0.00000000
14	261.53020000	5.69670000	0.00000000
15	271.77060000	0.12340000	0.00000000
16	278.85050000	0.35730000	0.00000000
17	314.55500000	7.39690000	0.00000000
18	326.33330000	13.62230000	0.00000000

19	357.27500000	2.57300000	0.00000000
20	392.74480000	5.69340000	0.00000000
21	405.16460000	1.07890000	0.00000000
22	415.16680000	0.58310000	0.00000000
23	417.86920000	0.48450000	0.00000000
24	440.78590000	4.42390000	0.00000000
25	464.51230000	0.70170000	0.00000000
26	469.51190000	2.80250000	0.00000000
27	477.65710000	9.55120000	0.00000000
28	505.91760000	9.33570000	0.00000000
29	575.29780000	4.91380000	0.00000000
30	600.56420000	9.52330000	0.00000000
31	637.85100000	0.00530000	0.00000000
32	710.88630000	2.12150000	0.00000000
33	713.15460000	61.30680000	0.00000000
34	718.52250000	40.77520000	0.00000000
35	734.94650000	21.01950000	0.00000000
36	748.92040000	9.57910000	0.00000000
37	773.42190000	7.34520000	0.00000000
38	784.35050000	25.28430000	0.00000000
39	804.00280000	20.57000000	0.00000000
40	827.04180000	4.38230000	0.00000000
41	849.66770000	33.64300000	0.00000000
42	860.62880000	15.85320000	0.00000000
43	861.46140000	10.00070000	0.00000000
44	913.76690000	41.35750000	0.00000000
45	915.89450000	27.51390000	0.00000000
46	928.53370000	5.82950000	0.00000000
47	932.86870000	0.26370000	0.00000000
48	933.52650000	0.05850000	0.00000000
49	967.11210000	13.92840000	0.00000000
50	973.73310000	0.05980000	0.00000000
51	983.95220000	0.20110000	0.00000000
52	986.77430000	37.00620000	0.00000000
53	998.11610000	1.90470000	0.00000000
54	1006.42800000	24.54190000	0.00000000
55	1018.00640000	110.97990000	0.00000000
56	1025.30400000	2.26210000	0.00000000
57	1035.69340000	4.87300000	0.00000000
58	1052.62120000	1.42290000	0.00000000
59	1056.51370000	8.29570000	0.00000000
60	1063.54580000	6.43210000	0.00000000
61	1107.45320000	4.43170000	0.00000000

62	1115.47710000	8.03460000	0.00000000
63	1141.70900000	54.94870000	0.00000000
64	1171.16760000	794.05400000	0.00000000
65	1178.98830000	1.62680000	0.00000000
66	1189.96740000	32.07480000	0.00000000
67	1205.22120000	5.57420000	0.00000000
68	1223.01140000	17.64620000	0.00000000
69	1241.78100000	18.22370000	0.00000000
70	1257.75030000	358.18980000	0.00000000
71	1265.67380000	38.00510000	0.00000000
72	1272.74730000	14.08290000	0.00000000
73	1283.27890000	23.90660000	0.00000000
74	1300.01630000	3.36060000	0.00000000
75	1315.75380000	173.52700000	0.00000000
76	1331.22240000	12.25510000	0.00000000
77	1356.06010000	6.34880000	0.00000000
78	1359.88720000	0.61050000	0.00000000
79	1373.65650000	14.40330000	0.00000000
80	1388.63100000	22.40930000	0.00000000
81	1398.77680000	25.24190000	0.00000000
82	1400.79580000	82.60940000	0.00000000
83	1402.10280000	16.24410000	0.00000000
84	1424.35760000	20.27530000	0.00000000
85	1454.88940000	6.84020000	0.00000000
86	1468.10210000	0.16970000	0.00000000
87	1471.34600000	9.54530000	0.00000000
88	1482.33100000	6.62360000	0.00000000
89	1483.94320000	0.13160000	0.00000000
90	1484.40340000	1.79140000	0.00000000
91	1488.74420000	14.54780000	0.00000000
92	1490.39090000	13.76500000	0.00000000
93	1493.08770000	42.64310000	0.00000000
94	1497.75260000	2.49610000	0.00000000
95	1516.35680000	22.25900000	0.00000000
96	1532.66880000	19.31640000	0.00000000
97	1624.83580000	0.98610000	0.00000000
98	1645.52740000	7.56640000	0.00000000
99	1711.07940000	46.90890000	0.00000000
100	1725.90830000	409.89140000	0.00000000
101	3026.63730000	21.59110000	0.00000000
102	3030.35170000	26.56590000	0.00000000
103	3039.69360000	34.44730000	0.00000000
104	3043.16920000	15.98070000	0.00000000

105	3045.34700000	35.36230000	0.00000000
106	3051.87130000	17.79040000	0.00000000
107	3052.31350000	25.43060000	0.00000000
108	3079.47610000	46.23520000	0.00000000
109	3089.99010000	41.61060000	0.00000000
110	3101.11850000	31.14560000	0.00000000
111	3104.92520000	7.95730000	0.00000000
112	3106.69580000	21.79160000	0.00000000
113	3113.64150000	42.38280000	0.00000000
114	3119.15650000	56.48330000	0.00000000
115	3129.27860000	15.05490000	0.00000000
116	3143.05000000	0.33170000	0.00000000
117	3145.98600000	27.38600000	0.00000000
118	3167.81770000	0.52460000	0.00000000
119	3176.53650000	18.33070000	0.00000000
120	3190.25080000	31.82520000	0.00000000
121	3199.90810000	15.64520000	0.00000000
122	3207.64620000	22.43350000	0.00000000
123	3218.12170000	13.62020000	0.00000000

Axial Anticlockwise Transition State

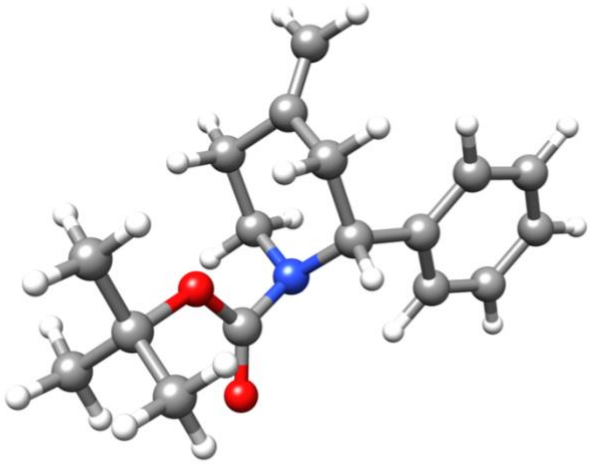

Route : # opt freq b3lyp scrf=(solvent=thf) geom=connectivity def2tzvp empiricaldispersion=gd3bj int=ultrafine pop=(regular,mk)

SMILES : C=C2CCN(C(=O)OC(C)(C)C)C(c1ccccc1)C2

Formula : $C_{17}H_{23}NO_2$

Charge : 0

Multiplicity : 1

Dipole : 3.5670 Debye

Energy : -867.273949768 a.u.

Gibbs Energy : -866.951141 a.u.

Number of imaginary frequencies : -1

Cartesian Coordinates (XYZ format)

43

C	-0.44218200	1.37968600	-0.98042400
C	-0.71349300	-0.09447100	-0.59845300
C	-0.12107200	0.41386800	1.74459700
C	0.13910800	1.89141700	1.41094500
H	-0.34356100	-0.72047700	-1.41540600
H	0.60031500	1.43413600	-1.29916100
H	-1.05301900	1.68157600	-1.82925300
H	-1.14765600	0.30430000	2.10253500
H	0.53698400	0.07551700	2.54542400
H	-0.12752100	2.51168300	2.26842000
H	1.20667100	2.02435700	1.21622600
C	-0.64265900	2.29635100	0.19275900
C	-1.45632600	3.34805900	0.16687500
H	-2.01880800	3.60772400	-0.72191100
H	-1.58238800	3.98614000	1.03350600
N	0.03728600	-0.49399900	0.60386700
C	1.35212600	-0.97376500	0.38000400

O	1.67258900	-2.12073000	0.57786400
O	2.17234700	-0.01595400	-0.06321100
C	3.59903300	-0.26325100	-0.37764200
C	4.33293100	-0.70634700	0.88111500
H	4.00254900	-1.68814600	1.20990800
H	5.40283900	-0.74969400	0.67320400
H	4.17206900	0.01258200	1.68575300
C	3.70620400	-1.27629000	-1.50966300
H	4.74826100	-1.35214600	-1.82312200
H	3.36517400	-2.25983600	-1.19719900
H	3.11638600	-0.94952800	-2.36728700
C	4.07706100	1.10965000	-0.82985000
H	3.95091900	1.84184200	-0.03188300
H	5.13343300	1.06376900	-1.09505100
H	3.51462100	1.44401700	-1.70202100
C	-2.19018900	-0.44150100	-0.41020500
C	-2.53774000	-1.58342500	0.31451100
C	-3.21424000	0.30963400	-0.98507100
C	-3.86539200	-1.96245100	0.46173200
H	-1.75103400	-2.16725500	0.77156000
C	-4.54606800	-0.06689300	-0.83945300
H	-2.98919200	1.20132300	-1.55015600
C	-4.87886200	-1.20362100	-0.11467500
H	-4.10895300	-2.85201000	1.02898400
H	-5.32310000	0.53623600	-1.29178500
H	-5.91491100	-1.49453100	0.00180000

Frequencies

Mode	IR frequency	IR intensity	Raman intensity
1	-59.18510000	1.44730000	0.00000000
2	25.91400000	0.01060000	0.00000000
3	39.50930000	0.59840000	0.00000000
4	51.24310000	0.69810000	0.00000000
5	75.25580000	0.28450000	0.00000000
6	88.58310000	0.34500000	0.00000000
7	111.80080000	0.86990000	0.00000000
8	145.41630000	1.30550000	0.00000000
9	164.76570000	2.71210000	0.00000000
10	191.92930000	3.71560000	0.00000000
11	201.07120000	0.18810000	0.00000000
12	211.15130000	1.56750000	0.00000000
13	241.50300000	0.18980000	0.00000000
14	262.62710000	1.15780000	0.00000000
15	272.70660000	0.07740000	0.00000000
16	282.42120000	0.44300000	0.00000000
17	324.23980000	7.80350000	0.00000000
18	354.69970000	1.40820000	0.00000000
19	361.82840000	1.47610000	0.00000000

20	370.55270000	2.09520000	0.00000000
21	409.71310000	3.37910000	0.00000000
22	416.42160000	1.15560000	0.00000000
23	419.10020000	1.13830000	0.00000000
24	446.04990000	0.23510000	0.00000000
25	463.14660000	10.80840000	0.00000000
26	467.32400000	2.92060000	0.00000000
27	476.18130000	9.58680000	0.00000000
28	506.85780000	8.09920000	0.00000000
29	566.56470000	5.60460000	0.00000000
30	623.62620000	12.49160000	0.00000000
31	638.79960000	1.69680000	0.00000000
32	662.34740000	23.34970000	0.00000000
33	714.30570000	44.72200000	0.00000000
34	717.27160000	29.61410000	0.00000000
35	735.18610000	21.22240000	0.00000000
36	760.61120000	6.82790000	0.00000000
37	775.89410000	4.75030000	0.00000000
38	783.83840000	23.71400000	0.00000000
39	802.05500000	20.43040000	0.00000000
40	825.62200000	2.93690000	0.00000000
41	853.03730000	26.52530000	0.00000000
42	858.87890000	47.15520000	0.00000000
43	862.63370000	0.60710000	0.00000000
44	914.46750000	63.85320000	0.00000000
45	916.60890000	8.52560000	0.00000000
46	928.39330000	3.19280000	0.00000000
47	932.57090000	0.07570000	0.00000000
48	933.54890000	0.06970000	0.00000000
49	966.76650000	18.28770000	0.00000000
50	974.44450000	0.04580000	0.00000000
51	983.90780000	3.36710000	0.00000000
52	984.58540000	2.45280000	0.00000000
53	1001.36330000	3.79660000	0.00000000
54	1006.41400000	22.94210000	0.00000000
55	1017.40190000	88.10660000	0.00000000
56	1024.97090000	1.32510000	0.00000000
57	1034.91490000	46.51150000	0.00000000
58	1053.42260000	1.22520000	0.00000000
59	1056.94210000	6.21440000	0.00000000
60	1063.58720000	7.98150000	0.00000000
61	1105.96730000	113.92590000	0.00000000
62	1111.56520000	37.83290000	0.00000000

63	1145.21760000	32.49280000	0.00000000
64	1157.00670000	728.46710000	0.00000000
65	1179.26360000	3.21350000	0.00000000
66	1189.19510000	32.04360000	0.00000000
67	1206.15150000	3.63580000	0.00000000
68	1220.62200000	13.08660000	0.00000000
69	1239.58220000	41.04680000	0.00000000
70	1242.80000000	89.00680000	0.00000000
71	1263.75390000	36.28720000	0.00000000
72	1272.81270000	18.97810000	0.00000000
73	1283.12050000	20.02690000	0.00000000
74	1299.21310000	0.15550000	0.00000000
75	1315.07490000	33.84820000	0.00000000
76	1329.91240000	8.90160000	0.00000000
77	1353.76960000	11.12780000	0.00000000
78	1360.12170000	2.01140000	0.00000000
79	1372.09550000	16.72420000	0.00000000
80	1388.46500000	30.60150000	0.00000000
81	1399.94710000	26.16950000	0.00000000
82	1402.46780000	35.93290000	0.00000000
83	1404.60300000	112.69910000	0.00000000
84	1425.96980000	17.55900000	0.00000000
85	1454.10090000	2.49510000	0.00000000
86	1467.92510000	0.10590000	0.00000000
87	1473.12600000	11.09810000	0.00000000
88	1481.96240000	8.50350000	0.00000000
89	1483.99980000	0.54870000	0.00000000
90	1484.64320000	1.71900000	0.00000000
91	1489.22540000	33.25490000	0.00000000
92	1490.73460000	12.80570000	0.00000000
93	1495.12570000	28.64690000	0.00000000
94	1497.43000000	3.72080000	0.00000000
95	1516.28150000	23.02430000	0.00000000
96	1532.87560000	20.55690000	0.00000000
97	1624.71720000	0.20600000	0.00000000
98	1645.07690000	7.04300000	0.00000000
99	1711.26920000	60.40630000	0.00000000
100	1748.87210000	591.63120000	0.00000000
101	3028.81140000	23.93120000	0.00000000
102	3032.59260000	28.21360000	0.00000000
103	3037.61720000	37.07980000	0.00000000
104	3043.23080000	15.25890000	0.00000000
105	3045.51350000	35.94040000	0.00000000

106	3051.71260000	15.69310000	0.00000000
107	3060.44560000	27.43290000	0.00000000
108	3080.80520000	45.63880000	0.00000000
109	3090.73810000	43.73360000	0.00000000
110	3105.33030000	5.47760000	0.00000000
111	3106.00040000	33.63850000	0.00000000
112	3107.10360000	21.90450000	0.00000000
113	3113.15020000	42.91280000	0.00000000
114	3118.89820000	59.65160000	0.00000000
115	3129.70260000	14.73710000	0.00000000
116	3142.34920000	2.54930000	0.00000000
117	3145.84560000	22.92640000	0.00000000
118	3167.84600000	0.39200000	0.00000000
119	3176.50510000	18.74020000	0.00000000
120	3190.32060000	32.34200000	0.00000000
121	3201.20290000	15.17260000	0.00000000
122	3208.23080000	20.90180000	0.00000000
123	3215.78980000	15.95760000	0.00000000

Equatorial Clockwise Transition State

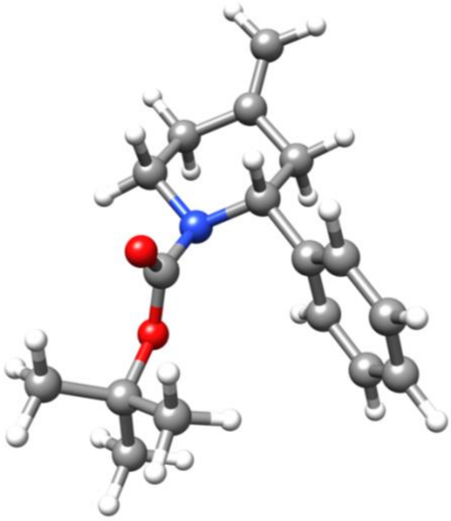

Route : # opt freq b3lyp scrf=(solvent=thf) geom=connectivity def2tzvp empiricaldispersion=gd3bj int=ultrafine pop=(regular,mk)

SMILES : C=C2CCN(C(=O)OC(C)(C)C)C(c1ccccc1)C2

Formula : $C_{17}H_{23}NO_2$

Charge : 0

Multiplicity : 1

Dipole : 2.0073 Debye

Energy : -867.282502567 a.u.

Gibbs Energy : -866.959664 a.u.

Number of imaginary frequencies : 1

Cartesian Coordinates (XYZ format)

43

C	3.11774700	-1.79716500	0.65711600
C	1.81122000	-2.00477900	-0.10579000
C	1.46272100	0.41244500	-0.32782900
C	2.77455500	0.67896700	0.42961600
H	2.02327500	-2.11170900	-1.17886400
H	1.31757500	-2.91725900	0.23135200
H	2.89725600	-1.81527100	1.72935900
H	3.80364200	-2.61662200	0.43936700
H	1.69462100	0.34940600	-1.39959200
H	3.21766400	1.60219700	0.05524900
H	2.52976600	0.82825100	1.48554900
C	3.72601000	-0.47380200	0.29120400
C	4.96903800	-0.33964300	-0.16106500
H	5.62803600	-1.19349800	-0.26432600
H	5.36906600	0.62906300	-0.43596800
N	0.90056000	-0.87476300	0.13957400

C	-0.37655100	-1.11204800	-0.46445200
O	-0.56214300	-1.17523100	-1.65732300
O	-1.30020200	-1.24376300	0.47676200
C	-2.73977300	-1.36276100	0.14570200
C	-3.18557400	-0.12434400	-0.62306200
H	-2.75072000	-0.09401200	-1.61838600
H	-4.27218100	-0.13811200	-0.71757100
H	-2.89741200	0.77917100	-0.08479800
C	-2.99299600	-2.65632400	-0.61700700
H	-4.06839900	-2.79443100	-0.73759100
H	-2.53252400	-2.63305100	-1.60115500
H	-2.60126100	-3.50836000	-0.05945300
C	-3.38856400	-1.40560600	1.52210100
H	-3.16884100	-0.49281300	2.07632200
H	-4.46975500	-1.49743500	1.41742800
H	-3.02125300	-2.25858800	2.09346100
C	0.46092300	1.52537800	-0.12586600
C	-0.10421300	1.75812600	1.12848400
C	0.07773200	2.33001900	-1.19514600
C	-1.03089300	2.77598800	1.30784300
H	0.16765500	1.12182300	1.95971100
C	-0.84796900	3.35327000	-1.01915700
H	0.49865100	2.14533600	-2.17578200
C	-1.40560400	3.57800000	0.23356000
H	-1.46676900	2.94075600	2.28497500
H	-1.13868600	3.96727200	-1.86183200
H	-2.13141800	4.36885500	0.37251300

Frequencies

Mode	IR frequency	IR intensity	Raman intensity
1	-39.47030000	2.26220000	0.00000000
2	38.91050000	0.02730000	0.00000000
3	47.98910000	0.24430000	0.00000000
4	56.92300000	0.92340000	0.00000000
5	71.03100000	0.87940000	0.00000000
6	82.09310000	0.94760000	0.00000000
7	110.58590000	0.36250000	0.00000000
8	131.69830000	2.49220000	0.00000000
9	169.68940000	3.60200000	0.00000000
10	194.69180000	3.12970000	0.00000000
11	204.87450000	0.23440000	0.00000000
12	210.08580000	0.19270000	0.00000000
13	236.71190000	1.48750000	0.00000000
14	240.97010000	0.38860000	0.00000000
15	270.84690000	0.16000000	0.00000000
16	280.07540000	5.80680000	0.00000000
17	303.68130000	2.04010000	0.00000000
18	318.60930000	3.25890000	0.00000000

19	324.79350000	8.11840000	0.00000000
20	359.58360000	3.50000000	0.00000000
21	398.99980000	0.57560000	0.00000000
22	415.44560000	0.39430000	0.00000000
23	416.73260000	1.77420000	0.00000000
24	425.53520000	5.72040000	0.00000000
25	456.56500000	0.78770000	0.00000000
26	475.62050000	5.67650000	0.00000000
27	494.40260000	0.36960000	0.00000000
28	524.03070000	15.57160000	0.00000000
29	548.62870000	9.34360000	0.00000000
30	607.54710000	18.01940000	0.00000000
31	636.79160000	0.13910000	0.00000000
32	662.77380000	11.89950000	0.00000000
33	715.36630000	70.53890000	0.00000000
34	733.90810000	0.58180000	0.00000000
35	745.03640000	23.94530000	0.00000000
36	758.41210000	11.38350000	0.00000000
37	778.46840000	22.17820000	0.00000000
38	799.93540000	7.82720000	0.00000000
39	808.13820000	12.85460000	0.00000000
40	836.67340000	2.41810000	0.00000000
41	850.09220000	42.20760000	0.00000000
42	861.10890000	1.84030000	0.00000000
43	868.56240000	23.62850000	0.00000000
44	918.70820000	18.85890000	0.00000000
45	928.78900000	42.05080000	0.00000000
46	932.80550000	0.91130000	0.00000000
47	933.42260000	1.15300000	0.00000000
48	935.94080000	10.16590000	0.00000000
49	970.33280000	1.04890000	0.00000000
50	973.84040000	0.11710000	0.00000000
51	983.40850000	0.21090000	0.00000000
52	1003.14110000	0.75290000	0.00000000
53	1006.86270000	11.89700000	0.00000000
54	1020.42100000	45.69750000	0.00000000
55	1026.00290000	1.47100000	0.00000000
56	1039.05040000	12.63060000	0.00000000
57	1053.65100000	5.70480000	0.00000000
58	1053.82370000	6.39170000	0.00000000
59	1063.15680000	3.38860000	0.00000000
60	1082.35620000	15.11080000	0.00000000
61	1105.11370000	14.05590000	0.00000000

62	1117.71290000	32.35900000	0.00000000
63	1122.06070000	73.26530000	0.00000000
64	1178.25390000	598.96420000	0.00000000
65	1179.80380000	11.84360000	0.00000000
66	1200.10580000	0.66780000	0.00000000
67	1204.86630000	10.87870000	0.00000000
68	1231.09980000	4.43350000	0.00000000
69	1248.95250000	25.34100000	0.00000000
70	1263.85290000	261.82070000	0.00000000
71	1271.71720000	44.86020000	0.00000000
72	1276.92080000	159.77430000	0.00000000
73	1285.44050000	39.08760000	0.00000000
74	1297.28940000	59.26740000	0.00000000
75	1307.30490000	2.16310000	0.00000000
76	1338.83170000	36.19790000	0.00000000
77	1347.01080000	6.86090000	0.00000000
78	1354.81750000	4.64680000	0.00000000
79	1363.50000000	32.25460000	0.00000000
80	1398.88240000	20.81290000	0.00000000
81	1399.27610000	29.97500000	0.00000000
82	1401.82940000	22.36960000	0.00000000
83	1406.34650000	15.90450000	0.00000000
84	1425.22130000	18.43580000	0.00000000
85	1452.95710000	1.65320000	0.00000000
86	1464.70880000	7.13160000	0.00000000
87	1468.26330000	0.44530000	0.00000000
88	1479.38230000	10.85980000	0.00000000
89	1483.72010000	0.17120000	0.00000000
90	1484.94500000	1.77500000	0.00000000
91	1489.50510000	12.11190000	0.00000000
92	1492.16620000	15.93620000	0.00000000
93	1497.52090000	2.55420000	0.00000000
94	1504.29240000	4.29950000	0.00000000
95	1516.21320000	23.91240000	0.00000000
96	1532.88730000	14.89370000	0.00000000
97	1628.44520000	1.16060000	0.00000000
98	1647.76620000	3.67280000	0.00000000
99	1714.72950000	43.52100000	0.00000000
100	1738.10260000	368.86690000	0.00000000
101	2967.13620000	64.02620000	0.00000000
102	2971.57480000	63.59170000	0.00000000
103	3020.73580000	29.54160000	0.00000000
104	3029.83300000	37.65400000	0.00000000

105	3042.68120000	16.84340000	0.00000000
106	3046.12940000	28.98450000	0.00000000
107	3052.80500000	22.99980000	0.00000000
108	3076.01760000	45.24360000	0.00000000
109	3086.89610000	44.42280000	0.00000000
110	3091.17950000	25.84970000	0.00000000
111	3104.66740000	13.89400000	0.00000000
112	3110.09420000	3.83530000	0.00000000
113	3113.70270000	43.40250000	0.00000000
114	3119.19330000	55.52020000	0.00000000
115	3130.39240000	16.96080000	0.00000000
116	3142.98780000	4.16990000	0.00000000
117	3146.95710000	21.61530000	0.00000000
118	3167.27750000	4.23140000	0.00000000
119	3173.15570000	1.49220000	0.00000000
120	3182.35090000	28.54420000	0.00000000
121	3192.27580000	32.83000000	0.00000000
122	3198.66290000	8.82900000	0.00000000
123	3209.85930000	20.46900000	0.00000000

Equatorial Anticlockwise Transition State

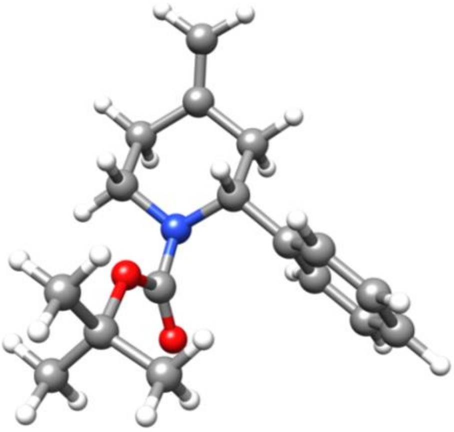

Route : # opt freq b3lyp scrf=(solvent=thf) geom=connectivity def2tzvp empiricaldispersion=gd3bj int=ultrafine pop=(regular,mk)

SMILES : C=C2CCN(C(=O)OC(C)(C)C)C(c1ccccc1)C2

Formula : $C_{17}H_{23}NO_2$

Charge : 0

Multiplicity : 1

Dipole : 3.0946 Debye

Energy : -867.280856122 a.u.

Gibbs Energy : -866.958475 a.u.

Number of imaginary frequencies : 1

Cartesian Coordinates (XYZ format)

43

C	-2.53577700	-2.39902300	-0.66626400
C	-1.02728600	-2.22702400	-0.49539700
C	-1.29038000	0.06187200	0.31377100
C	-2.81932200	-0.05307600	0.18563500
H	-0.72946600	-2.57112500	0.50338900
H	-0.49273000	-2.82951600	-1.23006700
H	-2.79651400	-2.15103400	-1.70016800
H	-2.80925600	-3.43981300	-0.48879000
H	-1.01030600	-0.29931700	1.31353400
H	-3.28561800	0.55164000	0.96395000
H	-3.10880100	0.36442500	-0.78304700
C	-3.26775700	-1.48325300	0.27228500
C	-4.18626000	-1.90373300	1.13686000
H	-4.48009100	-2.94543700	1.18712800
H	-4.67889200	-1.21826500	1.81618100
N	-0.66558400	-0.81671800	-0.70659900
C	0.73997200	-0.60582100	-0.82483000
O	1.23686000	0.02762400	-1.72145100
O	1.40588400	-1.19160100	0.17288600

C	2.87857600	-1.11052800	0.29932700
C	3.53753600	-1.76690500	-0.90696800
H	3.36251500	-1.19670100	-1.81548600
H	4.61305100	-1.82886900	-0.73559700
H	3.15598100	-2.78000800	-1.04215900
C	3.29368400	0.34430500	0.47807800
H	4.36172000	0.38598600	0.69602100
H	3.09681600	0.92680000	-0.41793100
H	2.75540100	0.79179400	1.31409600
C	3.14584500	-1.91102200	1.56598600
H	2.80489800	-2.94015700	1.44917500
H	4.21529300	-1.92149400	1.77707600
H	2.62858100	-1.46611500	2.41651800
C	-0.82021300	1.49051400	0.19328500
C	-1.04510400	2.23178400	-0.96700900
C	-0.15224200	2.09212100	1.25605600
C	-0.61333300	3.54715000	-1.05804300
H	-1.54120400	1.76847700	-1.80918800
C	0.28127600	3.41143600	1.16992300
H	0.03426200	1.52074700	2.15721100
C	0.05101400	4.14216600	0.01156100
H	-0.78962200	4.10947100	-1.96605700
H	0.80079800	3.86372400	2.00490400
H	0.38895500	5.16784400	-0.06150900

Frequencies

Mode	IR frequency	IR intensity	Raman intensity
1	-42.48740000	0.88420000	0.00000000
2	35.05330000	0.42770000	0.00000000
3	42.40640000	0.28900000	0.00000000
4	45.62610000	0.28600000	0.00000000
5	75.81450000	1.74350000	0.00000000
6	82.59030000	0.36540000	0.00000000
7	107.38750000	1.07350000	0.00000000
8	132.44440000	2.22120000	0.00000000
9	168.81710000	4.49220000	0.00000000
10	197.13810000	2.40750000	0.00000000
11	201.22090000	0.32400000	0.00000000
12	213.18560000	0.30310000	0.00000000
13	225.64620000	1.34280000	0.00000000
14	241.54980000	0.21250000	0.00000000
15	269.88320000	0.14970000	0.00000000
16	278.17730000	1.17640000	0.00000000
17	308.41920000	0.68990000	0.00000000
18	322.49200000	7.74770000	0.00000000
19	346.03140000	2.18850000	0.00000000
20	363.57470000	3.02640000	0.00000000

21	389.17030000	3.42530000	0.00000000
22	413.90550000	1.71630000	0.00000000
23	417.65560000	0.38440000	0.00000000
24	425.45120000	1.20040000	0.00000000
25	463.21940000	6.66380000	0.00000000
26	472.72180000	5.07540000	0.00000000
27	495.32920000	2.14250000	0.00000000
28	540.41530000	2.45830000	0.00000000
29	545.88580000	17.52430000	0.00000000
30	614.64620000	25.30710000	0.00000000
31	636.26050000	0.30200000	0.00000000
32	647.63560000	2.98880000	0.00000000
33	694.34840000	19.73390000	0.00000000
34	715.26600000	65.64530000	0.00000000
35	735.72760000	0.74200000	0.00000000
36	773.03040000	34.33490000	0.00000000
37	782.54240000	5.21440000	0.00000000
38	800.60960000	10.40250000	0.00000000
39	806.34300000	7.55160000	0.00000000
40	836.11720000	1.66770000	0.00000000
41	853.30340000	54.52430000	0.00000000
42	860.42500000	0.58380000	0.00000000
43	872.01190000	8.67530000	0.00000000
44	920.27020000	28.05880000	0.00000000
45	928.85020000	32.47810000	0.00000000
46	932.33110000	0.54280000	0.00000000
47	933.13090000	0.13980000	0.00000000
48	935.65370000	16.48160000	0.00000000
49	969.70690000	0.47840000	0.00000000
50	972.95520000	0.05900000	0.00000000
51	980.66360000	0.50140000	0.00000000
52	995.73730000	1.31790000	0.00000000
53	1007.94300000	18.45660000	0.00000000
54	1018.48290000	28.69020000	0.00000000
55	1025.71540000	1.74820000	0.00000000
56	1037.19490000	15.04570000	0.00000000
57	1052.89870000	1.79970000	0.00000000
58	1054.02020000	10.64950000	0.00000000
59	1062.15930000	1.88270000	0.00000000
60	1081.87920000	14.18900000	0.00000000
61	1103.90540000	49.07390000	0.00000000
62	1111.79650000	11.59640000	0.00000000
63	1121.66430000	120.28380000	0.00000000

64	1162.60540000	747.42000000	0.00000000
65	1179.90730000	0.10890000	0.00000000
66	1201.02030000	2.27120000	0.00000000
67	1204.69730000	10.38180000	0.00000000
68	1233.45330000	5.57100000	0.00000000
69	1240.85300000	144.51120000	0.00000000
70	1251.55850000	12.58690000	0.00000000
71	1271.87900000	25.84670000	0.00000000
72	1277.72420000	78.68680000	0.00000000
73	1283.93960000	24.76750000	0.00000000
74	1297.42430000	48.28560000	0.00000000
75	1307.91710000	1.65890000	0.00000000
76	1339.76610000	13.04040000	0.00000000
77	1350.20740000	2.76380000	0.00000000
78	1356.16710000	3.70000000	0.00000000
79	1368.89920000	7.52650000	0.00000000
80	1396.91810000	42.74210000	0.00000000
81	1399.51930000	25.61690000	0.00000000
82	1402.09170000	11.85820000	0.00000000
83	1408.96060000	16.54140000	0.00000000
84	1425.47030000	17.10870000	0.00000000
85	1452.78500000	4.51110000	0.00000000
86	1463.89070000	7.56770000	0.00000000
87	1467.45520000	0.35390000	0.00000000
88	1478.67490000	11.12470000	0.00000000
89	1483.14210000	0.02790000	0.00000000
90	1484.11640000	1.37570000	0.00000000
91	1488.99970000	17.34530000	0.00000000
92	1492.62760000	13.52950000	0.00000000
93	1497.03910000	3.58940000	0.00000000
94	1503.24400000	5.70480000	0.00000000
95	1515.70710000	22.76620000	0.00000000
96	1533.60560000	12.09980000	0.00000000
97	1628.95930000	0.67030000	0.00000000
98	1648.38660000	2.04990000	0.00000000
99	1714.00030000	53.15130000	0.00000000
100	1762.34840000	504.90030000	0.00000000
101	2959.22230000	49.26570000	0.00000000
102	2982.38180000	67.53720000	0.00000000
103	3022.00090000	31.16210000	0.00000000
104	3032.65310000	35.06550000	0.00000000
105	3042.58560000	16.72340000	0.00000000
106	3046.55090000	32.59210000	0.00000000

107	3053.10670000	18.00930000	0.00000000
108	3083.08500000	45.32810000	0.00000000
109	3086.66820000	43.90960000	0.00000000
110	3091.62700000	24.30400000	0.00000000
111	3105.34650000	11.67880000	0.00000000
112	3109.51670000	8.44340000	0.00000000
113	3112.88400000	47.70500000	0.00000000
114	3118.64150000	63.48340000	0.00000000
115	3130.26630000	16.50410000	0.00000000
116	3143.71740000	2.68810000	0.00000000
117	3146.76130000	22.35930000	0.00000000
118	3166.04800000	5.82570000	0.00000000
119	3172.92640000	0.97550000	0.00000000
120	3181.98550000	26.93430000	0.00000000
121	3191.54270000	36.17120000	0.00000000
122	3198.47670000	10.69830000	0.00000000
123	3209.70780000	20.52620000	0.00000000

Equatorial Clockwise Transition State Re-Optimized

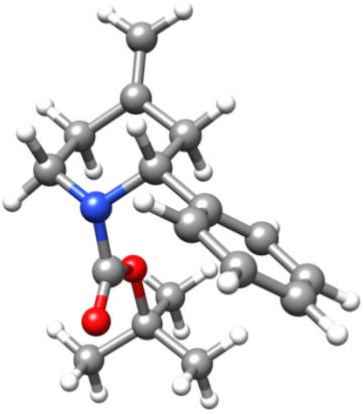

Route : # opt freq b3lyp scrf=(solvent=thf) geom=connectivity def2tzvp empiricaldispersion=gd3bj int=ultrafine pop=(regular,mk)

SMILES : C=C2CCN(C(=O)OC(C)(C)C)C(c1ccccc1)C2

Formula : $C_{17}H_{23}NO_2$

Charge : 0

Multiplicity : 1

Dipole : 3.1521 Debye

Energy : -867.276495435 a.u.

Gibbs Energy : -866.953764 a.u.

Number of imaginary frequencies : 1

Cartesian Coordinates (XYZ format)

43

C	-2.45954600	1.87277700	0.32747800
C	-1.61477400	1.47622400	1.55014300
C	0.45822800	1.55169800	0.22947900
C	-0.34904900	1.89881300	-1.03814700
H	-1.36519300	2.38008800	2.11302500
H	-2.18307800	0.82706500	2.21689200
H	-2.85322800	0.96590600	-0.13705400
H	-3.30468500	2.48128400	0.65387200
H	0.70049800	2.50161900	0.71692800
H	0.26552400	2.51089500	-1.69992000
H	-0.60593600	0.97811000	-1.56575000
C	-1.61368900	2.61801700	-0.66415800
C	-1.92193600	3.82745400	-1.12543600
H	-2.83149700	4.33265500	-0.82288200
H	-1.27830900	4.34613400	-1.82608400
N	-0.34277000	0.81429500	1.23433700
C	-0.42022300	-0.59498900	1.05379600
O	0.15794500	-1.37843300	1.76466800
O	-1.20987500	-0.92983300	0.02738000

C	-1.48609800	-2.33770600	-0.34072500
C	-0.19150500	-3.03283400	-0.74102700
H	0.48619100	-3.12859300	0.10281200
H	-0.42646700	-4.02930200	-1.11777100
H	0.30926500	-2.47695700	-1.53372300
C	-2.19633700	-3.03729000	0.81063900
H	-2.52377900	-4.02428300	0.48130000
H	-1.53976600	-3.15773800	1.66850700
H	-3.07787800	-2.46975100	1.11236000
C	-2.41197300	-2.18672000	-1.53985800
H	-1.91636600	-1.63338900	-2.33805400
H	-2.68786500	-3.17065700	-1.91964400
H	-3.32167300	-1.65497700	-1.25963800
C	1.76303700	0.83081400	-0.03166600
C	1.98036000	0.05802700	-1.16898500
C	2.77724200	0.91387300	0.92353100
C	3.18219700	-0.62089800	-1.34731600
H	1.21298700	-0.02437000	-1.92522000
C	3.97751200	0.24055800	0.74997400
H	2.61397000	1.50730200	1.81470700
C	4.18308200	-0.53313800	-0.38918800
H	3.33239900	-1.21944100	-2.23664800
H	4.75262000	0.31713800	1.50164800
H	5.11793500	-1.06074400	-0.52761500

Frequencies

Mode	IR frequency	IR intensity	Raman intensity
1	-43.55750000	1.22530000	0.00000000
2	27.49670000	0.02790000	0.00000000
3	42.04470000	0.45600000	0.00000000
4	54.28840000	0.13690000	0.00000000
5	73.42250000	2.03530000	0.00000000
6	99.11210000	0.06530000	0.00000000
7	109.37080000	1.13150000	0.00000000
8	130.55660000	1.61310000	0.00000000
9	167.12840000	2.89180000	0.00000000
10	188.51880000	2.88820000	0.00000000
11	201.12330000	0.34030000	0.00000000
12	208.88270000	0.41460000	0.00000000
13	246.69360000	0.57830000	0.00000000
14	260.26260000	1.37140000	0.00000000
15	272.22480000	0.07660000	0.00000000
16	279.90700000	0.27290000	0.00000000
17	323.62200000	7.21350000	0.00000000
18	328.08850000	0.35390000	0.00000000
19	356.50670000	0.83810000	0.00000000
20	365.17370000	2.25960000	0.00000000
21	394.80640000	4.24150000	0.00000000

22	414.12130000	0.15180000	0.00000000
23	418.24070000	0.60610000	0.00000000
24	433.22280000	1.87710000	0.00000000
25	457.18530000	7.51370000	0.00000000
26	473.50990000	2.24380000	0.00000000
27	490.53090000	7.20430000	0.00000000
28	522.25190000	10.04710000	0.00000000
29	565.76800000	5.86380000	0.00000000
30	612.64990000	15.68930000	0.00000000
31	630.13510000	13.98290000	0.00000000
32	638.82640000	1.79480000	0.00000000
33	680.53840000	35.98380000	0.00000000
34	712.37400000	66.13550000	0.00000000
35	732.38320000	1.02790000	0.00000000
36	768.08430000	47.15980000	0.00000000
37	778.88280000	7.96020000	0.00000000
38	793.48070000	1.91280000	0.00000000
39	807.88350000	5.83560000	0.00000000
40	832.73290000	2.97120000	0.00000000
41	854.53300000	55.34800000	0.00000000
42	857.79410000	0.39300000	0.00000000
43	865.07590000	12.08790000	0.00000000
44	915.57850000	59.63140000	0.00000000
45	925.29670000	5.35910000	0.00000000
46	932.30310000	0.31480000	0.00000000
47	933.49310000	0.06810000	0.00000000
48	943.66090000	12.73690000	0.00000000
49	954.11380000	5.76170000	0.00000000
50	974.23640000	0.06830000	0.00000000
51	978.80900000	0.24020000	0.00000000
52	983.43840000	12.79900000	0.00000000
53	993.85670000	1.62290000	0.00000000
54	1006.74480000	7.76670000	0.00000000
55	1016.94030000	38.08610000	0.00000000
56	1026.70680000	1.57300000	0.00000000
57	1049.63490000	37.95240000	0.00000000
58	1054.39490000	1.74500000	0.00000000
59	1058.79420000	7.26680000	0.00000000
60	1063.89020000	10.30190000	0.00000000
61	1108.81390000	24.19110000	0.00000000
62	1122.69550000	111.72410000	0.00000000
63	1134.69170000	87.36940000	0.00000000
64	1151.00610000	677.38790000	0.00000000

65	1181.20220000	0.04520000	0.00000000
66	1191.76110000	11.93480000	0.00000000
67	1206.43390000	3.51460000	0.00000000
68	1222.91660000	11.37360000	0.00000000
69	1233.46940000	61.09110000	0.00000000
70	1254.32870000	11.12180000	0.00000000
71	1272.62380000	25.79670000	0.00000000
72	1281.84680000	8.96320000	0.00000000
73	1283.42500000	18.84460000	0.00000000
74	1299.86020000	49.61730000	0.00000000
75	1306.17410000	23.14810000	0.00000000
76	1342.13010000	3.11710000	0.00000000
77	1349.91380000	7.83080000	0.00000000
78	1357.44580000	2.68220000	0.00000000
79	1382.61080000	24.60660000	0.00000000
80	1390.54990000	51.27420000	0.00000000
81	1400.19530000	25.99290000	0.00000000
82	1402.57680000	16.84250000	0.00000000
83	1409.11140000	47.58000000	0.00000000
84	1424.94590000	19.13440000	0.00000000
85	1452.92880000	0.58790000	0.00000000
86	1468.09160000	1.56420000	0.00000000
87	1468.76570000	9.84700000	0.00000000
88	1482.61690000	10.14690000	0.00000000
89	1484.04640000	15.87120000	0.00000000
90	1485.00810000	1.35750000	0.00000000
91	1488.04540000	13.64210000	0.00000000
92	1489.45000000	2.79600000	0.00000000
93	1490.45890000	16.88350000	0.00000000
94	1497.39230000	3.80900000	0.00000000
95	1515.78960000	24.23540000	0.00000000
96	1535.26060000	11.67470000	0.00000000
97	1628.26770000	2.21110000	0.00000000
98	1648.79480000	2.06420000	0.00000000
99	1709.40250000	72.67880000	0.00000000
100	1755.98490000	538.78110000	0.00000000
101	3010.69150000	30.14110000	0.00000000
102	3025.24060000	57.76750000	0.00000000
103	3041.56550000	31.45630000	0.00000000
104	3042.62320000	19.30020000	0.00000000
105	3045.46900000	17.82330000	0.00000000
106	3046.57920000	33.52120000	0.00000000
107	3053.98530000	18.79750000	0.00000000

108	3083.48340000	46.42970000	0.00000000
109	3090.17650000	19.38330000	0.00000000
110	3091.33050000	51.79810000	0.00000000
111	3105.73280000	13.21500000	0.00000000
112	3109.63950000	4.41830000	0.00000000
113	3112.95840000	47.74350000	0.00000000
114	3118.57130000	69.00070000	0.00000000
115	3128.96180000	16.55610000	0.00000000
116	3142.47140000	8.70530000	0.00000000
117	3149.33170000	15.98330000	0.00000000
118	3167.72170000	3.47740000	0.00000000
119	3174.17500000	1.86610000	0.00000000
120	3183.41830000	29.21860000	0.00000000
121	3193.98510000	34.64070000	0.00000000
122	3203.56560000	10.60060000	0.00000000
123	3208.19560000	22.23090000	0.00000000

Equatorial Anticlockwise Transition State Re-Optimized

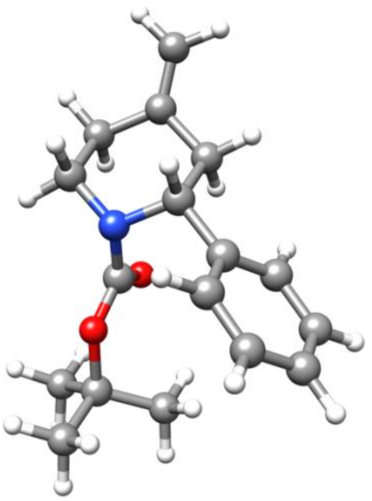

Route : # opt freq b3lyp scrf=(solvent=thf) geom=connectivity def2tzvp empiricaldispersion=gd3bj int=ultrafine pop=(regular,mk)

SMILES : C=C2CCN(C(=O)OC(C)(C)C)C(c1ccccc1)C2

Formula : $C_{17}H_{23}NO_2$

Charge : 0

Multiplicity : 1

Dipole : 2.0699 Debye

Energy : -867.277898089 a.u.

Gibbs Energy : -866.954363 a.u.

Number of imaginary frequencies : 1

Cartesian Coordinates (XYZ format)

43

C	-3.07647900	-1.62868300	0.03361200
C	-2.00625900	-1.55150600	1.13494100
C	-1.43258100	0.75266300	0.50356700
C	-2.47889700	0.72396400	-0.62679000
H	-2.47653900	-1.23349700	2.06932900
H	-1.56024600	-2.53102200	1.31137100
H	-2.62816000	-2.07666700	-0.85724000
H	-3.89472200	-2.27173900	0.36219300
H	-1.94895600	1.09393400	1.40716100
H	-2.88414300	1.72615700	-0.77461800
H	-1.99187100	0.40955600	-1.55225200
C	-3.57365400	-0.24990900	-0.29374800
C	-4.85763300	0.09523100	-0.23987200
H	-5.62578700	-0.62390900	0.01877300
H	-5.18109100	1.10536400	-0.46179200
N	-0.92835100	-0.59537700	0.85459500
C	0.05454900	-1.08630400	-0.05576800

O	-0.13111200	-1.31177600	-1.23053500
O	1.20477600	-1.25321800	0.58695000
C	2.44822100	-1.64058900	-0.11702400
C	2.29874300	-3.03442800	-0.71272500
H	1.57175700	-3.04534400	-1.52047200
H	3.26305900	-3.35921300	-1.10609700
H	1.98911100	-3.74405300	0.05602100
C	2.79071700	-0.58385000	-1.16033300
H	3.78687000	-0.78600200	-1.55635300
H	2.08149700	-0.59076800	-1.98365900
H	2.79439300	0.40770400	-0.70682900
C	3.47370700	-1.63740300	1.00838500
H	3.19567800	-2.35255800	1.78312400
H	4.45282600	-1.91312300	0.61634200
H	3.54435700	-0.64620700	1.45691000
C	-0.25837000	1.68263200	0.26913700
C	0.04211600	2.22334300	-0.97752100
C	0.58158400	1.97789000	1.34530200
C	1.15742200	3.03935000	-1.14793800
H	-0.58522900	2.00715700	-1.83013700
C	1.69468100	2.78825900	1.18002500
H	0.36284500	1.55000800	2.31538300
C	1.98776800	3.32229900	-0.07233800
H	1.37719300	3.44748000	-2.12608500
H	2.33546700	3.00330200	2.02557800
H	2.85703600	3.95329600	-0.20558300

Frequencies

Mode	IR frequency	IR intensity	Raman intensity
1	-36.28790000	2.05310000	0.00000000
2	37.24280000	0.08640000	0.00000000
3	49.74100000	0.29480000	0.00000000
4	61.86440000	0.57400000	0.00000000
5	71.24450000	1.21260000	0.00000000
6	109.41120000	0.67950000	0.00000000
7	114.68320000	0.41520000	0.00000000
8	140.34340000	1.78870000	0.00000000
9	162.81490000	3.85650000	0.00000000
10	187.13370000	1.09280000	0.00000000
11	202.32970000	2.83310000	0.00000000
12	208.36590000	0.28120000	0.00000000
13	244.73410000	2.31520000	0.00000000
14	253.06100000	2.48280000	0.00000000
15	272.68680000	0.12720000	0.00000000
16	295.43020000	2.75540000	0.00000000
17	306.50560000	2.81630000	0.00000000
18	325.41930000	10.49390000	0.00000000
19	343.39340000	0.81660000	0.00000000

20	363.83060000	2.52690000	0.00000000
21	403.74110000	2.55460000	0.00000000
22	413.71490000	0.13760000	0.00000000
23	420.74010000	0.70130000	0.00000000
24	434.04670000	5.72880000	0.00000000
25	460.99460000	1.26950000	0.00000000
26	474.58600000	0.97420000	0.00000000
27	486.88530000	7.34920000	0.00000000
28	519.54160000	9.98000000	0.00000000
29	565.56850000	3.17100000	0.00000000
30	605.90480000	14.68880000	0.00000000
31	638.04990000	0.60350000	0.00000000
32	658.94680000	7.04080000	0.00000000
33	698.14420000	55.51060000	0.00000000
34	713.99140000	74.42750000	0.00000000
35	732.89530000	1.37990000	0.00000000
36	757.87930000	28.34230000	0.00000000
37	776.35480000	19.28570000	0.00000000
38	796.20120000	4.23400000	0.00000000
39	811.90100000	12.80450000	0.00000000
40	833.29830000	0.79140000	0.00000000
41	852.72670000	31.85500000	0.00000000
42	859.40010000	1.00810000	0.00000000
43	864.83070000	19.26960000	0.00000000
44	915.66040000	60.64890000	0.00000000
45	925.03390000	5.71840000	0.00000000
46	932.67220000	0.29930000	0.00000000
47	933.90050000	0.11840000	0.00000000
48	941.77150000	9.98540000	0.00000000
49	958.82090000	10.02800000	0.00000000
50	974.33460000	0.12950000	0.00000000
51	983.15540000	0.38010000	0.00000000
52	987.10690000	24.10100000	0.00000000
53	1003.13710000	3.34600000	0.00000000
54	1007.19090000	4.12040000	0.00000000
55	1020.64780000	38.80560000	0.00000000
56	1026.78400000	1.12500000	0.00000000
57	1051.09410000	23.51510000	0.00000000
58	1053.55380000	1.52700000	0.00000000
59	1059.54260000	4.23210000	0.00000000
60	1064.84910000	3.34920000	0.00000000
61	1110.97630000	20.26700000	0.00000000
62	1128.04780000	51.43140000	0.00000000

63	1134.77840000	91.13630000	0.00000000
64	1175.25910000	681.85100000	0.00000000
65	1181.33980000	0.17950000	0.00000000
66	1191.33540000	10.17590000	0.00000000
67	1207.57890000	3.99950000	0.00000000
68	1221.24570000	8.66970000	0.00000000
69	1252.25000000	33.47820000	0.00000000
70	1260.09310000	372.56950000	0.00000000
71	1272.16790000	25.28810000	0.00000000
72	1280.96240000	10.59340000	0.00000000
73	1283.78560000	13.75040000	0.00000000
74	1296.42570000	50.57480000	0.00000000
75	1304.25800000	4.10520000	0.00000000
76	1342.46550000	3.45940000	0.00000000
77	1348.39500000	15.08020000	0.00000000
78	1358.68630000	3.97410000	0.00000000
79	1380.27810000	11.78640000	0.00000000
80	1388.51230000	36.16720000	0.00000000
81	1399.25420000	28.84380000	0.00000000
82	1401.49220000	36.48890000	0.00000000
83	1405.26040000	70.77100000	0.00000000
84	1425.36940000	20.36770000	0.00000000
85	1451.55430000	5.01230000	0.00000000
86	1468.16750000	11.01480000	0.00000000
87	1468.62230000	0.55320000	0.00000000
88	1483.55060000	17.47110000	0.00000000
89	1484.56480000	0.48250000	0.00000000
90	1485.03750000	1.38040000	0.00000000
91	1487.36000000	15.48730000	0.00000000
92	1488.84540000	7.67330000	0.00000000
93	1490.21620000	14.36330000	0.00000000
94	1497.81580000	2.87930000	0.00000000
95	1517.12830000	24.13950000	0.00000000
96	1535.59420000	14.79500000	0.00000000
97	1627.76810000	2.86190000	0.00000000
98	1648.62060000	3.63680000	0.00000000
99	1709.32370000	59.19490000	0.00000000
100	1727.93210000	354.39160000	0.00000000
101	3007.13080000	27.60430000	0.00000000
102	3027.98990000	53.98430000	0.00000000
103	3035.28300000	24.97730000	0.00000000
104	3042.47400000	17.39470000	0.00000000
105	3043.83100000	22.28220000	0.00000000

106	3045.98350000	29.71080000	0.00000000
107	3052.56700000	23.91970000	0.00000000
108	3081.09680000	53.36990000	0.00000000
109	3087.53080000	35.11190000	0.00000000
110	3090.79380000	31.74670000	0.00000000
111	3104.57240000	13.84260000	0.00000000
112	3110.20040000	2.77120000	0.00000000
113	3113.27760000	43.27270000	0.00000000
114	3118.79710000	57.30710000	0.00000000
115	3128.52420000	16.93260000	0.00000000
116	3143.10270000	1.12020000	0.00000000
117	3146.03960000	26.51310000	0.00000000
118	3168.63970000	1.08180000	0.00000000
119	3176.20050000	4.25420000	0.00000000
120	3184.86650000	30.23030000	0.00000000
121	3194.17050000	29.97500000	0.00000000
122	3206.31400000	9.01640000	0.00000000
123	3207.73580000	22.25460000	0.00000000

Thermochemistry Values

Left Rotamer Axial:

	Temperature		
	298 K	213 K	195 K
ε_0	–867.300243526 a.u.	–867.300243526 a.u.	–867.300243526 a.u.
ε_{ZPE}	0.370384 a.u.	0.370384 a.u.	0.370384 a.u.
E_{tot}	0.389867 a.u.	0.380965 a.u.	0.379439 a.u.
H_{corr}	0.390811 a.u.	0.381640 a.u.	0.380057 a.u.
G_{corr}	0.320714 a.u.	0.339211 a.u.	0.342727 a.u.
S_{tot}	147.533 calmol^{-1}K^{-1}	124.998 calmol^{-1}K^{-1}	120.127 calmol^{-1}K^{-1}
$\varepsilon_0 + \varepsilon_{ZPE}$	–866.929859 a.u.	–866.929860 a.u.	–866.929860 a.u.
$\varepsilon_0 + E_{tot}$	–866.910376 a.u.	–866.919279 a.u.	–866.920805 a.u.
$\varepsilon_0 + H_{corr}$	–866.909432 a.u.	–866.918604 a.u.	–866.920187 a.u.
$\varepsilon_0 + G_{corr}$	–866.979530 a.u.	–866.961033 a.u.	–866.957517 a.u.

Right Rotamer Axial:

	Temperature		
	298 K	223 K	195 K
ε_0	–867.300166083 a.u.	–867.300166083 a.u.	–867.300166083 a.u.
ε_{ZPE}	0.370126 a.u.	0.370126 a.u.	0.370126 a.u.
E_{tot}	0.389725 a.u.	0.380814 a.u.	0.379285 a.u.
H_{corr}	0.390669 a.u.	0.381488 a.u.	0.379903 a.u.
G_{corr}	0.317101 a.u.	0.336588 a.u.	0.340312 a.u.
S_{tot}	154.836 calmol^{-1}K^{-1}	132.279 calmol^{-1}K^{-1}	127.402 calmol^{-1}K^{-1}
$\varepsilon_0 + \varepsilon_{ZPE}$	–866.930040 a.u.	–866.930040 a.u.	–866.930040 a.u.
$\varepsilon_0 + E_{tot}$	–866.910441 a.u.	–866.919352 a.u.	–866.920881 a.u.
$\varepsilon_0 + H_{corr}$	–866.909497 a.u.	–866.918678 a.u.	–866.920263 a.u.
$\varepsilon_0 + G_{corr}$	–866.983065 a.u.	–866.963578 a.u.	–866.959854 a.u.

Left Rotamer Equatorial:

	Temperature	
	298 K	195 K
ε_0	–867.295403418 a.u.	–867.295403418 a.u.
ε_{ZPE}	0.369956 a.u.	0.369956 a.u.
E_{tot}	0.389434 a.u.	0.378969 a.u.
H_{corr}	0.390379 a.u.	0.379587 a.u.
G_{corr}	0.321920 a.u.	0.343360 a.u.
S_{tot}	144.083 calmol^{-1}K^{-1}	116.578 calmol^{-1}K^{-1}
$\varepsilon_0 + \varepsilon_{ZPE}$	–866.925448 a.u.	–866.925447 a.u.
$\varepsilon_0 + E_{tot}$	–866.905969 a.u.	–866.916434 a.u.
$\varepsilon_0 + H_{corr}$	–866.905025 a.u.	–866.915816 a.u.
$\varepsilon_0 + G_{corr}$	–866.973483 a.u.	–866.952043 a.u.

Right Rotamer Equatorial:

	Temperature	
	298 K	195 K
ε_0	–867.292815979 a.u.	–867.292815979 a.u.
ε_{ZPE}	0.369571 a.u.	0.369571 a.u.
E_{tot}	0.389259 a.u.	0.378763 a.u.
H_{corr}	0.390203 a.u.	0.379380 a.u.
G_{corr}	0.320236 a.u.	0.342192 a.u.
S_{tot}	147.258 calmol^{-1}K^{-1}	119.671 calmol^{-1}K^{-1}
$\varepsilon_0 + \varepsilon_{ZPE}$	–866.923245 a.u.	–866.923245 a.u.
$\varepsilon_0 + E_{tot}$	–866.903557 a.u.	–866.914053 a.u.
$\varepsilon_0 + H_{corr}$	–866.902613 a.u.	–866.913436 a.u.
$\varepsilon_0 + G_{corr}$	–866.972580 a.u.	–866.950624 a.u.

Left Rotamer Equatorial Re-Optimized:

	Temperature	
	298 K	195 K
ε_0	–867.296096363 a.u.	–867.296096363 a.u.
ε_{ZPE}	0.370295 a.u.	0.370295 a.u.
E_{tot}	0.389628 a.u.	0.379210 a.u.
H_{corr}	0.390572 a.u.	0.379828 a.u.
G_{corr}	0.322547 a.u.	0.343845 a.u.
S_{tot}	143.171 calmol^{-1}K^{-1}	115.792 calmol^{-1}K^{-1}
$\varepsilon_0 + \varepsilon_{ZPE}$	–866.925801 a.u.	–866.925801 a.u.
$\varepsilon_0 + E_{tot}$	–866.906468 a.u.	–866.916886 a.u.
$\varepsilon_0 + H_{corr}$	–866.905524 a.u.	–866.916268 a.u.
$\varepsilon_0 + G_{corr}$	–866.973549 a.u.	–866.952251 a.u.

Right Rotamer Equatorial Re-Optimized:

	Temperature	
	298 K	195 K
ε_0	–867.293496677 a.u.	–867.293496677 a.u.
ε_{ZPE}	0.370033 a.u.	0.370033 a.u.
E_{tot}	0.389484 a.u.	0.379051 a.u.
H_{corr}	0.390428 a.u.	0.379668 a.u.
G_{corr}	0.321322 a.u.	0.342991 a.u.
S_{tot}	145.446 calmol^{-1}K^{-1}	118.028 calmol^{-1}K^{-1}
$\varepsilon_0 + \varepsilon_{ZPE}$	–866.923464 a.u.	–866.923464 a.u.
$\varepsilon_0 + E_{tot}$	–866.904013 a.u.	–866.914446 a.u.
$\varepsilon_0 + H_{corr}$	–866.903069 a.u.	–866.913829 a.u.
$\varepsilon_0 + G_{corr}$	–866.972175 a.u.	–866.950506 a.u.

Axial Clockwise Transition State:

	Temperature	
	298 K	195 K
ε_0	–867.274004513 a.u.	–867.274004513 a.u.
ε_{ZPE}	0.369562 a.u.	0.369562 a.u.
E_{tot}	0.388146 a.u.	0.378026 a.u.
H_{corr}	0.389090 a.u.	0.378644 a.u.
G_{corr}	0.322532 a.u.	0.343381 a.u.
S_{tot}	140.084 calmol^{-1}K^{-1}	113.477 calmol^{-1}K^{-1}
$\varepsilon_0 + \varepsilon_{ZPE}$	–866.904443 a.u.	–866.904443 a.u.
$\varepsilon_0 + E_{tot}$	–866.885859 a.u.	–866.895979 a.u.
$\varepsilon_0 + H_{corr}$	–866.884914 a.u.	–866.895361 a.u.
$\varepsilon_0 + G_{corr}$	–866.951473 a.u.	–866.930624 a.u.

Axial Anticlockwise Transition State:

	Temperature	
	298 K	195 K
ε_0	–867.273949768 a.u.	–867.273949768 a.u.
ε_{ZPE}	0.369571 a.u.	0.369571 a.u.
E_{tot}	0.388153 a.u.	0.378034 a.u.
H_{corr}	0.389097 a.u.	0.378651 a.u.
G_{corr}	0.322809 a.u.	0.343564 a.u.
S_{tot}	139.515 calmol^{-1}K^{-1}	112.910 calmol^{-1}K^{-1}
$\varepsilon_0 + \varepsilon_{ZPE}$	–866.904379 a.u.	–866.904379 a.u.
$\varepsilon_0 + E_{tot}$	–866.885797 a.u.	–866.895916 a.u.
$\varepsilon_0 + H_{corr}$	–866.884853 a.u.	–866.895299 a.u.
$\varepsilon_0 + G_{corr}$	–866.951141 a.u.	–866.930386 a.u.

Equatorial Clockwise Transition State:

	Temperature	
	298 K	195 K
ε_0	–867.282502567 a.u.	–867.282502567 a.u.
ε_{ZPE}	0.369283 a.u.	0.369283 a.u.
E_{tot}	0.387984 a.u.	0.377829 a.u.
H_{corr}	0.388928 a.u.	0.378447 a.u.
G_{corr}	0.322839 a.u.	0.343520 a.u.
S_{tot}	139.095 calmol^{-1}K^{-1}	112.393 calmol^{-1}K^{-1}
$\varepsilon_0 + \varepsilon_{ZPE}$	–866.913219 a.u.	–866.913220 a.u.
$\varepsilon_0 + E_{tot}$	–866.894519 a.u.	–866.904674 a.u.
$\varepsilon_0 + H_{corr}$	–866.893575 a.u.	–866.904056 a.u.
$\varepsilon_0 + G_{corr}$	–866.959664 a.u.	–866.938983 a.u.

Equatorial Anticlockwise Transition State:

	Temperature	
	298 K	195 K
ε_0	–867.280856122 a.u.	–867.280856122 a.u.
ε_{ZPE}	0.369200 a.u.	0.369200 a.u.
E_{tot}	0.387933 a.u.	0.377775 a.u.
H_{corr}	0.388877 a.u.	0.378393 a.u.
G_{corr}	0.322381 a.u.	0.343203 a.u.
S_{tot}	139.953 calmol^{-1}K^{-1}	113.243 calmol^{-1}K^{-1}
$\varepsilon_0 + \varepsilon_{ZPE}$	–866.911656 a.u.	–866.911656 a.u.
$\varepsilon_0 + E_{tot}$	–866.892923 a.u.	–866.903081 a.u.
$\varepsilon_0 + H_{corr}$	–866.891979 a.u.	–866.902463 a.u.
$\varepsilon_0 + G_{corr}$	–866.958475 a.u.	–866.937653 a.u.

Equatorial Clockwise Transition State Re-Optimized:

	Temperature	
	298 K	195 K
ε_0	–867.276495435 a.u.	–867.276495435 a.u.
ε_{ZPE}	0.369342 a.u.	0.369342 a.u.
E_{tot}	0.387999 a.u.	0.377845 a.u.
H_{corr}	0.388944 a.u.	0.378462 a.u.
G_{corr}	0.322732 a.u.	0.343454 a.u.
S_{tot}	139.354 calmol^{-1}K^{-1}	112.656 calmol^{-1}K^{-1}
$\varepsilon_0 + \varepsilon_{ZPE}$	–866.907154 a.u.	–866.907153 a.u.
$\varepsilon_0 + E_{tot}$	–866.888496 a.u.	–866.898650 a.u.
$\varepsilon_0 + H_{corr}$	–866.887552 a.u.	–866.898033 a.u.
$\varepsilon_0 + G_{corr}$	–866.953764 a.u.	–866.933041 a.u.

Equatorial Anticlockwise Transition State Re-Optimized:

	Temperature	
	298 K	195 K
ε_0	–867.277898089 a.u.	–867.277898089 a.u.
ε_{ZPE}	0.369540 a.u.	0.369540 a.u.
E_{tot}	0.388109 a.u.	0.377970 a.u.
H_{corr}	0.389053 a.u.	0.378588 a.u.
G_{corr}	0.323535 a.u.	0.344021 a.u.
S_{tot}	137.895 calmol^{-1}K^{-1}	111.237 calmol^{-1}K^{-1}
$\varepsilon_0 + \varepsilon_{ZPE}$	–866.908358 a.u.	–866.908358 a.u.
$\varepsilon_0 + E_{tot}$	–866.889789 a.u.	–866.899928 a.u.
$\varepsilon_0 + H_{corr}$	–866.888845 a.u.	–866.899310 a.u.
$\varepsilon_0 + G_{corr}$	–866.954363 a.u.	–866.933877 a.u.
